# Supplementary material for: A hepatocyte-specific transcriptional program driven by Rela and Stat3 exacerbates experimental colitis in mice by modulating bile synthesis
Source: eLife. 2024 Aug 13;12:RP93273. doi: 10.7554/eLife.93273 (PMC11321761; doi:10.7554/eLife.93273)
Supplement: Figure 1—source data 1. [file elife-93273-fig1-data1.docx]

|  | **ID** | **Description** | **setSize** | **enrichmentScore** | **NES** | **pvalue** | **p.adjust** | **qvalues** |
| --- | --- | --- | --- | --- | --- | --- | --- | --- |
| **GO:0006950** | GO:0006950 | response to stress | 244 | 0.3934916821 | 2.152229755 | 0.0001179801793 | 0.0001179801793 | 0.008825839003 |
| **GO:0002376** | GO:0002376 | immune system process | 211 | 0.4694103894 | 2.529592767 | 0.0001204093919 | 0.0001204093919 | 0.008825839003 |
| **GO:0009605** | GO:0009605 | response to external stimulus | 211 | 0.4514637325 | 2.43288052 | 0.0001204093919 | 0.0001204093919 | 0.008825839003 |
| **GO:0006955** | GO:0006955 | immune response | 155 | 0.5155549435 | 2.668932996 | 0.0001252975818 | 0.0001252975818 | 0.008825839003 |
| **GO:0006952** | GO:0006952 | defense response | 145 | 0.5151021406 | 2.64955825 | 0.0001255492781 | 0.0001255492781 | 0.008825839003 |
| **GO:0009607** | GO:0009607 | response to biotic stimulus | 145 | 0.5217009009 | 2.683500644 | 0.0001255492781 | 0.0001255492781 | 0.008825839003 |
| **GO:0044419** | GO:0044419 | biological process involved in interspecies interaction between organisms | 149 | 0.5238408872 | 2.700696786 | 0.0001257071025 | 0.0001257071025 | 0.008825839003 |
| **GO:0043207** | GO:0043207 | response to external biotic stimulus | 143 | 0.5266513781 | 2.70103379 | 0.0001263104711 | 0.0001263104711 | 0.008825839003 |
| **GO:0051707** | GO:0051707 | response to other organism | 143 | 0.5266513781 | 2.70103379 | 0.0001263104711 | 0.0001263104711 | 0.008825839003 |
| **GO:0002682** | GO:0002682 | regulation of immune system process | 123 | 0.4486329757 | 2.256070536 | 0.0001290489095 | 0.0001290489095 | 0.008825839003 |
| **GO:0080134** | GO:0080134 | regulation of response to stress | 117 | 0.3825284729 | 1.907434507 | 0.0001299545159 | 0.0001299545159 | 0.008825839003 |
| **GO:0098542** | GO:0098542 | defense response to other organism | 112 | 0.5481146476 | 2.713004584 | 0.0001304121022 | 0.0001304121022 | 0.008825839003 |
| **GO:0032101** | GO:0032101 | regulation of response to external stimulus | 103 | 0.471455542 | 2.299019077 | 0.0001320480655 | 0.0001320480655 | 0.008825839003 |
| **GO:0045087** | GO:0045087 | innate immune response | 90 | 0.5392124067 | 2.575957286 | 0.0001346076188 | 0.0001346076188 | 0.008825839003 |
| **GO:0050776** | GO:0050776 | regulation of immune response | 84 | 0.4898024596 | 2.307544398 | 0.0001362026696 | 0.0001362026696 | 0.008825839003 |
| **GO:0002684** | GO:0002684 | positive regulation of immune system process | 80 | 0.44672857 | 2.086409305 | 0.0001367240908 | 0.0001367240908 | 0.008825839003 |
| **GO:0034097** | GO:0034097 | response to cytokine | 82 | 0.5342340233 | 2.503293069 | 0.0001369112815 | 0.0001369112815 | 0.008825839003 |
| **GO:0010628** | GO:0010628 | positive regulation of gene expression | 77 | 0.4398489995 | 2.04000583 | 0.000137136588 | 0.000137136588 | 0.008825839003 |
| **GO:0009617** | GO:0009617 | response to bacterium | 76 | 0.5176820533 | 2.396095395 | 0.0001372306848 | 0.0001372306848 | 0.008825839003 |
| **GO:0071345** | GO:0071345 | cellular response to cytokine stimulus | 71 | 0.5022989702 | 2.293186403 | 0.0001382552191 | 0.0001382552191 | 0.008825839003 |
| **GO:0031347** | GO:0031347 | regulation of defense response | 69 | 0.4633657719 | 2.10477428 | 0.0001391207568 | 0.0001391207568 | 0.008825839003 |
| **GO:0001817** | GO:0001817 | regulation of cytokine production | 66 | 0.4742141999 | 2.136226317 | 0.0001400952648 | 0.0001400952648 | 0.008825839003 |
| **GO:0002831** | GO:0002831 | regulation of response to biotic stimulus | 52 | 0.573529446 | 2.471172091 | 0.0001435338022 | 0.0001435338022 | 0.008825839003 |
| **GO:0009615** | GO:0009615 | response to virus | 52 | 0.5800819965 | 2.499405131 | 0.0001435338022 | 0.0001435338022 | 0.008825839003 |
| **GO:0051607** | GO:0051607 | defense response to virus | 46 | 0.5840971332 | 2.450142217 | 0.0001448435689 | 0.0001448435689 | 0.008825839003 |
| **GO:0140546** | GO:0140546 | defense response to symbiont | 46 | 0.5840971332 | 2.450142217 | 0.0001448435689 | 0.0001448435689 | 0.008825839003 |
| **GO:0002683** | GO:0002683 | negative regulation of immune system process | 40 | 0.5219775386 | 2.117481091 | 0.0001478633742 | 0.0001478633742 | 0.008825839003 |
| **GO:0045088** | GO:0045088 | regulation of innate immune response | 39 | 0.5376115116 | 2.162317349 | 0.0001483459427 | 0.0001483459427 | 0.008825839003 |
| **GO:0032102** | GO:0032102 | negative regulation of response to external stimulus | 38 | 0.53158436 | 2.12664927 | 0.0001486767767 | 0.0001486767767 | 0.008825839003 |
| **GO:0019221** | GO:0019221 | cytokine-mediated signaling pathway | 35 | 0.6097829746 | 2.383647379 | 0.0001509206158 | 0.0001509206158 | 0.008825839003 |
| **GO:0002237** | GO:0002237 | response to molecule of bacterial origin | 34 | 0.5658901785 | 2.19471933 | 0.0001514692517 | 0.0001514692517 | 0.008825839003 |
| **GO:0002833** | GO:0002833 | positive regulation of response to biotic stimulus | 33 | 0.5782179138 | 2.225040832 | 0.0001517680983 | 0.0001517680983 | 0.008825839003 |
| **GO:0002221** | GO:0002221 | pattern recognition receptor signaling pathway | 30 | 0.5963144778 | 2.247291589 | 0.0001528117359 | 0.0001528117359 | 0.008825839003 |
| **GO:0050900** | GO:0050900 | leukocyte migration | 22 | 0.6298418529 | 2.183653476 | 0.0001570845115 | 0.0001570845115 | 0.008825839003 |
| **GO:0035456** | GO:0035456 | response to interferon-beta | 15 | 0.6807887164 | 2.124657793 | 0.0001631587535 | 0.0001631587535 | 0.008825839003 |
| **GO:0097529** | GO:0097529 | myeloid leukocyte migration | 15 | 0.7108724768 | 2.218545507 | 0.0001631587535 | 0.0001631587535 | 0.008825839003 |
| **GO:0019373** | GO:0019373 | epoxygenase P450 pathway | 6 | -0.8693271568 | -2.188027723 | 0.0002294630564 | 0.0002294630564 | 0.009715807382 |
| **GO:0019369** | GO:0019369 | arachidonic acid metabolic process | 7 | -0.8799299607 | -2.336658972 | 0.0002357378595 | 0.0002357378595 | 0.009715807382 |
| **GO:0042221** | GO:0042221 | response to chemical | 248 | 0.2999613171 | 1.643263352 | 0.0002359046945 | 0.0002359046945 | 0.009715807382 |
| **GO:0042178** | GO:0042178 | xenobiotic catabolic process | 8 | -0.8498155253 | -2.36779301 | 0.0002379819134 | 0.0002379819134 | 0.009715807382 |
| **GO:0006805** | GO:0006805 | xenobiotic metabolic process | 10 | -0.7833898358 | -2.392098188 | 0.0002421893921 | 0.0002421893921 | 0.009715807382 |
| **GO:0048584** | GO:0048584 | positive regulation of response to stimulus | 169 | 0.3365670103 | 1.762885682 | 0.0002471882338 | 0.0002471882338 | 0.009715807382 |
| **GO:0000070** | GO:0000070 | mitotic sister chromatid segregation | 13 | -0.7242803517 | -2.415299474 | 0.0002523977789 | 0.0002523977789 | 0.009715807382 |
| **GO:0006690** | GO:0006690 | icosanoid metabolic process | 13 | -0.7439100597 | -2.480759794 | 0.0002523977789 | 0.0002523977789 | 0.009715807382 |
| **GO:0033559** | GO:0033559 | unsaturated fatty acid metabolic process | 13 | -0.8042289127 | -2.681908553 | 0.0002523977789 | 0.0002523977789 | 0.009715807382 |
| **GO:0071466** | GO:0071466 | cellular response to xenobiotic stimulus | 13 | -0.7392438012 | -2.465198952 | 0.0002523977789 | 0.0002523977789 | 0.009715807382 |
| **GO:0120254** | GO:0120254 | olefinic compound metabolic process | 13 | -0.6911550461 | -2.304834606 | 0.0002523977789 | 0.0002523977789 | 0.009715807382 |
| **GO:0000819** | GO:0000819 | sister chromatid segregation | 14 | -0.7330260528 | -2.494498806 | 0.0002555583951 | 0.0002555583951 | 0.009715807382 |
| **GO:0098813** | GO:0098813 | nuclear chromosome segregation | 17 | -0.7118131979 | -2.566884035 | 0.0002629503024 | 0.0002629503024 | 0.009715807382 |
| **GO:0140014** | GO:0140014 | mitotic nuclear division | 17 | -0.6475567727 | -2.335167635 | 0.0002629503024 | 0.0002629503024 | 0.009715807382 |
| **GO:0007059** | GO:0007059 | chromosome segregation | 19 | -0.7251253268 | -2.709235493 | 0.0002680965147 | 0.0002680965147 | 0.009715807382 |
| **GO:0001816** | GO:0001816 | cytokine production | 70 | 0.4540191427 | 2.069292932 | 0.0002770850651 | 0.0002770850651 | 0.009715807382 |
| **GO:0006631** | GO:0006631 | fatty acid metabolic process | 28 | -0.6361050373 | -2.66458942 | 0.0002883506344 | 0.0002883506344 | 0.009715807382 |
| **GO:0001819** | GO:0001819 | positive regulation of cytokine production | 43 | 0.497955578 | 2.049908373 | 0.0002937288882 | 0.0002937288882 | 0.009715807382 |
| **GO:0051301** | GO:0051301 | cell division | 34 | -0.5356435776 | -2.385074415 | 0.0002941176471 | 0.0002941176471 | 0.009715807382 |
| **GO:0032496** | GO:0032496 | response to lipopolysaccharide | 31 | 0.5533560887 | 2.102685965 | 0.0003047386866 | 0.0003047386866 | 0.009715807382 |
| **GO:1903047** | GO:1903047 | mitotic cell cycle process | 39 | -0.4624416797 | -2.128960628 | 0.0003066544005 | 0.0003066544005 | 0.009715807382 |
| **GO:0032787** | GO:0032787 | monocarboxylic acid metabolic process | 42 | -0.5079024574 | -2.383686192 | 0.0003100775194 | 0.0003100775194 | 0.009715807382 |
| **GO:0000278** | GO:0000278 | mitotic cell cycle | 44 | -0.4591592366 | -2.174664649 | 0.0003164556962 | 0.0003164556962 | 0.009715807382 |
| **GO:0044255** | GO:0044255 | cellular lipid metabolic process | 50 | -0.4399854606 | -2.152945299 | 0.0003263707572 | 0.0003263707572 | 0.009715807382 |
| **GO:0050896** | GO:0050896 | response to stimulus | 446 | 0.2779035508 | 1.584806447 | 0.0003310527477 | 0.0003310527477 | 0.009715807382 |
| **GO:0019752** | GO:0019752 | carboxylic acid metabolic process | 60 | -0.4279866777 | -2.199151158 | 0.0003401360544 | 0.0003401360544 | 0.009715807382 |
| **GO:0006082** | GO:0006082 | organic acid metabolic process | 64 | -0.4253718211 | -2.221412202 | 0.0003421142662 | 0.0003421142662 | 0.009715807382 |
| **GO:0043436** | GO:0043436 | oxoacid metabolic process | 62 | -0.4188841295 | -2.166280202 | 0.0003441156228 | 0.0003441156228 | 0.009715807382 |
| **GO:0071677** | GO:0071677 | positive regulation of mononuclear cell migration | 7 | 0.8363687545 | 2.043748494 | 0.0003471619511 | 0.0003471619511 | 0.009715807382 |
| **GO:1990266** | GO:1990266 | neutrophil migration | 7 | 0.8468095347 | 2.069261557 | 0.0003471619511 | 0.0003471619511 | 0.009715807382 |
| **GO:0048247** | GO:0048247 | lymphocyte chemotaxis | 5 | 0.8919805251 | 1.956759955 | 0.0003540449637 | 0.0003540449637 | 0.009715807382 |
| **GO:1901623** | GO:1901623 | regulation of lymphocyte chemotaxis | 5 | 0.8919805251 | 1.956759955 | 0.0003540449637 | 0.0003540449637 | 0.009715807382 |
| **GO:1990868** | GO:1990868 | response to chemokine | 5 | 0.8942110401 | 1.961653092 | 0.0003540449637 | 0.0003540449637 | 0.009715807382 |
| **GO:1990869** | GO:1990869 | cellular response to chemokine | 5 | 0.8942110401 | 1.961653092 | 0.0003540449637 | 0.0003540449637 | 0.009715807382 |
| **GO:2000401** | GO:2000401 | regulation of lymphocyte migration | 6 | 0.8484576641 | 1.970255287 | 0.0003542330854 | 0.0003542330854 | 0.009715807382 |
| **GO:0010033** | GO:0010033 | response to organic substance | 200 | 0.3286719774 | 1.757737243 | 0.0003649191096 | 0.0003649191096 | 0.009869888198 |
| **GO:0032103** | GO:0032103 | positive regulation of response to external stimulus | 58 | 0.4710445615 | 2.068155628 | 0.0004275331338 | 0.0004275331338 | 0.01140499348 |
| **GO:0002764** | GO:0002764 | immune response-regulating signaling pathway | 43 | 0.4853576471 | 1.998047112 | 0.0004405933324 | 0.0004405933324 | 0.01159456138 |
| **GO:0071216** | GO:0071216 | cellular response to biotic stimulus | 27 | 0.5581981812 | 2.049480506 | 0.0004633204633 | 0.0004633204633 | 0.01182904536 |
| **GO:0071219** | GO:0071219 | cellular response to molecule of bacterial origin | 25 | 0.5833099084 | 2.101989943 | 0.0004664904369 | 0.0004664904369 | 0.01182904536 |
| **GO:0031348** | GO:0031348 | negative regulation of defense response | 24 | 0.5829092026 | 2.073225384 | 0.0004677268475 | 0.0004677268475 | 0.01182904536 |
| **GO:0001676** | GO:0001676 | long-chain fatty acid metabolic process | 9 | -0.7963066894 | -2.312513891 | 0.0004909180167 | 0.0004909180167 | 0.0122434933 |
| **GO:0030595** | GO:0030595 | leukocyte chemotaxis | 13 | 0.7209662012 | 2.160430823 | 0.0004966887417 | 0.0004966887417 | 0.0122434933 |
| **GO:0097530** | GO:0097530 | granulocyte migration | 9 | 0.7931732158 | 2.111093838 | 0.000505987519 | 0.000505987519 | 0.01231680145 |
| **GO:0048583** | GO:0048583 | regulation of response to stimulus | 253 | 0.2921903891 | 1.602307191 | 0.0005892751915 | 0.0005892751915 | 0.01416710987 |
| **GO:0071222** | GO:0071222 | cellular response to lipopolysaccharide | 24 | 0.5768815187 | 2.051786801 | 0.0006236357967 | 0.0006236357967 | 0.01465659054 |
| **GO:0071310** | GO:0071310 | cellular response to organic substance | 161 | 0.3334793844 | 1.733458593 | 0.0006246876562 | 0.0006246876562 | 0.01465659054 |
| **GO:0034341** | GO:0034341 | response to interferon-gamma | 16 | 0.6561626287 | 2.086031244 | 0.0006512536633 | 0.0006512536633 | 0.0149294563 |
| **GO:0071674** | GO:0071674 | mononuclear cell migration | 14 | 0.6919505206 | 2.117951458 | 0.0006569223189 | 0.0006569223189 | 0.0149294563 |
| **GO:0035458** | GO:0035458 | cellular response to interferon-beta | 11 | 0.7409568261 | 2.097791693 | 0.0006723819129 | 0.0006723819129 | 0.0149294563 |
| **GO:0046916** | GO:0046916 | cellular transition metal ion homeostasis | 9 | 0.7828418994 | 2.083596215 | 0.0006746500253 | 0.0006746500253 | 0.0149294563 |
| **GO:0071347** | GO:0071347 | cellular response to interleukin-1 | 9 | 0.782285237 | 2.082114613 | 0.0006746500253 | 0.0006746500253 | 0.0149294563 |
| **GO:1901992** | GO:1901992 | positive regulation of mitotic cell cycle phase transition | 7 | -0.8013937282 | -2.128105564 | 0.0007072135785 | 0.0007072135785 | 0.01547421786 |
| **GO:0006629** | GO:0006629 | lipid metabolic process | 71 | -0.3550922935 | -1.887948684 | 0.0007222824124 | 0.0007222824124 | 0.0156283329 |
| **GO:0045931** | GO:0045931 | positive regulation of mitotic cell cycle | 9 | -0.7454069321 | -2.164698486 | 0.000736377025 | 0.000736377025 | 0.0157582128 |
| **GO:0019058** | GO:0019058 | viral life cycle | 33 | 0.5146023987 | 1.980241916 | 0.0007588404917 | 0.0007588404917 | 0.01606241315 |
| **GO:0009410** | GO:0009410 | response to xenobiotic stimulus | 18 | -0.6066236001 | -2.229257077 | 0.0007995735608 | 0.0007995735608 | 0.01670969941 |
| **GO:0062207** | GO:0062207 | regulation of pattern recognition receptor signaling pathway | 17 | 0.6323001188 | 2.052016359 | 0.0008065817067 | 0.0008065817067 | 0.01670969941 |
| **GO:0070555** | GO:0070555 | response to interleukin-1 | 10 | 0.7447000641 | 2.035873237 | 0.0008510638298 | 0.0008510638298 | 0.01744562975 |
| **GO:0030593** | GO:0030593 | neutrophil chemotaxis | 6 | 0.830679819 | 1.928972269 | 0.0008855827134 | 0.0008855827134 | 0.01796412302 |
| **GO:0055076** | GO:0055076 | transition metal ion homeostasis | 10 | 0.737680053 | 2.016681816 | 0.001021276596 | 0.001021276596 | 0.02050311125 |
| **GO:0019730** | GO:0019730 | antimicrobial humoral response | 7 | 0.792814251 | 1.937318824 | 0.001041485853 | 0.001041485853 | 0.02069547614 |
| **GO:1903900** | GO:1903900 | regulation of viral life cycle | 27 | 0.545769275 | 2.003846533 | 0.001081081081 | 0.001081081081 | 0.02126528442 |
| **GO:0000280** | GO:0000280 | nuclear division | 23 | -0.5098445427 | -2.026490499 | 0.001100412655 | 0.001100412655 | 0.02142908854 |
| **GO:0050777** | GO:0050777 | negative regulation of immune response | 18 | 0.62 | 2.039759438 | 0.001119820829 | 0.001119820829 | 0.02156633786 |
| **GO:0006954** | GO:0006954 | inflammatory response | 64 | 0.4188831961 | 1.874428601 | 0.001130103122 | 0.001130103122 | 0.02156633786 |
| **GO:0048699** | GO:0048699 | generation of neurons | 87 | -0.3240813429 | -1.793955057 | 0.001140684411 | 0.001140684411 | 0.02156633786 |
| **GO:0071621** | GO:0071621 | granulocyte chemotaxis | 8 | 0.7649234855 | 1.952522769 | 0.001206688502 | 0.001206688502 | 0.0225948758 |
| **GO:0060326** | GO:0060326 | cell chemotaxis | 19 | 0.6293552648 | 2.099546325 | 0.001275510204 | 0.001275510204 | 0.02365607897 |
| **GO:0070887** | GO:0070887 | cellular response to chemical stimulus | 199 | 0.3066160865 | 1.638384403 | 0.001338525189 | 0.001338525189 | 0.02459058192 |
| **GO:0050792** | GO:0050792 | regulation of viral process | 28 | 0.5332288195 | 1.979549915 | 0.001377410468 | 0.001377410468 | 0.02506846401 |
| **GO:1901989** | GO:1901989 | positive regulation of cell cycle phase transition | 8 | -0.7410803705 | -2.064830388 | 0.00142789148 | 0.00142789148 | 0.02574658127 |
| **GO:0002832** | GO:0002832 | negative regulation of response to biotic stimulus | 15 | 0.6471277843 | 2.019606167 | 0.001468428781 | 0.001468428781 | 0.02618838651 |
| **GO:0007049** | GO:0007049 | cell cycle | 79 | -0.3303608626 | -1.799438621 | 0.001479289941 | 0.001479289941 | 0.02618838651 |
| **GO:0016032** | GO:0016032 | viral process | 36 | 0.4935034383 | 1.945187341 | 0.001497678598 | 0.001497678598 | 0.02627506313 |
| **GO:0071675** | GO:0071675 | regulation of mononuclear cell migration | 10 | 0.7225734188 | 1.975383052 | 0.001531914894 | 0.001531914894 | 0.02654573292 |
| **GO:0002520** | GO:0002520 | immune system development | 66 | 0.4169336946 | 1.878190765 | 0.001541047913 | 0.001541047913 | 0.02654573292 |
| **GO:0042742** | GO:0042742 | defense response to bacterium | 26 | 0.5396894022 | 1.959886309 | 0.001554001554 | 0.001554001554 | 0.02654573292 |
| **GO:0002697** | GO:0002697 | regulation of immune effector process | 35 | 0.4857577216 | 1.8988315 | 0.001660126773 | 0.001660126773 | 0.02811198655 |
| **GO:0007051** | GO:0007051 | spindle organization | 10 | -0.6784351885 | -2.071616851 | 0.001695325745 | 0.001695325745 | 0.02831444492 |
| **GO:0048285** | GO:0048285 | organelle fission | 27 | -0.4684016092 | -1.94578595 | 0.001701162461 | 0.001701162461 | 0.02831444492 |
| **GO:0048525** | GO:0048525 | negative regulation of viral process | 21 | 0.5799097541 | 1.989122535 | 0.001736933523 | 0.001736933523 | 0.0286648262 |
| **GO:0030182** | GO:0030182 | neuron differentiation | 75 | -0.3270647296 | -1.762163732 | 0.001826150475 | 0.001826150475 | 0.02988393081 |
| **GO:0001818** | GO:0001818 | negative regulation of cytokine production | 25 | 0.5397976382 | 1.945191039 | 0.001865961748 | 0.001865961748 | 0.03028095819 |
| **GO:0072676** | GO:0072676 | lymphocyte migration | 9 | 0.7440782139 | 1.980423571 | 0.002023950076 | 0.002023950076 | 0.0325733592 |
| **GO:0002690** | GO:0002690 | positive regulation of leukocyte chemotaxis | 9 | 0.725647783 | 1.931369508 | 0.002192612582 | 0.002192612582 | 0.03499856149 |
| **GO:0032479** | GO:0032479 | regulation of type I interferon production | 18 | 0.6036980297 | 1.986127022 | 0.002239641657 | 0.002239641657 | 0.03517264063 |
| **GO:0032606** | GO:0032606 | type I interferon production | 18 | 0.6036980297 | 1.986127022 | 0.002239641657 | 0.002239641657 | 0.03517264063 |
| **GO:0045071** | GO:0045071 | negative regulation of viral genome replication | 15 | 0.629913214 | 1.965881612 | 0.002284222549 | 0.002284222549 | 0.03558578286 |
| **GO:0033993** | GO:0033993 | response to lipid | 47 | 0.4432108736 | 1.865936698 | 0.002316825948 | 0.002316825948 | 0.0358072515 |
| **GO:0045824** | GO:0045824 | negative regulation of innate immune response | 14 | 0.6463700234 | 1.978436742 | 0.002791919855 | 0.002791919855 | 0.04281020914 |
| **GO:0002224** | GO:0002224 | toll-like receptor signaling pathway | 18 | 0.5988599826 | 1.970210163 | 0.002879539274 | 0.002879539274 | 0.04380878007 |
| **GO:0071706** | GO:0071706 | tumor necrosis factor superfamily cytokine production | 22 | 0.5549855259 | 1.924127568 | 0.002984605718 | 0.002984605718 | 0.04459622338 |
| **GO:1903555** | GO:1903555 | regulation of tumor necrosis factor superfamily cytokine production | 22 | 0.5549855259 | 1.924127568 | 0.002984605718 | 0.002984605718 | 0.04459622338 |
| **GO:0022402** | GO:0022402 | cell cycle process | 55 | -0.3700319782 | -1.856958409 | 0.003 | 0.003 | 0.04459622338 |
| **GO:0006875** | GO:0006875 | cellular metal ion homeostasis | 38 | 0.4612109353 | 1.84511429 | 0.00312221231 | 0.00312221231 | 0.04606134589 |
| **GO:0006633** | GO:0006633 | fatty acid biosynthetic process | 5 | -0.8079402686 | -1.894373193 | 0.003215434084 | 0.003215434084 | 0.04707996086 |
| **GO:0002218** | GO:0002218 | activation of innate immune response | 13 | 0.6552246381 | 1.963431159 | 0.003311258278 | 0.003311258278 | 0.04812119257 |
| **GO:1902850** | GO:1902850 | microtubule cytoskeleton organization involved in mitosis | 7 | -0.7386759582 | -1.961558172 | 0.003536067893 | 0.003536067893 | 0.05071624126 |
| **GO:0035710** | GO:0035710 | CD4-positive, alpha-beta T cell activation | 9 | 0.7067532405 | 1.881080175 | 0.003541912633 | 0.003541912633 | 0.05071624126 |
| **GO:0045069** | GO:0045069 | regulation of viral genome replication | 16 | 0.6008491591 | 1.910182116 | 0.003581895148 | 0.003581895148 | 0.05091437591 |
| **GO:0019748** | GO:0019748 | secondary metabolic process | 7 | -0.7360850134 | -1.954677904 | 0.003771805752 | 0.003771805752 | 0.05322532907 |
| **GO:0071396** | GO:0071396 | cellular response to lipid | 33 | 0.4781802248 | 1.840085718 | 0.003945970557 | 0.003945970557 | 0.05528243491 |
| **GO:0048519** | GO:0048519 | negative regulation of biological process | 286 | 0.2689677078 | 1.493181587 | 0.004176818656 | 0.004176818656 | 0.05809860537 |
| **GO:0034121** | GO:0034121 | regulation of toll-like receptor signaling pathway | 12 | 0.6539429493 | 1.904953073 | 0.004494007989 | 0.004494007989 | 0.06206729959 |
| **GO:0048534** | GO:0048534 | hematopoietic or lymphoid organ development | 60 | 0.3954032433 | 1.748357279 | 0.004531294251 | 0.004531294251 | 0.06214154458 |
| **GO:0045787** | GO:0045787 | positive regulation of cell cycle | 21 | -0.4874076138 | -1.883066031 | 0.004633415099 | 0.004633415099 | 0.06309766606 |
| **GO:0006873** | GO:0006873 | cellular ion homeostasis | 41 | 0.435387312 | 1.777262195 | 0.004865103936 | 0.004865103936 | 0.06579270674 |
| **GO:0002687** | GO:0002687 | positive regulation of leukocyte migration | 14 | 0.6176274563 | 1.890460275 | 0.005091147972 | 0.005091147972 | 0.06837476405 |
| **GO:0019079** | GO:0019079 | viral genome replication | 19 | 0.5615583526 | 1.873373977 | 0.005261479592 | 0.005261479592 | 0.07017835072 |
| **GO:1901701** | GO:1901701 | cellular response to oxygen-containing compound | 67 | 0.3819034059 | 1.725492657 | 0.005310229178 | 0.005310229178 | 0.07034675245 |
| **GO:0060759** | GO:0060759 | regulation of response to cytokine stimulus | 19 | 0.5601252638 | 1.868593154 | 0.005420918367 | 0.005420918367 | 0.07096047885 |
| **GO:0000226** | GO:0000226 | microtubule cytoskeleton organization | 29 | -0.4378582084 | -1.856463389 | 0.005500868558 | 0.005500868558 | 0.07096047885 |
| **GO:0032608** | GO:0032608 | interferon-beta production | 15 | 0.6016103603 | 1.877551889 | 0.005547397618 | 0.005547397618 | 0.07096047885 |
| **GO:0032648** | GO:0032648 | regulation of interferon-beta production | 15 | 0.6016103603 | 1.877551889 | 0.005547397618 | 0.005547397618 | 0.07096047885 |
| **GO:0002250** | GO:0002250 | adaptive immune response | 34 | 0.4602954726 | 1.78518626 | 0.005604362314 | 0.005604362314 | 0.07096047885 |
| **GO:0030003** | GO:0030003 | cellular cation homeostasis | 40 | 0.4387962963 | 1.78004376 | 0.005618808221 | 0.005618808221 | 0.07096047885 |
| **GO:0044281** | GO:0044281 | small molecule metabolic process | 115 | -0.2739282503 | -1.606208751 | 0.005664488017 | 0.005664488017 | 0.07096047885 |
| **GO:0032640** | GO:0032640 | tumor necrosis factor production | 21 | 0.5433412733 | 1.863690623 | 0.005684509711 | 0.005684509711 | 0.07096047885 |
| **GO:0032680** | GO:0032680 | regulation of tumor necrosis factor production | 21 | 0.5433412733 | 1.863690623 | 0.005684509711 | 0.005684509711 | 0.07096047885 |
| **GO:0050778** | GO:0050778 | positive regulation of immune response | 49 | 0.4150854865 | 1.763425036 | 0.005782853838 | 0.005782853838 | 0.07172832451 |
| **GO:0000281** | GO:0000281 | mitotic cytokinesis | 9 | -0.6538233358 | -1.898735207 | 0.0058910162 | 0.0058910162 | 0.0726074615 |
| **GO:1901700** | GO:1901700 | response to oxygen-containing compound | 93 | 0.3434626862 | 1.649132696 | 0.006042701759 | 0.006042701759 | 0.07400859486 |
| **GO:0001906** | GO:0001906 | cell killing | 10 | 0.6735558181 | 1.841377932 | 0.006127659574 | 0.006127659574 | 0.07414591917 |
| **GO:0090068** | GO:0090068 | positive regulation of cell cycle process | 18 | -0.5092312656 | -1.871353839 | 0.006130063966 | 0.006130063966 | 0.07414591917 |
| **GO:0002699** | GO:0002699 | positive regulation of immune effector process | 23 | 0.5210155943 | 1.83125592 | 0.006282393592 | 0.006282393592 | 0.07551935117 |
| **GO:0002688** | GO:0002688 | regulation of leukocyte chemotaxis | 11 | 0.6534924024 | 1.850163039 | 0.006387628173 | 0.006387628173 | 0.07583538484 |
| **GO:0000086** | GO:0000086 | G2/M transition of mitotic cell cycle | 8 | -0.6709365014 | -1.869392487 | 0.006425511661 | 0.006425511661 | 0.07583538484 |
| **GO:0044839** | GO:0044839 | cell cycle G2/M phase transition | 8 | -0.6709365014 | -1.869392487 | 0.006425511661 | 0.006425511661 | 0.07583538484 |
| **GO:0032481** | GO:0032481 | positive regulation of type I interferon production | 16 | 0.5792415748 | 1.841488634 | 0.006512536633 | 0.006512536633 | 0.07639944687 |
| **GO:0007052** | GO:0007052 | mitotic spindle organization | 6 | -0.7431500978 | -1.870450041 | 0.006654428637 | 0.006654428637 | 0.07759655202 |
| **GO:0051276** | GO:0051276 | chromosome organization | 34 | -0.3995922673 | -1.779275124 | 0.007058823529 | 0.007058823529 | 0.08162613978 |
| **GO:0071356** | GO:0071356 | cellular response to tumor necrosis factor | 9 | 0.6827668859 | 1.817238577 | 0.007083825266 | 0.007083825266 | 0.08162613978 |
| **GO:0002252** | GO:0002252 | immune effector process | 53 | 0.398638868 | 1.725209989 | 0.007140816909 | 0.007140816909 | 0.08179883147 |
| **GO:0006935** | GO:0006935 | chemotaxis | 31 | 0.4656348903 | 1.76935606 | 0.007313728478 | 0.007313728478 | 0.08280537138 |
| **GO:0042330** | GO:0042330 | taxis | 31 | 0.4656348903 | 1.76935606 | 0.007313728478 | 0.007313728478 | 0.08280537138 |
| **GO:0072330** | GO:0072330 | monocarboxylic acid biosynthetic process | 11 | -0.6048748873 | -1.906683456 | 0.007648655317 | 0.007648655317 | 0.08609681981 |
| **GO:0034612** | GO:0034612 | response to tumor necrosis factor | 10 | 0.6616459074 | 1.808818423 | 0.007829787234 | 0.007829787234 | 0.08762919772 |
| **GO:0032728** | GO:0032728 | positive regulation of interferon-beta production | 14 | 0.5945459319 | 1.819811368 | 0.008211528987 | 0.008211528987 | 0.09052457462 |
| **GO:0061640** | GO:0061640 | cytoskeleton-dependent cytokinesis | 10 | -0.6127899361 | -1.871167621 | 0.008234439332 | 0.008234439332 | 0.09052457462 |
| **GO:0043367** | GO:0043367 | CD4-positive, alpha-beta T cell differentiation | 8 | 0.7007510336 | 1.788717924 | 0.008274435442 | 0.008274435442 | 0.09052457462 |
| **GO:0046632** | GO:0046632 | alpha-beta T cell differentiation | 8 | 0.7007510336 | 1.788717924 | 0.008274435442 | 0.008274435442 | 0.09052457462 |
| **GO:0030522** | GO:0030522 | intracellular receptor signaling pathway | 17 | 0.5564153146 | 1.805745869 | 0.008549766091 | 0.008549766091 | 0.09301421505 |
| **GO:0032733** | GO:0032733 | positive regulation of interleukin-10 production | 5 | 0.7821291017 | 1.715776144 | 0.008674101611 | 0.008674101611 | 0.09384261977 |
| **GO:0071346** | GO:0071346 | cellular response to interferon-gamma | 13 | 0.6070578617 | 1.819095699 | 0.008774834437 | 0.008774834437 | 0.09440793084 |
| **GO:0002753** | GO:0002753 | cytoplasmic pattern recognition receptor signaling pathway | 13 | 0.6057266702 | 1.815106681 | 0.008940397351 | 0.008940397351 | 0.09533131012 |
| **GO:0043547** | GO:0043547 | positive regulation of GTPase activity | 18 | 0.5505535346 | 1.81128511 | 0.008958566629 | 0.008958566629 | 0.09533131012 |
| **GO:0007165** | GO:0007165 | signal transduction | 278 | 0.2623868382 | 1.453572076 | 0.00917964211 | 0.00917964211 | 0.09715296284 |
| **GO:0006638** | GO:0006638 | neutral lipid metabolic process | 8 | -0.6526159565 | -1.818346987 | 0.009519276535 | 0.009519276535 | 0.09913122201 |
| **GO:0006639** | GO:0006639 | acylglycerol metabolic process | 8 | -0.6526159565 | -1.818346987 | 0.009519276535 | 0.009519276535 | 0.09913122201 |
| **GO:0006641** | GO:0006641 | triglyceride metabolic process | 8 | -0.6526159565 | -1.818346987 | 0.009519276535 | 0.009519276535 | 0.09913122201 |
| **GO:0022008** | GO:0022008 | neurogenesis | 99 | -0.272384603 | -1.550929396 | 0.009657947686 | 0.009657947686 | 0.1000403316 |
| **GO:0003341** | GO:0003341 | cilium movement | 5 | 0.7788591292 | 1.708602724 | 0.009913258984 | 0.009913258984 | 0.102141627 |
| **GO:0030097** | GO:0030097 | hemopoiesis | 59 | 0.3804262426 | 1.676385802 | 0.01019252548 | 0.01019252548 | 0.1041025765 |
| **GO:0045089** | GO:0045089 | positive regulation of innate immune response | 22 | 0.5127498157 | 1.777696912 | 0.01021049325 | 0.01021049325 | 0.1041025765 |
| **GO:0055065** | GO:0055065 | metal ion homeostasis | 43 | 0.4115330633 | 1.694137207 | 0.01057423998 | 0.01057423998 | 0.106853166 |
| **GO:0006959** | GO:0006959 | humoral immune response | 11 | 0.6253384769 | 1.770453846 | 0.01059001513 | 0.01059001513 | 0.106853166 |
| **GO:0000910** | GO:0000910 | cytokinesis | 14 | -0.5275975976 | -1.795422649 | 0.01098901099 | 0.01098901099 | 0.1103074896 |
| **GO:0032635** | GO:0032635 | interleukin-6 production | 19 | 0.5293310642 | 1.765862864 | 0.01132015306 | 0.01132015306 | 0.1120523572 |
| **GO:0032675** | GO:0032675 | regulation of interleukin-6 production | 19 | 0.5293310642 | 1.765862864 | 0.01132015306 | 0.01132015306 | 0.1120523572 |
| **GO:0060700** | GO:0060700 | regulation of ribonuclease activity | 6 | 0.7378190255 | 1.713334557 | 0.01133545873 | 0.01133545873 | 0.1120523572 |
| **GO:0034340** | GO:0034340 | response to type I interferon | 12 | 0.6062283032 | 1.765959049 | 0.0119840213 | 0.0119840213 | 0.1166865232 |
| **GO:0060337** | GO:0060337 | type I interferon signaling pathway | 12 | 0.6062283032 | 1.765959049 | 0.0119840213 | 0.0119840213 | 0.1166865232 |
| **GO:0071357** | GO:0071357 | cellular response to type I interferon | 12 | 0.6062283032 | 1.765959049 | 0.0119840213 | 0.0119840213 | 0.1166865232 |
| **GO:0002685** | GO:0002685 | regulation of leukocyte migration | 17 | 0.5439636019 | 1.765336075 | 0.01242135828 | 0.01242135828 | 0.1203244279 |
| **GO:0007154** | GO:0007154 | cell communication | 293 | 0.2562660924 | 1.426005402 | 0.01248122039 | 0.01248122039 | 0.1203244279 |
| **GO:0051248** | GO:0051248 | negative regulation of protein metabolic process | 66 | 0.3595676996 | 1.619770102 | 0.01274866909 | 0.01274866909 | 0.1222973183 |
| **GO:0031664** | GO:0031664 | regulation of lipopolysaccharide-mediated signaling pathway | 7 | 0.7119628339 | 1.739750513 | 0.01319215414 | 0.01319215414 | 0.125404068 |
| **GO:0032465** | GO:0032465 | regulation of cytokinesis | 7 | -0.6716858038 | -1.78366544 | 0.01320132013 | 0.01320132013 | 0.125404068 |
| **GO:0051336** | GO:0051336 | regulation of hydrolase activity | 71 | 0.3505391365 | 1.600344872 | 0.01327250104 | 0.01327250104 | 0.1254682009 |
| **GO:0023052** | GO:0023052 | signaling | 294 | 0.254536977 | 1.416779088 | 0.01338718984 | 0.01338718984 | 0.1259410181 |
| **GO:0035455** | GO:0035455 | response to interferon-alpha | 11 | 0.6130952384 | 1.735790876 | 0.01445621113 | 0.01445621113 | 0.1353239966 |
| **GO:0032653** | GO:0032653 | regulation of interleukin-10 production | 6 | 0.7262122843 | 1.686381835 | 0.0145235565 | 0.0145235565 | 0.1353239966 |
| **GO:0050921** | GO:0050921 | positive regulation of chemotaxis | 13 | 0.5819917779 | 1.743983246 | 0.01473509934 | 0.01473509934 | 0.1366412721 |
| **GO:0060338** | GO:0060338 | regulation of type I interferon-mediated signaling pathway | 8 | 0.6738728224 | 1.720109337 | 0.01499741424 | 0.01499741424 | 0.1384146488 |
| **GO:0002040** | GO:0002040 | sprouting angiogenesis | 6 | -0.7041763341 | -1.772356159 | 0.01514456173 | 0.01514456173 | 0.1386091586 |
| **GO:0065009** | GO:0065009 | regulation of molecular function | 166 | 0.2823995973 | 1.474349274 | 0.01516092954 | 0.01516092954 | 0.1386091586 |
| **GO:0034122** | GO:0034122 | negative regulation of toll-like receptor signaling pathway | 6 | 0.7231197007 | 1.679200358 | 0.01523202267 | 0.01523202267 | 0.1386091586 |
| **GO:0030258** | GO:0030258 | lipid modification | 6 | -0.7010173956 | -1.764405361 | 0.01583295089 | 0.01583295089 | 0.1434073888 |
| **GO:0007166** | GO:0007166 | cell surface receptor signaling pathway | 173 | 0.2786995772 | 1.464006077 | 0.01616685178 | 0.01616685178 | 0.1457537807 |
| **GO:0050732** | GO:0050732 | negative regulation of peptidyl-tyrosine phosphorylation | 6 | 0.7199160394 | 1.671760941 | 0.01629472193 | 0.01629472193 | 0.1462296171 |
| **GO:0055082** | GO:0055082 | cellular chemical homeostasis | 49 | 0.3851994618 | 1.636458987 | 0.01662570479 | 0.01662570479 | 0.1481758291 |
| **GO:0032069** | GO:0032069 | regulation of nuclease activity | 7 | 0.6998878339 | 1.710244075 | 0.01666377365 | 0.01666377365 | 0.1481758291 |
| **GO:0001959** | GO:0001959 | regulation of cytokine-mediated signaling pathway | 16 | 0.5452828487 | 1.733529173 | 0.01693259525 | 0.01693259525 | 0.1498818239 |
| **GO:0051092** | GO:0051092 | positive regulation of NF-kappaB transcription factor activity | 18 | 0.5193623133 | 1.708668032 | 0.01775715885 | 0.01775715885 | 0.1564693683 |
| **GO:0051225** | GO:0051225 | spindle assembly | 5 | -0.737562407 | -1.729358601 | 0.01837390905 | 0.01837390905 | 0.1608554298 |
| **GO:0002507** | GO:0002507 | tolerance induction | 6 | 0.7121578756 | 1.653745235 | 0.01842012044 | 0.01842012044 | 0.1608554298 |
| **GO:0007017** | GO:0007017 | microtubule-based process | 37 | -0.3588789952 | -1.62816687 | 0.01907934585 | 0.01907934585 | 0.1658683732 |
| **GO:2001237** | GO:2001237 | negative regulation of extrinsic apoptotic signaling pathway | 6 | 0.709241527 | 1.646973005 | 0.01965993624 | 0.01965993624 | 0.1701561733 |
| **GO:0051726** | GO:0051726 | regulation of cell cycle | 58 | -0.3034903969 | -1.54575105 | 0.01976549414 | 0.01976549414 | 0.1703128279 |
| **GO:0051302** | GO:0051302 | regulation of cell division | 12 | -0.5377461252 | -1.745402937 | 0.02003004507 | 0.02003004507 | 0.1718320583 |
| **GO:0032655** | GO:0032655 | regulation of interleukin-12 production | 10 | 0.6161655336 | 1.684483432 | 0.02025531915 | 0.02025531915 | 0.1727032965 |
| **GO:0032613** | GO:0032613 | interleukin-10 production | 7 | 0.6835557838 | 1.67033512 | 0.02030897414 | 0.02030897414 | 0.1727032965 |
| **GO:0007062** | GO:0007062 | sister chromatid cohesion | 5 | -0.7298745308 | -1.711332879 | 0.02044097382 | 0.02044097382 | 0.17307003 |
| **GO:0048585** | GO:0048585 | negative regulation of response to stimulus | 103 | 0.3092381323 | 1.507977534 | 0.02165588274 | 0.02165588274 | 0.182461883 |
| **GO:0032269** | GO:0032269 | negative regulation of cellular protein metabolic process | 61 | 0.3513839011 | 1.559709494 | 0.02175755863 | 0.02175755863 | 0.182461883 |
| **GO:0043087** | GO:0043087 | regulation of GTPase activity | 23 | 0.4755317092 | 1.671390007 | 0.02183131773 | 0.02183131773 | 0.182461883 |
| **GO:0008625** | GO:0008625 | extrinsic apoptotic signaling pathway via death domain receptors | 10 | 0.6142191142 | 1.679162278 | 0.02195744681 | 0.02195744681 | 0.1827317886 |
| **GO:0055080** | GO:0055080 | cation homeostasis | 47 | 0.3798303025 | 1.599101788 | 0.02244425138 | 0.02244425138 | 0.1859881973 |
| **GO:0006879** | GO:0006879 | cellular iron ion homeostasis | 7 | 0.6775570693 | 1.655676677 | 0.02256552682 | 0.02256552682 | 0.1862008234 |
| **GO:0032602** | GO:0032602 | chemokine production | 12 | 0.5784093123 | 1.684921594 | 0.02313581891 | 0.02313581891 | 0.1893023661 |
| **GO:0032642** | GO:0032642 | regulation of chemokine production | 12 | 0.5784093123 | 1.684921594 | 0.02313581891 | 0.02313581891 | 0.1893023661 |
| **GO:1903556** | GO:1903556 | negative regulation of tumor necrosis factor superfamily cytokine production | 7 | 0.6753612275 | 1.650310923 | 0.0234334317 | 0.0234334317 | 0.1909352506 |
| **GO:0010564** | GO:0010564 | regulation of cell cycle process | 39 | -0.3448858021 | -1.587764092 | 0.02361238884 | 0.02361238884 | 0.1914189143 |
| **GO:0032715** | GO:0032715 | negative regulation of interleukin-6 production | 7 | 0.6739623696 | 1.646892678 | 0.02378059365 | 0.02378059365 | 0.1914189143 |
| **GO:0008286** | GO:0008286 | insulin receptor signaling pathway | 8 | 0.6534585225 | 1.668000353 | 0.02396138597 | 0.02396138597 | 0.1914189143 |
| **GO:0050801** | GO:0050801 | ion homeostasis | 49 | 0.3742584625 | 1.589977882 | 0.02399884343 | 0.02399884343 | 0.1914189143 |
| **GO:0098771** | GO:0098771 | inorganic ion homeostasis | 49 | 0.3742584625 | 1.589977882 | 0.02399884343 | 0.02399884343 | 0.1914189143 |
| **GO:0019725** | GO:0019725 | cellular homeostasis | 51 | 0.36816651 | 1.581180446 | 0.02408256881 | 0.02408256881 | 0.1914189143 |
| **GO:0032615** | GO:0032615 | interleukin-12 production | 11 | 0.5903267272 | 1.671328829 | 0.02437384434 | 0.02437384434 | 0.1929465641 |
| **GO:0050808** | GO:0050808 | synapse organization | 19 | -0.4454132899 | -1.664166799 | 0.02466487936 | 0.02466487936 | 0.1939273302 |
| **GO:0046686** | GO:0046686 | response to cadmium ion | 5 | 0.7391265802 | 1.621440439 | 0.02496016994 | 0.02496016994 | 0.1939273302 |
| **GO:2000514** | GO:2000514 | regulation of CD4-positive, alpha-beta T cell activation | 5 | 0.7392815759 | 1.621780457 | 0.02496016994 | 0.02496016994 | 0.1939273302 |
| **GO:0044782** | GO:0044782 | cilium organization | 7 | 0.6712560218 | 1.640279454 | 0.02499566048 | 0.02499566048 | 0.1939273302 |
| **GO:0060271** | GO:0060271 | cilium assembly | 7 | 0.6712560218 | 1.640279454 | 0.02499566048 | 0.02499566048 | 0.1939273302 |
| **GO:0010647** | GO:0010647 | positive regulation of cell communication | 128 | 0.2896252276 | 1.464371458 | 0.02530998338 | 0.02530998338 | 0.1947804998 |
| **GO:0023056** | GO:0023056 | positive regulation of signaling | 128 | 0.2896252276 | 1.464371458 | 0.02530998338 | 0.02530998338 | 0.1947804998 |
| **GO:0007600** | GO:0007600 | sensory perception | 21 | 0.4851911716 | 1.664232558 | 0.02542239065 | 0.02542239065 | 0.1947804998 |
| **GO:0007399** | GO:0007399 | nervous system development | 119 | -0.238126663 | -1.403871932 | 0.0255057168 | 0.0255057168 | 0.1947804998 |
| **GO:0001960** | GO:0001960 | negative regulation of cytokine-mediated signaling pathway | 7 | 0.66842851 | 1.633370153 | 0.02725221316 | 0.02725221316 | 0.2064984409 |
| **GO:0060761** | GO:0060761 | negative regulation of response to cytokine stimulus | 7 | 0.66842851 | 1.633370153 | 0.02725221316 | 0.02725221316 | 0.2064984409 |
| **GO:0010629** | GO:0010629 | negative regulation of gene expression | 63 | 0.3433687222 | 1.53116923 | 0.0276757978 | 0.0276757978 | 0.2085395031 |
| **GO:0031663** | GO:0031663 | lipopolysaccharide-mediated signaling pathway | 11 | 0.5835105128 | 1.652030812 | 0.02773575391 | 0.02773575391 | 0.2085395031 |
| **GO:0009967** | GO:0009967 | positive regulation of signal transduction | 122 | 0.2894932709 | 1.453599292 | 0.02867846531 | 0.02867846531 | 0.214798222 |
| **GO:0043067** | GO:0043067 | regulation of programmed cell death | 103 | 0.3002264445 | 1.464032686 | 0.02891852634 | 0.02891852634 | 0.2157663793 |
| **GO:0051091** | GO:0051091 | positive regulation of DNA-binding transcription factor activity | 24 | 0.458255426 | 1.62987096 | 0.02962270034 | 0.02962270034 | 0.2201767603 |
| **GO:0016053** | GO:0016053 | organic acid biosynthetic process | 14 | -0.4848897811 | -1.6500873 | 0.02990033223 | 0.02990033223 | 0.2203354312 |
| **GO:0046394** | GO:0046394 | carboxylic acid biosynthetic process | 14 | -0.4848897811 | -1.6500873 | 0.02990033223 | 0.02990033223 | 0.2203354312 |
| **GO:0010605** | GO:0010605 | negative regulation of macromolecule metabolic process | 139 | 0.2806969452 | 1.434069626 | 0.02998348367 | 0.02998348367 | 0.2203354312 |
| **GO:1903131** | GO:1903131 | mononuclear cell differentiation | 28 | 0.4371975527 | 1.623045017 | 0.0301499847 | 0.0301499847 | 0.2207260455 |
| **GO:0034114** | GO:0034114 | regulation of heterotypic cell-cell adhesion | 5 | 0.7253765933 | 1.591276749 | 0.03133297929 | 0.03133297929 | 0.2285275446 |
| **GO:0060339** | GO:0060339 | negative regulation of type I interferon-mediated signaling pathway | 5 | 0.7219003476 | 1.583650822 | 0.03274915914 | 0.03274915914 | 0.2372601366 |
| **GO:0051716** | GO:0051716 | cellular response to stimulus | 357 | 0.2363884061 | 1.334290292 | 0.03277396103 | 0.03277396103 | 0.2372601366 |
| **GO:0032609** | GO:0032609 | interferon-gamma production | 11 | 0.5727448676 | 1.621551193 | 0.03328290469 | 0.03328290469 | 0.2393029536 |
| **GO:0044403** | GO:0044403 | biological process involved in symbiotic interaction | 22 | 0.4646077803 | 1.610789105 | 0.03330191643 | 0.03330191643 | 0.2393029536 |
| **GO:0022898** | GO:0022898 | regulation of transmembrane transporter activity | 8 | 0.6312604676 | 1.611338205 | 0.03499396656 | 0.03499396656 | 0.2496031671 |
| **GO:0032412** | GO:0032412 | regulation of ion transmembrane transporter activity | 8 | 0.6312604676 | 1.611338205 | 0.03499396656 | 0.03499396656 | 0.2496031671 |
| **GO:0046631** | GO:0046631 | alpha-beta T cell activation | 12 | 0.554029536 | 1.613902662 | 0.03511984021 | 0.03511984021 | 0.2496031671 |
| **GO:0002698** | GO:0002698 | negative regulation of immune effector process | 11 | 0.570622999 | 1.615543774 | 0.03530005043 | 0.03530005043 | 0.249971649 |
| **GO:0060627** | GO:0060627 | regulation of vesicle-mediated transport | 34 | 0.4023736223 | 1.560545138 | 0.03574674341 | 0.03574674341 | 0.2512543189 |
| **GO:0032760** | GO:0032760 | positive regulation of tumor necrosis factor production | 14 | 0.5281030445 | 1.616440164 | 0.03596649696 | 0.03596649696 | 0.2512543189 |
| **GO:1903557** | GO:1903557 | positive regulation of tumor necrosis factor superfamily cytokine production | 14 | 0.5281030445 | 1.616440164 | 0.03596649696 | 0.03596649696 | 0.2512543189 |
| **GO:0010941** | GO:0010941 | regulation of cell death | 113 | 0.2897932908 | 1.435987357 | 0.03608180279 | 0.03608180279 | 0.2512543189 |
| **GO:0002367** | GO:0002367 | cytokine production involved in immune response | 10 | 0.5879035783 | 1.607220435 | 0.03625531915 | 0.03625531915 | 0.2512543189 |
| **GO:0002718** | GO:0002718 | regulation of cytokine production involved in immune response | 10 | 0.5879035783 | 1.607220435 | 0.03625531915 | 0.03625531915 | 0.2512543189 |
| **GO:0034249** | GO:0034249 | negative regulation of cellular amide metabolic process | 11 | 0.5668912511 | 1.604978476 | 0.0378214826 | 0.0378214826 | 0.2607697479 |
| **GO:0007156** | GO:0007156 | homophilic cell adhesion via plasma membrane adhesion molecules | 5 | -0.6906141367 | -1.619279244 | 0.03789618741 | 0.03789618741 | 0.2607697479 |
| **GO:0062208** | GO:0062208 | positive regulation of pattern recognition receptor signaling pathway | 11 | 0.5664816962 | 1.603818947 | 0.03815767356 | 0.03815767356 | 0.261644537 |
| **GO:0002460** | GO:0002460 | adaptive immune response based on somatic recombination of immune receptors built from immunoglobulin superfamily domains | 26 | 0.4370641399 | 1.587201862 | 0.03869463869 | 0.03869463869 | 0.2643954999 |
| **GO:0043903** | GO:0043903 | regulation of biological process involved in symbiotic interaction | 13 | 0.5373985138 | 1.610356091 | 0.0392384106 | 0.0392384106 | 0.2671735723 |
| **GO:0031638** | GO:0031638 | zymogen activation | 7 | 0.6464544454 | 1.579674387 | 0.04079152925 | 0.04079152925 | 0.2765490944 |
| **GO:0051241** | GO:0051241 | negative regulation of multicellular organismal process | 84 | 0.3079927468 | 1.451007286 | 0.04099700354 | 0.04099700354 | 0.2765490944 |
| **GO:0010942** | GO:0010942 | positive regulation of cell death | 59 | 0.3349116398 | 1.475821211 | 0.04105322763 | 0.04105322763 | 0.2765490944 |
| **GO:1904035** | GO:1904035 | regulation of epithelial cell apoptotic process | 11 | 0.56151056 | 1.589744702 | 0.04118339217 | 0.04118339217 | 0.2765490944 |
| **GO:0072503** | GO:0072503 | cellular divalent inorganic cation homeostasis | 31 | 0.408862131 | 1.553626466 | 0.04144446137 | 0.04144446137 | 0.2773458258 |
| **GO:0031349** | GO:0031349 | positive regulation of defense response | 37 | 0.3851794872 | 1.530336453 | 0.04208955224 | 0.04208955224 | 0.2797404188 |
| **GO:0050877** | GO:0050877 | nervous system process | 39 | 0.3791557868 | 1.5249955 | 0.04213024774 | 0.04213024774 | 0.2797404188 |
| **GO:0002440** | GO:0002440 | production of molecular mediator of immune response | 18 | 0.482726938 | 1.588140045 | 0.04223324268 | 0.04223324268 | 0.2797404188 |
| **GO:0032409** | GO:0032409 | regulation of transporter activity | 10 | 0.5788709561 | 1.582526905 | 0.04272340426 | 0.04272340426 | 0.2820278247 |
| **GO:2000377** | GO:2000377 | regulation of reactive oxygen species metabolic process | 13 | 0.5297622603 | 1.587473468 | 0.04321192053 | 0.04321192053 | 0.2842889509 |
| **GO:0050920** | GO:0050920 | regulation of chemotaxis | 18 | 0.4811562752 | 1.582972667 | 0.04351303791 | 0.04351303791 | 0.2849266121 |
| **GO:0042981** | GO:0042981 | regulation of apoptotic process | 100 | 0.2936905115 | 1.427271086 | 0.04361122191 | 0.04361122191 | 0.2849266121 |
| **GO:0002720** | GO:0002720 | positive regulation of cytokine production involved in immune response | 6 | 0.6683302614 | 1.55197046 | 0.04374778604 | 0.04374778604 | 0.2849266121 |
| **GO:0050728** | GO:0050728 | negative regulation of inflammatory response | 12 | 0.5415929907 | 1.577674678 | 0.04444074567 | 0.04444074567 | 0.2877270818 |
| **GO:0051240** | GO:0051240 | positive regulation of multicellular organismal process | 121 | 0.2801788145 | 1.404289185 | 0.04454227632 | 0.04454227632 | 0.2877270818 |
| **GO:0070371** | GO:0070371 | ERK1 and ERK2 cascade | 30 | 0.4085683029 | 1.539744791 | 0.04462102689 | 0.04462102689 | 0.2877270818 |
| **GO:0032651** | GO:0032651 | regulation of interleukin-1 beta production | 12 | 0.5387171571 | 1.569297299 | 0.04610519308 | 0.04610519308 | 0.2939263126 |
| **GO:0032652** | GO:0032652 | regulation of interleukin-1 production | 12 | 0.5387171571 | 1.569297299 | 0.04610519308 | 0.04610519308 | 0.2939263126 |
| **GO:0050866** | GO:0050866 | negative regulation of cell activation | 21 | 0.4543368842 | 1.558400645 | 0.04626559293 | 0.04626559293 | 0.2939263126 |
| **GO:0002443** | GO:0002443 | leukocyte mediated immunity | 30 | 0.4072681474 | 1.534844979 | 0.04630195599 | 0.04630195599 | 0.2939263126 |
| **GO:0002700** | GO:0002700 | regulation of production of molecular mediator of immune response | 15 | 0.5022610589 | 1.567494947 | 0.04633708598 | 0.04633708598 | 0.2939263126 |
| **GO:0051345** | GO:0051345 | positive regulation of hydrolase activity | 40 | 0.3717504593 | 1.508062148 | 0.04687268963 | 0.04687268963 | 0.2954987732 |
| **GO:0055072** | GO:0055072 | iron ion homeostasis | 8 | 0.6138950566 | 1.567011732 | 0.04688846751 | 0.04688846751 | 0.2954987732 |
| **GO:0001909** | GO:0001909 | leukocyte mediated cytotoxicity | 8 | 0.6126857244 | 1.563924824 | 0.04706085158 | 0.04706085158 | 0.2956284395 |
| **GO:0002703** | GO:0002703 | regulation of leukocyte mediated immunity | 21 | 0.4528880436 | 1.553431042 | 0.04737091426 | 0.04737091426 | 0.296619365 |
| **GO:0072507** | GO:0072507 | divalent inorganic cation homeostasis | 32 | 0.4017733529 | 1.535914101 | 0.04815433693 | 0.04815433693 | 0.3005584457 |
| **GO:0009966** | GO:0009966 | regulation of signal transduction | 182 | 0.25391745 | 1.341771184 | 0.04910164903 | 0.04910164903 | 0.3046979623 |
| **GO:0010646** | GO:0010646 | regulation of cell communication | 196 | 0.2518002033 | 1.34470168 | 0.04913048766 | 0.04913048766 | 0.3046979623 |
| **GO:0051090** | GO:0051090 | regulation of DNA-binding transcription factor activity | 36 | 0.3808062816 | 1.500981555 | 0.0495731616 | 0.0495731616 | 0.3064673315 |
| **GO:0009968** | GO:0009968 | negative regulation of signal transduction | 72 | 0.3129792503 | 1.432712637 | 0.04990323472 | 0.04990323472 | 0.3075315931 |
