# Supplementary material for: A hepatocyte-specific transcriptional program driven by Rela and Stat3 exacerbates experimental colitis in mice by modulating bile synthesis
Source: eLife. 2024 Aug 13;12:RP93273. doi: 10.7554/eLife.93273 (PMC11321761; doi:10.7554/eLife.93273)
Supplement: Figure 1—source data 2. [file elife-93273-fig1-data2.docx]

|  |  |  |  |  |  |  |  |
| --- | --- | --- | --- | --- | --- | --- | --- |
| **Blot Quantification** |  |  |  |  |  |  |  |
| sample | prel | stat3 ser | stat3 tyr |  |  |  |  |
| D6 | 3.37301322 | 17.871526 | 4.4800465 |  |  |  |  |
| D6 | 1.13132733 | 16.747813 | 2.69036589 |  |  |  |  |
| D6 | 2.13994222 | 17.1907064 | 3.45024729 |  |  |  |  |
| D4 | 2.20268752 | 9.68023885 | 4.35725264 |  |  |  |  |
| D4 | 3.5012384 | 10.1364446 | 15.2227365 |  |  |  |  |
| D4 | 2.84117127 | 10.6262155 | 7.61868445 |  |  |  |  |
| D2 | 10.4455546 | 7.40188258 | 3.64555475 |  |  |  |  |
| D2 | 6.49267588 | 7.27456551 | 0.82433389 |  |  |  |  |
| D2 | 8.11498526 | 7.3598531 | 2.38913419 |  |  |  |  |
| C | 0.14639625 | 0.12867949 | 0.12423075 |  |  |  |  |
| C | 0.14639625 | 0.12867949 | 0.12423075 |  |  |  |  |
| C | 0.14639625 | 0.12867949 | 0.12423075 |  |  |  |  |
|  |  |  |  |  |  |  |  |
|  |  |  |  |  |  |  |  |
| **pRelA Quantification** |  |  |  |  |  |  |  |
| ANOVA summary |  |  | Dunnett's multiple comparisons test | Mean Diff. | 95.00% CI of diff. | Summary | Adjusted P Value |
| F | 22.6 |  | control vs. day2 | -7.351 | -10.14 to -4.561 | *** | 0.0002 |
| P value | 0.0003 |  | control vs. day4 | -1.848 | -4.638 to 0.9412 | ns | 0.2084 |
| P value summary | *** |  | control vs. day6 | -1.215 | -4.004 to 1.575 | ns | 0.4904 |
| Significant diff. among means (P < 0.05)? | Yes |  |  |  |  |  |  |
| R squared | 0.8945 |  |  |  |  |  |  |
| F (DFn, DFd) | 1.845 (3, 8) |  |  |  |  |  |  |
|  |  |  |  |  |  |  |  |
| **pStat3 ser Quantification** |  |  |  |  |  |  |  |
| ANOVA summary |  |  | Dunnett's multiple comparisons test | Mean Diff. | 95.00% CI of diff. | Summary | Adjusted P Value |
| F | 995.1 |  | control vs. day2 | -6.345 | -7.216 to -5.475 | **** | <0.0001 |
| P value | <0.0001 |  | control vs. day4 | -9.148 | -10.02 to -8.277 | **** | <0.0001 |
| P value summary | **** |  | control vs. day6 | -16.27 | -17.14 to -15.40 | **** | <0.0001 |
| Significant diff. among means (P < 0.05)? | Yes |  |  |  |  |  |  |
| R squared | 0.9973 |  |  |  |  |  |  |
| F (DFn, DFd) | 2.192 (3, 8) |  |  |  |  |  |  |
|  |  |  |  |  |  |  |  |
| **pStat3 tyr Quantification** |  |  |  |  |  |  |  |
| ANOVA summary |  |  | Dunnett's multiple comparisons test | Mean Diff. | 95.00% CI of diff. | Summary | Adjusted P Value |
| F | 4.463 |  | control vs. day2 | -1.286 | -8.130 to 5.558 | ns | 0.9057 |
| P value | 0.0403 |  | control vs. day4 | -8.066 | -14.91 to -1.222 | * | 0.0235 |
| P value summary | * |  | control vs. day6 | -2.54 | -9.384 to 4.304 | ns | 0.6009 |
| Significant diff. among means (P < 0.05)? | Yes |  |  |  |  |  |  |
| R squared | 0.626 |  |  |  |  |  |  |
| F (DFn, DFd) | 1.985 (3, 8) |  |  |  |  |  |  |
|  |  |  |  |  |  |  |  |
