# Supplementary material for: A hepatocyte-specific transcriptional program driven by Rela and Stat3 exacerbates experimental colitis in mice by modulating bile synthesis
Source: eLife. 2024 Aug 13;12:RP93273. doi: 10.7554/eLife.93273 (PMC11321761; doi:10.7554/eLife.93273)

STAT3-Ser727

Labelling:
M - Protein ladder
C - wild type animals without any treatment
D2 - wildtype animals with DSS treatment for 2 days.
D4 - wildtype animals with DSS treatment for 4 days.
D6 - wildtype animals with DSS treatment for 6 days.

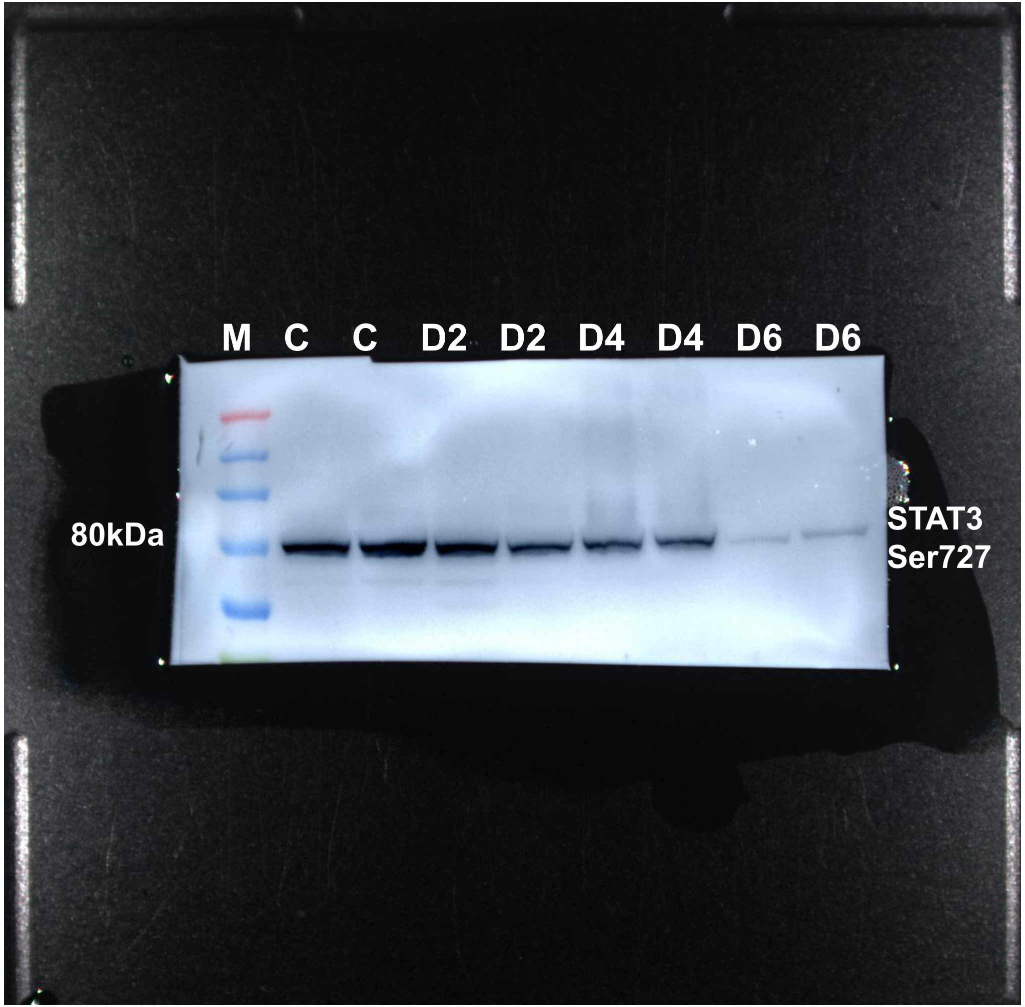

STAT3-Tyr705

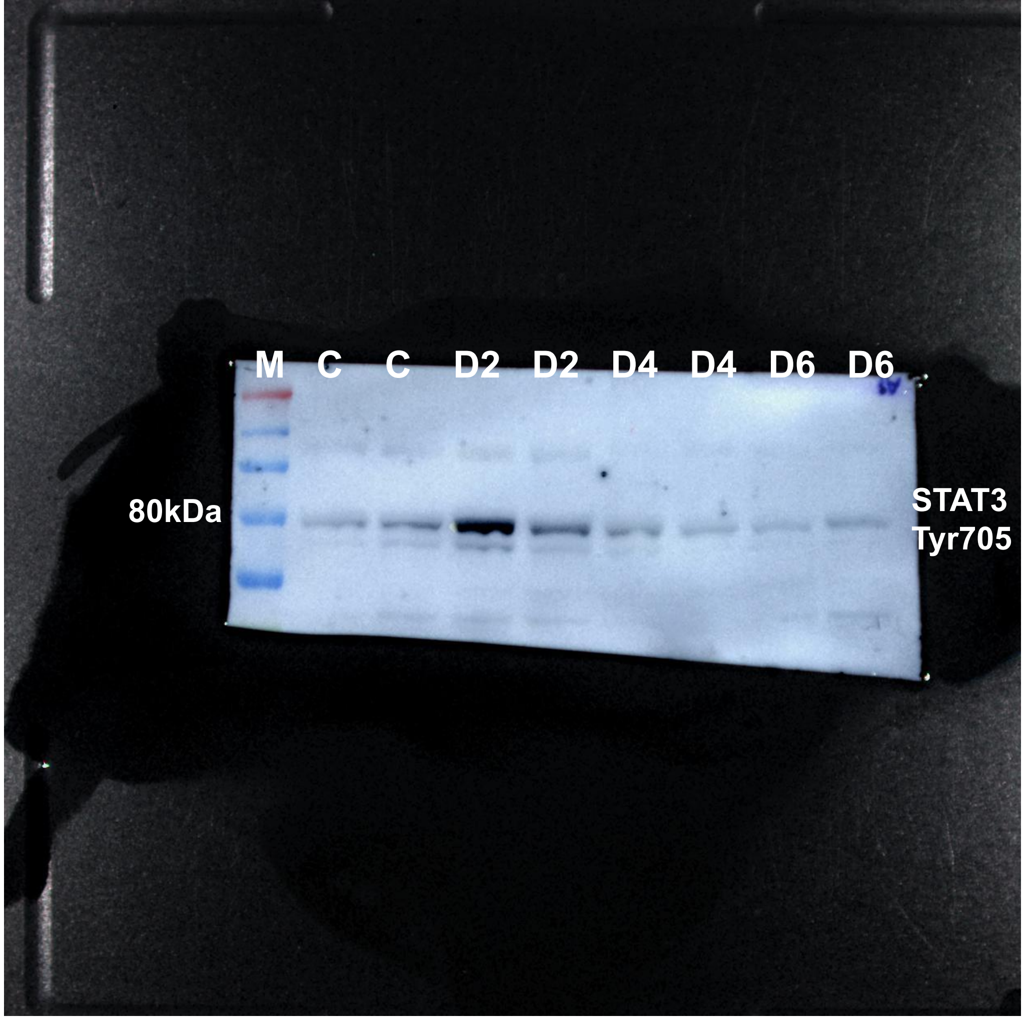

Total STAT3

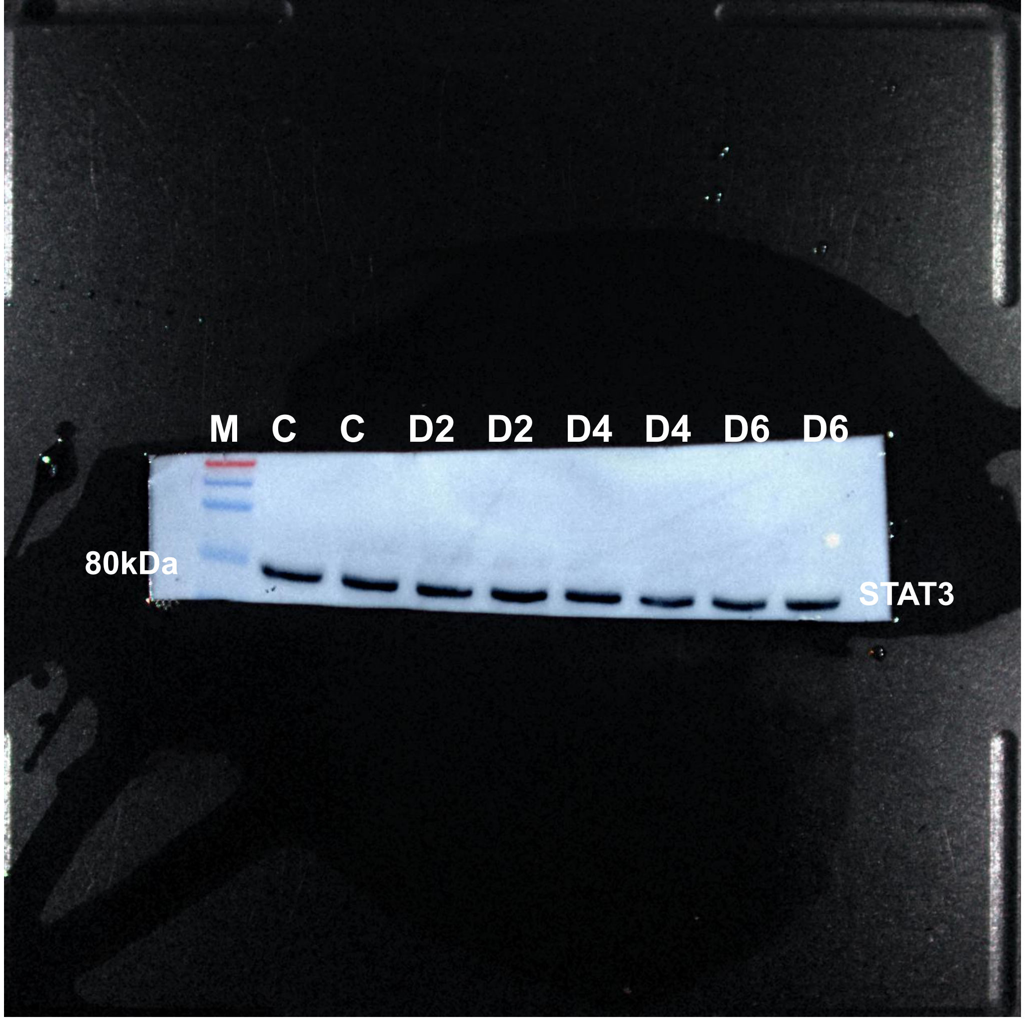

RelA-Ser536

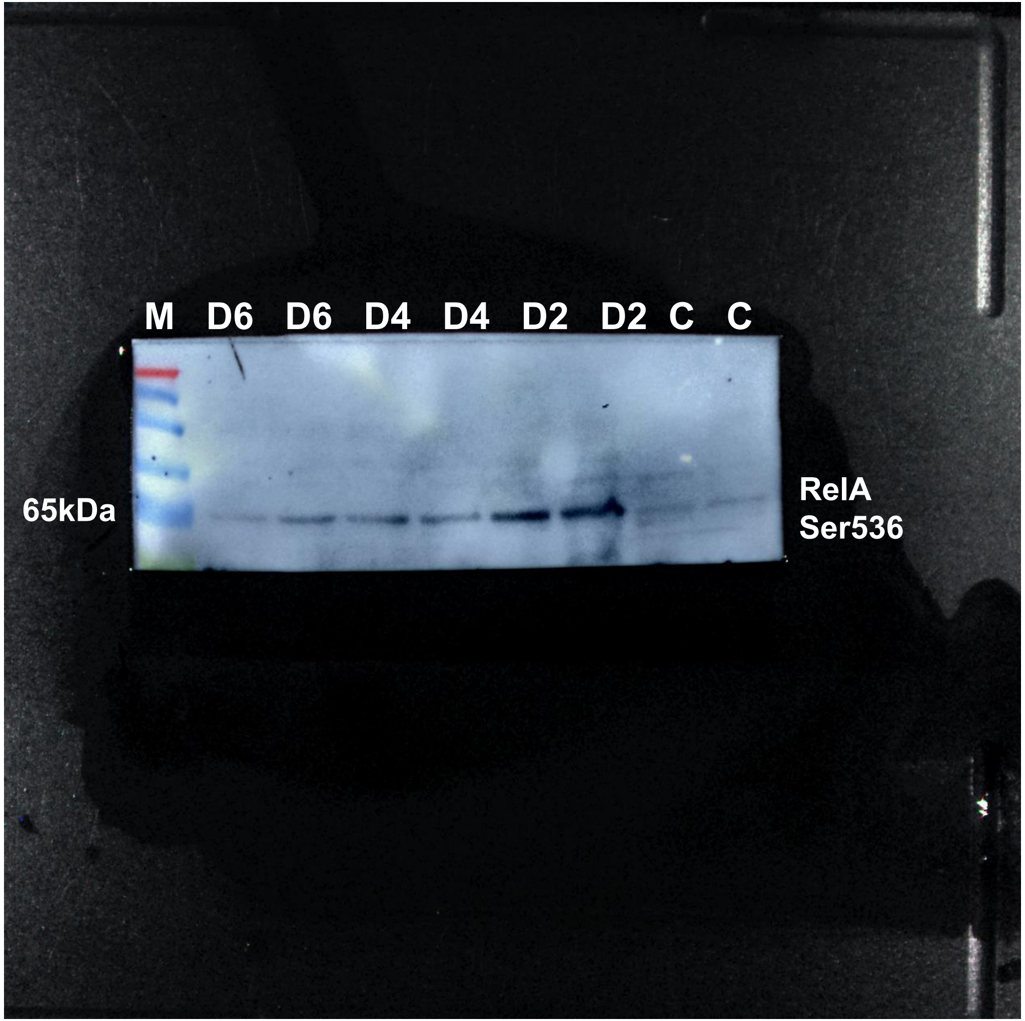

Total RelA

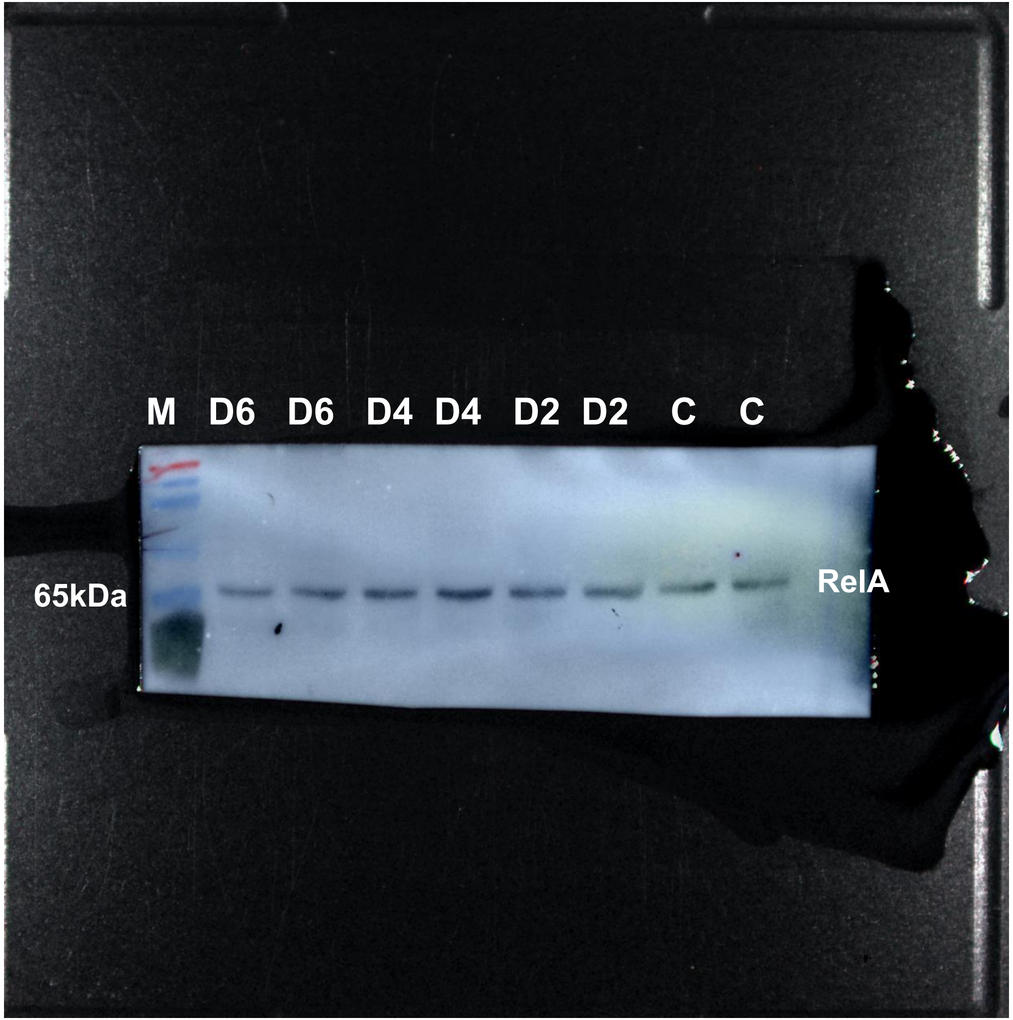

Beta actin

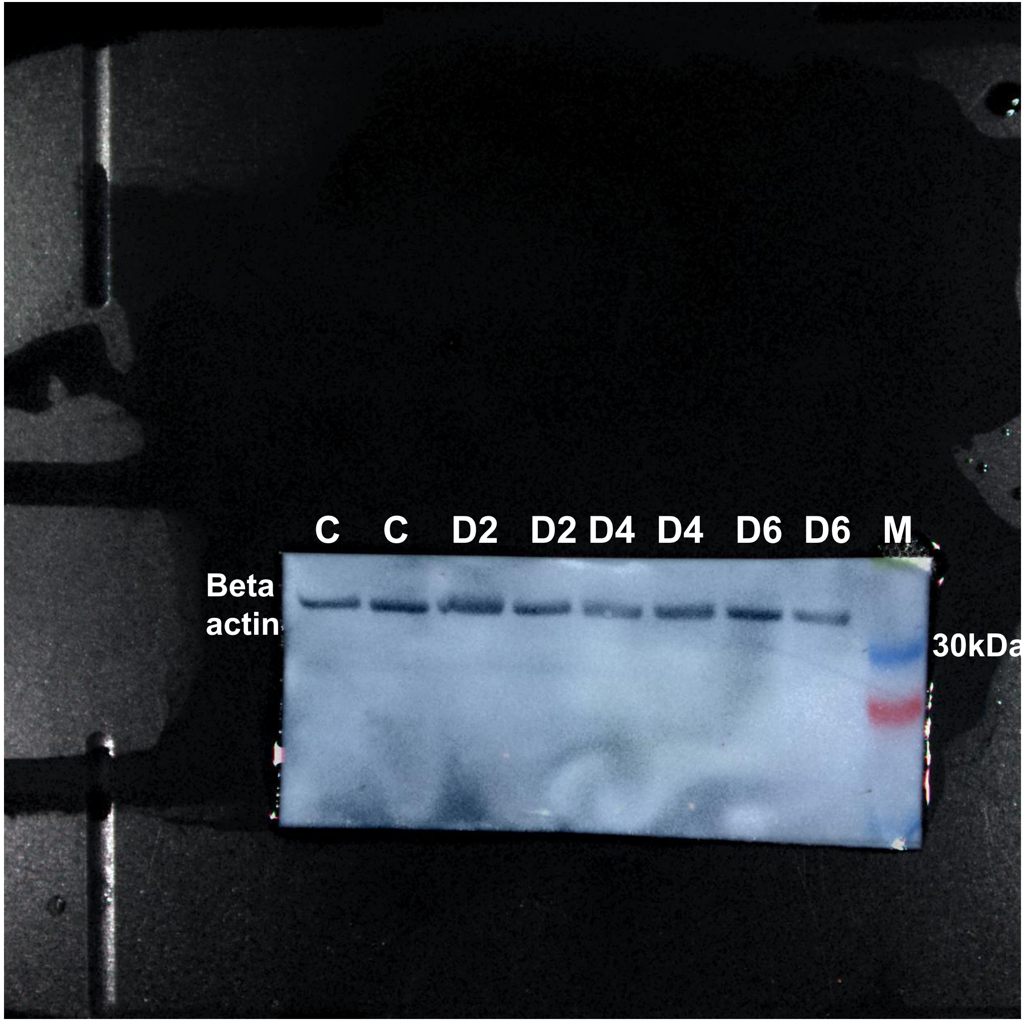

Supplement: Figure 1—source data 3. [file elife-93273-fig1-data3.pdf]
