# Supplementary figures and images for: A hepatocyte-specific transcriptional program driven by Rela and Stat3 exacerbates experimental colitis in mice by modulating bile synthesis
Source: eLife. 2024 Aug 13;12:RP93273. doi: 10.7554/eLife.93273 (PMC11321761; doi:10.7554/eLife.93273)

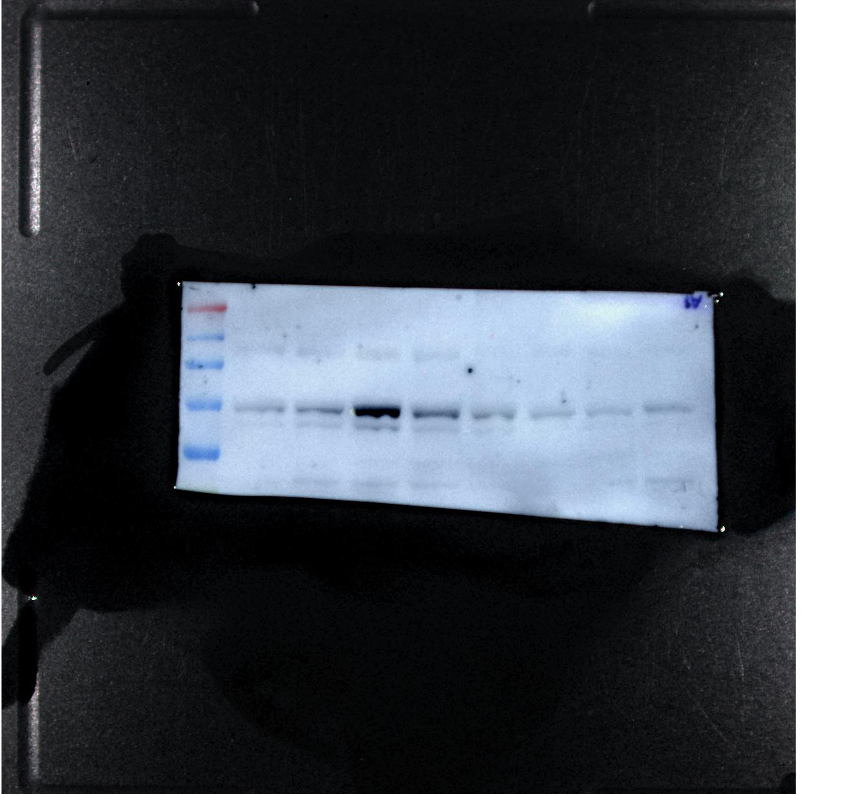

Supplement: Figure 1—source data 4. [file elife-93273-fig1-data4.zip › original blots_ fig1/fig 1_blot2.png]

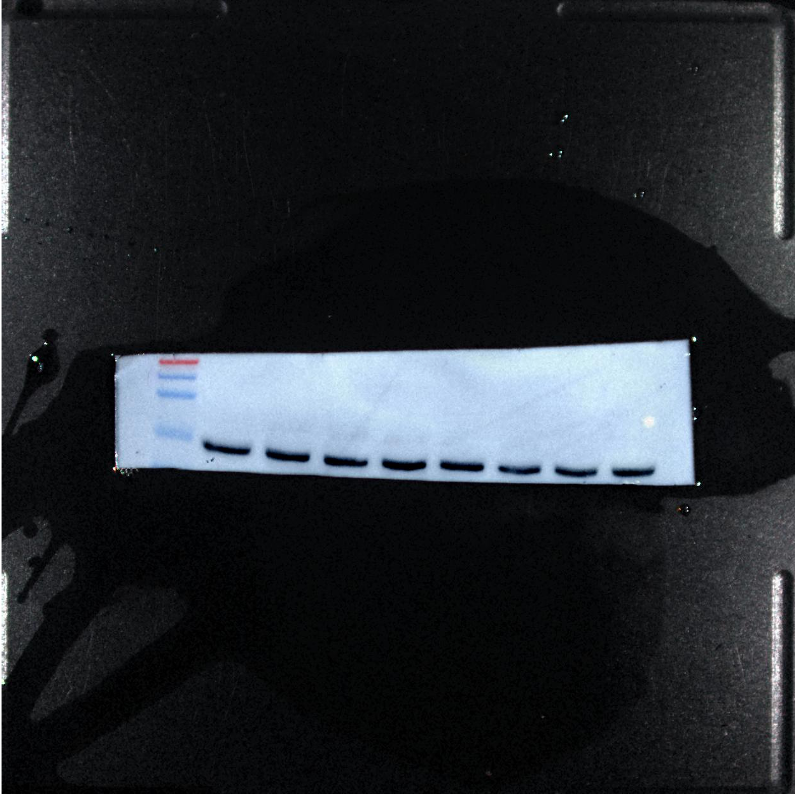

Supplement: Figure 1—source data 4. [file elife-93273-fig1-data4.zip › original blots_ fig1/fig 1_blot3.png]

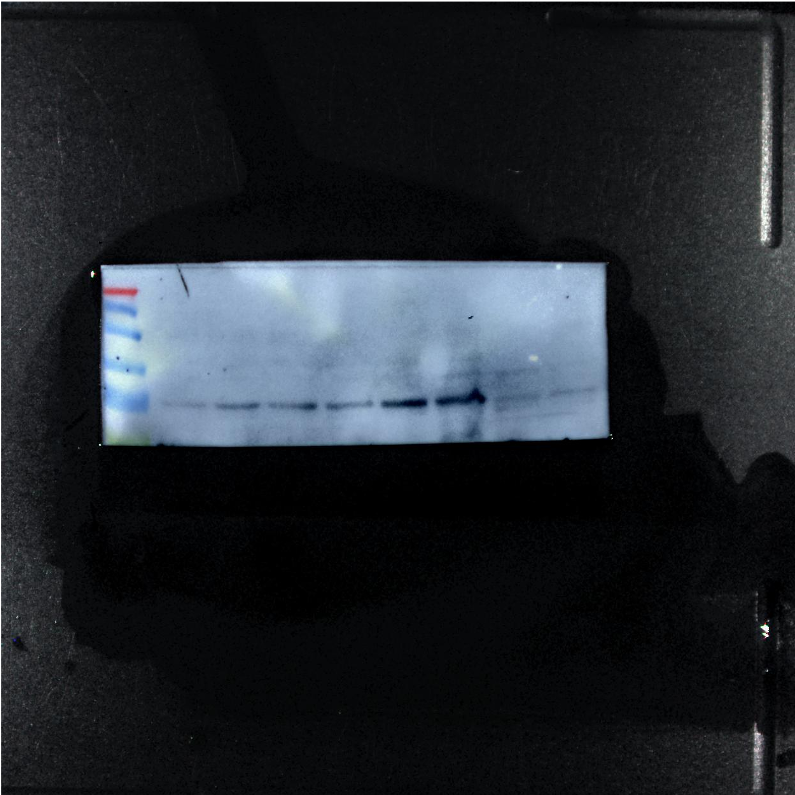

Supplement: Figure 1—source data 4. [file elife-93273-fig1-data4.zip › original blots_ fig1/fig 1_blot4.png]

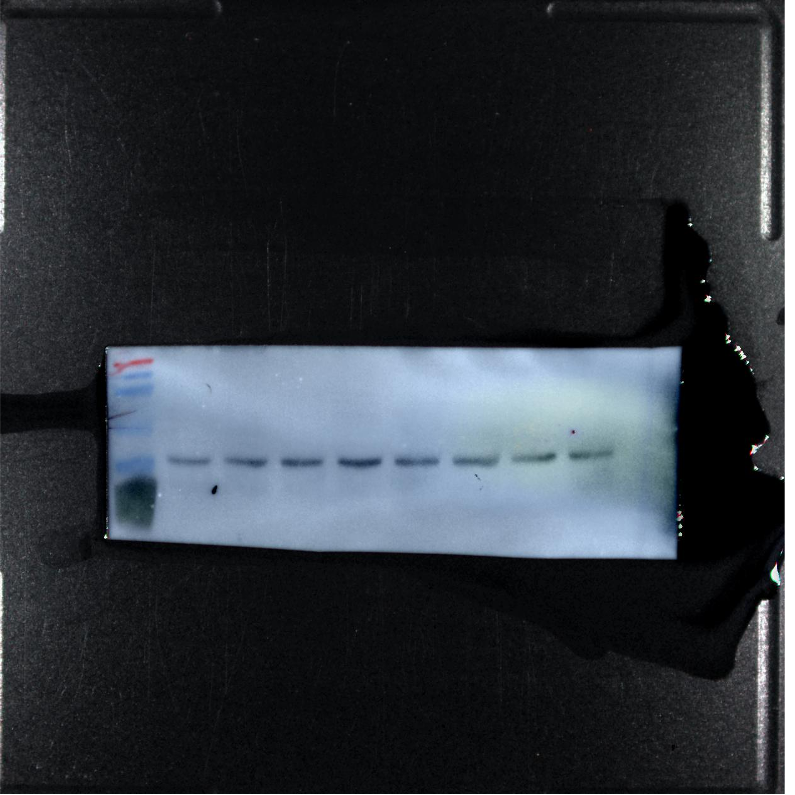

Supplement: Figure 1—source data 4. [file elife-93273-fig1-data4.zip › original blots_ fig1/fig 1_blot5.png]

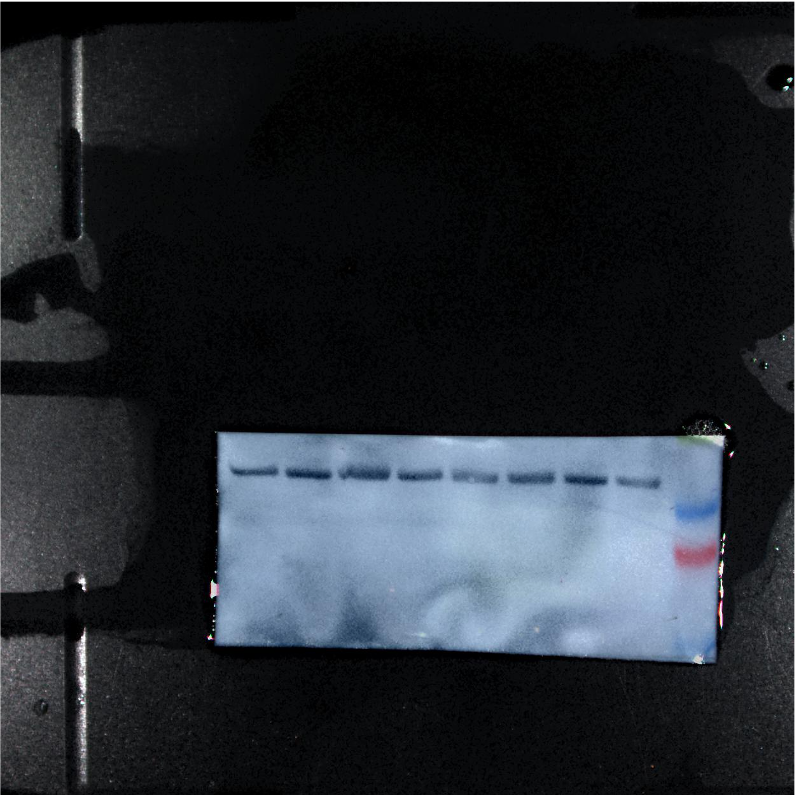

Supplement: Figure 1—source data 4. [file elife-93273-fig1-data4.zip › original blots_ fig1/fig 1_blot 6.png]

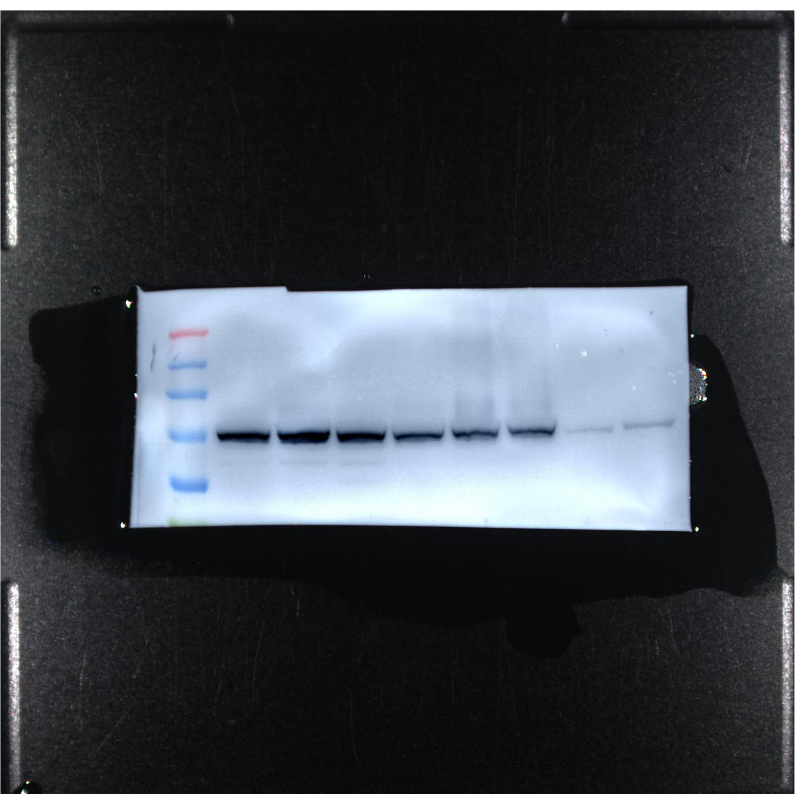

Supplement: Figure 1—source data 4. [file elife-93273-fig1-data4.zip › original blots_ fig1/fig 1_blot 1.png]

STAT3- Ser727

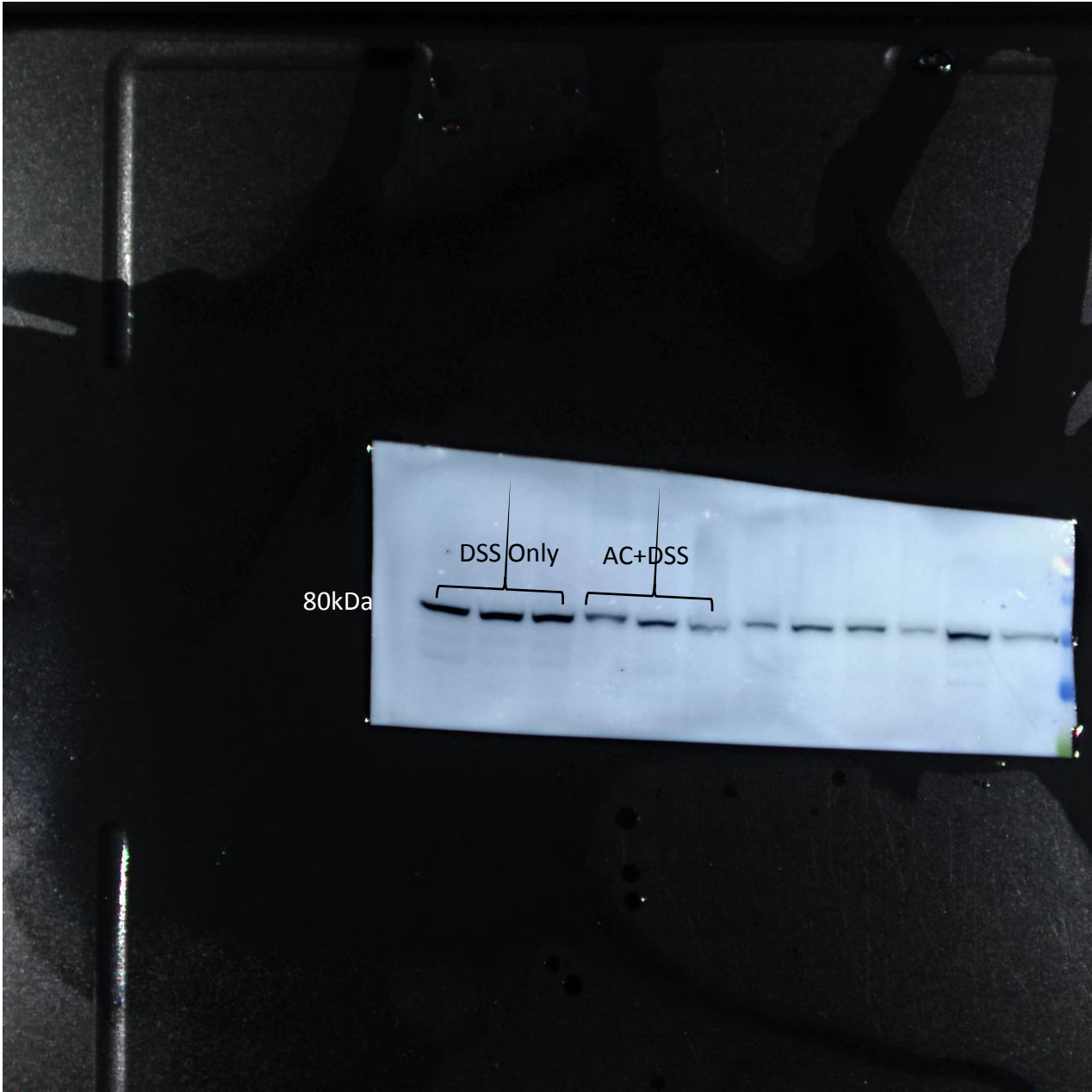

STAT3-  
Ser727

Total STAT3

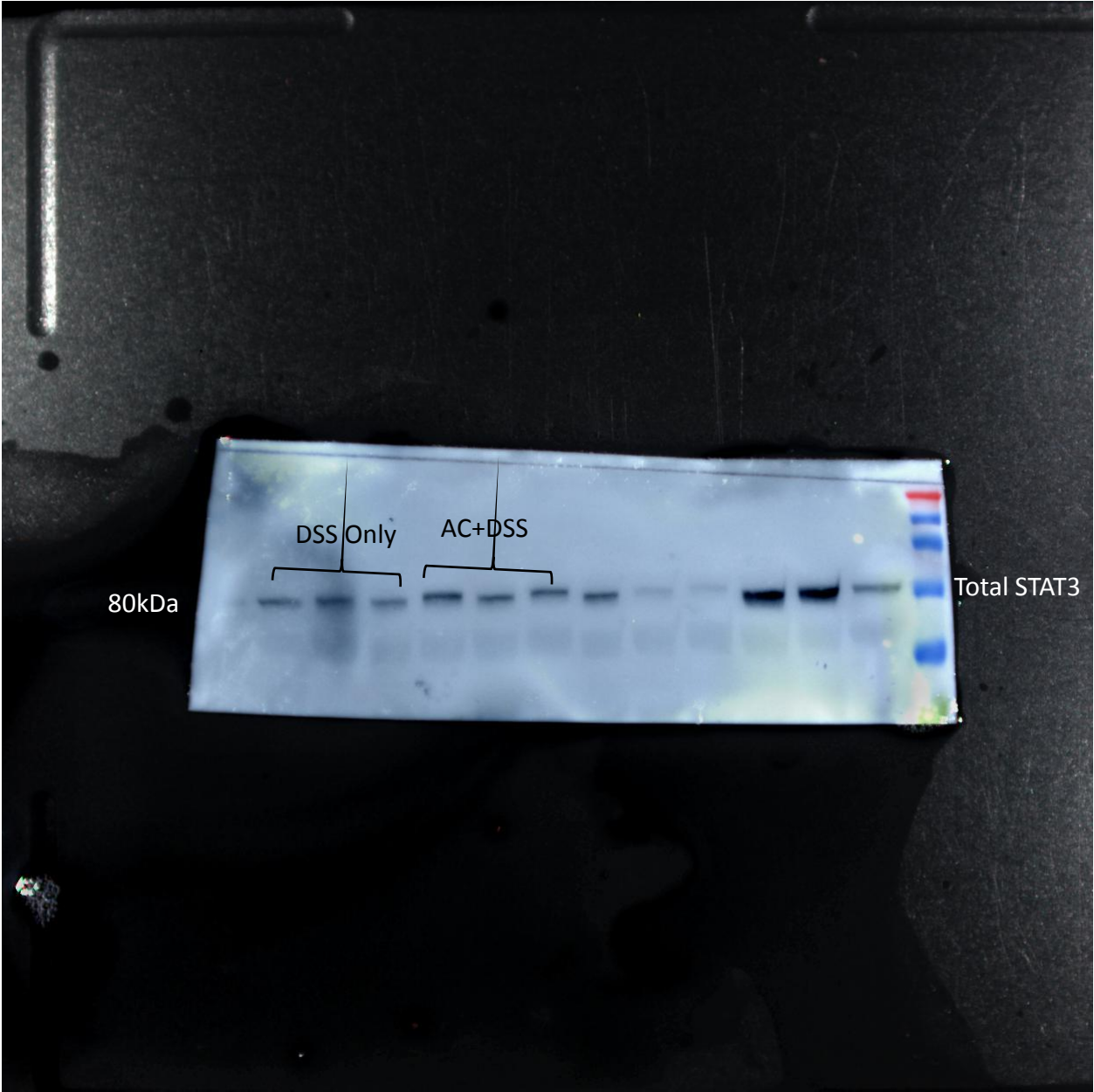

Beta actin

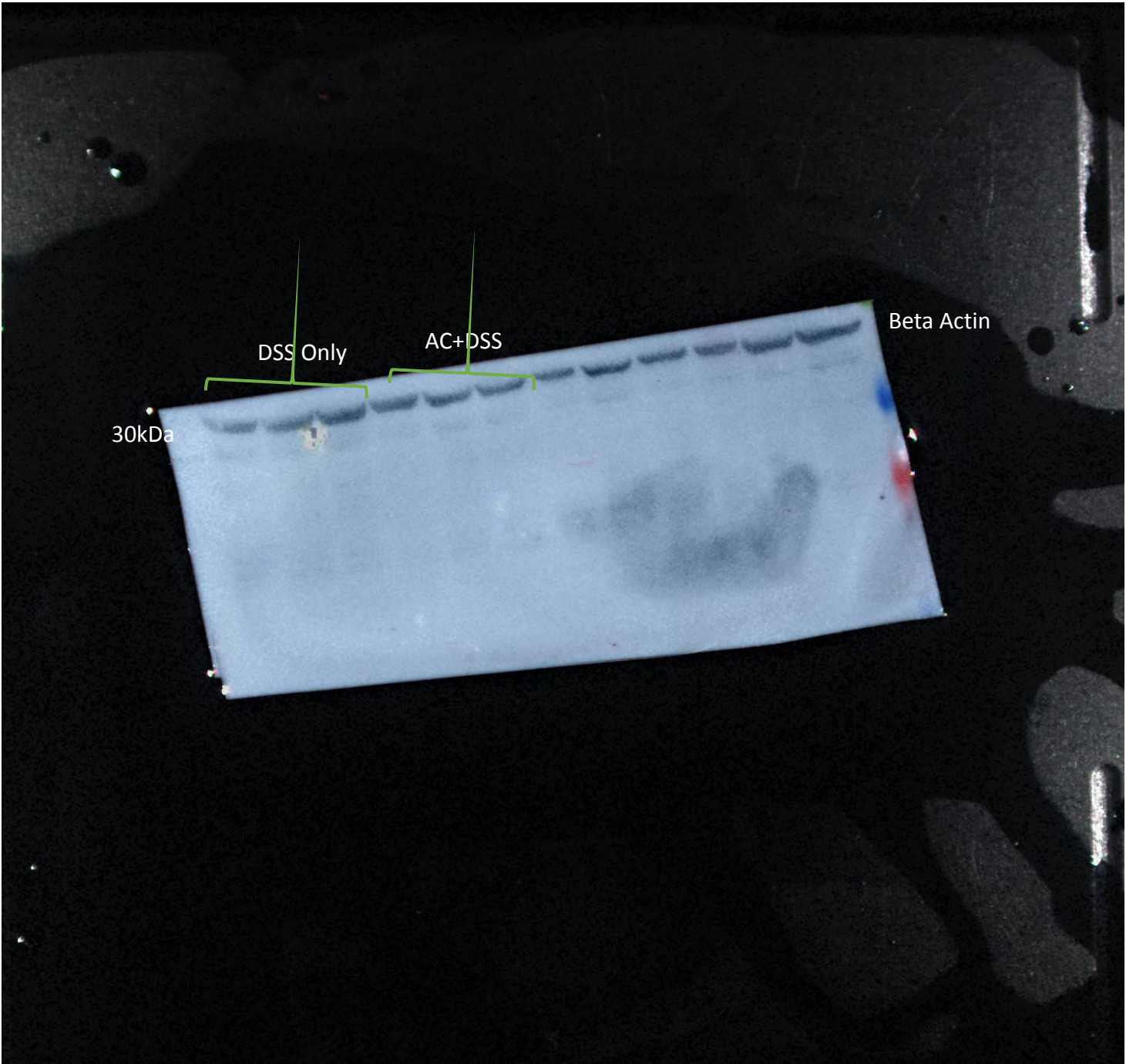

Supplement: Figure 1—figure supplement 1—source data 2. [file elife-93273-fig1-figsupp1-data2.pdf]

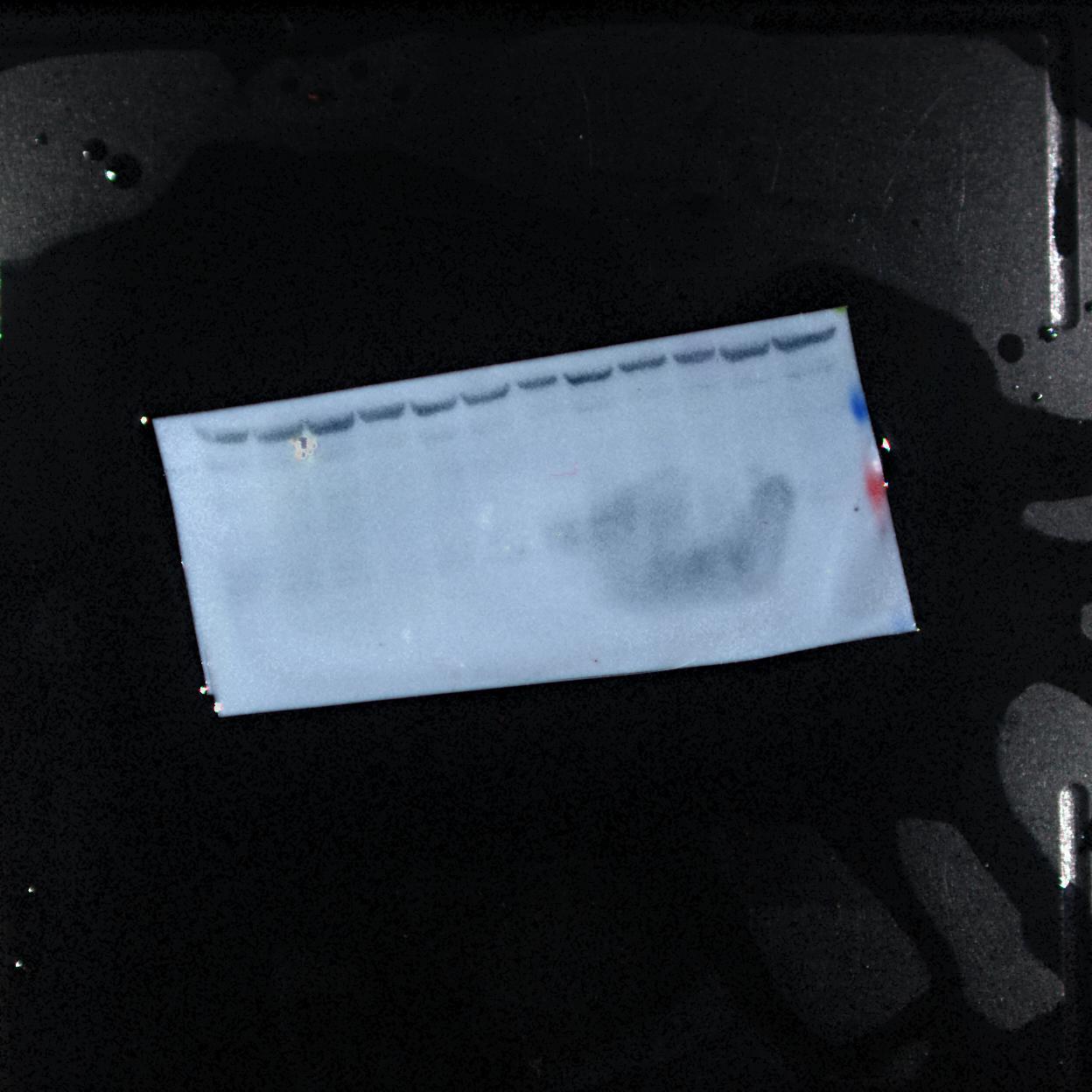

Supplement: Figure 1—figure supplement 1—source data 3. [file elife-93273-fig1-figsupp1-data3.zip › 7marACT 20240307_111139_Ch+Marker.jpg]

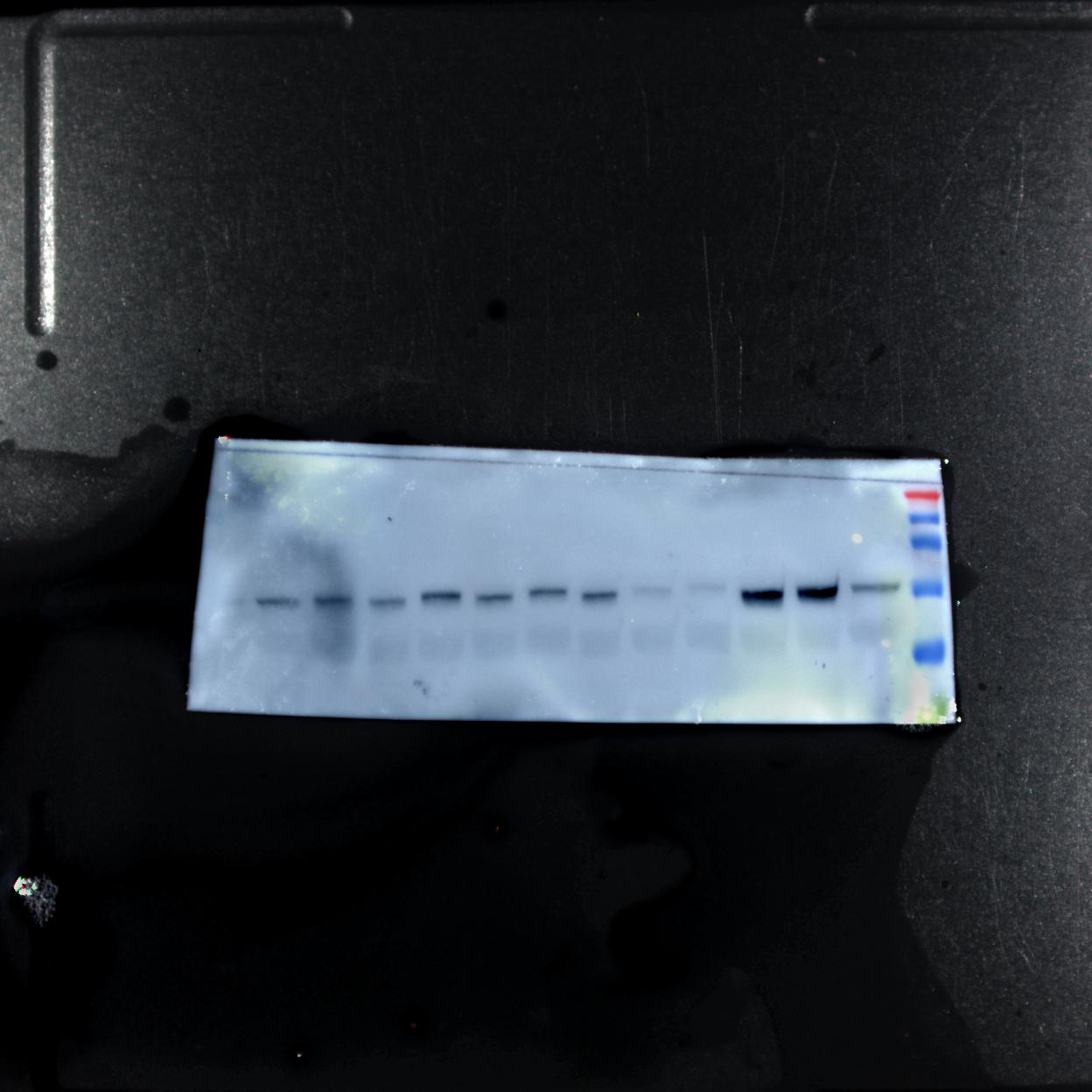

Supplement: Figure 1—figure supplement 1—source data 3. [file elife-93273-fig1-figsupp1-data3.zip › stat3 20240321_132743_Ch+Marker.jpg]

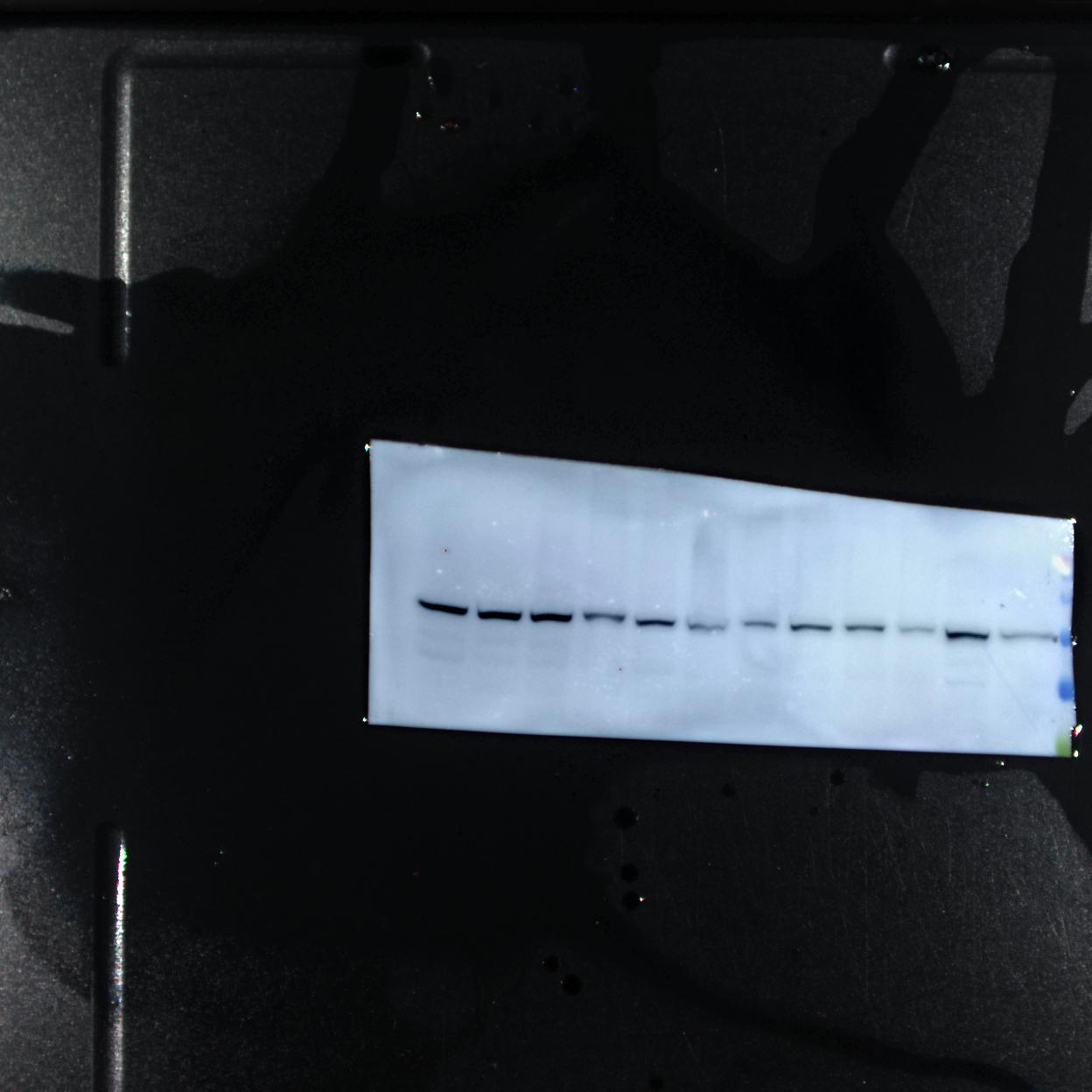

Supplement: Figure 1—figure supplement 1—source data 3. [file elife-93273-fig1-figsupp1-data3.zip › stat3P727 20240307_104906_Ch+Marker.jpg]

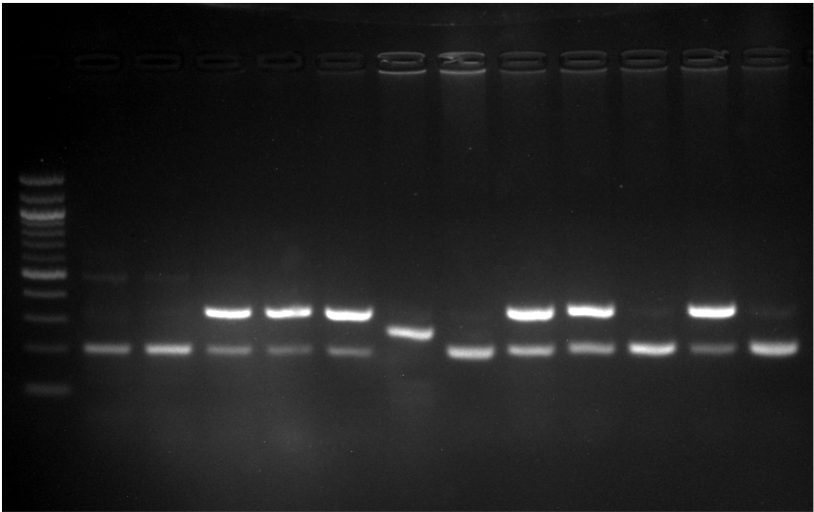

Supplement: Figure 2—figure supplement 1—source data 2. [file elife-93273-fig2-figsupp1-data2.zip › original gels/sup_gel1.png]

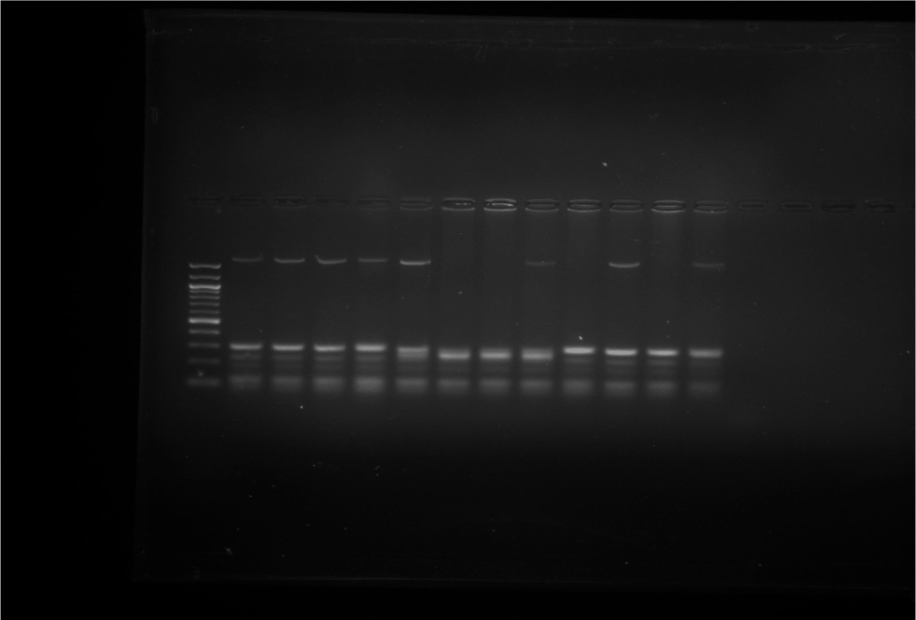

Supplement: Figure 2—figure supplement 1—source data 2. [file elife-93273-fig2-figsupp1-data2.zip › original gels/sup_gel3.png]

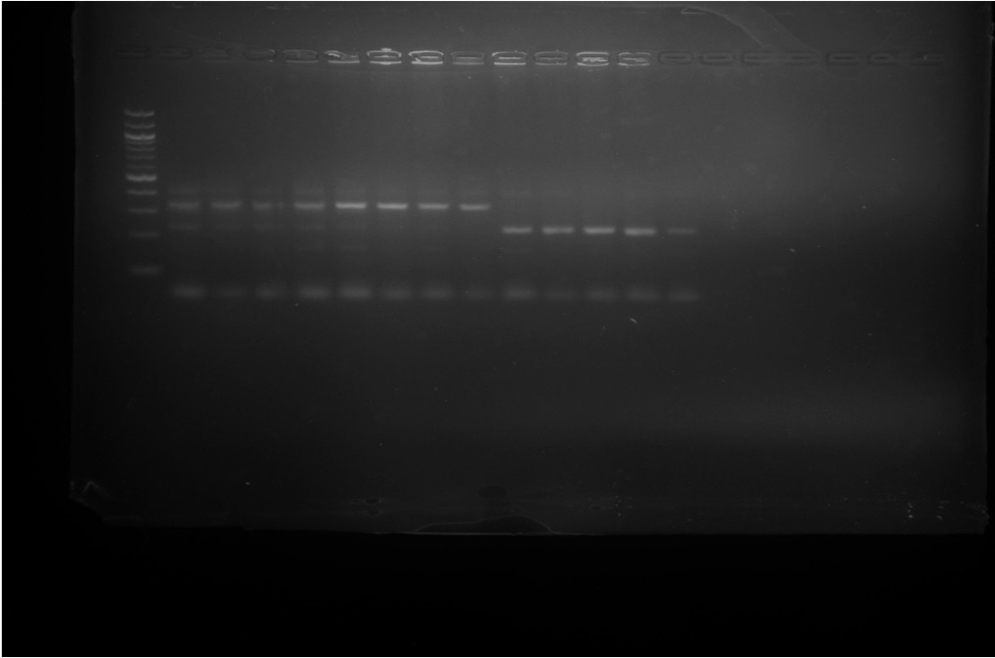

Supplement: Figure 2—figure supplement 1—source data 2. [file elife-93273-fig2-figsupp1-data2.zip › original gels/sup_gel2.png]

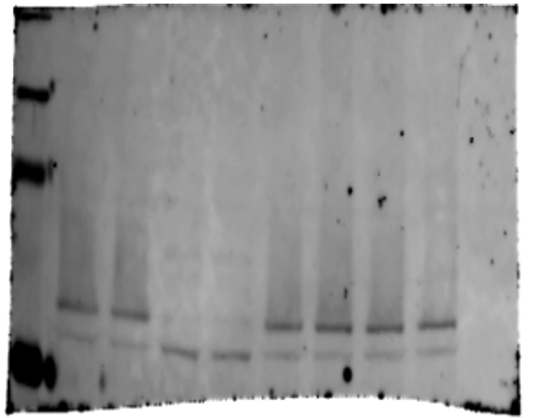

Supplement: Figure 2—figure supplement 1—source data 4. [file elife-93273-fig2-figsupp1-data4.zip › original blots_sup/Sup_blot1.png]

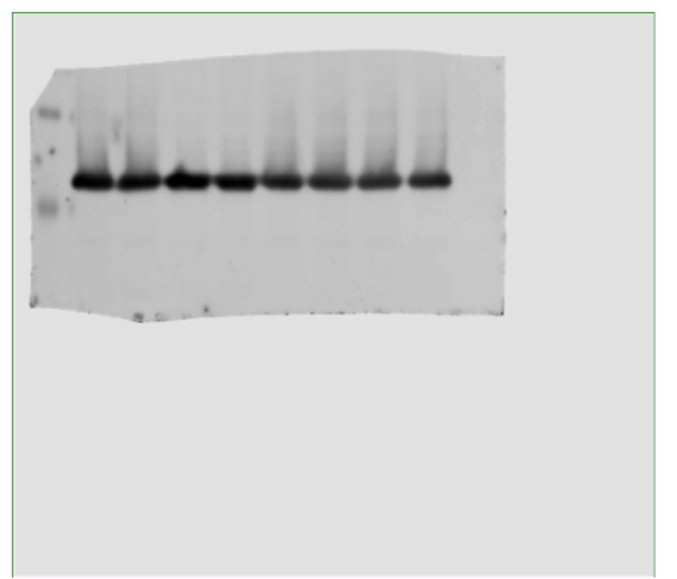

Supplement: Figure 2—figure supplement 1—source data 4. [file elife-93273-fig2-figsupp1-data4.zip › original blots_sup/Sup_blot3.png]

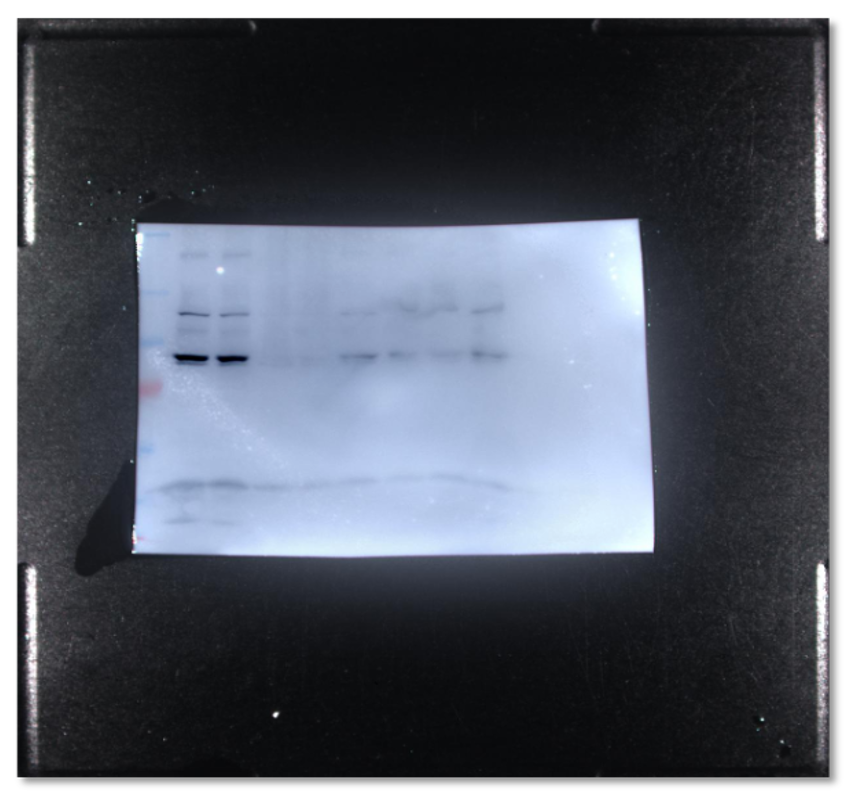

Supplement: Figure 2—figure supplement 1—source data 4. [file elife-93273-fig2-figsupp1-data4.zip › original blots_sup/Sup_blot2.png]
