# Supplementary material for: A hepatocyte-specific transcriptional program driven by Rela and Stat3 exacerbates experimental colitis in mice by modulating bile synthesis
Source: eLife. 2024 Aug 13;12:RP93273. doi: 10.7554/eLife.93273 (PMC11321761; doi:10.7554/eLife.93273)
Supplement: Figure 1—figure supplement 1—source data 1. [file elife-93273-fig1-figsupp1-data1.docx]

|  |  |  |  |
| --- | --- | --- | --- |
| **SGPT** | **wt_con** | **wt_d4** | **wt_d6** |
|  | 81.82 | 74.58 | 67.87 |
|  | 68.98 | 62.4 | 89.46 |
|  | 137.34 | 49.21 | 78.42 |
|  |  |  |  |
| **Bilurubin-D** | **wt_con** | **wt_d4** | **wt_d6** |
|  | 0.68 | 0.7 | 1.03 |
|  | 0.31 | 1.12 | 0.89 |
|  | 1.06 | 1.02 | 0.95 |
|  |  |  |  |
| **GGT** | **wt_con** | **wt_d4** | **wt_d6** |
|  | 6.57 | 6.99 | 11.73 |
|  | 4.31 | 8.92 | 7.92 |
|  | 9.84 | 9.7 | 9.95 |
|  |  |  |  |
| **SGOT** | **wt_con** | **wt_d4** | **wt_d6** |
|  | 137.98 | 135.13 | 123.8 |
|  | 119.35 | 124.26 | 145.31 |
|  | 302.92 | 119.61 | 134.35 |
