# Supplementary material for: A hepatocyte-specific transcriptional program driven by Rela and Stat3 exacerbates experimental colitis in mice by modulating bile synthesis
Source: eLife. 2024 Aug 13;12:RP93273. doi: 10.7554/eLife.93273 (PMC11321761; doi:10.7554/eLife.93273)
Supplement: Figure 2—source data 1. [file elife-93273-fig2-data1.docx]

| **Disease activity index** |  |  |  |  |  | |  |  |  |  |  | |  |  |  |  |  |
| --- | --- | --- | --- | --- | --- | --- | --- | --- | --- | --- | --- | --- | --- | --- | --- | --- | --- |
| Days post DSS treatment | **WT** | | | | | **rela-/-** | | | | | | **stat3-/-** | | | | | |
| 1 | 0 | 0 | 0 | 0 | 0 | | 0 | 0 | 0 | 0 | 0 | | 0 | 0 | 0 | 0 | 0 |
| 2 | 0 | 0 | 0 | 0 | 0 | | 0 | 0 | 0 | 0 | 0 | | 0 | 0 | 0 | 0 | 0 |
| 3 | 0 | 0 | 0 | 0 | 0 | | 0 | 0 | 1 | 0 | 0 | | 0 | 0 | 0 | 0 | 0 |
| 4 | 1 | 2 | 1 | 2 | 1 | | 1 | 1 | 2 | 1 | 1 | | 1 | 1 | 1 | 1 | 1 |
| 5 | 2 | 2 | 2 | 3 | 3 | | 2 | 2 | 2 | 2 | 2 | | 1 | 2 | 1 | 2 | 1 |
| 6 | 2 | 2 | 2 | 3 | 3 | | 2 | 2 | 2 | 3 | 3 | | 2 | 2 | 2 | 2 | 2 |
|  |  |  |  |  |  | |  |  |  |  |  | |  |  |  |  |  |
|  |  |  |  |  |  | |  |  |  |  |  | |  |  |  |  |  |
|  |  |  |  |  |  | |  |  |  |  |  | |  |  |  |  |  |
|  |  |  |  |  |  | |  |  |  |  |  | |  |  |  |  |  |
| **WT vs dKO** |  |  |  |  |  | |  |  |  |  |  | |  |  |  |  |  |
| Test details |  |  | Below threshold? | P value | Mean of WT | | Mean of rela-/-stat3-/- | Difference | SE of difference | t ratio | df | | Adjusted P Value |  |  |  |  |
| Test name | Unpaired t test |  |  |  | 0 | | 0 | 0 | 0 |  |  | |  |  |  |  |  |
| Variance assumption | Individual variance for each row |  |  |  | 0 | | 0 | 0 | 0 |  |  | |  |  |  |  |  |
| Multiple comparisons | Set P value threshold |  |  |  | 0 | | 0 | 0 | 0 |  |  | |  |  |  |  |  |
| Method | Holm-Šídák method |  | Yes | 0.006635 | 1.667 | | 0.2 | 1.467 | 0.3611 | 4.062 | 6 | | 0.006635 |  |  |  |  |
| Alpha | 0.05 |  | Yes | 0.00001 | 2.4 | | 0 | 2.4 | 0.2449 | 9.798 | 8 | | 0.000049 |  |  |  |  |
|  |  |  | Yes | 0.001476 | 2.5 | | 0.6 | 1.9 | 0.3761 | 5.052 | 7 | | 0.004867 |  |  |  |  |
|  |  |  | Yes | 0.001219 | 2.75 | | 0.9 | 1.85 | 0.3541 | 5.225 | 7 | | 0.004867 |  |  |  |  |
|  |  |  | Yes | 0.001496 | 2.5 | | 1.1 | 1.4 | 0.2777 | 5.041 | 7 | | 0.004867 |  |  |  |  |
|  |  |  |  |  |  | |  |  |  |  |  | |  |  |  |  |  |
|  |  |  |  |  |  | |  |  |  |  |  | |  |  |  |  |  |
|  |  |  |  |  |  | |  |  |  |  |  | |  |  |  |  |  |
