# Supplementary material for: A hepatocyte-specific transcriptional program driven by Rela and Stat3 exacerbates experimental colitis in mice by modulating bile synthesis
Source: eLife. 2024 Aug 13;12:RP93273. doi: 10.7554/eLife.93273 (PMC11321761; doi:10.7554/eLife.93273)
Supplement: Figure 2—source data 2. [file elife-93273-fig2-data2.docx]

| **colon length** |  |  |  |  |  |  |  |  |
| --- | --- | --- | --- | --- | --- | --- | --- | --- |
| **(in cms)** | **WT_Con** | **WT_DSS** | **AR_Con** | **AR_DSS** | **AS_Con** | **AS_DSS** | **dKO_Con** | **dKO_DSS** |
| rep1 | 7.9 | 5.2 | 7.8 | 5.5 | 7.2 | 5.5 | 7.3 | 6.9 |
| rep2 | 7.2 | 4.6 | 7.3 | 6.1 | 7.8 | 5.6 | 7.5 | 6.5 |
| rep3 | 7.4 | 5.2 | 7.2 | 5.2 | 7 | 5.6 | 7 | 6.5 |
| rep4 | 7.3 | 4.5 | 7.1 | 5.7 | 7.4 | 6 | 7.3 | 7 |
|  |  |  |  |  |  |  |  |  |
|  |  |  |  |  |  |  |  |  |
|  |  |  |  |  |  |  |  |  |
|  | ANOVA summary |  |  | Tukey's multiple comparisons test | Mean Diff. | 95.00% CI of diff. | Summary | Adjusted P Value |
|  | F | 42.78 |  | WT_Con vs. WT_DSS | 2.575 | 1.855 to 3.295 | **** | <0.0001 |
|  | P value | <0.0001 |  | WT_Con vs. AR_Con | 0 | -0.6198 to 0.8198 | ns | 0.9997 |
|  | P value summary | **** |  | WT_Con vs. AR_DSS | 0.1 | 1.105 to 2.545 | **** | <0.0001 |
|  | Significant diff. among means (P < 0.05)? | Yes |  | WT_Con vs. AS_Con | 1.825 | -0.6198 to 0.8198 | ns | 0.9997 |
|  | R squared | 0.9258 |  | WT_Con vs. AS_DSS | 1.775 | 1.055 to 2.495 | **** | <0.0001 |
|  |  |  |  | WT_Con vs. dKO_Con | 0.175 | -0.5448 to 0.8948 | ns | 0.9912 |
|  | Brown-Forsythe test |  |  | WT_Con vs. dKO_DSS | 0.725 | 0.005167 to 1.445 | * | 0.0475 |
|  | F (DFn, DFd) | 0.6017 (7, 24) |  | WT_DSS vs. AR_Con | -2.475 | -3.195 to -1.755 | **** | <0.0001 |
|  |  |  |  | WT_DSS vs. AR_DSS | -0.75 | -1.470 to -0.03017 | * | 0.0369 |
|  |  |  |  | WT_DSS vs. AS_Con | -2.475 | -3.195 to -1.755 | **** | <0.0001 |
|  |  |  |  | WT_DSS vs. AS_DSS | -0.8 | -1.520 to -0.08017 | * | 0.022 |
|  |  |  |  | WT_DSS vs. dKO_Con | -2.4 | -3.120 to -1.680 | **** | <0.0001 |
|  |  |  |  | WT_DSS vs. dKO_DSS | -1.85 | -2.570 to -1.130 | **** | <0.0001 |
|  |  |  |  | AR_Con vs. AR_DSS | 1.725 | 1.005 to 2.445 | **** | <0.0001 |
|  |  |  |  | AR_Con vs. AS_Con | 0 | -0.7198 to 0.7198 | ns | >0.9999 |
|  |  |  |  | AR_Con vs. AS_DSS | 1.675 | 0.9552 to 2.395 | **** | <0.0001 |
|  |  |  |  | AR_Con vs. dKO_Con | 0.075 | -0.6448 to 0.7948 | ns | >0.9999 |
|  |  |  |  | AR_Con vs. dKO_DSS | 0.625 | -0.09483 to 1.345 | ns | 0.1226 |
|  |  |  |  | AR_DSS vs. AS_Con | -1.725 | -2.445 to -1.005 | **** | <0.0001 |
|  |  |  |  | AR_DSS vs. AS_DSS | -0.05 | -0.7698 to 0.6698 | ns | >0.9999 |
|  |  |  |  | AR_DSS vs. dKO_Con | -1.65 | -2.370 to -0.9302 | **** | <0.0001 |
|  |  |  |  | AR_DSS vs. dKO_DSS | -1.1 | -1.820 to -0.3802 | *** | 0.0008 |
|  |  |  |  | AS_Con vs. AS_DSS | 1.675 | 0.9552 to 2.395 | **** | <0.0001 |
|  |  |  |  | AS_Con vs. dKO_Con | 0.075 | -0.6448 to 0.7948 | ns | >0.9999 |
|  |  |  |  | AS_Con vs. dKO_DSS | 0.625 | -0.09483 to 1.345 | ns | 0.1226 |
|  |  |  |  | AS_DSS vs. dKO_Con | -1.6 | -2.320 to -0.8802 | **** | <0.0001 |
|  |  |  |  | AS_DSS vs. dKO_DSS | -1.05 | -1.770 to -0.3302 | ** | 0.0014 |
|  |  |  |  | dKO_Con vs. dKO_DSS | 0.55 | -0.1698 to 1.270 | ns | 0.2302 |
