# Supplementary material for: A hepatocyte-specific transcriptional program driven by Rela and Stat3 exacerbates experimental colitis in mice by modulating bile synthesis
Source: eLife. 2024 Aug 13;12:RP93273. doi: 10.7554/eLife.93273 (PMC11321761; doi:10.7554/eLife.93273)
Supplement: Figure 2—source data 3. [file elife-93273-fig2-data3.docx]

| **Pearmeability Assay** |  |  |  |  |  |  |  |  |  |
| --- | --- | --- | --- | --- | --- | --- | --- | --- | --- |
| **WT_Untreated** | **WT_Treated** | **dKO_Untreated** | **dKO_Treated** |  |  |  |  |  |  |
| 230 | 2526 | 210 | 1388 |  |  |  |  |  |  |
| 280 | 2041 | 260 | 1020 |  |  |  |  |  |  |
| 210 | 2496 | 250 | 1081 |  |  |  |  |  |  |
|  |  |  |  |  |  |  |  |  |  |
|  |  |  |  |  |  |  |  |  |  |
| P value | 0.0048 |  |  |  |  |  |  |  |  |
| P value summary | ** |  |  |  |  |  |  |  |  |
| Significantly different (P < 0.05)? | Yes |  |  |  |  |  |  |  |  |
| One- or two-tailed P value? | Two-tailed |  |  |  |  |  |  |  |  |
| Welch-corrected t, df | t=6.145, df=3.649 |  |  |  |  |  |  |  |  |
|  |  |  |  |  |  |  |  |  |  |
| ANOVA summary |  |  | Tukey's multiple comparisons test | Mean Diff. | 95.00% CI of diff. | Below threshold? | Summary | Adjusted P Value |  |
| F | 105.1 |  | WT_Untreated vs. WT_Treated | -2114 | -2557 to -1671 | Yes | **** | <0.0001 | A-B |
| P value | <0.0001 |  | WT_Untreated vs. dKO_Untreated | 0 | -442.9 to 442.9 | No | ns | >0.9999 | A-C |
| P value summary | **** |  | WT_Untreated vs. dKO_Treated | -923 | -1366 to -480.1 | Yes | *** | 0.0007 | A-D |
| Significant diff. among means (P < 0.05)? | Yes |  | WT_Treated vs. dKO_Untreated | 2114 | 1671 to 2557 | Yes | **** | <0.0001 | B-C |
| R squared | 0.9753 |  | WT_Treated vs. dKO_Treated | 1191 | 748.5 to 1634 | Yes | *** | 0.0001 | B-D |
| F (DFn, DFd) | 0.6812 (3, 8) |  | dKO_Untreated vs. dKO_Treated | -923 | -1366 to -480.1 | Yes | *** | 0.0007 | C-D |
