# Supplementary material for: A hepatocyte-specific transcriptional program driven by Rela and Stat3 exacerbates experimental colitis in mice by modulating bile synthesis
Source: eLife. 2024 Aug 13;12:RP93273. doi: 10.7554/eLife.93273 (PMC11321761; doi:10.7554/eLife.93273)
Supplement: Figure 2—source data 4. [file elife-93273-fig2-data4.docx]

| **RT-qPCR** |  |  |  |  |  |  |  |
| --- | --- | --- | --- | --- | --- | --- | --- |
| **tj1** | **WT_control** | **WT_D6** | **KO_control** | **KO_D6** |  | **Unpaired t test** |  |
|  | 1 | 0.4 | 1 | 0.86 |  | P value | 0.0238 |
|  | 1 | 0.44 | 1 | 0.6 |  | P value summary | * |
|  | 1 | 0.19 | 1 | 0.72 |  | Significantly different (P < 0.05)? | Yes |
|  |  |  |  |  |  | One- or two-tailed P value? | Two-tailed |
|  |  |  |  |  |  | Welch-corrected t, df | t=3.551, df=3.996 |
|  |  |  |  |  |  |  |  |
|  |  |  |  |  |  |  |  |
| **occ** | **WT_control** | **WT_D6** | **KO_control** | **KO_D6** |  | **Unpaired t test** |  |
|  | 1 | 0.6 | 1 | 1.06 |  | P value | 0.0032 |
|  | 1 | 0.62 | 1 | 1.2 |  | P value summary | ** |
|  | 1 | 0.4 | 1 | 1.2 |  | Significantly different (P < 0.05)? | Yes |
|  |  |  |  |  |  | One- or two-tailed P value? | Two-tailed |
|  |  |  |  |  |  | Welch-corrected t, df | t=7.273, df=3.478 |
|  |  |  |  |  |  |  |  |
|  |  |  |  |  |  |  |  |
| **tff3** | **WT_control** | **WT_D6** | **KO_control** | **KO_D6** |  | **Unpaired t test** |  |
|  | 1 | 0.32 | 1 | 1.27 |  | P value | 0.0002 |
|  | 1 | 0.44 | 1 | 1.35 |  | P value summary | *** |
|  | 1 | 0.48 | 1 | 1.46 |  | Significantly different (P < 0.05)? | Yes |
|  |  |  |  |  |  | One- or two-tailed P value? | Two-tailed |
|  |  |  |  |  |  | Welch-corrected t, df | t=12.95, df=3.928 |
|  |  |  |  |  |  |  |  |
|  |  |  |  |  |  |  |  |
| **muc2** | **WT_control** | **wt_treated** | **KO_control** | **KO_D6** |  | **Unpaired t test** |  |
|  | 1 | 0.13 | 9.64 | 1.96 |  | P value | 0.0522 |
|  | 1 | 0.35 | 9.84 | 3.35 |  | P value summary | ns |
|  | 1 | 0.33 | 9.48 | 4.45 |  | Significantly different (P < 0.05)? | No |
|  |  |  |  |  |  | One- or two-tailed P value? | Two-tailed |
|  |  |  |  |  |  | Welch-corrected t, df | t=4.128, df=2.040 |
|  |  |  |  |  |  |  |  |
