# Supplementary material for: A hepatocyte-specific transcriptional program driven by Rela and Stat3 exacerbates experimental colitis in mice by modulating bile synthesis
Source: eLife. 2024 Aug 13;12:RP93273. doi: 10.7554/eLife.93273 (PMC11321761; doi:10.7554/eLife.93273)

Gel run for PCR product of CRE and internal control

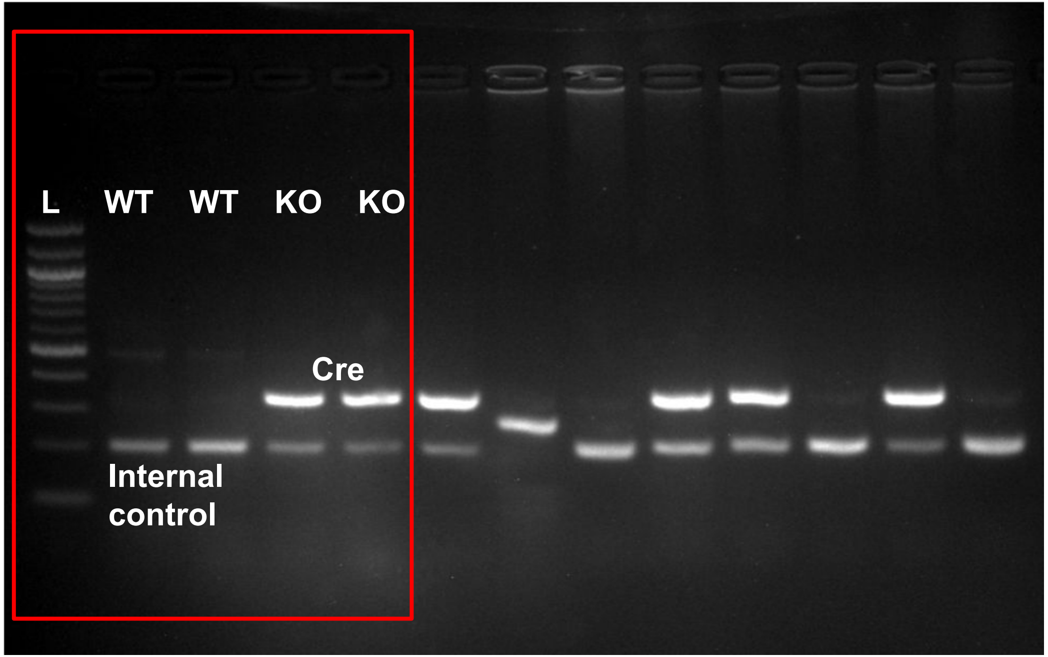

Gel run for PCR product of fRelA

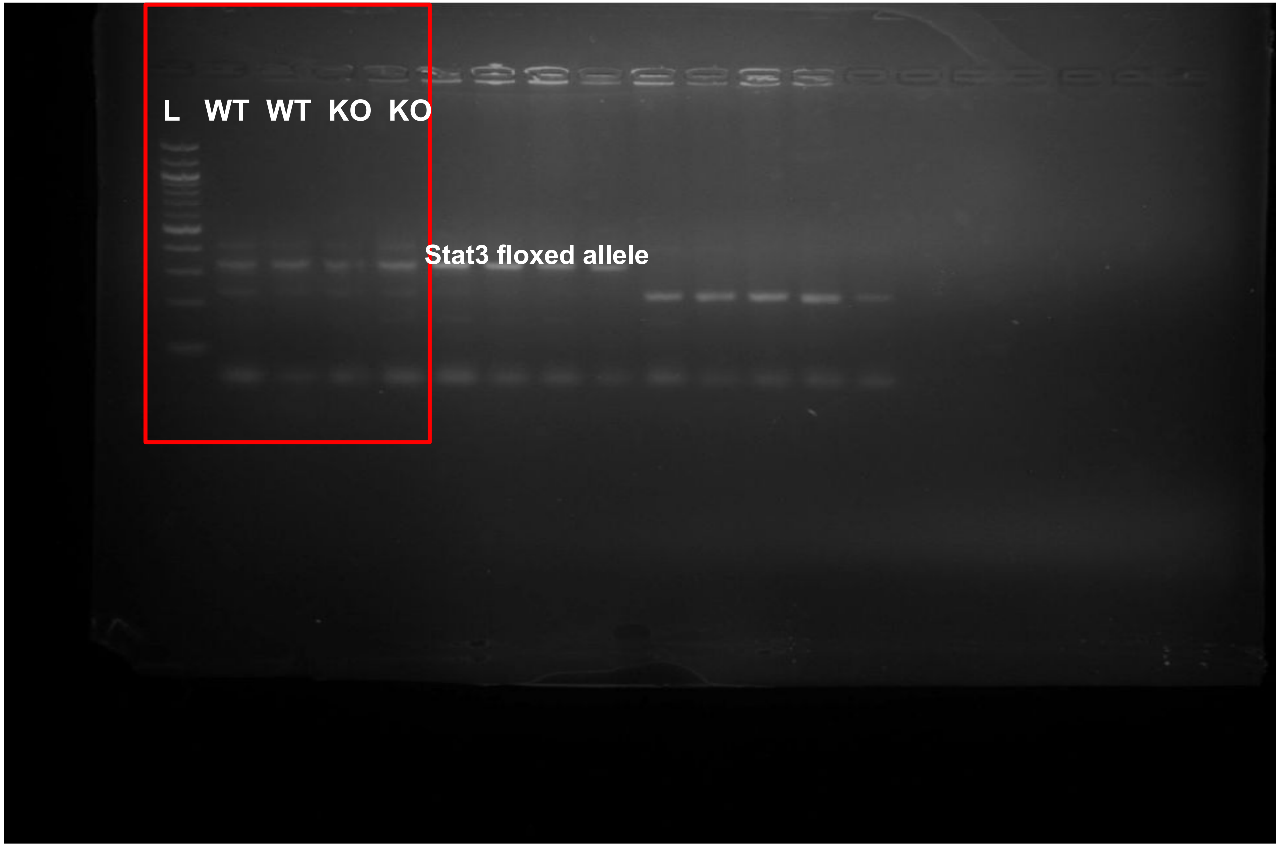

Gel run for PCR product of fRelA

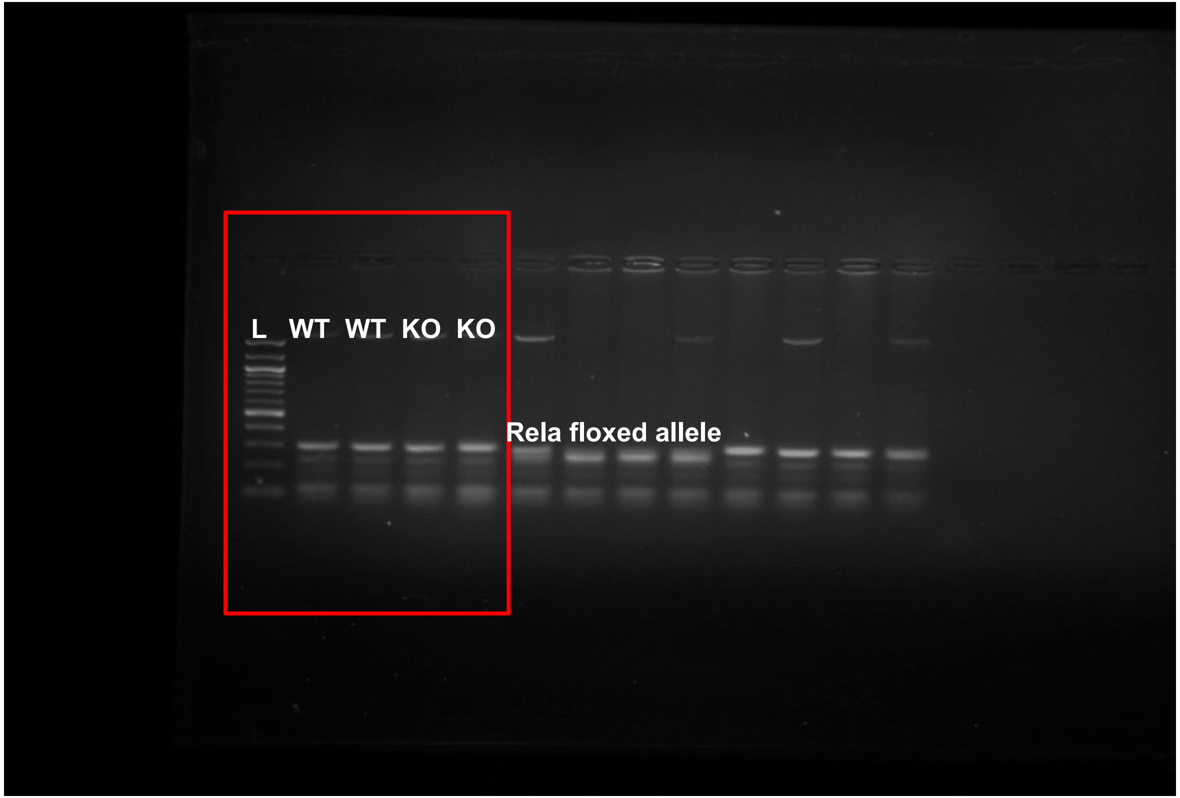

Supplement: Figure 2—figure supplement 1—source data 1. [file elife-93273-fig2-figsupp1-data1.pdf]
