# Supplementary material for: A hepatocyte-specific transcriptional program driven by Rela and Stat3 exacerbates experimental colitis in mice by modulating bile synthesis
Source: eLife. 2024 Aug 13;12:RP93273. doi: 10.7554/eLife.93273 (PMC11321761; doi:10.7554/eLife.93273)
Supplement: Figure 2—figure supplement 1—source data 3. [file elife-93273-fig2-figsupp1-data3.pdf]

Total RelA

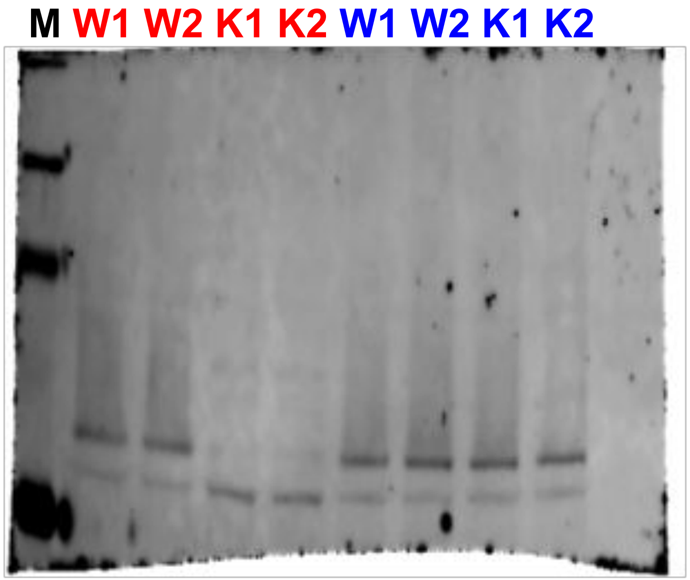

Red – liver
Blue – colon
W – wild type
K – knockout

Total Stat3

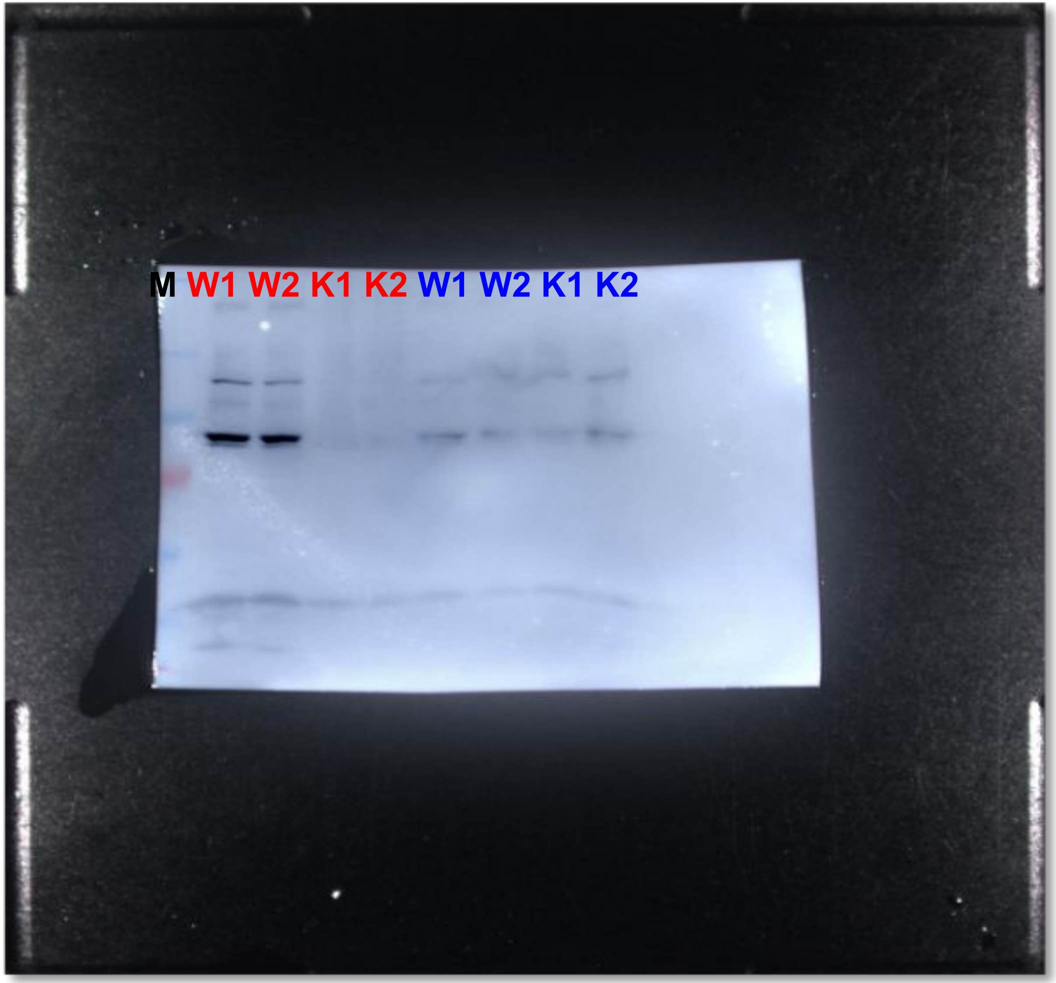

Red – liver
Blue – colon
W – wild type
K – knockout

Gapdh

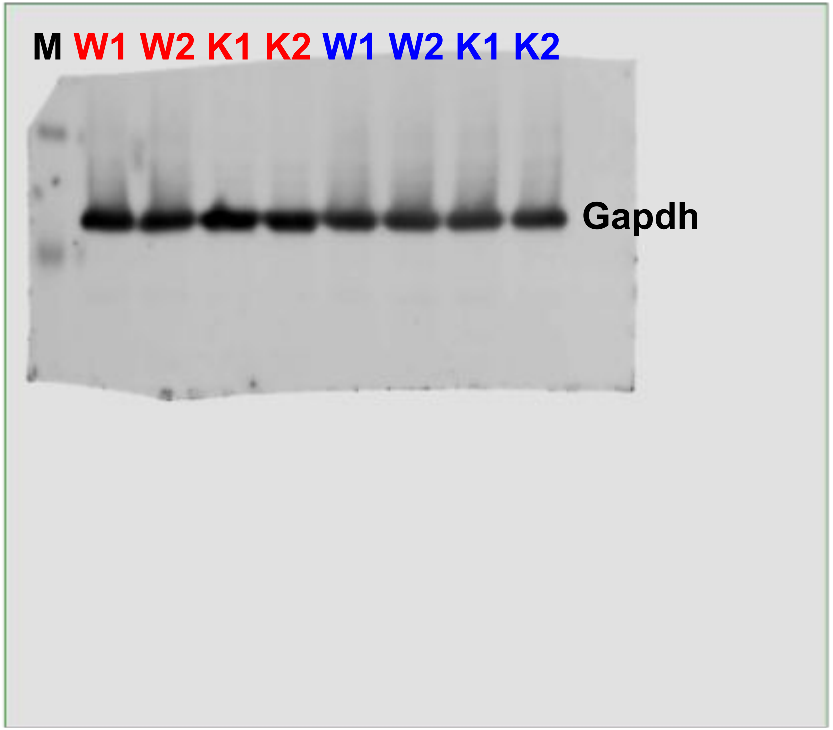

Red – liver
Blue – colon
W – wild type
K – knockout
