# Supplementary material for: A hepatocyte-specific transcriptional program driven by Rela and Stat3 exacerbates experimental colitis in mice by modulating bile synthesis
Source: eLife. 2024 Aug 13;12:RP93273. doi: 10.7554/eLife.93273 (PMC11321761; doi:10.7554/eLife.93273)
Supplement: Figure 2—figure supplement 1—source data 5. [file elife-93273-fig2-figsupp1-data5.docx]

| untreated wild type | DSS treated wild type | untreated relaΔhepstat3Δhep | untreated relaΔhepstat3Δhep |
| --- | --- | --- | --- |
| 0 | 6 | 0 | 3 |
| 0 | 4 | 0 | 2 |
| 0 | 5 | 0 | 3 |
| 0 | 5 | 0 | 3 |
| 0 | 4 | 0 | 3 |
| 0 | 5 | 0 | 4 |
| 0 | 6 | 0 | 2 |
| 0 | 6 | 0 | 3 |
| 0 | 6 | 0 | 2 |
| 0 | 6 | 0 | 3 |
| 0 | 5 | 0 | 2 |
|  |  |  |  |
