# Supplementary material for: A hepatocyte-specific transcriptional program driven by Rela and Stat3 exacerbates experimental colitis in mice by modulating bile synthesis
Source: eLife. 2024 Aug 13;12:RP93273. doi: 10.7554/eLife.93273 (PMC11321761; doi:10.7554/eLife.93273)
Supplement: Figure 3—source data 1. [file elife-93273-fig3-data1.docx]

|  | **Cluster** | **ID** | **Description** | **GeneRatio** | **BgRatio** | **pvalue** | **p.adjust** | **qvalue** |
| --- | --- | --- | --- | --- | --- | --- | --- | --- |
| **1** | WT | GO:0002831 | regulation of response to biotic stimulus | 46/530 | 337/29008 | 1.67E-26 | 7.34E-23 | 5.22E-23 |
| **2** | WT | GO:0009615 | response to virus | 44/530 | 312/29008 | 5.47E-26 | 1.20E-22 | 8.54E-23 |
| **3** | WT | GO:0051607 | defense response to virus | 40/530 | 255/29008 | 1.64E-25 | 1.80E-22 | 1.28E-22 |
| **4** | WT | GO:0140546 | defense response to symbiont | 40/530 | 255/29008 | 1.64E-25 | 1.80E-22 | 1.28E-22 |
| **5** | WT | GO:0032103 | positive regulation of response to external stimulus | 49/530 | 427/29008 | 1.01E-24 | 8.86E-22 | 6.31E-22 |
| **6** | WT | GO:0045088 | regulation of innate immune response | 35/530 | 217/29008 | 7.05E-23 | 5.16E-20 | 3.67E-20 |
| **7** | WT | GO:0002833 | positive regulation of response to biotic stimulus | 29/530 | 173/29008 | 1.20E-19 | 7.54E-17 | 5.36E-17 |
| **8** | WT | GO:0002221 | pattern recognition receptor signaling pathway | 28/530 | 171/29008 | 9.88E-19 | 5.42E-16 | 3.86E-16 |
| **9** | WT | GO:0031349 | positive regulation of defense response | 32/530 | 273/29008 | 8.36E-17 | 4.08E-14 | 2.90E-14 |
| **10** | WT | GO:1903900 | regulation of viral life cycle | 24/530 | 153/29008 | 8.37E-16 | 3.67E-13 | 2.61E-13 |
| **11** | WT | GO:0050792 | regulation of viral process | 25/530 | 174/29008 | 1.74E-15 | 6.96E-13 | 4.95E-13 |
| **12** | WT | GO:0048525 | negative regulation of viral process | 20/530 | 102/29008 | 2.50E-15 | 9.16E-13 | 6.52E-13 |
| **13** | WT | GO:0019058 | viral life cycle | 28/530 | 257/29008 | 4.69E-14 | 1.58E-11 | 1.13E-11 |
| **14** | WT | GO:0035456 | response to interferon-beta | 15/530 | 63/29008 | 3.79E-13 | 1.19E-10 | 8.45E-11 |
| **15** | WT | GO:0016032 | viral process | 31/530 | 346/29008 | 4.18E-13 | 1.22E-10 | 8.70E-11 |
| **16** | WT | GO:0045071 | negative regulation of viral genome replication | 14/530 | 58/29008 | 1.89E-12 | 5.19E-10 | 3.70E-10 |
| **17** | WT | GO:0032728 | positive regulation of interferon-beta production | 13/530 | 49/29008 | 3.15E-12 | 7.89E-10 | 5.61E-10 |
| **18** | WT | GO:0032102 | negative regulation of response to external stimulus | 32/530 | 398/29008 | 3.23E-12 | 7.89E-10 | 5.61E-10 |
| **19** | WT | GO:0045089 | positive regulation of innate immune response | 19/530 | 131/29008 | 3.64E-12 | 8.40E-10 | 5.98E-10 |
| **20** | WT | GO:0002224 | toll-like receptor signaling pathway | 18/530 | 124/29008 | 1.33E-11 | 2.92E-09 | 2.07E-09 |
| **21** | WT | GO:0019221 | cytokine-mediated signaling pathway | 31/530 | 397/29008 | 1.50E-11 | 3.14E-09 | 2.24E-09 |
| **22** | WT | GO:0032608 | interferon-beta production | 14/530 | 68/29008 | 1.95E-11 | 3.71E-09 | 2.64E-09 |
| **23** | WT | GO:0032648 | regulation of interferon-beta production | 14/530 | 68/29008 | 1.95E-11 | 3.71E-09 | 2.64E-09 |
| **24** | WT | GO:0032481 | positive regulation of type I interferon production | 14/530 | 69/29008 | 2.40E-11 | 4.39E-09 | 3.12E-09 |
| **25** | WT | GO:0062207 | regulation of pattern recognition receptor signaling pathway | 16/530 | 99/29008 | 3.45E-11 | 6.06E-09 | 4.31E-09 |
| **26** | WT | GO:0060759 | regulation of response to cytokine stimulus | 18/530 | 132/29008 | 3.88E-11 | 6.56E-09 | 4.67E-09 |
| **27** | WT | GO:0032479 | regulation of type I interferon production | 16/530 | 101/29008 | 4.72E-11 | 7.68E-09 | 5.46E-09 |
| **28** | WT | GO:0032606 | type I interferon production | 16/530 | 103/29008 | 6.41E-11 | 1.01E-08 | 7.15E-09 |
| **29** | WT | GO:0001819 | positive regulation of cytokine production | 34/530 | 500/29008 | 6.72E-11 | 1.02E-08 | 7.23E-09 |
| **30** | WT | GO:0060337 | type I interferon signaling pathway | 12/530 | 50/29008 | 7.90E-11 | 1.09E-08 | 7.73E-09 |
| **31** | WT | GO:0071357 | cellular response to type I interferon | 12/530 | 50/29008 | 7.90E-11 | 1.09E-08 | 7.73E-09 |
| **32** | WT | GO:0002697 | regulation of immune effector process | 31/530 | 424/29008 | 7.92E-11 | 1.09E-08 | 7.73E-09 |
| **33** | WT | GO:0045069 | regulation of viral genome replication | 15/530 | 90/29008 | 9.03E-11 | 1.20E-08 | 8.55E-09 |
| **34** | WT | GO:0002218 | activation of innate immune response | 12/530 | 51/29008 | 1.02E-10 | 1.31E-08 | 9.34E-09 |
| **35** | WT | GO:0035455 | response to interferon-alpha | 10/530 | 31/29008 | 1.20E-10 | 1.50E-08 | 1.07E-08 |
| **36** | WT | GO:0002683 | negative regulation of immune system process | 32/530 | 460/29008 | 1.39E-10 | 1.70E-08 | 1.21E-08 |
| **37** | WT | GO:0002237 | response to molecule of bacterial origin | 30/530 | 409/29008 | 1.49E-10 | 1.73E-08 | 1.23E-08 |
| **38** | WT | GO:0033674 | positive regulation of kinase activity | 31/530 | 435/29008 | 1.50E-10 | 1.73E-08 | 1.23E-08 |
| **39** | WT | GO:0019079 | viral genome replication | 17/530 | 126/29008 | 1.61E-10 | 1.81E-08 | 1.29E-08 |
| **40** | WT | GO:0034340 | response to type I interferon | 12/530 | 56/29008 | 3.29E-10 | 3.61E-08 | 2.57E-08 |
| **41** | WT | GO:0045860 | positive regulation of protein kinase activity | 27/530 | 358/29008 | 6.79E-10 | 7.27E-08 | 5.17E-08 |
| **42** | WT | GO:0002753 | cytoplasmic pattern recognition receptor signaling pathway | 11/530 | 50/29008 | 1.34E-09 | 1.40E-07 | 9.97E-08 |
| **43** | WT | GO:1903555 | regulation of tumor necrosis factor superfamily cytokine production | 19/530 | 186/29008 | 1.71E-09 | 1.75E-07 | 1.24E-07 |
| **44** | WT | GO:0022407 | regulation of cell-cell adhesion | 30/530 | 457/29008 | 2.07E-09 | 2.07E-07 | 1.47E-07 |
| **45** | WT | GO:0045824 | negative regulation of innate immune response | 13/530 | 81/29008 | 2.64E-09 | 2.55E-07 | 1.81E-07 |
| **46** | WT | GO:0071706 | tumor necrosis factor superfamily cytokine production | 19/530 | 191/29008 | 2.67E-09 | 2.55E-07 | 1.81E-07 |
| **47** | WT | GO:0051092 | positive regulation of NF-kappaB transcription factor activity | 17/530 | 152/29008 | 3.09E-09 | 2.88E-07 | 2.05E-07 |
| **48** | WT | GO:0050729 | positive regulation of inflammatory response | 16/530 | 134/29008 | 3.45E-09 | 3.16E-07 | 2.25E-07 |
| **49** | WT | GO:0042060 | wound healing | 26/530 | 361/29008 | 3.71E-09 | 3.32E-07 | 2.36E-07 |
| **50** | WT | GO:0032496 | response to lipopolysaccharide | 27/530 | 388/29008 | 3.89E-09 | 3.40E-07 | 2.42E-07 |
| **51** | WT | GO:0035458 | cellular response to interferon-beta | 11/530 | 55/29008 | 3.96E-09 | 3.40E-07 | 2.42E-07 |
| **52** | WT | GO:0050727 | regulation of inflammatory response | 25/530 | 342/29008 | 5.51E-09 | 4.65E-07 | 3.31E-07 |
| **53** | WT | GO:0034121 | regulation of toll-like receptor signaling pathway | 12/530 | 71/29008 | 5.89E-09 | 4.87E-07 | 3.47E-07 |
| **54** | WT | GO:0051090 | regulation of DNA-binding transcription factor activity | 28/530 | 425/29008 | 6.64E-09 | 5.40E-07 | 3.84E-07 |
| **55** | WT | GO:0062208 | positive regulation of pattern recognition receptor signaling pathway | 10/530 | 45/29008 | 6.85E-09 | 5.45E-07 | 3.87E-07 |
| **56** | WT | GO:0098586 | cellular response to virus | 12/530 | 72/29008 | 6.95E-09 | 5.45E-07 | 3.87E-07 |
| **57** | WT | GO:0001959 | regulation of cytokine-mediated signaling pathway | 15/530 | 122/29008 | 7.11E-09 | 5.47E-07 | 3.89E-07 |
| **58** | WT | GO:0032680 | regulation of tumor necrosis factor production | 18/530 | 183/29008 | 8.37E-09 | 6.33E-07 | 4.50E-07 |
| **59** | WT | GO:0009896 | positive regulation of catabolic process | 30/530 | 494/29008 | 1.23E-08 | 9.18E-07 | 6.53E-07 |
| **60** | WT | GO:0032640 | tumor necrosis factor production | 18/530 | 188/29008 | 1.28E-08 | 9.36E-07 | 6.66E-07 |
| **61** | WT | GO:0002832 | negative regulation of response to biotic stimulus | 14/530 | 115/29008 | 2.57E-08 | 1.85E-06 | 1.31E-06 |
| **62** | WT | GO:0051091 | positive regulation of DNA-binding transcription factor activity | 21/530 | 267/29008 | 2.63E-08 | 1.86E-06 | 1.32E-06 |
| **63** | WT | GO:0018108 | peptidyl-tyrosine phosphorylation | 23/530 | 322/29008 | 3.45E-08 | 2.40E-06 | 1.71E-06 |
| **64** | WT | GO:0070371 | ERK1 and ERK2 cascade | 24/530 | 349/29008 | 3.58E-08 | 2.46E-06 | 1.75E-06 |
| **65** | WT | GO:0060760 | positive regulation of response to cytokine stimulus | 10/530 | 53/29008 | 3.67E-08 | 2.48E-06 | 1.76E-06 |
| **66** | WT | GO:0018212 | peptidyl-tyrosine modification | 23/530 | 325/29008 | 4.09E-08 | 2.72E-06 | 1.94E-06 |
| **67** | WT | GO:0032675 | regulation of interleukin-6 production | 16/530 | 166/29008 | 7.48E-08 | 4.90E-06 | 3.49E-06 |
| **68** | WT | GO:0071216 | cellular response to biotic stimulus | 23/530 | 337/29008 | 7.92E-08 | 5.11E-06 | 3.64E-06 |
| **69** | WT | GO:0031348 | negative regulation of defense response | 19/530 | 236/29008 | 8.36E-08 | 5.32E-06 | 3.78E-06 |
| **70** | WT | GO:0071219 | cellular response to molecule of bacterial origin | 22/530 | 313/29008 | 9.08E-08 | 5.70E-06 | 4.05E-06 |
| **71** | WT | GO:0097191 | extrinsic apoptotic signaling pathway | 19/530 | 238/29008 | 9.55E-08 | 5.84E-06 | 4.15E-06 |
| **72** | WT | GO:0010563 | negative regulation of phosphorus metabolic process | 27/530 | 453/29008 | 9.71E-08 | 5.84E-06 | 4.15E-06 |
| **73** | WT | GO:0045936 | negative regulation of phosphate metabolic process | 27/530 | 453/29008 | 9.71E-08 | 5.84E-06 | 4.15E-06 |
| **74** | WT | GO:0031331 | positive regulation of cellular catabolic process | 26/530 | 425/29008 | 1.01E-07 | 5.99E-06 | 4.26E-06 |
| **75** | WT | GO:0007596 | blood coagulation | 16/530 | 170/29008 | 1.04E-07 | 6.10E-06 | 4.34E-06 |
| **76** | WT | GO:1903034 | regulation of response to wounding | 16/530 | 171/29008 | 1.13E-07 | 6.54E-06 | 4.65E-06 |
| **77** | WT | GO:0007599 | hemostasis | 16/530 | 173/29008 | 1.33E-07 | 7.48E-06 | 5.32E-06 |
| **78** | WT | GO:0050817 | coagulation | 16/530 | 173/29008 | 1.33E-07 | 7.48E-06 | 5.32E-06 |
| **79** | WT | GO:0032635 | interleukin-6 production | 16/530 | 174/29008 | 1.44E-07 | 8.00E-06 | 5.69E-06 |
| **80** | WT | GO:0031663 | lipopolysaccharide-mediated signaling pathway | 10/530 | 61/29008 | 1.49E-07 | 8.18E-06 | 5.82E-06 |
| **81** | WT | GO:0002699 | positive regulation of immune effector process | 21/530 | 296/29008 | 1.52E-07 | 8.25E-06 | 5.87E-06 |
| **82** | WT | GO:0002730 | regulation of dendritic cell cytokine production | 6/530 | 15/29008 | 1.57E-07 | 8.32E-06 | 5.92E-06 |
| **83** | WT | GO:0060700 | regulation of ribonuclease activity | 6/530 | 15/29008 | 1.57E-07 | 8.32E-06 | 5.92E-06 |
| **84** | WT | GO:0070372 | regulation of ERK1 and ERK2 cascade | 22/530 | 326/29008 | 1.84E-07 | 9.62E-06 | 6.85E-06 |
| **85** | WT | GO:0071222 | cellular response to lipopolysaccharide | 21/530 | 303/29008 | 2.25E-07 | 1.16E-05 | 8.23E-06 |
| **86** | WT | GO:0060338 | regulation of type I interferon-mediated signaling pathway | 8/530 | 36/29008 | 2.27E-07 | 1.16E-05 | 8.23E-06 |
| **87** | WT | GO:0070374 | positive regulation of ERK1 and ERK2 cascade | 18/530 | 227/29008 | 2.29E-07 | 1.16E-05 | 8.23E-06 |
| **88** | WT | GO:0002371 | dendritic cell cytokine production | 6/530 | 16/29008 | 2.48E-07 | 1.24E-05 | 8.80E-06 |
| **89** | WT | GO:0043903 | regulation of biological process involved in symbiotic interaction | 11/530 | 81/29008 | 2.61E-07 | 1.29E-05 | 9.17E-06 |
| **90** | WT | GO:0009117 | nucleotide metabolic process | 26/530 | 451/29008 | 3.19E-07 | 1.54E-05 | 1.10E-05 |
| **91** | WT | GO:0030193 | regulation of blood coagulation | 10/530 | 66/29008 | 3.21E-07 | 1.54E-05 | 1.10E-05 |
| **92** | WT | GO:0046596 | regulation of viral entry into host cell | 9/530 | 51/29008 | 3.26E-07 | 1.54E-05 | 1.10E-05 |
| **93** | WT | GO:0070527 | platelet aggregation | 9/530 | 51/29008 | 3.26E-07 | 1.54E-05 | 1.10E-05 |
| **94** | WT | GO:0050878 | regulation of body fluid levels | 23/530 | 366/29008 | 3.45E-07 | 1.61E-05 | 1.15E-05 |
| **95** | WT | GO:0046718 | viral entry into host cell | 12/530 | 102/29008 | 3.72E-07 | 1.72E-05 | 1.22E-05 |
| **96** | WT | GO:0022409 | positive regulation of cell-cell adhesion | 20/530 | 288/29008 | 4.22E-07 | 1.89E-05 | 1.35E-05 |
| **97** | WT | GO:0031664 | regulation of lipopolysaccharide-mediated signaling pathway | 7/530 | 27/29008 | 4.23E-07 | 1.89E-05 | 1.35E-05 |
| **98** | WT | GO:0032069 | regulation of nuclease activity | 7/530 | 27/29008 | 4.23E-07 | 1.89E-05 | 1.35E-05 |
| **99** | WT | GO:1900046 | regulation of hemostasis | 10/530 | 68/29008 | 4.29E-07 | 1.90E-05 | 1.35E-05 |
| **100** | WT | GO:0045907 | positive regulation of vasoconstriction | 9/530 | 53/29008 | 4.60E-07 | 2.02E-05 | 1.44E-05 |
| **101** | WT | GO:0006753 | nucleoside phosphate metabolic process | 26/530 | 460/29008 | 4.64E-07 | 2.02E-05 | 1.44E-05 |
| **102** | WT | GO:0001818 | negative regulation of cytokine production | 20/530 | 292/29008 | 5.25E-07 | 2.26E-05 | 1.61E-05 |
| **103** | WT | GO:0050818 | regulation of coagulation | 10/530 | 70/29008 | 5.66E-07 | 2.38E-05 | 1.69E-05 |
| **104** | WT | GO:0044409 | entry into host | 12/530 | 106/29008 | 5.67E-07 | 2.38E-05 | 1.69E-05 |
| **105** | WT | GO:0052126 | movement in host environment | 13/530 | 126/29008 | 5.69E-07 | 2.38E-05 | 1.69E-05 |
| **106** | WT | GO:0051701 | biological process involved in interaction with host | 14/530 | 149/29008 | 6.65E-07 | 2.75E-05 | 1.96E-05 |
| **107** | WT | GO:0002703 | regulation of leukocyte mediated immunity | 20/530 | 297/29008 | 6.85E-07 | 2.81E-05 | 2.00E-05 |
| **108** | WT | GO:0045785 | positive regulation of cell adhesion | 26/530 | 471/29008 | 7.25E-07 | 2.95E-05 | 2.10E-05 |
| **109** | WT | GO:0052372 | modulation by symbiont of entry into host | 9/530 | 56/29008 | 7.49E-07 | 3.01E-05 | 2.14E-05 |
| **110** | WT | GO:1901652 | response to peptide | 24/530 | 412/29008 | 7.53E-07 | 3.01E-05 | 2.14E-05 |
| **111** | WT | GO:0042730 | fibrinolysis | 6/530 | 19/29008 | 8.02E-07 | 3.17E-05 | 2.26E-05 |
| **112** | WT | GO:0061041 | regulation of wound healing | 13/530 | 130/29008 | 8.16E-07 | 3.20E-05 | 2.28E-05 |
| **113** | WT | GO:0032760 | positive regulation of tumor necrosis factor production | 12/530 | 110/29008 | 8.49E-07 | 3.30E-05 | 2.35E-05 |
| **114** | WT | GO:0050866 | negative regulation of cell activation | 17/530 | 223/29008 | 8.59E-07 | 3.31E-05 | 2.35E-05 |
| **115** | WT | GO:0039528 | cytoplasmic pattern recognition receptor signaling pathway in response to virus | 7/530 | 30/29008 | 9.24E-07 | 3.53E-05 | 2.51E-05 |
| **116** | WT | GO:1903557 | positive regulation of tumor necrosis factor superfamily cytokine production | 12/530 | 112/29008 | 1.03E-06 | 3.90E-05 | 2.78E-05 |
| **117** | WT | GO:0010508 | positive regulation of autophagy | 13/530 | 133/29008 | 1.06E-06 | 3.98E-05 | 2.83E-05 |
| **118** | WT | GO:0042326 | negative regulation of phosphorylation | 23/530 | 396/29008 | 1.34E-06 | 5.00E-05 | 3.56E-05 |
| **119** | WT | GO:0034341 | response to interferon-gamma | 13/530 | 139/29008 | 1.75E-06 | 6.46E-05 | 4.59E-05 |
| **120** | WT | GO:0016126 | sterol biosynthetic process | 9/530 | 62/29008 | 1.82E-06 | 6.66E-05 | 4.74E-05 |
| **121** | WT | GO:0032611 | interleukin-1 beta production | 11/530 | 99/29008 | 2.02E-06 | 7.33E-05 | 5.22E-05 |
| **122** | WT | GO:1904019 | epithelial cell apoptotic process | 12/530 | 120/29008 | 2.16E-06 | 7.70E-05 | 5.48E-05 |
| **123** | WT | GO:0010038 | response to metal ion | 19/530 | 292/29008 | 2.16E-06 | 7.70E-05 | 5.48E-05 |
| **124** | WT | GO:0044403 | biological process involved in symbiotic interaction | 18/530 | 265/29008 | 2.18E-06 | 7.70E-05 | 5.48E-05 |
| **125** | WT | GO:0050730 | regulation of peptidyl-tyrosine phosphorylation | 18/530 | 266/29008 | 2.30E-06 | 8.06E-05 | 5.74E-05 |
| **126** | WT | GO:0030195 | negative regulation of blood coagulation | 8/530 | 48/29008 | 2.34E-06 | 8.15E-05 | 5.80E-05 |
| **127** | WT | GO:0032615 | interleukin-12 production | 9/530 | 65/29008 | 2.73E-06 | 9.43E-05 | 6.71E-05 |
| **128** | WT | GO:1900047 | negative regulation of hemostasis | 8/530 | 49/29008 | 2.75E-06 | 9.43E-05 | 6.71E-05 |
| **129** | WT | GO:0006694 | steroid biosynthetic process | 14/530 | 168/29008 | 2.80E-06 | 9.48E-05 | 6.74E-05 |
| **130** | WT | GO:0032722 | positive regulation of chemokine production | 10/530 | 83/29008 | 2.81E-06 | 9.48E-05 | 6.74E-05 |
| **131** | WT | GO:0050819 | negative regulation of coagulation | 8/530 | 50/29008 | 3.22E-06 | 0.0001079912353 | 7.68E-05 |
| **132** | WT | GO:0043254 | regulation of protein-containing complex assembly | 24/530 | 451/29008 | 3.63E-06 | 0.0001207003787 | 8.59E-05 |
| **133** | WT | GO:0019229 | regulation of vasoconstriction | 10/530 | 86/29008 | 3.89E-06 | 0.0001274942984 | 9.07E-05 |
| **134** | WT | GO:0030168 | platelet activation | 10/530 | 86/29008 | 3.89E-06 | 0.0001274942984 | 9.07E-05 |
| **135** | WT | GO:0034138 | toll-like receptor 3 signaling pathway | 6/530 | 25/29008 | 4.77E-06 | 0.0001550077999 | 0.0001102764997 |
| **136** | WT | GO:0002700 | regulation of production of molecular mediator of immune response | 14/530 | 177/29008 | 5.15E-06 | 0.0001663604284 | 0.000118353049 |
| **137** | WT | GO:0032755 | positive regulation of interleukin-6 production | 11/530 | 109/29008 | 5.22E-06 | 0.00016676446 | 0.0001186404874 |
| **138** | WT | GO:0007249 | I-kappaB kinase/NF-kappaB signaling | 16/530 | 228/29008 | 5.24E-06 | 0.00016676446 | 0.0001186404874 |
| **139** | WT | GO:0031639 | plasminogen activation | 6/530 | 26/29008 | 6.10E-06 | 0.0001913216341 | 0.000136111087 |
| **140** | WT | GO:0039529 | RIG-I signaling pathway | 6/530 | 26/29008 | 6.10E-06 | 0.0001913216341 | 0.000136111087 |
| **141** | WT | GO:0010506 | regulation of autophagy | 17/530 | 259/29008 | 6.55E-06 | 0.0002036116013 | 0.000144854483 |
| **142** | WT | GO:0050777 | negative regulation of immune response | 15/530 | 206/29008 | 6.59E-06 | 0.0002036116013 | 0.000144854483 |
| **143** | WT | GO:2001235 | positive regulation of apoptotic signaling pathway | 13/530 | 157/29008 | 6.78E-06 | 0.0002073447222 | 0.0001475103203 |
| **144** | WT | GO:0071346 | cellular response to interferon-gamma | 11/530 | 112/29008 | 6.80E-06 | 0.0002073447222 | 0.0001475103203 |
| **145** | WT | GO:0048638 | regulation of developmental growth | 22/530 | 406/29008 | 6.91E-06 | 0.0002092685069 | 0.0001488789498 |
| **146** | WT | GO:1904035 | regulation of epithelial cell apoptotic process | 10/530 | 92/29008 | 7.19E-06 | 0.0002150024684 | 0.0001529582361 |
| **147** | WT | GO:0002274 | myeloid leukocyte activation | 17/530 | 261/29008 | 7.25E-06 | 0.0002150024684 | 0.0001529582361 |
| **148** | WT | GO:0070663 | regulation of leukocyte proliferation | 17/530 | 261/29008 | 7.25E-06 | 0.0002150024684 | 0.0001529582361 |
| **149** | WT | GO:0002253 | activation of immune response | 23/530 | 439/29008 | 7.44E-06 | 0.0002177098067 | 0.0001548843056 |
| **150** | WT | GO:0034329 | cell junction assembly | 23/530 | 439/29008 | 7.44E-06 | 0.0002177098067 | 0.0001548843056 |
| **151** | WT | GO:0032651 | regulation of interleukin-1 beta production | 10/530 | 93/29008 | 7.92E-06 | 0.0002298638391 | 0.0001635309941 |
| **152** | WT | GO:0001933 | negative regulation of protein phosphorylation | 20/530 | 349/29008 | 7.96E-06 | 0.0002298638391 | 0.0001635309941 |
| **153** | WT | GO:0043434 | response to peptide hormone | 20/530 | 351/29008 | 8.66E-06 | 0.0002483525478 | 0.0001766843327 |
| **154** | WT | GO:0002366 | leukocyte activation involved in immune response | 19/530 | 324/29008 | 9.66E-06 | 0.0002753808563 | 0.0001959129602 |
| **155** | WT | GO:0051259 | protein complex oligomerization | 16/530 | 240/29008 | 1.00E-05 | 0.0002838667952 | 0.0002019500735 |
| **156** | WT | GO:0002263 | cell activation involved in immune response | 19/530 | 328/29008 | 1.15E-05 | 0.0003211003353 | 0.0002284389629 |
| **157** | WT | GO:0070661 | leukocyte proliferation | 20/530 | 358/29008 | 1.15E-05 | 0.0003211003353 | 0.0002284389629 |
| **158** | WT | GO:0002718 | regulation of cytokine production involved in immune response | 10/530 | 97/29008 | 1.16E-05 | 0.0003211003353 | 0.0002284389629 |
| **159** | WT | GO:0030522 | intracellular receptor signaling pathway | 15/530 | 217/29008 | 1.23E-05 | 0.0003395393211 | 0.0002415569274 |
| **160** | WT | GO:0034109 | homotypic cell-cell adhesion | 9/530 | 78/29008 | 1.26E-05 | 0.0003466668383 | 0.0002466276248 |
| **161** | WT | GO:0032612 | interleukin-1 production | 11/530 | 120/29008 | 1.32E-05 | 0.0003598291969 | 0.0002559916622 |
| **162** | WT | GO:0030099 | myeloid cell differentiation | 22/530 | 426/29008 | 1.46E-05 | 0.0003958911385 | 0.0002816470466 |
| **163** | WT | GO:0034123 | positive regulation of toll-like receptor signaling pathway | 6/530 | 30/29008 | 1.48E-05 | 0.0003958911385 | 0.0002816470466 |
| **164** | WT | GO:0046597 | negative regulation of viral entry into host cell | 6/530 | 30/29008 | 1.48E-05 | 0.0003958911385 | 0.0002816470466 |
| **165** | WT | GO:0009259 | ribonucleotide metabolic process | 20/530 | 367/29008 | 1.65E-05 | 0.0004399763289 | 0.0003130103747 |
| **166** | WT | GO:0008202 | steroid metabolic process | 19/530 | 337/29008 | 1.68E-05 | 0.0004430969467 | 0.0003152304618 |
| **167** | WT | GO:0035296 | regulation of tube diameter | 13/530 | 171/29008 | 1.70E-05 | 0.0004451904498 | 0.0003167198332 |
| **168** | WT | GO:0097746 | blood vessel diameter maintenance | 13/530 | 171/29008 | 1.70E-05 | 0.0004451904498 | 0.0003167198332 |
| **169** | WT | GO:2000352 | negative regulation of endothelial cell apoptotic process | 6/530 | 31/29008 | 1.81E-05 | 0.0004681318794 | 0.000333040951 |
| **170** | WT | GO:0035150 | regulation of tube size | 13/530 | 172/29008 | 1.81E-05 | 0.0004681318794 | 0.000333040951 |
| **171** | WT | GO:0035457 | cellular response to interferon-alpha | 5/530 | 19/29008 | 1.88E-05 | 0.0004820001225 | 0.0003429071726 |
| **172** | WT | GO:0032655 | regulation of interleukin-12 production | 8/530 | 63/29008 | 1.89E-05 | 0.0004820001225 | 0.0003429071726 |
| **173** | WT | GO:2001233 | regulation of apoptotic signaling pathway | 21/530 | 402/29008 | 1.92E-05 | 0.0004879409222 | 0.000347133609 |
| **174** | WT | GO:0002695 | negative regulation of leukocyte activation | 14/530 | 200/29008 | 2.07E-05 | 0.0005226532765 | 0.0003718288626 |
| **175** | WT | GO:0007159 | leukocyte cell-cell adhesion | 20/530 | 373/29008 | 2.09E-05 | 0.0005232978998 | 0.000372287464 |
| **176** | WT | GO:0001961 | positive regulation of cytokine-mediated signaling pathway | 7/530 | 47/29008 | 2.18E-05 | 0.000544312515 | 0.0003872377969 |
| **177** | WT | GO:0050920 | regulation of chemotaxis | 15/530 | 229/29008 | 2.32E-05 | 0.0005752485125 | 0.0004092464541 |
| **178** | WT | GO:0042742 | defense response to bacterium | 23/530 | 473/29008 | 2.44E-05 | 0.0006005980133 | 0.0004272807351 |
| **179** | WT | GO:0051348 | negative regulation of transferase activity | 16/530 | 259/29008 | 2.57E-05 | 0.0006297058799 | 0.0004479888132 |
| **180** | WT | GO:0019693 | ribose phosphate metabolic process | 20/530 | 379/29008 | 2.62E-05 | 0.0006352144935 | 0.0004519077814 |
| **181** | WT | GO:0031099 | regeneration | 12/530 | 153/29008 | 2.62E-05 | 0.0006352144935 | 0.0004519077814 |
| **182** | WT | GO:1903901 | negative regulation of viral life cycle | 6/530 | 33/29008 | 2.63E-05 | 0.0006352144935 | 0.0004519077814 |
| **183** | WT | GO:0090407 | organophosphate biosynthetic process | 23/530 | 476/29008 | 2.69E-05 | 0.0006445925528 | 0.0004585795718 |
| **184** | WT | GO:0016125 | sterol metabolic process | 12/530 | 154/29008 | 2.80E-05 | 0.0006676684631 | 0.0004749963625 |
| **185** | WT | GO:0002460 | adaptive immune response based on somatic recombination of immune receptors built from immunoglobulin superfamily domains | 23/530 | 480/29008 | 3.06E-05 | 0.0007259351222 | 0.0005164487489 |
| **186** | WT | GO:1901214 | regulation of neuron death | 20/530 | 384/29008 | 3.15E-05 | 0.0007432564572 | 0.0005287715882 |
| **187** | WT | GO:0042310 | vasoconstriction | 10/530 | 109/29008 | 3.22E-05 | 0.0007568040065 | 0.0005384096601 |
| **188** | WT | GO:0016055 | Wnt signaling pathway | 22/530 | 449/29008 | 3.25E-05 | 0.0007586761545 | 0.0005397415539 |
| **189** | WT | GO:0007219 | Notch signaling pathway | 13/530 | 182/29008 | 3.29E-05 | 0.0007640410191 | 0.0005435582553 |
| **190** | WT | GO:0198738 | cell-cell signaling by wnt | 22/530 | 451/29008 | 3.47E-05 | 0.0007936172041 | 0.0005645995071 |
| **191** | WT | GO:0002705 | positive regulation of leukocyte mediated immunity | 13/530 | 183/29008 | 3.48E-05 | 0.0007936172041 | 0.0005645995071 |
| **192** | WT | GO:0002526 | acute inflammatory response | 10/530 | 110/29008 | 3.49E-05 | 0.0007936172041 | 0.0005645995071 |
| **193** | WT | GO:0032642 | regulation of chemokine production | 10/530 | 110/29008 | 3.49E-05 | 0.0007936172041 | 0.0005645995071 |
| **194** | WT | GO:0002367 | cytokine production involved in immune response | 10/530 | 111/29008 | 3.77E-05 | 0.0008537288778 | 0.000607364484 |
| **195** | WT | GO:0001822 | kidney development | 18/530 | 327/29008 | 3.80E-05 | 0.0008557789603 | 0.0006088229649 |
| **196** | WT | GO:0051770 | positive regulation of nitric-oxide synthase biosynthetic process | 5/530 | 22/29008 | 4.07E-05 | 0.0009111815154 | 0.0006482377548 |
| **197** | WT | GO:1904036 | negative regulation of epithelial cell apoptotic process | 7/530 | 52/29008 | 4.29E-05 | 0.000956138905 | 0.0006802215876 |
| **198** | WT | GO:1903035 | negative regulation of response to wounding | 9/530 | 91/29008 | 4.40E-05 | 0.0009753365834 | 0.0006938793053 |
| **199** | WT | GO:0034122 | negative regulation of toll-like receptor signaling pathway | 6/530 | 36/29008 | 4.42E-05 | 0.0009753365834 | 0.0006938793053 |
| **200** | WT | GO:0032602 | chemokine production | 10/530 | 114/29008 | 4.75E-05 | 0.001036376774 | 0.0007373048525 |
| **201** | WT | GO:0032652 | regulation of interleukin-1 production | 10/530 | 114/29008 | 4.75E-05 | 0.001036376774 | 0.0007373048525 |
| **202** | WT | GO:1903037 | regulation of leukocyte cell-cell adhesion | 18/530 | 333/29008 | 4.81E-05 | 0.001045749531 | 0.0007439728711 |
| **203** | WT | GO:2000351 | regulation of endothelial cell apoptotic process | 7/530 | 53/29008 | 4.87E-05 | 0.001052390766 | 0.0007486976145 |
| **204** | WT | GO:0032943 | mononuclear cell proliferation | 18/530 | 334/29008 | 5.00E-05 | 0.001076344377 | 0.0007657388244 |
| **205** | WT | GO:0032944 | regulation of mononuclear cell proliferation | 15/530 | 245/29008 | 5.05E-05 | 0.001077450463 | 0.0007665257223 |
| **206** | WT | GO:0061045 | negative regulation of wound healing | 8/530 | 72/29008 | 5.06E-05 | 0.001077450463 | 0.0007665257223 |
| **207** | WT | GO:0050764 | regulation of phagocytosis | 10/530 | 115/29008 | 5.11E-05 | 0.001079520826 | 0.0007679986307 |
| **208** | WT | GO:1901623 | regulation of lymphocyte chemotaxis | 5/530 | 23/29008 | 5.12E-05 | 0.001079520826 | 0.0007679986307 |
| **209** | WT | GO:0043122 | regulation of I-kappaB kinase/NF-kappaB signaling | 13/530 | 190/29008 | 5.14E-05 | 0.001079520826 | 0.0007679986307 |
| **210** | WT | GO:0006986 | response to unfolded protein | 10/530 | 116/29008 | 5.51E-05 | 0.001145525746 | 0.0008149562135 |
| **211** | WT | GO:0038061 | NIK/NF-kappaB signaling | 10/530 | 116/29008 | 5.51E-05 | 0.001145525746 | 0.0008149562135 |
| **212** | WT | GO:0050731 | positive regulation of peptidyl-tyrosine phosphorylation | 13/530 | 192/29008 | 5.72E-05 | 0.001185197406 | 0.0008431796433 |
| **213** | WT | GO:0019932 | second-messenger-mediated signaling | 17/530 | 308/29008 | 6.00E-05 | 0.001235922916 | 0.0008792670644 |
| **214** | WT | GO:0050863 | regulation of T cell activation | 18/530 | 339/29008 | 6.05E-05 | 0.001241899708 | 0.0008835191065 |
| **215** | WT | GO:0071248 | cellular response to metal ion | 12/530 | 167/29008 | 6.19E-05 | 0.001264279901 | 0.0008994409459 |
| **216** | WT | GO:0032727 | positive regulation of interferon-alpha production | 5/530 | 24/29008 | 6.37E-05 | 0.001288871926 | 0.0009169363399 |
| **217** | WT | GO:0060339 | negative regulation of type I interferon-mediated signaling pathway | 5/530 | 24/29008 | 6.37E-05 | 0.001288871926 | 0.0009169363399 |
| **218** | WT | GO:0051250 | negative regulation of lymphocyte activation | 12/530 | 168/29008 | 6.56E-05 | 0.001314920551 | 0.0009354679955 |
| **219** | WT | GO:2001236 | regulation of extrinsic apoptotic signaling pathway | 12/530 | 168/29008 | 6.56E-05 | 0.001314920551 | 0.0009354679955 |
| **220** | WT | GO:1901222 | regulation of NIK/NF-kappaB signaling | 9/530 | 96/29008 | 6.72E-05 | 0.001339800841 | 0.0009531684679 |
| **221** | WT | GO:0030198 | extracellular matrix organization | 17/530 | 311/29008 | 6.76E-05 | 0.001339800841 | 0.0009531684679 |
| **222** | WT | GO:0072001 | renal system development | 18/530 | 342/29008 | 6.78E-05 | 0.001339800841 | 0.0009531684679 |
| **223** | WT | GO:0072376 | protein activation cascade | 4/530 | 13/29008 | 6.91E-05 | 0.001356713744 | 0.000965200738 |
| **224** | WT | GO:0006695 | cholesterol biosynthetic process | 7/530 | 56/29008 | 6.98E-05 | 0.001356713744 | 0.000965200738 |
| **225** | WT | GO:0072577 | endothelial cell apoptotic process | 7/530 | 56/29008 | 6.98E-05 | 0.001356713744 | 0.000965200738 |
| **226** | WT | GO:1902653 | secondary alcohol biosynthetic process | 7/530 | 56/29008 | 6.98E-05 | 0.001356713744 | 0.000965200738 |
| **227** | WT | GO:0043062 | extracellular structure organization | 17/530 | 312/29008 | 7.03E-05 | 0.001358947124 | 0.0009667896217 |
| **228** | WT | GO:0070555 | response to interleukin-1 | 9/530 | 97/29008 | 7.29E-05 | 0.001400900093 | 0.0009966360329 |
| **229** | WT | GO:0045229 | external encapsulating structure organization | 17/530 | 313/29008 | 7.31E-05 | 0.001400900093 | 0.0009966360329 |
| **230** | WT | GO:0007160 | cell-matrix adhesion | 14/530 | 225/29008 | 7.51E-05 | 0.00143369026 | 0.001019963793 |
| **231** | WT | GO:0008203 | cholesterol metabolic process | 11/530 | 145/29008 | 7.65E-05 | 0.001453042741 | 0.001033731642 |
| **232** | WT | GO:0034114 | regulation of heterotypic cell-cell adhesion | 5/530 | 25/29008 | 7.84E-05 | 0.001482827433 | 0.001054921231 |
| **233** | WT | GO:0033673 | negative regulation of kinase activity | 14/530 | 226/29008 | 7.88E-05 | 0.001482827433 | 0.001054921231 |
| **234** | WT | GO:0048732 | gland development | 22/530 | 477/29008 | 7.92E-05 | 0.001482827433 | 0.001054921231 |
| **235** | WT | GO:1901215 | negative regulation of neuron death | 15/530 | 255/29008 | 7.94E-05 | 0.001482827433 | 0.001054921231 |
| **236** | WT | GO:0030111 | regulation of Wnt signaling pathway | 17/530 | 317/29008 | 8.53E-05 | 0.001580398002 | 0.001124335421 |
| **237** | WT | GO:0034504 | protein localization to nucleus | 17/530 | 317/29008 | 8.53E-05 | 0.001580398002 | 0.001124335421 |
| **238** | WT | GO:1901136 | carbohydrate derivative catabolic process | 11/530 | 147/29008 | 8.65E-05 | 0.001595685477 | 0.00113521132 |
| **239** | WT | GO:0071900 | regulation of protein serine/threonine kinase activity | 18/530 | 349/29008 | 8.76E-05 | 0.001609347725 | 0.001144930992 |
| **240** | WT | GO:1901617 | organic hydroxy compound biosynthetic process | 14/530 | 230/29008 | 9.49E-05 | 0.001721277278 | 0.001224560528 |
| **241** | WT | GO:0034116 | positive regulation of heterotypic cell-cell adhesion | 4/530 | 14/29008 | 9.54E-05 | 0.001721277278 | 0.001224560528 |
| **242** | WT | GO:0051988 | regulation of attachment of spindle microtubules to kinetochore | 4/530 | 14/29008 | 9.54E-05 | 0.001721277278 | 0.001224560528 |
| **243** | WT | GO:0072683 | T cell extravasation | 4/530 | 14/29008 | 9.54E-05 | 0.001721277278 | 0.001224560528 |
| **244** | WT | GO:0045780 | positive regulation of bone resorption | 5/530 | 26/29008 | 9.57E-05 | 0.001721277278 | 0.001224560528 |
| **245** | WT | GO:0001655 | urogenital system development | 19/530 | 385/29008 | 0.0001005501545 | 0.001799039462 | 0.001279882528 |
| **246** | WT | GO:0006469 | negative regulation of protein kinase activity | 13/530 | 203/29008 | 0.0001008117785 | 0.001799039462 | 0.001279882528 |
| **247** | WT | GO:0002702 | positive regulation of production of molecular mediator of immune response | 10/530 | 125/29008 | 0.0001034697095 | 0.001838996051 | 0.001308308664 |
| **248** | WT | GO:0071347 | cellular response to interleukin-1 | 8/530 | 80/29008 | 0.0001078067961 | 0.001908354173 | 0.0013576518 |
| **249** | WT | GO:0019882 | antigen processing and presentation | 10/530 | 126/29008 | 0.0001105843247 | 0.001941860742 | 0.001381489227 |
| **250** | WT | GO:0043123 | positive regulation of I-kappaB kinase/NF-kappaB signaling | 10/530 | 126/29008 | 0.0001105843247 | 0.001941860742 | 0.001381489227 |
| **251** | WT | GO:0042176 | regulation of protein catabolic process | 19/530 | 389/29008 | 0.000114993285 | 0.002011237137 | 0.001430845363 |
| **252** | WT | GO:0022408 | negative regulation of cell-cell adhesion | 13/530 | 206/29008 | 0.0001167967723 | 0.00203467393 | 0.001447518894 |
| **253** | WT | GO:0043087 | regulation of GTPase activity | 17/530 | 326/29008 | 0.0001196628009 | 0.002076362434 | 0.001477177159 |
| **254** | WT | GO:0032735 | positive regulation of interleukin-12 production | 6/530 | 43/29008 | 0.0001242009726 | 0.002145124264 | 0.001526096078 |
| **255** | WT | GO:0002819 | regulation of adaptive immune response | 14/530 | 236/29008 | 0.0001246028901 | 0.002145124264 | 0.001526096078 |
| **256** | WT | GO:0008625 | extrinsic apoptotic signaling pathway via death domain receptors | 8/530 | 82/29008 | 0.0001284041086 | 0.002201929831 | 0.001566509006 |
| **257** | WT | GO:0043547 | positive regulation of GTPase activity | 14/530 | 237/29008 | 0.000130256809 | 0.002225009306 | 0.001582928333 |
| **258** | WT | GO:0001960 | negative regulation of cytokine-mediated signaling pathway | 7/530 | 62/29008 | 0.0001347913186 | 0.002293542204 | 0.001631684383 |
| **259** | WT | GO:0051767 | nitric-oxide synthase biosynthetic process | 5/530 | 28/29008 | 0.0001386967039 | 0.002329169607 | 0.001657030626 |
| **260** | WT | GO:0051769 | regulation of nitric-oxide synthase biosynthetic process | 5/530 | 28/29008 | 0.0001386967039 | 0.002329169607 | 0.001657030626 |
| **261** | WT | GO:0006091 | generation of precursor metabolites and energy | 20/530 | 428/29008 | 0.0001390073889 | 0.002329169607 | 0.001657030626 |
| **262** | WT | GO:0070997 | neuron death | 20/530 | 428/29008 | 0.0001390073889 | 0.002329169607 | 0.001657030626 |
| **263** | WT | GO:0072521 | purine-containing compound metabolic process | 19/530 | 395/29008 | 0.0001400904252 | 0.002338391507 | 0.00166359132 |
| **264** | WT | GO:0061098 | positive regulation of protein tyrosine kinase activity | 6/530 | 44/29008 | 0.000141610561 | 0.00235481198 | 0.001675273263 |
| **265** | WT | GO:0051592 | response to calcium ion | 10/530 | 130/29008 | 0.0001433264682 | 0.002374351681 | 0.001689174305 |
| **266** | WT | GO:0008643 | carbohydrate transport | 11/530 | 156/29008 | 0.0001469202945 | 0.00242473719 | 0.00172501984 |
| **267** | WT | GO:0008360 | regulation of cell shape | 11/530 | 157/29008 | 0.0001554378304 | 0.002543067557 | 0.001809203079 |
| **268** | WT | GO:1902652 | secondary alcohol metabolic process | 11/530 | 157/29008 | 0.0001554378304 | 0.002543067557 | 0.001809203079 |
| **269** | WT | GO:0043524 | negative regulation of neuron apoptotic process | 12/530 | 184/29008 | 0.0001558280576 | 0.002543067557 | 0.001809203079 |
| **270** | WT | GO:0042554 | superoxide anion generation | 6/530 | 45/29008 | 0.0001608968036 | 0.002616062843 | 0.001861133786 |
| **271** | WT | GO:0032647 | regulation of interferon-alpha production | 5/530 | 29/29008 | 0.0001650884306 | 0.00267431074 | 0.001902572825 |
| **272** | WT | GO:0034162 | toll-like receptor 9 signaling pathway | 4/530 | 16/29008 | 0.0001684035257 | 0.002708027391 | 0.00192655971 |
| **273** | WT | GO:0070431 | nucleotide-binding oligomerization domain containing 2 signaling pathway | 4/530 | 16/29008 | 0.0001684035257 | 0.002708027391 | 0.00192655971 |
| **274** | WT | GO:0032956 | regulation of actin cytoskeleton organization | 18/530 | 368/29008 | 0.0001696099985 | 0.002717474064 | 0.001933280313 |
| **275** | WT | GO:0072593 | reactive oxygen species metabolic process | 14/530 | 244/29008 | 0.0001764298632 | 0.00281646218 | 0.002003702982 |
| **276** | WT | GO:0022604 | regulation of cell morphogenesis | 17/530 | 337/29008 | 0.0001776467642 | 0.002825613387 | 0.002010213385 |
| **277** | WT | GO:0046323 | glucose import | 8/530 | 86/29008 | 0.0001793861661 | 0.002842979311 | 0.002022567946 |
| **278** | WT | GO:0032613 | interleukin-10 production | 7/530 | 65/29008 | 0.0001820408843 | 0.002856655828 | 0.002032297768 |
| **279** | WT | GO:0034142 | toll-like receptor 4 signaling pathway | 6/530 | 46/29008 | 0.0001822012829 | 0.002856655828 | 0.002032297768 |
| **280** | WT | GO:0060122 | inner ear receptor cell stereocilium organization | 6/530 | 46/29008 | 0.0001822012829 | 0.002856655828 | 0.002032297768 |
| **281** | WT | GO:0097193 | intrinsic apoptotic signaling pathway | 16/530 | 307/29008 | 0.0001892386872 | 0.002956433583 | 0.002103282206 |
| **282** | WT | GO:0006631 | fatty acid metabolic process | 20/530 | 439/29008 | 0.0001943004286 | 0.003024747807 | 0.002151882686 |
| **283** | WT | GO:0006163 | purine nucleotide metabolic process | 18/530 | 373/29008 | 0.0002000957094 | 0.003087964299 | 0.00219685651 |
| **284** | WT | GO:0050900 | leukocyte migration | 18/530 | 373/29008 | 0.0002000957094 | 0.003087964299 | 0.00219685651 |
| **285** | WT | GO:0060761 | negative regulation of response to cytokine stimulus | 7/530 | 66/29008 | 0.0002004714864 | 0.003087964299 | 0.00219685651 |
| **286** | WT | GO:0051346 | negative regulation of hydrolase activity | 18/530 | 374/29008 | 0.0002067370822 | 0.003173341927 | 0.002257596331 |
| **287** | WT | GO:0007162 | negative regulation of cell adhesion | 16/530 | 310/29008 | 0.0002111829609 | 0.00323028989 | 0.002298110563 |
| **288** | WT | GO:0071360 | cellular response to exogenous dsRNA | 4/530 | 17/29008 | 0.0002170478162 | 0.003297023921 | 0.002345586854 |
| **289** | WT | GO:2000047 | regulation of cell-cell adhesion mediated by cadherin | 4/530 | 17/29008 | 0.0002170478162 | 0.003297023921 | 0.002345586854 |
| **290** | WT | GO:0001894 | tissue homeostasis | 15/530 | 280/29008 | 0.0002216640523 | 0.003344004089 | 0.002379009743 |
| **291** | WT | GO:0060560 | developmental growth involved in morphogenesis | 15/530 | 280/29008 | 0.0002216640523 | 0.003344004089 | 0.002379009743 |
| **292** | WT | GO:0002822 | regulation of adaptive immune response based on somatic recombination of immune receptors built from immunoglobulin superfamily domains | 13/530 | 220/29008 | 0.000223655808 | 0.003362496565 | 0.002392165761 |
| **293** | WT | GO:0006066 | alcohol metabolic process | 17/530 | 344/29008 | 0.0002261771603 | 0.003377271203 | 0.002402676816 |
| **294** | WT | GO:0042063 | gliogenesis | 17/530 | 344/29008 | 0.0002261771603 | 0.003377271203 | 0.002402676816 |
| **295** | WT | GO:0008593 | regulation of Notch signaling pathway | 8/530 | 89/29008 | 0.0002276626025 | 0.00338792822 | 0.002410258489 |
| **296** | WT | GO:0032607 | interferon-alpha production | 5/530 | 31/29008 | 0.0002292041785 | 0.003399345756 | 0.002418381215 |
| **297** | WT | GO:0035966 | response to topologically incorrect protein | 10/530 | 138/29008 | 0.0002336964033 | 0.003432161471 | 0.002441727151 |
| **298** | WT | GO:0046165 | alcohol biosynthetic process | 10/530 | 138/29008 | 0.0002336964033 | 0.003432161471 | 0.002441727151 |
| **299** | WT | GO:0048639 | positive regulation of developmental growth | 13/530 | 221/29008 | 0.0002337622505 | 0.003432161471 | 0.002441727151 |
| **300** | WT | GO:0010720 | positive regulation of cell development | 18/530 | 378/29008 | 0.0002352679363 | 0.00343590665 | 0.002444391567 |
| **301** | WT | GO:0008630 | intrinsic apoptotic signaling pathway in response to DNA damage | 9/530 | 113/29008 | 0.0002355826655 | 0.00343590665 | 0.002444391567 |
| **302** | WT | GO:1901224 | positive regulation of NIK/NF-kappaB signaling | 7/530 | 68/29008 | 0.000241849056 | 0.003515620384 | 0.002501101949 |
| **303** | WT | GO:0002685 | regulation of leukocyte migration | 13/530 | 223/29008 | 0.0002551522718 | 0.003696760637 | 0.002629969742 |
| **304** | WT | GO:0007157 | heterophilic cell-cell adhesion via plasma membrane cell adhesion molecules | 6/530 | 49/29008 | 0.0002597272073 | 0.003750665921 | 0.002668319335 |
| **305** | WT | GO:0009266 | response to temperature stimulus | 11/530 | 167/29008 | 0.0002663183856 | 0.003833238403 | 0.002727063503 |
| **306** | WT | GO:1902042 | negative regulation of extrinsic apoptotic signaling pathway via death domain receptors | 5/530 | 32/29008 | 0.0002675970503 | 0.003839055722 | 0.002731202092 |
| **307** | WT | GO:0032970 | regulation of actin filament-based process | 19/530 | 416/29008 | 0.0002699418128 | 0.003860079994 | 0.002746159296 |
| **308** | WT | GO:0070423 | nucleotide-binding oligomerization domain containing signaling pathway | 4/530 | 18/29008 | 0.0002750441782 | 0.003907585573 | 0.002779955976 |
| **309** | WT | GO:1900225 | regulation of NLRP3 inflammasome complex assembly | 4/530 | 18/29008 | 0.0002750441782 | 0.003907585573 | 0.002779955976 |
| **310** | WT | GO:0051924 | regulation of calcium ion transport | 15/530 | 286/29008 | 0.0002781295699 | 0.003938673586 | 0.00280207278 |
| **311** | WT | GO:0046324 | regulation of glucose import | 7/530 | 70/29008 | 0.0002898384686 | 0.004091288994 | 0.002910647271 |
| **312** | WT | GO:0007015 | actin filament organization | 20/530 | 453/29008 | 0.0002920281321 | 0.004108985576 | 0.002923237071 |
| **313** | WT | GO:0051480 | regulation of cytosolic calcium ion concentration | 18/530 | 386/29008 | 0.0003027901301 | 0.004246800866 | 0.003021282381 |
| **314** | WT | GO:0051017 | actin filament bundle assembly | 11/530 | 172/29008 | 0.000343064258 | 0.00478547707 | 0.003404510362 |
| **315** | WT | GO:0035872 | nucleotide-binding domain, leucine rich repeat containing receptor signaling pathway | 4/530 | 19/29008 | 0.0003433770563 | 0.00478547707 | 0.003404510362 |
| **316** | WT | GO:0032963 | collagen metabolic process | 9/530 | 119/29008 | 0.0003466952921 | 0.004816431431 | 0.003426532084 |
| **317** | WT | GO:0051258 | protein polymerization | 15/530 | 293/29008 | 0.0003592936492 | 0.004975707003 | 0.003539844786 |
| **318** | WT | GO:1902041 | regulation of extrinsic apoptotic signaling pathway via death domain receptors | 6/530 | 52/29008 | 0.0003610700227 | 0.004984583018 | 0.003546159409 |
| **319** | WT | GO:0051962 | positive regulation of nervous system development | 17/530 | 359/29008 | 0.0003703924664 | 0.005097250557 | 0.003626313967 |
| **320** | WT | GO:0009408 | response to heat | 8/530 | 96/29008 | 0.0003825285722 | 0.005247813849 | 0.003733428531 |
| **321** | WT | GO:1901361 | organic cyclic compound catabolic process | 20/530 | 463/29008 | 0.0003859448813 | 0.005278187006 | 0.003755036769 |
| **322** | WT | GO:0043467 | regulation of generation of precursor metabolites and energy | 10/530 | 147/29008 | 0.0003881444852 | 0.00529178351 | 0.003764709669 |
| **323** | WT | GO:0061572 | actin filament bundle organization | 11/530 | 175/29008 | 0.0003974634954 | 0.005402058034 | 0.003843161776 |
| **324** | WT | GO:0003018 | vascular process in circulatory system | 13/530 | 234/29008 | 0.0004051730556 | 0.005489844797 | 0.003905615516 |
| **325** | WT | GO:0046651 | lymphocyte proliferation | 16/530 | 330/29008 | 0.0004222685109 | 0.005679478362 | 0.004040525669 |
| **326** | WT | GO:0002755 | MyD88-dependent toll-like receptor signaling pathway | 4/530 | 20/29008 | 0.0004230499828 | 0.005679478362 | 0.004040525669 |
| **327** | WT | GO:0044546 | NLRP3 inflammasome complex assembly | 4/530 | 20/29008 | 0.0004230499828 | 0.005679478362 | 0.004040525669 |
| **328** | WT | GO:0050769 | positive regulation of neurogenesis | 15/530 | 298/29008 | 0.0004290379974 | 0.005742307344 | 0.004085223807 |
| **329** | WT | GO:0009161 | ribonucleoside monophosphate metabolic process | 6/530 | 54/29008 | 0.0004441906551 | 0.00592704248 | 0.004216649092 |
| **330** | WT | GO:1902806 | regulation of cell cycle G1/S phase transition | 10/530 | 150/29008 | 0.00045550429 | 0.006059587373 | 0.004310944907 |
| **331** | WT | GO:0031589 | cell-substrate adhesion | 17/530 | 366/29008 | 0.0004612922823 | 0.006118045678 | 0.004352533635 |
| **332** | WT | GO:0019373 | epoxygenase P450 pathway | 5/530 | 36/29008 | 0.0004717650894 | 0.006238098621 | 0.004437942359 |
| **333** | WT | GO:0050921 | positive regulation of chemotaxis | 10/530 | 151/29008 | 0.0004800058994 | 0.006328005701 | 0.004501904547 |
| **334** | WT | GO:0032964 | collagen biosynthetic process | 6/530 | 55/29008 | 0.0004909794481 | 0.006443643286 | 0.004584172073 |
| **335** | WT | GO:0006914 | autophagy | 19/530 | 437/29008 | 0.0004946486987 | 0.006443643286 | 0.004584172073 |
| **336** | WT | GO:0019439 | aromatic compound catabolic process | 19/530 | 437/29008 | 0.0004946486987 | 0.006443643286 | 0.004584172073 |
| **337** | WT | GO:0061919 | process utilizing autophagic mechanism | 19/530 | 437/29008 | 0.0004946486987 | 0.006443643286 | 0.004584172073 |
| **338** | WT | GO:0002824 | positive regulation of adaptive immune response based on somatic recombination of immune receptors built from immunoglobulin superfamily domains | 10/530 | 152/29008 | 0.0005055909279 | 0.006566698739 | 0.004671716858 |
| **339** | WT | GO:0002281 | macrophage activation involved in immune response | 4/530 | 21/29008 | 0.0005150820137 | 0.006631114487 | 0.004717543863 |
| **340** | WT | GO:0002577 | regulation of antigen processing and presentation | 4/530 | 21/29008 | 0.0005150820137 | 0.006631114487 | 0.004717543863 |
| **341** | WT | GO:0007252 | I-kappaB phosphorylation | 4/530 | 21/29008 | 0.0005150820137 | 0.006631114487 | 0.004717543863 |
| **342** | WT | GO:0061097 | regulation of protein tyrosine kinase activity | 7/530 | 77/29008 | 0.000521132214 | 0.006689387191 | 0.004759000551 |
| **343** | WT | GO:0071674 | mononuclear cell migration | 11/530 | 181/29008 | 0.0005281425509 | 0.006759608741 | 0.004808957951 |
| **344** | WT | GO:0002687 | positive regulation of leukocyte migration | 10/530 | 153/29008 | 0.0005322961302 | 0.006792965149 | 0.00483268855 |
| **345** | WT | GO:0050670 | regulation of lymphocyte proliferation | 13/530 | 241/29008 | 0.0005352588862 | 0.006810975392 | 0.004845501496 |
| **346** | WT | GO:0015980 | energy derivation by oxidation of organic compounds | 15/530 | 305/29008 | 0.0005459689 | 0.006927177661 | 0.004928170752 |
| **347** | WT | GO:0052547 | regulation of peptidase activity | 19/530 | 441/29008 | 0.0005522076741 | 0.006986143197 | 0.004970120337 |
| **348** | WT | GO:0005977 | glycogen metabolic process | 7/530 | 78/29008 | 0.0005636362654 | 0.007049467821 | 0.005015171089 |
| **349** | WT | GO:0006073 | cellular glucan metabolic process | 7/530 | 78/29008 | 0.0005636362654 | 0.007049467821 | 0.005015171089 |
| **350** | WT | GO:0044042 | glucan metabolic process | 7/530 | 78/29008 | 0.0005636362654 | 0.007049467821 | 0.005015171089 |
| **351** | WT | GO:2000379 | positive regulation of reactive oxygen species metabolic process | 7/530 | 78/29008 | 0.0005636362654 | 0.007049467821 | 0.005015171089 |
| **352** | WT | GO:0031334 | positive regulation of protein-containing complex assembly | 13/530 | 243/29008 | 0.0005783442605 | 0.007212873022 | 0.005131421713 |
| **353** | WT | GO:0098742 | cell-cell adhesion via plasma-membrane adhesion molecules | 12/530 | 213/29008 | 0.0005910925915 | 0.00735098152 | 0.005229675619 |
| **354** | WT | GO:0072527 | pyrimidine-containing compound metabolic process | 6/530 | 57/29008 | 0.000596002045 | 0.007391098806 | 0.005258216079 |
| **355** | WT | GO:0043523 | regulation of neuron apoptotic process | 14/530 | 276/29008 | 0.0006114038877 | 0.007560741034 | 0.005378903884 |
| **356** | WT | GO:0014909 | smooth muscle cell migration | 8/530 | 103/29008 | 0.0006139366379 | 0.007566705952 | 0.005383147479 |
| **357** | WT | GO:0035634 | response to stilbenoid | 4/530 | 22/29008 | 0.0006205043606 | 0.007566705952 | 0.005383147479 |
| **358** | WT | GO:0060749 | mammary gland alveolus development | 4/530 | 22/29008 | 0.0006205043606 | 0.007566705952 | 0.005383147479 |
| **359** | WT | GO:0061377 | mammary gland lobule development | 4/530 | 22/29008 | 0.0006205043606 | 0.007566705952 | 0.005383147479 |
| **360** | WT | GO:0140632 | inflammasome complex assembly | 4/530 | 22/29008 | 0.0006205043606 | 0.007566705952 | 0.005383147479 |
| **361** | WT | GO:0043330 | response to exogenous dsRNA | 6/530 | 58/29008 | 0.0006546465196 | 0.007873947921 | 0.005601727246 |
| **362** | WT | GO:0045123 | cellular extravasation | 7/530 | 80/29008 | 0.0006568951237 | 0.007873947921 | 0.005601727246 |
| **363** | WT | GO:0032621 | interleukin-18 production | 3/530 | 10/29008 | 0.00066136288 | 0.007873947921 | 0.005601727246 |
| **364** | WT | GO:0034163 | regulation of toll-like receptor 9 signaling pathway | 3/530 | 10/29008 | 0.00066136288 | 0.007873947921 | 0.005601727246 |
| **365** | WT | GO:1900246 | positive regulation of RIG-I signaling pathway | 3/530 | 10/29008 | 0.00066136288 | 0.007873947921 | 0.005601727246 |
| **366** | WT | GO:1902033 | regulation of hematopoietic stem cell proliferation | 3/530 | 10/29008 | 0.00066136288 | 0.007873947921 | 0.005601727246 |
| **367** | WT | GO:1902732 | positive regulation of chondrocyte proliferation | 3/530 | 10/29008 | 0.00066136288 | 0.007873947921 | 0.005601727246 |
| **368** | WT | GO:0002698 | negative regulation of immune effector process | 9/530 | 130/29008 | 0.0006618420917 | 0.007873947921 | 0.005601727246 |
| **369** | WT | GO:0043406 | positive regulation of MAP kinase activity | 9/530 | 130/29008 | 0.0006618420917 | 0.007873947921 | 0.005601727246 |
| **370** | WT | GO:0032506 | cytokinetic process | 5/530 | 39/29008 | 0.0006888179335 | 0.008172731697 | 0.00581428964 |
| **371** | WT | GO:0008217 | regulation of blood pressure | 12/530 | 217/29008 | 0.0006964058653 | 0.008240489888 | 0.005862494545 |
| **372** | WT | GO:0046700 | heterocycle catabolic process | 18/530 | 415/29008 | 0.0007082530531 | 0.008358147588 | 0.005946199232 |
| **373** | WT | GO:0032731 | positive regulation of interleukin-1 beta production | 6/530 | 59/29008 | 0.0007176618777 | 0.008430347293 | 0.005997563942 |
| **374** | WT | GO:0002821 | positive regulation of adaptive immune response | 10/530 | 159/29008 | 0.0007182118195 | 0.008430347293 | 0.005997563942 |
| **375** | WT | GO:0050767 | regulation of neurogenesis | 19/530 | 451/29008 | 0.0007220676979 | 0.00845300585 | 0.006013683816 |
| **376** | WT | GO:0001659 | temperature homeostasis | 11/530 | 188/29008 | 0.0007241453901 | 0.008454782613 | 0.006014947851 |
| **377** | WT | GO:0071359 | cellular response to dsRNA | 4/530 | 23/29008 | 0.0007403572112 | 0.008621135695 | 0.006133295674 |
| **378** | WT | GO:0009150 | purine ribonucleotide metabolic process | 16/530 | 348/29008 | 0.0007475148084 | 0.00868145505 | 0.0061762084 |
| **379** | WT | GO:2000377 | regulation of reactive oxygen species metabolic process | 10/530 | 160/29008 | 0.0007538559005 | 0.008718630306 | 0.006202655825 |
| **380** | WT | GO:0002285 | lymphocyte activation involved in immune response | 12/530 | 219/29008 | 0.0007546878169 | 0.008718630306 | 0.006202655825 |
| **381** | WT | GO:0010543 | regulation of platelet activation | 5/530 | 40/29008 | 0.0007755199354 | 0.008912388786 | 0.006340500547 |
| **382** | WT | GO:0050850 | positive regulation of calcium-mediated signaling | 5/530 | 40/29008 | 0.0007755199354 | 0.008912388786 | 0.006340500547 |
| **383** | WT | GO:0033559 | unsaturated fatty acid metabolic process | 9/530 | 133/29008 | 0.0007797475731 | 0.008937440045 | 0.00635832265 |
| **384** | WT | GO:0006874 | cellular calcium ion homeostasis | 20/530 | 490/29008 | 0.000781771521 | 0.008937440045 | 0.00635832265 |
| **385** | WT | GO:2001238 | positive regulation of extrinsic apoptotic signaling pathway | 6/530 | 60/29008 | 0.000785265749 | 0.00895406919 | 0.006370153048 |
| **386** | WT | GO:0072676 | lymphocyte migration | 8/530 | 107/29008 | 0.0007900622977 | 0.008985423541 | 0.006392459333 |
| **387** | WT | GO:0050848 | regulation of calcium-mediated signaling | 7/530 | 83/29008 | 0.0008192836691 | 0.009269730173 | 0.006594722317 |
| **388** | WT | GO:0072089 | stem cell proliferation | 7/530 | 83/29008 | 0.0008192836691 | 0.009269730173 | 0.006594722317 |
| **389** | WT | GO:0034655 | nucleobase-containing compound catabolic process | 17/530 | 386/29008 | 0.0008341395156 | 0.009413553917 | 0.006697042194 |
| **390** | WT | GO:0051054 | positive regulation of DNA metabolic process | 12/530 | 222/29008 | 0.0008497206763 | 0.009540342121 | 0.006787242554 |
| **391** | WT | GO:2001020 | regulation of response to DNA damage stimulus | 12/530 | 222/29008 | 0.0008497206763 | 0.009540342121 | 0.006787242554 |
| **392** | WT | GO:0006457 | protein folding | 10/530 | 163/29008 | 0.0008696277538 | 0.009670331574 | 0.006879720365 |
| **393** | WT | GO:0006220 | pyrimidine nucleotide metabolic process | 5/530 | 41/29008 | 0.0008701095608 | 0.009670331574 | 0.006879720365 |
| **394** | WT | GO:0006953 | acute-phase response | 5/530 | 41/29008 | 0.0008701095608 | 0.009670331574 | 0.006879720365 |
| **395** | WT | GO:0032733 | positive regulation of interleukin-10 production | 5/530 | 41/29008 | 0.0008701095608 | 0.009670331574 | 0.006879720365 |
| **396** | WT | GO:0035988 | chondrocyte proliferation | 4/530 | 24/29008 | 0.0008756867305 | 0.00970773926 | 0.006906333146 |
| **397** | WT | GO:0006809 | nitric oxide biosynthetic process | 7/530 | 84/29008 | 0.0008799326902 | 0.00973023806 | 0.006922339365 |
| **398** | KO | GO:0009615 | response to virus | 103/2334 | 312/29008 | 5.60E-37 | 3.41E-33 | 1.92E-33 |
| **399** | KO | GO:0001819 | positive regulation of cytokine production | 134/2334 | 500/29008 | 1.11E-36 | 3.41E-33 | 1.92E-33 |
| **400** | KO | GO:0032103 | positive regulation of response to external stimulus | 118/2334 | 427/29008 | 8.73E-34 | 1.79E-30 | 1.01E-30 |
| **401** | KO | GO:0051607 | defense response to virus | 87/2334 | 255/29008 | 1.24E-32 | 1.52E-29 | 8.57E-30 |
| **402** | KO | GO:0140546 | defense response to symbiont | 87/2334 | 255/29008 | 1.24E-32 | 1.52E-29 | 8.57E-30 |
| **403** | KO | GO:0002831 | regulation of response to biotic stimulus | 97/2334 | 337/29008 | 1.69E-29 | 1.73E-26 | 9.73E-27 |
| **404** | KO | GO:0050900 | leukocyte migration | 101/2334 | 373/29008 | 2.68E-28 | 2.35E-25 | 1.32E-25 |
| **405** | KO | GO:0007159 | leukocyte cell-cell adhesion | 100/2334 | 373/29008 | 1.18E-27 | 9.04E-25 | 5.09E-25 |
| **406** | KO | GO:0031349 | positive regulation of defense response | 82/2334 | 273/29008 | 1.63E-26 | 1.12E-23 | 6.28E-24 |
| **407** | KO | GO:0002833 | positive regulation of response to biotic stimulus | 63/2334 | 173/29008 | 8.73E-26 | 5.37E-23 | 3.02E-23 |
| **408** | KO | GO:0002697 | regulation of immune effector process | 104/2334 | 424/29008 | 2.47E-25 | 1.38E-22 | 7.75E-23 |
| **409** | KO | GO:0002274 | myeloid leukocyte activation | 76/2334 | 261/29008 | 9.74E-24 | 4.99E-21 | 2.81E-21 |
| **410** | KO | GO:0045088 | regulation of innate immune response | 68/2334 | 217/29008 | 2.22E-23 | 1.05E-20 | 5.90E-21 |
| **411** | KO | GO:0045785 | positive regulation of cell adhesion | 107/2334 | 471/29008 | 3.51E-23 | 1.54E-20 | 8.68E-21 |
| **412** | KO | GO:0097529 | myeloid leukocyte migration | 67/2334 | 226/29008 | 1.45E-21 | 5.95E-19 | 3.35E-19 |
| **413** | KO | GO:0044282 | small molecule catabolic process | 84/2334 | 335/29008 | 2.27E-21 | 8.70E-19 | 4.90E-19 |
| **414** | KO | GO:0050727 | regulation of inflammatory response | 85/2334 | 342/29008 | 2.44E-21 | 8.81E-19 | 4.96E-19 |
| **415** | KO | GO:0022407 | regulation of cell-cell adhesion | 101/2334 | 457/29008 | 5.12E-21 | 1.75E-18 | 9.84E-19 |
| **416** | KO | GO:0071706 | tumor necrosis factor superfamily cytokine production | 60/2334 | 191/29008 | 7.42E-21 | 2.40E-18 | 1.35E-18 |
| **417** | KO | GO:0019221 | cytokine-mediated signaling pathway | 92/2334 | 397/29008 | 9.88E-21 | 3.03E-18 | 1.71E-18 |
| **418** | KO | GO:0032640 | tumor necrosis factor production | 59/2334 | 188/29008 | 1.65E-20 | 4.83E-18 | 2.72E-18 |
| **419** | KO | GO:0006631 | fatty acid metabolic process | 97/2334 | 439/29008 | 3.15E-20 | 8.80E-18 | 4.95E-18 |
| **420** | KO | GO:1903555 | regulation of tumor necrosis factor superfamily cytokine production | 58/2334 | 186/29008 | 4.93E-20 | 1.32E-17 | 7.41E-18 |
| **421** | KO | GO:1903037 | regulation of leukocyte cell-cell adhesion | 81/2334 | 333/29008 | 8.87E-20 | 2.22E-17 | 1.25E-17 |
| **422** | KO | GO:0002221 | pattern recognition receptor signaling pathway | 55/2334 | 171/29008 | 9.04E-20 | 2.22E-17 | 1.25E-17 |
| **423** | KO | GO:0032680 | regulation of tumor necrosis factor production | 57/2334 | 183/29008 | 1.09E-19 | 2.59E-17 | 1.46E-17 |
| **424** | KO | GO:0002699 | positive regulation of immune effector process | 75/2334 | 296/29008 | 1.65E-19 | 3.74E-17 | 2.11E-17 |
| **425** | KO | GO:0032760 | positive regulation of tumor necrosis factor production | 43/2334 | 110/29008 | 2.01E-19 | 4.40E-17 | 2.48E-17 |
| **426** | KO | GO:0050792 | regulation of viral process | 55/2334 | 174/29008 | 2.24E-19 | 4.74E-17 | 2.67E-17 |
| **427** | KO | GO:0002683 | negative regulation of immune system process | 98/2334 | 460/29008 | 2.99E-19 | 6.13E-17 | 3.45E-17 |
| **428** | KO | GO:0002685 | regulation of leukocyte migration | 63/2334 | 223/29008 | 3.56E-19 | 7.05E-17 | 3.97E-17 |
| **429** | KO | GO:1903557 | positive regulation of tumor necrosis factor superfamily cytokine production | 43/2334 | 112/29008 | 4.53E-19 | 8.69E-17 | 4.89E-17 |
| **430** | KO | GO:0060326 | cell chemotaxis | 76/2334 | 312/29008 | 1.11E-18 | 2.07E-16 | 1.16E-16 |
| **431** | KO | GO:0045089 | positive regulation of innate immune response | 46/2334 | 131/29008 | 1.76E-18 | 3.19E-16 | 1.79E-16 |
| **432** | KO | GO:0030595 | leukocyte chemotaxis | 62/2334 | 226/29008 | 3.38E-18 | 5.93E-16 | 3.34E-16 |
| **433** | KO | GO:0032943 | mononuclear cell proliferation | 78/2334 | 334/29008 | 5.46E-18 | 9.32E-16 | 5.25E-16 |
| **434** | KO | GO:0032635 | interleukin-6 production | 53/2334 | 174/29008 | 6.28E-18 | 1.04E-15 | 5.87E-16 |
| **435** | KO | GO:0022409 | positive regulation of cell-cell adhesion | 71/2334 | 288/29008 | 7.60E-18 | 1.23E-15 | 6.92E-16 |
| **436** | KO | GO:0070661 | leukocyte proliferation | 81/2334 | 358/29008 | 9.37E-18 | 1.48E-15 | 8.31E-16 |
| **437** | KO | GO:1903900 | regulation of viral life cycle | 49/2334 | 153/29008 | 1.10E-17 | 1.69E-15 | 9.52E-16 |
| **438** | KO | GO:0050863 | regulation of T cell activation | 78/2334 | 339/29008 | 1.36E-17 | 2.04E-15 | 1.15E-15 |
| **439** | KO | GO:1903039 | positive regulation of leukocyte cell-cell adhesion | 63/2334 | 240/29008 | 1.98E-17 | 2.90E-15 | 1.63E-15 |
| **440** | KO | GO:0002687 | positive regulation of leukocyte migration | 48/2334 | 153/29008 | 6.02E-17 | 8.60E-15 | 4.84E-15 |
| **441** | KO | GO:0032675 | regulation of interleukin-6 production | 50/2334 | 166/29008 | 9.02E-17 | 1.26E-14 | 7.09E-15 |
| **442** | KO | GO:0090407 | organophosphate biosynthetic process | 95/2334 | 476/29008 | 9.74E-17 | 1.33E-14 | 7.48E-15 |
| **443** | KO | GO:0006066 | alcohol metabolic process | 77/2334 | 344/29008 | 1.14E-16 | 1.53E-14 | 8.60E-15 |
| **444** | KO | GO:0046651 | lymphocyte proliferation | 75/2334 | 330/29008 | 1.18E-16 | 1.54E-14 | 8.66E-15 |
| **445** | KO | GO:0044242 | cellular lipid catabolic process | 59/2334 | 224/29008 | 1.73E-16 | 2.22E-14 | 1.25E-14 |
| **446** | KO | GO:0097530 | granulocyte migration | 48/2334 | 157/29008 | 1.91E-16 | 2.39E-14 | 1.35E-14 |
| **447** | KO | GO:0006790 | sulfur compound metabolic process | 74/2334 | 326/29008 | 2.02E-16 | 2.48E-14 | 1.40E-14 |
| **448** | KO | GO:0002224 | toll-like receptor signaling pathway | 42/2334 | 124/29008 | 2.38E-16 | 2.84E-14 | 1.60E-14 |
| **449** | KO | GO:0032944 | regulation of mononuclear cell proliferation | 62/2334 | 245/29008 | 2.41E-16 | 2.84E-14 | 1.60E-14 |
| **450** | KO | GO:0042098 | T cell proliferation | 58/2334 | 221/29008 | 3.73E-16 | 4.32E-14 | 2.43E-14 |
| **451** | KO | GO:0070663 | regulation of leukocyte proliferation | 64/2334 | 261/29008 | 4.19E-16 | 4.77E-14 | 2.68E-14 |
| **452** | KO | GO:0009117 | nucleotide metabolic process | 90/2334 | 451/29008 | 6.34E-16 | 7.08E-14 | 3.99E-14 |
| **453** | KO | GO:1990266 | neutrophil migration | 42/2334 | 128/29008 | 8.68E-16 | 9.52E-14 | 5.36E-14 |
| **454** | KO | GO:0071674 | mononuclear cell migration | 51/2334 | 181/29008 | 9.58E-16 | 1.03E-13 | 5.81E-14 |
| **455** | KO | GO:0002237 | response to molecule of bacterial origin | 84/2334 | 409/29008 | 1.04E-15 | 1.11E-13 | 6.23E-14 |
| **456** | KO | GO:0072521 | purine-containing compound metabolic process | 82/2334 | 395/29008 | 1.21E-15 | 1.26E-13 | 7.07E-14 |
| **457** | KO | GO:0009259 | ribonucleotide metabolic process | 78/2334 | 367/29008 | 1.55E-15 | 1.58E-13 | 8.91E-14 |
| **458** | KO | GO:0002700 | regulation of production of molecular mediator of immune response | 50/2334 | 177/29008 | 1.63E-15 | 1.62E-13 | 9.10E-14 |
| **459** | KO | GO:0032606 | type I interferon production | 37/2334 | 103/29008 | 1.63E-15 | 1.62E-13 | 9.10E-14 |
| **460** | KO | GO:0006753 | nucleoside phosphate metabolic process | 90/2334 | 460/29008 | 2.22E-15 | 2.15E-13 | 1.21E-13 |
| **461** | KO | GO:0010876 | lipid localization | 87/2334 | 437/29008 | 2.24E-15 | 2.15E-13 | 1.21E-13 |
| **462** | KO | GO:0019693 | ribose phosphate metabolic process | 79/2334 | 379/29008 | 3.17E-15 | 2.99E-13 | 1.68E-13 |
| **463** | KO | GO:0032479 | regulation of type I interferon production | 36/2334 | 101/29008 | 5.19E-15 | 4.83E-13 | 2.72E-13 |
| **464** | KO | GO:0016032 | viral process | 74/2334 | 346/29008 | 5.80E-15 | 5.32E-13 | 3.00E-13 |
| **465** | KO | GO:0050670 | regulation of lymphocyte proliferation | 59/2334 | 241/29008 | 6.34E-15 | 5.73E-13 | 3.22E-13 |
| **466** | KO | GO:0030593 | neutrophil chemotaxis | 36/2334 | 102/29008 | 7.41E-15 | 6.50E-13 | 3.66E-13 |
| **467** | KO | GO:0048525 | negative regulation of viral process | 36/2334 | 102/29008 | 7.41E-15 | 6.50E-13 | 3.66E-13 |
| **468** | KO | GO:0046395 | carboxylic acid catabolic process | 55/2334 | 216/29008 | 8.73E-15 | 7.45E-13 | 4.19E-13 |
| **469** | KO | GO:0050870 | positive regulation of T cell activation | 55/2334 | 216/29008 | 8.73E-15 | 7.45E-13 | 4.19E-13 |
| **470** | KO | GO:0019058 | viral life cycle | 61/2334 | 257/29008 | 1.02E-14 | 8.56E-13 | 4.82E-13 |
| **471** | KO | GO:0002263 | cell activation involved in immune response | 71/2334 | 328/29008 | 1.08E-14 | 8.87E-13 | 4.99E-13 |
| **472** | KO | GO:0016042 | lipid catabolic process | 71/2334 | 328/29008 | 1.08E-14 | 8.87E-13 | 4.99E-13 |
| **473** | KO | GO:0015849 | organic acid transport | 69/2334 | 314/29008 | 1.17E-14 | 9.44E-13 | 5.31E-13 |
| **474** | KO | GO:0032755 | positive regulation of interleukin-6 production | 37/2334 | 109/29008 | 1.31E-14 | 1.04E-12 | 5.87E-13 |
| **475** | KO | GO:0016054 | organic acid catabolic process | 55/2334 | 218/29008 | 1.33E-14 | 1.05E-12 | 5.90E-13 |
| **476** | KO | GO:0032481 | positive regulation of type I interferon production | 29/2334 | 69/29008 | 1.51E-14 | 1.18E-12 | 6.61E-13 |
| **477** | KO | GO:0042060 | wound healing | 75/2334 | 361/29008 | 1.90E-14 | 1.46E-12 | 8.22E-13 |
| **478** | KO | GO:0071621 | granulocyte chemotaxis | 40/2334 | 127/29008 | 1.98E-14 | 1.50E-12 | 8.47E-13 |
| **479** | KO | GO:0034341 | response to interferon-gamma | 42/2334 | 139/29008 | 2.25E-14 | 1.69E-12 | 9.50E-13 |
| **480** | KO | GO:0002703 | regulation of leukocyte mediated immunity | 66/2334 | 297/29008 | 2.50E-14 | 1.85E-12 | 1.04E-12 |
| **481** | KO | GO:1903131 | mononuclear cell differentiation | 91/2334 | 489/29008 | 3.55E-14 | 2.60E-12 | 1.46E-12 |
| **482** | KO | GO:0072593 | reactive oxygen species metabolic process | 58/2334 | 244/29008 | 4.27E-14 | 3.09E-12 | 1.74E-12 |
| **483** | KO | GO:0032102 | negative regulation of response to external stimulus | 79/2334 | 398/29008 | 5.13E-14 | 3.64E-12 | 2.05E-12 |
| **484** | KO | GO:0002696 | positive regulation of leukocyte activation | 92/2334 | 500/29008 | 5.16E-14 | 3.64E-12 | 2.05E-12 |
| **485** | KO | GO:0002366 | leukocyte activation involved in immune response | 69/2334 | 324/29008 | 5.95E-14 | 4.15E-12 | 2.34E-12 |
| **486** | KO | GO:0007162 | negative regulation of cell adhesion | 67/2334 | 310/29008 | 6.55E-14 | 4.52E-12 | 2.54E-12 |
| **487** | KO | GO:0018212 | peptidyl-tyrosine modification | 69/2334 | 325/29008 | 6.97E-14 | 4.76E-12 | 2.68E-12 |
| **488** | KO | GO:0030258 | lipid modification | 53/2334 | 213/29008 | 7.34E-14 | 4.96E-12 | 2.79E-12 |
| **489** | KO | GO:0032608 | interferon-beta production | 28/2334 | 68/29008 | 8.01E-14 | 5.29E-12 | 2.98E-12 |
| **490** | KO | GO:0032648 | regulation of interferon-beta production | 28/2334 | 68/29008 | 8.01E-14 | 5.29E-12 | 2.98E-12 |
| **491** | KO | GO:0050764 | regulation of phagocytosis | 37/2334 | 115/29008 | 8.86E-14 | 5.79E-12 | 3.26E-12 |
| **492** | KO | GO:0002367 | cytokine production involved in immune response | 36/2334 | 111/29008 | 1.45E-13 | 9.37E-12 | 5.28E-12 |
| **493** | KO | GO:0006644 | phospholipid metabolic process | 73/2334 | 360/29008 | 1.53E-13 | 9.78E-12 | 5.51E-12 |
| **494** | KO | GO:0007015 | actin filament organization | 85/2334 | 453/29008 | 1.54E-13 | 9.78E-12 | 5.51E-12 |
| **495** | KO | GO:0097191 | extrinsic apoptotic signaling pathway | 56/2334 | 238/29008 | 1.84E-13 | 1.15E-11 | 6.49E-12 |
| **496** | KO | GO:0062012 | regulation of small molecule metabolic process | 74/2334 | 369/29008 | 1.88E-13 | 1.17E-11 | 6.56E-12 |
| **497** | KO | GO:0042116 | macrophage activation | 34/2334 | 101/29008 | 2.01E-13 | 1.24E-11 | 6.96E-12 |
| **498** | KO | GO:0016053 | organic acid biosynthetic process | 64/2334 | 296/29008 | 2.32E-13 | 1.41E-11 | 7.96E-12 |
| **499** | KO | GO:0051090 | regulation of DNA-binding transcription factor activity | 81/2334 | 425/29008 | 2.43E-13 | 1.47E-11 | 8.25E-12 |
| **500** | KO | GO:0006869 | lipid transport | 76/2334 | 387/29008 | 2.74E-13 | 1.63E-11 | 9.20E-12 |
| **501** | KO | GO:0032496 | response to lipopolysaccharide | 76/2334 | 388/29008 | 3.14E-13 | 1.86E-11 | 1.05E-11 |
| **502** | KO | GO:0051235 | maintenance of location | 69/2334 | 335/29008 | 3.24E-13 | 1.90E-11 | 1.07E-11 |
| **503** | KO | GO:0031589 | cell-substrate adhesion | 73/2334 | 366/29008 | 3.62E-13 | 2.10E-11 | 1.18E-11 |
| **504** | KO | GO:0001818 | negative regulation of cytokine production | 63/2334 | 292/29008 | 3.95E-13 | 2.27E-11 | 1.28E-11 |
| **505** | KO | GO:0015711 | organic anion transport | 71/2334 | 352/29008 | 4.31E-13 | 2.42E-11 | 1.36E-11 |
| **506** | KO | GO:0018108 | peptidyl-tyrosine phosphorylation | 67/2334 | 322/29008 | 4.33E-13 | 2.42E-11 | 1.36E-11 |
| **507** | KO | GO:0008202 | steroid metabolic process | 69/2334 | 337/29008 | 4.36E-13 | 2.42E-11 | 1.36E-11 |
| **508** | KO | GO:0022604 | regulation of cell morphogenesis | 69/2334 | 337/29008 | 4.36E-13 | 2.42E-11 | 1.36E-11 |
| **509** | KO | GO:0032728 | positive regulation of interferon-beta production | 23/2334 | 49/29008 | 4.52E-13 | 2.48E-11 | 1.39E-11 |
| **510** | KO | GO:0062208 | positive regulation of pattern recognition receptor signaling pathway | 22/2334 | 45/29008 | 5.08E-13 | 2.76E-11 | 1.56E-11 |
| **511** | KO | GO:0006820 | anion transport | 89/2334 | 495/29008 | 5.18E-13 | 2.79E-11 | 1.57E-11 |
| **512** | KO | GO:0002253 | activation of immune response | 82/2334 | 439/29008 | 5.33E-13 | 2.85E-11 | 1.60E-11 |
| **513** | KO | GO:0046394 | carboxylic acid biosynthetic process | 63/2334 | 295/29008 | 6.40E-13 | 3.39E-11 | 1.91E-11 |
| **514** | KO | GO:0006163 | purine nucleotide metabolic process | 73/2334 | 373/29008 | 9.61E-13 | 5.05E-11 | 2.84E-11 |
| **515** | KO | GO:0010563 | negative regulation of phosphorus metabolic process | 83/2334 | 453/29008 | 1.12E-12 | 5.80E-11 | 3.26E-11 |
| **516** | KO | GO:0045936 | negative regulation of phosphate metabolic process | 83/2334 | 453/29008 | 1.12E-12 | 5.80E-11 | 3.26E-11 |
| **517** | KO | GO:0046631 | alpha-beta T cell activation | 46/2334 | 180/29008 | 1.15E-12 | 5.90E-11 | 3.32E-11 |
| **518** | KO | GO:0046486 | glycerolipid metabolic process | 73/2334 | 377/29008 | 1.65E-12 | 8.39E-11 | 4.72E-11 |
| **519** | KO | GO:0030098 | lymphocyte differentiation | 81/2334 | 441/29008 | 1.84E-12 | 9.26E-11 | 5.21E-11 |
| **520** | KO | GO:0002718 | regulation of cytokine production involved in immune response | 32/2334 | 97/29008 | 1.91E-12 | 9.54E-11 | 5.37E-11 |
| **521** | KO | GO:0009150 | purine ribonucleotide metabolic process | 69/2334 | 348/29008 | 2.13E-12 | 1.05E-10 | 5.93E-11 |
| **522** | KO | GO:0046942 | carboxylic acid transport | 61/2334 | 288/29008 | 2.15E-12 | 1.05E-10 | 5.93E-11 |
| **523** | KO | GO:0019216 | regulation of lipid metabolic process | 73/2334 | 379/29008 | 2.16E-12 | 1.05E-10 | 5.93E-11 |
| **524** | KO | GO:0050729 | positive regulation of inflammatory response | 38/2334 | 134/29008 | 3.39E-12 | 1.64E-10 | 9.23E-11 |
| **525** | KO | GO:0009991 | response to extracellular stimulus | 74/2334 | 391/29008 | 3.73E-12 | 1.79E-10 | 1.01E-10 |
| **526** | KO | GO:0045071 | negative regulation of viral genome replication | 24/2334 | 58/29008 | 4.19E-12 | 1.99E-10 | 1.12E-10 |
| **527** | KO | GO:0019395 | fatty acid oxidation | 34/2334 | 112/29008 | 5.64E-12 | 2.66E-10 | 1.50E-10 |
| **528** | KO | GO:0071675 | regulation of mononuclear cell migration | 35/2334 | 118/29008 | 5.81E-12 | 2.73E-10 | 1.53E-10 |
| **529** | KO | GO:0034440 | lipid oxidation | 35/2334 | 119/29008 | 7.60E-12 | 3.54E-10 | 1.99E-10 |
| **530** | KO | GO:0051091 | positive regulation of DNA-binding transcription factor activity | 57/2334 | 267/29008 | 8.05E-12 | 3.72E-10 | 2.09E-10 |
| **531** | KO | GO:0042129 | regulation of T cell proliferation | 45/2334 | 183/29008 | 8.48E-12 | 3.89E-10 | 2.19E-10 |
| **532** | KO | GO:0071219 | cellular response to molecule of bacterial origin | 63/2334 | 313/29008 | 9.77E-12 | 4.45E-10 | 2.50E-10 |
| **533** | KO | GO:0071216 | cellular response to biotic stimulus | 66/2334 | 337/29008 | 1.13E-11 | 5.12E-10 | 2.88E-10 |
| **534** | KO | GO:0032970 | regulation of actin filament-based process | 76/2334 | 416/29008 | 1.16E-11 | 5.20E-10 | 2.93E-10 |
| **535** | KO | GO:0072329 | monocarboxylic acid catabolic process | 35/2334 | 121/29008 | 1.29E-11 | 5.73E-10 | 3.22E-10 |
| **536** | KO | GO:0050777 | negative regulation of immune response | 48/2334 | 206/29008 | 1.39E-11 | 6.12E-10 | 3.45E-10 |
| **537** | KO | GO:2001233 | regulation of apoptotic signaling pathway | 74/2334 | 402/29008 | 1.49E-11 | 6.53E-10 | 3.68E-10 |
| **538** | KO | GO:0062207 | regulation of pattern recognition receptor signaling pathway | 31/2334 | 99/29008 | 1.96E-11 | 8.54E-10 | 4.80E-10 |
| **539** | KO | GO:0032609 | interferon-gamma production | 37/2334 | 135/29008 | 1.97E-11 | 8.54E-10 | 4.80E-10 |
| **540** | KO | GO:0050730 | regulation of peptidyl-tyrosine phosphorylation | 56/2334 | 266/29008 | 2.19E-11 | 9.39E-10 | 5.29E-10 |
| **541** | KO | GO:1901361 | organic cyclic compound catabolic process | 81/2334 | 463/29008 | 2.40E-11 | 1.02E-09 | 5.77E-10 |
| **542** | KO | GO:1903706 | regulation of hemopoiesis | 73/2334 | 398/29008 | 2.43E-11 | 1.03E-09 | 5.80E-10 |
| **543** | KO | GO:1990845 | adaptive thermogenesis | 41/2334 | 162/29008 | 2.66E-11 | 1.12E-09 | 6.30E-10 |
| **544** | KO | GO:0031668 | cellular response to extracellular stimulus | 51/2334 | 231/29008 | 2.73E-11 | 1.14E-09 | 6.42E-10 |
| **545** | KO | GO:0071346 | cellular response to interferon-gamma | 33/2334 | 112/29008 | 2.83E-11 | 1.18E-09 | 6.62E-10 |
| **546** | KO | GO:0070486 | leukocyte aggregation | 13/2334 | 18/29008 | 3.35E-11 | 1.37E-09 | 7.73E-10 |
| **547** | KO | GO:0006801 | superoxide metabolic process | 25/2334 | 68/29008 | 3.35E-11 | 1.37E-09 | 7.73E-10 |
| **548** | KO | GO:0030217 | T cell differentiation | 60/2334 | 299/29008 | 3.44E-11 | 1.40E-09 | 7.88E-10 |
| **549** | KO | GO:0002702 | positive regulation of production of molecular mediator of immune response | 35/2334 | 125/29008 | 3.54E-11 | 1.43E-09 | 8.06E-10 |
| **550** | KO | GO:0033674 | positive regulation of kinase activity | 77/2334 | 435/29008 | 4.17E-11 | 1.67E-09 | 9.42E-10 |
| **551** | KO | GO:0008203 | cholesterol metabolic process | 38/2334 | 145/29008 | 4.59E-11 | 1.82E-09 | 1.02E-09 |
| **552** | KO | GO:0032946 | positive regulation of mononuclear cell proliferation | 38/2334 | 145/29008 | 4.59E-11 | 1.82E-09 | 1.02E-09 |
| **553** | KO | GO:0071677 | positive regulation of mononuclear cell migration | 25/2334 | 69/29008 | 4.86E-11 | 1.91E-09 | 1.08E-09 |
| **554** | KO | GO:1901652 | response to peptide | 74/2334 | 412/29008 | 4.94E-11 | 1.93E-09 | 1.09E-09 |
| **555** | KO | GO:0042326 | negative regulation of phosphorylation | 72/2334 | 396/29008 | 5.04E-11 | 1.96E-09 | 1.10E-09 |
| **556** | KO | GO:0070555 | response to interleukin-1 | 30/2334 | 97/29008 | 5.83E-11 | 2.25E-09 | 1.27E-09 |
| **557** | KO | GO:0071222 | cellular response to lipopolysaccharide | 60/2334 | 303/29008 | 6.06E-11 | 2.33E-09 | 1.31E-09 |
| **558** | KO | GO:0002819 | regulation of adaptive immune response | 51/2334 | 236/29008 | 6.24E-11 | 2.38E-09 | 1.34E-09 |
| **559** | KO | GO:0120254 | olefinic compound metabolic process | 40/2334 | 160/29008 | 6.94E-11 | 2.63E-09 | 1.48E-09 |
| **560** | KO | GO:0032956 | regulation of actin cytoskeleton organization | 68/2334 | 368/29008 | 8.30E-11 | 3.13E-09 | 1.76E-09 |
| **561** | KO | GO:0002218 | activation of innate immune response | 21/2334 | 51/29008 | 1.02E-10 | 3.83E-09 | 2.16E-09 |
| **562** | KO | GO:0001774 | microglial cell activation | 19/2334 | 42/29008 | 1.10E-10 | 4.06E-09 | 2.29E-09 |
| **563** | KO | GO:0036230 | granulocyte activation | 19/2334 | 42/29008 | 1.10E-10 | 4.06E-09 | 2.29E-09 |
| **564** | KO | GO:0031663 | lipopolysaccharide-mediated signaling pathway | 23/2334 | 61/29008 | 1.12E-10 | 4.14E-09 | 2.33E-09 |
| **565** | KO | GO:0006637 | acyl-CoA metabolic process | 28/2334 | 88/29008 | 1.19E-10 | 4.34E-09 | 2.44E-09 |
| **566** | KO | GO:0035383 | thioester metabolic process | 28/2334 | 88/29008 | 1.19E-10 | 4.34E-09 | 2.44E-09 |
| **567** | KO | GO:0006091 | generation of precursor metabolites and energy | 75/2334 | 428/29008 | 1.21E-10 | 4.34E-09 | 2.44E-09 |
| **568** | KO | GO:0070997 | neuron death | 75/2334 | 428/29008 | 1.21E-10 | 4.34E-09 | 2.44E-09 |
| **569** | KO | GO:0009062 | fatty acid catabolic process | 30/2334 | 100/29008 | 1.36E-10 | 4.84E-09 | 2.73E-09 |
| **570** | KO | GO:0034123 | positive regulation of toll-like receptor signaling pathway | 16/2334 | 30/29008 | 1.43E-10 | 5.09E-09 | 2.87E-09 |
| **571** | KO | GO:1902652 | secondary alcohol metabolic process | 39/2334 | 157/29008 | 1.47E-10 | 5.18E-09 | 2.92E-09 |
| **572** | KO | GO:0040013 | negative regulation of locomotion | 64/2334 | 341/29008 | 1.51E-10 | 5.31E-09 | 2.99E-09 |
| **573** | KO | GO:0045860 | positive regulation of protein kinase activity | 66/2334 | 358/29008 | 1.73E-10 | 6.05E-09 | 3.41E-09 |
| **574** | KO | GO:0006909 | phagocytosis | 69/2334 | 383/29008 | 1.92E-10 | 6.67E-09 | 3.75E-09 |
| **575** | KO | GO:0019079 | viral genome replication | 34/2334 | 126/29008 | 2.01E-10 | 6.95E-09 | 3.91E-09 |
| **576** | KO | GO:0002695 | negative regulation of leukocyte activation | 45/2334 | 200/29008 | 2.04E-10 | 7.00E-09 | 3.94E-09 |
| **577** | KO | GO:0045069 | regulation of viral genome replication | 28/2334 | 90/29008 | 2.16E-10 | 7.36E-09 | 4.14E-09 |
| **578** | KO | GO:0007264 | small GTPase mediated signal transduction | 74/2334 | 425/29008 | 2.17E-10 | 7.36E-09 | 4.14E-09 |
| **579** | KO | GO:0072330 | monocarboxylic acid biosynthetic process | 46/2334 | 208/29008 | 2.37E-10 | 8.00E-09 | 4.50E-09 |
| **580** | KO | GO:0035456 | response to interferon-beta | 23/2334 | 63/29008 | 2.40E-10 | 8.07E-09 | 4.54E-09 |
| **581** | KO | GO:0050767 | regulation of neurogenesis | 77/2334 | 451/29008 | 2.43E-10 | 8.11E-09 | 4.57E-09 |
| **582** | KO | GO:0002755 | MyD88-dependent toll-like receptor signaling pathway | 13/2334 | 20/29008 | 2.59E-10 | 8.62E-09 | 4.85E-09 |
| **583** | KO | GO:0042593 | glucose homeostasis | 56/2334 | 283/29008 | 2.68E-10 | 8.85E-09 | 4.98E-09 |
| **584** | KO | GO:0033500 | carbohydrate homeostasis | 56/2334 | 284/29008 | 3.08E-10 | 1.01E-08 | 5.69E-09 |
| **585** | KO | GO:0016125 | sterol metabolic process | 38/2334 | 154/29008 | 3.10E-10 | 1.01E-08 | 5.70E-09 |
| **586** | KO | GO:0048872 | homeostasis of number of cells | 66/2334 | 363/29008 | 3.18E-10 | 1.03E-08 | 5.81E-09 |
| **587** | KO | GO:0008654 | phospholipid biosynthetic process | 47/2334 | 217/29008 | 3.19E-10 | 1.03E-08 | 5.81E-09 |
| **588** | KO | GO:0071347 | cellular response to interleukin-1 | 26/2334 | 80/29008 | 3.23E-10 | 1.04E-08 | 5.84E-09 |
| **589** | KO | GO:0019439 | aromatic compound catabolic process | 75/2334 | 437/29008 | 3.25E-10 | 1.04E-08 | 5.85E-09 |
| **590** | KO | GO:0007160 | cell-matrix adhesion | 48/2334 | 225/29008 | 3.59E-10 | 1.14E-08 | 6.42E-09 |
| **591** | KO | GO:0009410 | response to xenobiotic stimulus | 61/2334 | 325/29008 | 4.03E-10 | 1.28E-08 | 7.19E-09 |
| **592** | KO | GO:0001933 | negative regulation of protein phosphorylation | 64/2334 | 349/29008 | 4.09E-10 | 1.29E-08 | 7.25E-09 |
| **593** | KO | GO:0002705 | positive regulation of leukocyte mediated immunity | 42/2334 | 183/29008 | 4.22E-10 | 1.31E-08 | 7.37E-09 |
| **594** | KO | GO:0045834 | positive regulation of lipid metabolic process | 42/2334 | 183/29008 | 4.22E-10 | 1.31E-08 | 7.37E-09 |
| **595** | KO | GO:1905952 | regulation of lipid localization | 42/2334 | 183/29008 | 4.22E-10 | 1.31E-08 | 7.37E-09 |
| **596** | KO | GO:0006520 | cellular amino acid metabolic process | 52/2334 | 256/29008 | 4.36E-10 | 1.34E-08 | 7.54E-09 |
| **597** | KO | GO:0032649 | regulation of interferon-gamma production | 33/2334 | 123/29008 | 4.36E-10 | 1.34E-08 | 7.54E-09 |
| **598** | KO | GO:0006979 | response to oxidative stress | 71/2334 | 407/29008 | 4.60E-10 | 1.41E-08 | 7.92E-09 |
| **599** | KO | GO:0050671 | positive regulation of lymphocyte proliferation | 36/2334 | 143/29008 | 4.96E-10 | 1.50E-08 | 8.44E-09 |
| **600** | KO | GO:0002720 | positive regulation of cytokine production involved in immune response | 23/2334 | 65/29008 | 4.96E-10 | 1.50E-08 | 8.44E-09 |
| **601** | KO | GO:1901605 | alpha-amino acid metabolic process | 41/2334 | 177/29008 | 5.04E-10 | 1.52E-08 | 8.54E-09 |
| **602** | KO | GO:1901214 | regulation of neuron death | 68/2334 | 384/29008 | 5.55E-10 | 1.66E-08 | 9.36E-09 |
| **603** | KO | GO:0070665 | positive regulation of leukocyte proliferation | 38/2334 | 157/29008 | 5.63E-10 | 1.68E-08 | 9.45E-09 |
| **604** | KO | GO:1902105 | regulation of leukocyte differentiation | 60/2334 | 320/29008 | 5.83E-10 | 1.73E-08 | 9.75E-09 |
| **605** | KO | GO:0042063 | gliogenesis | 63/2334 | 344/29008 | 5.90E-10 | 1.74E-08 | 9.82E-09 |
| **606** | KO | GO:0030099 | myeloid cell differentiation | 73/2334 | 426/29008 | 5.98E-10 | 1.76E-08 | 9.89E-09 |
| **607** | KO | GO:0034121 | regulation of toll-like receptor signaling pathway | 24/2334 | 71/29008 | 6.17E-10 | 1.81E-08 | 1.02E-08 |
| **608** | KO | GO:0001667 | ameboidal-type cell migration | 75/2334 | 444/29008 | 6.81E-10 | 1.98E-08 | 1.12E-08 |
| **609** | KO | GO:0007346 | regulation of mitotic cell cycle | 77/2334 | 462/29008 | 7.63E-10 | 2.21E-08 | 1.24E-08 |
| **610** | KO | GO:0045444 | fat cell differentiation | 52/2334 | 260/29008 | 7.76E-10 | 2.24E-08 | 1.26E-08 |
| **611** | KO | GO:0051092 | positive regulation of NF-kappaB transcription factor activity | 37/2334 | 152/29008 | 7.96E-10 | 2.28E-08 | 1.29E-08 |
| **612** | KO | GO:0050866 | negative regulation of cell activation | 47/2334 | 223/29008 | 8.32E-10 | 2.38E-08 | 1.34E-08 |
| **613** | KO | GO:0002460 | adaptive immune response based on somatic recombination of immune receptors built from immunoglobulin superfamily domains | 79/2334 | 480/29008 | 8.41E-10 | 2.38E-08 | 1.34E-08 |
| **614** | KO | GO:0072676 | lymphocyte migration | 30/2334 | 107/29008 | 8.42E-10 | 2.38E-08 | 1.34E-08 |
| **615** | KO | GO:0042180 | cellular ketone metabolic process | 49/2334 | 238/29008 | 8.51E-10 | 2.40E-08 | 1.35E-08 |
| **616** | KO | GO:0019882 | antigen processing and presentation | 33/2334 | 126/29008 | 8.61E-10 | 2.41E-08 | 1.36E-08 |
| **617** | KO | GO:1901653 | cellular response to peptide | 58/2334 | 308/29008 | 9.47E-10 | 2.64E-08 | 1.49E-08 |
| **618** | KO | GO:0001659 | temperature homeostasis | 42/2334 | 188/29008 | 1.02E-09 | 2.83E-08 | 1.59E-08 |
| **619** | KO | GO:0050766 | positive regulation of phagocytosis | 26/2334 | 84/29008 | 1.07E-09 | 2.96E-08 | 1.66E-08 |
| **620** | KO | GO:1901136 | carbohydrate derivative catabolic process | 36/2334 | 147/29008 | 1.12E-09 | 3.09E-08 | 1.74E-08 |
| **621** | KO | GO:0046434 | organophosphate catabolic process | 37/2334 | 154/29008 | 1.18E-09 | 3.23E-08 | 1.82E-08 |
| **622** | KO | GO:0006694 | steroid biosynthetic process | 39/2334 | 168/29008 | 1.23E-09 | 3.34E-08 | 1.88E-08 |
| **623** | KO | GO:0051250 | negative regulation of lymphocyte activation | 39/2334 | 168/29008 | 1.23E-09 | 3.34E-08 | 1.88E-08 |
| **624** | KO | GO:0055088 | lipid homeostasis | 39/2334 | 168/29008 | 1.23E-09 | 3.34E-08 | 1.88E-08 |
| **625** | KO | GO:0016052 | carbohydrate catabolic process | 35/2334 | 141/29008 | 1.29E-09 | 3.48E-08 | 1.96E-08 |
| **626** | KO | GO:0010810 | regulation of cell-substrate adhesion | 47/2334 | 226/29008 | 1.32E-09 | 3.54E-08 | 1.99E-08 |
| **627** | KO | GO:0002285 | lymphocyte activation involved in immune response | 46/2334 | 219/29008 | 1.41E-09 | 3.76E-08 | 2.11E-08 |
| **628** | KO | GO:0001894 | tissue homeostasis | 54/2334 | 280/29008 | 1.46E-09 | 3.90E-08 | 2.19E-08 |
| **629** | KO | GO:0032655 | regulation of interleukin-12 production | 22/2334 | 63/29008 | 1.57E-09 | 4.16E-08 | 2.34E-08 |
| **630** | KO | GO:0038061 | NIK/NF-kappaB signaling | 31/2334 | 116/29008 | 1.62E-09 | 4.27E-08 | 2.40E-08 |
| **631** | KO | GO:0007249 | I-kappaB kinase/NF-kappaB signaling | 47/2334 | 228/29008 | 1.79E-09 | 4.69E-08 | 2.64E-08 |
| **632** | KO | GO:0002690 | positive regulation of leukocyte chemotaxis | 28/2334 | 98/29008 | 1.91E-09 | 4.98E-08 | 2.80E-08 |
| **633** | KO | GO:0031348 | negative regulation of defense response | 48/2334 | 236/29008 | 1.92E-09 | 5.01E-08 | 2.82E-08 |
| **634** | KO | GO:0060249 | anatomical structure homeostasis | 59/2334 | 322/29008 | 2.01E-09 | 5.22E-08 | 2.94E-08 |
| **635** | KO | GO:0002698 | negative regulation of immune effector process | 33/2334 | 130/29008 | 2.05E-09 | 5.30E-08 | 2.98E-08 |
| **636** | KO | GO:0051271 | negative regulation of cellular component movement | 58/2334 | 315/29008 | 2.28E-09 | 5.85E-08 | 3.29E-08 |
| **637** | KO | GO:0002688 | regulation of leukocyte chemotaxis | 32/2334 | 124/29008 | 2.30E-09 | 5.89E-08 | 3.31E-08 |
| **638** | KO | GO:0043903 | regulation of biological process involved in symbiotic interaction | 25/2334 | 81/29008 | 2.38E-09 | 6.06E-08 | 3.41E-08 |
| **639** | KO | GO:0044270 | cellular nitrogen compound catabolic process | 70/2334 | 414/29008 | 2.40E-09 | 6.09E-08 | 3.43E-08 |
| **640** | KO | GO:0046700 | heterocycle catabolic process | 70/2334 | 415/29008 | 2.66E-09 | 6.73E-08 | 3.79E-08 |
| **641** | KO | GO:0002456 | T cell mediated immunity | 35/2334 | 145/29008 | 2.87E-09 | 7.19E-08 | 4.05E-08 |
| **642** | KO | GO:0120161 | regulation of cold-induced thermogenesis | 35/2334 | 145/29008 | 2.87E-09 | 7.19E-08 | 4.05E-08 |
| **643** | KO | GO:0032615 | interleukin-12 production | 22/2334 | 65/29008 | 3.09E-09 | 7.72E-08 | 4.34E-08 |
| **644** | KO | GO:0031331 | positive regulation of cellular catabolic process | 71/2334 | 425/29008 | 3.13E-09 | 7.78E-08 | 4.38E-08 |
| **645** | KO | GO:0043254 | regulation of protein-containing complex assembly | 74/2334 | 451/29008 | 3.28E-09 | 8.11E-08 | 4.57E-08 |
| **646** | KO | GO:0034599 | cellular response to oxidative stress | 52/2334 | 271/29008 | 3.51E-09 | 8.67E-08 | 4.88E-08 |
| **647** | KO | GO:0002262 | myeloid cell homeostasis | 42/2334 | 196/29008 | 3.84E-09 | 9.44E-08 | 5.31E-08 |
| **648** | KO | GO:0042554 | superoxide anion generation | 18/2334 | 45/29008 | 3.88E-09 | 9.49E-08 | 5.34E-08 |
| **649** | KO | GO:0106106 | cold-induced thermogenesis | 35/2334 | 147/29008 | 4.22E-09 | 1.03E-07 | 5.79E-08 |
| **650** | KO | GO:0042119 | neutrophil activation | 16/2334 | 36/29008 | 4.52E-09 | 1.10E-07 | 6.17E-08 |
| **651** | KO | GO:0098586 | cellular response to virus | 23/2334 | 72/29008 | 4.89E-09 | 1.18E-07 | 6.66E-08 |
| **652** | KO | GO:2000146 | negative regulation of cell motility | 56/2334 | 306/29008 | 5.36E-09 | 1.29E-07 | 7.24E-08 |
| **653** | KO | GO:0001952 | regulation of cell-matrix adhesion | 32/2334 | 128/29008 | 5.37E-09 | 1.29E-07 | 7.24E-08 |
| **654** | KO | GO:0007229 | integrin-mediated signaling pathway | 27/2334 | 96/29008 | 5.38E-09 | 1.29E-07 | 7.24E-08 |
| **655** | KO | GO:0032729 | positive regulation of interferon-gamma production | 25/2334 | 84/29008 | 5.50E-09 | 1.31E-07 | 7.37E-08 |
| **656** | KO | GO:0052372 | modulation by symbiont of entry into host | 20/2334 | 56/29008 | 5.52E-09 | 1.31E-07 | 7.37E-08 |
| **657** | KO | GO:0046596 | regulation of viral entry into host cell | 19/2334 | 51/29008 | 5.87E-09 | 1.39E-07 | 7.81E-08 |
| **658** | KO | GO:0044262 | cellular carbohydrate metabolic process | 54/2334 | 291/29008 | 6.05E-09 | 1.42E-07 | 8.02E-08 |
| **659** | KO | GO:0015748 | organophosphate ester transport | 31/2334 | 122/29008 | 6.09E-09 | 1.43E-07 | 8.04E-08 |
| **660** | KO | GO:0050920 | regulation of chemotaxis | 46/2334 | 229/29008 | 6.25E-09 | 1.46E-07 | 8.22E-08 |
| **661** | KO | GO:0009132 | nucleoside diphosphate metabolic process | 32/2334 | 129/29008 | 6.60E-09 | 1.54E-07 | 8.64E-08 |
| **662** | KO | GO:0033865 | nucleoside bisphosphate metabolic process | 29/2334 | 110/29008 | 7.54E-09 | 1.74E-07 | 9.77E-08 |
| **663** | KO | GO:0033875 | ribonucleoside bisphosphate metabolic process | 29/2334 | 110/29008 | 7.54E-09 | 1.74E-07 | 9.77E-08 |
| **664** | KO | GO:0034032 | purine nucleoside bisphosphate metabolic process | 29/2334 | 110/29008 | 7.54E-09 | 1.74E-07 | 9.77E-08 |
| **665** | KO | GO:0030336 | negative regulation of cell migration | 54/2334 | 293/29008 | 7.75E-09 | 1.78E-07 | 1.00E-07 |
| **666** | KO | GO:0070372 | regulation of ERK1 and ERK2 cascade | 58/2334 | 326/29008 | 8.42E-09 | 1.92E-07 | 1.08E-07 |
| **667** | KO | GO:0014812 | muscle cell migration | 30/2334 | 117/29008 | 8.47E-09 | 1.93E-07 | 1.09E-07 |
| **668** | KO | GO:0045123 | cellular extravasation | 24/2334 | 80/29008 | 9.28E-09 | 2.10E-07 | 1.18E-07 |
| **669** | KO | GO:0046890 | regulation of lipid biosynthetic process | 42/2334 | 202/29008 | 9.81E-09 | 2.22E-07 | 1.25E-07 |
| **670** | KO | GO:0007204 | positive regulation of cytosolic calcium ion concentration | 60/2334 | 344/29008 | 1.01E-08 | 2.27E-07 | 1.28E-07 |
| **671** | KO | GO:1902107 | positive regulation of leukocyte differentiation | 40/2334 | 188/29008 | 1.11E-08 | 2.47E-07 | 1.39E-07 |
| **672** | KO | GO:1903708 | positive regulation of hemopoiesis | 40/2334 | 188/29008 | 1.11E-08 | 2.47E-07 | 1.39E-07 |
| **673** | KO | GO:0060759 | regulation of response to cytokine stimulus | 32/2334 | 132/29008 | 1.20E-08 | 2.68E-07 | 1.51E-07 |
| **674** | KO | GO:0006650 | glycerophospholipid metabolic process | 51/2334 | 273/29008 | 1.26E-08 | 2.79E-07 | 1.57E-07 |
| **675** | KO | GO:0046889 | positive regulation of lipid biosynthetic process | 28/2334 | 106/29008 | 1.30E-08 | 2.87E-07 | 1.61E-07 |
| **676** | KO | GO:0009185 | ribonucleoside diphosphate metabolic process | 29/2334 | 113/29008 | 1.46E-08 | 3.22E-07 | 1.81E-07 |
| **677** | KO | GO:0014910 | regulation of smooth muscle cell migration | 26/2334 | 94/29008 | 1.50E-08 | 3.30E-07 | 1.86E-07 |
| **678** | KO | GO:0032677 | regulation of interleukin-8 production | 23/2334 | 76/29008 | 1.56E-08 | 3.42E-07 | 1.92E-07 |
| **679** | KO | GO:0048511 | rhythmic process | 55/2334 | 307/29008 | 1.58E-08 | 3.44E-07 | 1.94E-07 |
| **680** | KO | GO:0045621 | positive regulation of lymphocyte differentiation | 30/2334 | 120/29008 | 1.60E-08 | 3.46E-07 | 1.94E-07 |
| **681** | KO | GO:0009896 | positive regulation of catabolic process | 77/2334 | 494/29008 | 1.60E-08 | 3.46E-07 | 1.94E-07 |
| **682** | KO | GO:0062197 | cellular response to chemical stress | 58/2334 | 333/29008 | 1.86E-08 | 4.00E-07 | 2.25E-07 |
| **683** | KO | GO:0031667 | response to nutrient levels | 61/2334 | 358/29008 | 1.87E-08 | 4.02E-07 | 2.26E-07 |
| **684** | KO | GO:0061082 | myeloid leukocyte cytokine production | 14/2334 | 30/29008 | 1.94E-08 | 4.15E-07 | 2.33E-07 |
| **685** | KO | GO:0051222 | positive regulation of protein transport | 56/2334 | 317/29008 | 1.95E-08 | 4.17E-07 | 2.34E-07 |
| **686** | KO | GO:0015718 | monocarboxylic acid transport | 30/2334 | 121/29008 | 1.96E-08 | 4.17E-07 | 2.34E-07 |
| **687** | KO | GO:0051402 | neuron apoptotic process | 55/2334 | 309/29008 | 1.99E-08 | 4.22E-07 | 2.38E-07 |
| **688** | KO | GO:0007623 | circadian rhythm | 44/2334 | 222/29008 | 2.01E-08 | 4.25E-07 | 2.39E-07 |
| **689** | KO | GO:0051258 | protein polymerization | 53/2334 | 293/29008 | 2.05E-08 | 4.31E-07 | 2.43E-07 |
| **690** | KO | GO:0032637 | interleukin-8 production | 23/2334 | 77/29008 | 2.06E-08 | 4.32E-07 | 2.43E-07 |
| **691** | KO | GO:0043087 | regulation of GTPase activity | 57/2334 | 326/29008 | 2.13E-08 | 4.45E-07 | 2.50E-07 |
| **692** | KO | GO:0051251 | positive regulation of lymphocyte activation | 70/2334 | 436/29008 | 2.13E-08 | 4.45E-07 | 2.50E-07 |
| **693** | KO | GO:0042886 | amide transport | 59/2334 | 343/29008 | 2.24E-08 | 4.65E-07 | 2.62E-07 |
| **694** | KO | GO:1901222 | regulation of NIK/NF-kappaB signaling | 26/2334 | 96/29008 | 2.42E-08 | 5.02E-07 | 2.82E-07 |
| **695** | KO | GO:0051480 | regulation of cytosolic calcium ion concentration | 64/2334 | 386/29008 | 2.47E-08 | 5.09E-07 | 2.87E-07 |
| **696** | KO | GO:0006084 | acetyl-CoA metabolic process | 15/2334 | 35/29008 | 2.53E-08 | 5.19E-07 | 2.92E-07 |
| **697** | KO | GO:2001235 | positive regulation of apoptotic signaling pathway | 35/2334 | 157/29008 | 2.58E-08 | 5.29E-07 | 2.98E-07 |
| **698** | KO | GO:0071496 | cellular response to external stimulus | 53/2334 | 295/29008 | 2.59E-08 | 5.30E-07 | 2.98E-07 |
| **699** | KO | GO:0014909 | smooth muscle cell migration | 27/2334 | 103/29008 | 2.80E-08 | 5.69E-07 | 3.20E-07 |
| **700** | KO | GO:0051259 | protein complex oligomerization | 46/2334 | 240/29008 | 2.85E-08 | 5.77E-07 | 3.25E-07 |
| **701** | KO | GO:0019318 | hexose metabolic process | 48/2334 | 256/29008 | 2.95E-08 | 5.97E-07 | 3.36E-07 |
| **702** | KO | GO:0002440 | production of molecular mediator of immune response | 63/2334 | 380/29008 | 3.18E-08 | 6.40E-07 | 3.60E-07 |
| **703** | KO | GO:0032607 | interferon-alpha production | 14/2334 | 31/29008 | 3.27E-08 | 6.57E-07 | 3.70E-07 |
| **704** | KO | GO:0005996 | monosaccharide metabolic process | 50/2334 | 273/29008 | 3.37E-08 | 6.75E-07 | 3.80E-07 |
| **705** | KO | GO:0042102 | positive regulation of T cell proliferation | 27/2334 | 104/29008 | 3.49E-08 | 6.96E-07 | 3.92E-07 |
| **706** | KO | GO:0062013 | positive regulation of small molecule metabolic process | 36/2334 | 166/29008 | 3.50E-08 | 6.96E-07 | 3.92E-07 |
| **707** | KO | GO:1901657 | glycosyl compound metabolic process | 23/2334 | 79/29008 | 3.52E-08 | 6.97E-07 | 3.92E-07 |
| **708** | KO | GO:0002449 | lymphocyte mediated immunity | 74/2334 | 477/29008 | 3.69E-08 | 7.28E-07 | 4.10E-07 |
| **709** | KO | GO:0050868 | negative regulation of T cell activation | 31/2334 | 131/29008 | 3.70E-08 | 7.28E-07 | 4.10E-07 |
| **710** | KO | GO:0097193 | intrinsic apoptotic signaling pathway | 54/2334 | 307/29008 | 4.04E-08 | 7.92E-07 | 4.46E-07 |
| **711** | KO | GO:0070371 | ERK1 and ERK2 cascade | 59/2334 | 349/29008 | 4.24E-08 | 8.29E-07 | 4.67E-07 |
| **712** | KO | GO:0009135 | purine nucleoside diphosphate metabolic process | 27/2334 | 105/29008 | 4.34E-08 | 8.44E-07 | 4.75E-07 |
| **713** | KO | GO:0009179 | purine ribonucleoside diphosphate metabolic process | 27/2334 | 105/29008 | 4.34E-08 | 8.44E-07 | 4.75E-07 |
| **714** | KO | GO:0010565 | regulation of cellular ketone metabolic process | 34/2334 | 153/29008 | 4.41E-08 | 8.49E-07 | 4.78E-07 |
| **715** | KO | GO:2001251 | negative regulation of chromosome organization | 24/2334 | 86/29008 | 4.42E-08 | 8.49E-07 | 4.78E-07 |
| **716** | KO | GO:0002822 | regulation of adaptive immune response based on somatic recombination of immune receptors built from immunoglobulin superfamily domains | 43/2334 | 220/29008 | 4.42E-08 | 8.49E-07 | 4.78E-07 |
| **717** | KO | GO:0051651 | maintenance of location in cell | 43/2334 | 220/29008 | 4.42E-08 | 8.49E-07 | 4.78E-07 |
| **718** | KO | GO:1904951 | positive regulation of establishment of protein localization | 57/2334 | 333/29008 | 4.59E-08 | 8.78E-07 | 4.94E-07 |
| **719** | KO | GO:0032611 | interleukin-1 beta production | 26/2334 | 99/29008 | 4.82E-08 | 9.18E-07 | 5.17E-07 |
| **720** | KO | GO:2001236 | regulation of extrinsic apoptotic signaling pathway | 36/2334 | 168/29008 | 4.84E-08 | 9.18E-07 | 5.17E-07 |
| **721** | KO | GO:0043523 | regulation of neuron apoptotic process | 50/2334 | 276/29008 | 4.84E-08 | 9.18E-07 | 5.17E-07 |
| **722** | KO | GO:0006874 | cellular calcium ion homeostasis | 75/2334 | 490/29008 | 5.28E-08 | 9.97E-07 | 5.61E-07 |
| **723** | KO | GO:0022408 | negative regulation of cell-cell adhesion | 41/2334 | 206/29008 | 5.34E-08 | 1.01E-06 | 5.66E-07 |
| **724** | KO | GO:0014911 | positive regulation of smooth muscle cell migration | 20/2334 | 63/29008 | 5.47E-08 | 1.02E-06 | 5.75E-07 |
| **725** | KO | GO:0072678 | T cell migration | 20/2334 | 63/29008 | 5.47E-08 | 1.02E-06 | 5.75E-07 |
| **726** | KO | GO:2000401 | regulation of lymphocyte migration | 20/2334 | 63/29008 | 5.47E-08 | 1.02E-06 | 5.75E-07 |
| **727** | KO | GO:0015908 | fatty acid transport | 24/2334 | 87/29008 | 5.64E-08 | 1.05E-06 | 5.91E-07 |
| **728** | KO | GO:0034655 | nucleobase-containing compound catabolic process | 63/2334 | 386/29008 | 5.76E-08 | 1.07E-06 | 6.01E-07 |
| **729** | KO | GO:0045619 | regulation of lymphocyte differentiation | 40/2334 | 199/29008 | 5.82E-08 | 1.08E-06 | 6.06E-07 |
| **730** | KO | GO:0006635 | fatty acid beta-oxidation | 22/2334 | 75/29008 | 5.98E-08 | 1.10E-06 | 6.21E-07 |
| **731** | KO | GO:0006090 | pyruvate metabolic process | 29/2334 | 120/29008 | 6.16E-08 | 1.13E-06 | 6.36E-07 |
| **732** | KO | GO:0032612 | interleukin-1 production | 29/2334 | 120/29008 | 6.16E-08 | 1.13E-06 | 6.36E-07 |
| **733** | KO | GO:0050731 | positive regulation of peptidyl-tyrosine phosphorylation | 39/2334 | 192/29008 | 6.32E-08 | 1.16E-06 | 6.50E-07 |
| **734** | KO | GO:0006778 | porphyrin-containing compound metabolic process | 16/2334 | 42/29008 | 6.46E-08 | 1.18E-06 | 6.62E-07 |
| **735** | KO | GO:0045017 | glycerolipid biosynthetic process | 41/2334 | 208/29008 | 7.06E-08 | 1.28E-06 | 7.23E-07 |
| **736** | KO | GO:0006720 | isoprenoid metabolic process | 24/2334 | 88/29008 | 7.16E-08 | 1.30E-06 | 7.30E-07 |
| **737** | KO | GO:0051098 | regulation of binding | 64/2334 | 397/29008 | 7.25E-08 | 1.31E-06 | 7.37E-07 |
| **738** | KO | GO:0006081 | cellular aldehyde metabolic process | 20/2334 | 64/29008 | 7.36E-08 | 1.33E-06 | 7.46E-07 |
| **739** | KO | GO:0046031 | ADP metabolic process | 26/2334 | 101/29008 | 7.49E-08 | 1.35E-06 | 7.57E-07 |
| **740** | KO | GO:0031346 | positive regulation of cell projection organization | 69/2334 | 441/29008 | 7.54E-08 | 1.35E-06 | 7.60E-07 |
| **741** | KO | GO:0006887 | exocytosis | 66/2334 | 415/29008 | 7.71E-08 | 1.38E-06 | 7.75E-07 |
| **742** | KO | GO:0032727 | positive regulation of interferon-alpha production | 12/2334 | 24/29008 | 7.74E-08 | 1.38E-06 | 7.75E-07 |
| **743** | KO | GO:0032731 | positive regulation of interleukin-1 beta production | 19/2334 | 59/29008 | 9.05E-08 | 1.61E-06 | 9.05E-07 |
| **744** | KO | GO:0046633 | alpha-beta T cell proliferation | 16/2334 | 43/29008 | 9.51E-08 | 1.68E-06 | 9.48E-07 |
| **745** | KO | GO:0008154 | actin polymerization or depolymerization | 42/2334 | 218/29008 | 9.62E-08 | 1.70E-06 | 9.56E-07 |
| **746** | KO | GO:0032722 | positive regulation of chemokine production | 23/2334 | 83/29008 | 9.67E-08 | 1.70E-06 | 9.58E-07 |
| **747** | KO | GO:0048545 | response to steroid hormone | 43/2334 | 226/29008 | 9.87E-08 | 1.73E-06 | 9.75E-07 |
| **748** | KO | GO:0071900 | regulation of protein serine/threonine kinase activity | 58/2334 | 349/29008 | 1.01E-07 | 1.77E-06 | 9.96E-07 |
| **749** | KO | GO:0032922 | circadian regulation of gene expression | 21/2334 | 71/29008 | 1.01E-07 | 1.77E-06 | 9.96E-07 |
| **750** | KO | GO:0032647 | regulation of interferon-alpha production | 13/2334 | 29/29008 | 1.14E-07 | 1.98E-06 | 1.11E-06 |
| **751** | KO | GO:0009743 | response to carbohydrate | 40/2334 | 204/29008 | 1.18E-07 | 2.04E-06 | 1.15E-06 |
| **752** | KO | GO:0002821 | positive regulation of adaptive immune response | 34/2334 | 159/29008 | 1.19E-07 | 2.06E-06 | 1.16E-06 |
| **753** | KO | GO:1903038 | negative regulation of leukocyte cell-cell adhesion | 32/2334 | 145/29008 | 1.29E-07 | 2.22E-06 | 1.25E-06 |
| **754** | KO | GO:0006109 | regulation of carbohydrate metabolic process | 39/2334 | 197/29008 | 1.29E-07 | 2.23E-06 | 1.25E-06 |
| **755** | KO | GO:2000377 | regulation of reactive oxygen species metabolic process | 34/2334 | 160/29008 | 1.40E-07 | 2.39E-06 | 1.35E-06 |
| **756** | KO | GO:0010720 | positive regulation of cell development | 61/2334 | 378/29008 | 1.40E-07 | 2.40E-06 | 1.35E-06 |
| **757** | KO | GO:1903532 | positive regulation of secretion by cell | 59/2334 | 361/29008 | 1.43E-07 | 2.44E-06 | 1.37E-06 |
| **758** | KO | GO:2000403 | positive regulation of lymphocyte migration | 15/2334 | 39/29008 | 1.43E-07 | 2.44E-06 | 1.37E-06 |
| **759** | KO | GO:0035458 | cellular response to interferon-beta | 18/2334 | 55/29008 | 1.49E-07 | 2.52E-06 | 1.42E-06 |
| **760** | KO | GO:0060612 | adipose tissue development | 18/2334 | 55/29008 | 1.49E-07 | 2.52E-06 | 1.42E-06 |
| **761** | KO | GO:0046634 | regulation of alpha-beta T cell activation | 27/2334 | 111/29008 | 1.50E-07 | 2.54E-06 | 1.43E-06 |
| **762** | KO | GO:0002275 | myeloid cell activation involved in immune response | 28/2334 | 118/29008 | 1.56E-07 | 2.63E-06 | 1.48E-06 |
| **763** | KO | GO:0140014 | mitotic nuclear division | 49/2334 | 278/29008 | 1.57E-07 | 2.64E-06 | 1.49E-06 |
| **764** | KO | GO:0070664 | negative regulation of leukocyte proliferation | 25/2334 | 98/29008 | 1.60E-07 | 2.69E-06 | 1.51E-06 |
| **765** | KO | GO:0033013 | tetrapyrrole metabolic process | 17/2334 | 50/29008 | 1.76E-07 | 2.93E-06 | 1.65E-06 |
| **766** | KO | GO:0071887 | leukocyte apoptotic process | 31/2334 | 140/29008 | 1.86E-07 | 3.09E-06 | 1.74E-06 |
| **767** | KO | GO:0034754 | cellular hormone metabolic process | 28/2334 | 119/29008 | 1.89E-07 | 3.13E-06 | 1.76E-06 |
| **768** | KO | GO:0052126 | movement in host environment | 29/2334 | 126/29008 | 1.91E-07 | 3.15E-06 | 1.78E-06 |
| **769** | KO | GO:0010639 | negative regulation of organelle organization | 59/2334 | 364/29008 | 1.91E-07 | 3.15E-06 | 1.78E-06 |
| **770** | KO | GO:0034340 | response to type I interferon | 18/2334 | 56/29008 | 2.03E-07 | 3.35E-06 | 1.88E-06 |
| **771** | KO | GO:0042168 | heme metabolic process | 14/2334 | 35/29008 | 2.10E-07 | 3.45E-06 | 1.94E-06 |
| **772** | KO | GO:0051099 | positive regulation of binding | 38/2334 | 193/29008 | 2.16E-07 | 3.54E-06 | 1.99E-06 |
| **773** | KO | GO:0008630 | intrinsic apoptotic signaling pathway in response to DNA damage | 27/2334 | 113/29008 | 2.22E-07 | 3.63E-06 | 2.04E-06 |
| **774** | KO | GO:0001776 | leukocyte homeostasis | 28/2334 | 120/29008 | 2.27E-07 | 3.70E-06 | 2.09E-06 |
| **775** | KO | GO:0050878 | regulation of body fluid levels | 59/2334 | 366/29008 | 2.31E-07 | 3.76E-06 | 2.12E-06 |
| **776** | KO | GO:0071692 | protein localization to extracellular region | 63/2334 | 401/29008 | 2.35E-07 | 3.81E-06 | 2.15E-06 |
| **777** | KO | GO:0030522 | intracellular receptor signaling pathway | 41/2334 | 217/29008 | 2.36E-07 | 3.81E-06 | 2.15E-06 |
| **778** | KO | GO:1903305 | regulation of regulated secretory pathway | 37/2334 | 186/29008 | 2.37E-07 | 3.82E-06 | 2.15E-06 |
| **779** | KO | GO:0002706 | regulation of lymphocyte mediated immunity | 42/2334 | 225/29008 | 2.39E-07 | 3.83E-06 | 2.16E-06 |
| **780** | KO | GO:0006006 | glucose metabolic process | 42/2334 | 225/29008 | 2.39E-07 | 3.83E-06 | 2.16E-06 |
| **781** | KO | GO:0031343 | positive regulation of cell killing | 25/2334 | 100/29008 | 2.44E-07 | 3.91E-06 | 2.20E-06 |
| **782** | KO | GO:0015850 | organic hydroxy compound transport | 49/2334 | 282/29008 | 2.46E-07 | 3.93E-06 | 2.21E-06 |
| **783** | KO | GO:0051701 | biological process involved in interaction with host | 32/2334 | 149/29008 | 2.49E-07 | 3.97E-06 | 2.23E-06 |
| **784** | KO | GO:0032602 | chemokine production | 27/2334 | 114/29008 | 2.69E-07 | 4.27E-06 | 2.40E-06 |
| **785** | KO | GO:0010769 | regulation of cell morphogenesis involved in differentiation | 28/2334 | 121/29008 | 2.73E-07 | 4.32E-06 | 2.43E-06 |
| **786** | KO | GO:0035634 | response to stilbenoid | 11/2334 | 22/29008 | 2.74E-07 | 4.32E-06 | 2.43E-06 |
| **787** | KO | GO:0008360 | regulation of cell shape | 33/2334 | 157/29008 | 2.77E-07 | 4.36E-06 | 2.45E-06 |
| **788** | KO | GO:0043434 | response to peptide hormone | 57/2334 | 351/29008 | 2.87E-07 | 4.51E-06 | 2.54E-06 |
| **789** | KO | GO:0008347 | glial cell migration | 19/2334 | 63/29008 | 2.91E-07 | 4.55E-06 | 2.56E-06 |
| **790** | KO | GO:0051348 | negative regulation of transferase activity | 46/2334 | 259/29008 | 2.97E-07 | 4.64E-06 | 2.61E-06 |
| **791** | KO | GO:0034248 | regulation of cellular amide metabolic process | 68/2334 | 448/29008 | 2.99E-07 | 4.66E-06 | 2.62E-06 |
| **792** | KO | GO:0001959 | regulation of cytokine-mediated signaling pathway | 28/2334 | 122/29008 | 3.28E-07 | 5.10E-06 | 2.87E-06 |
| **793** | KO | GO:0034308 | primary alcohol metabolic process | 22/2334 | 82/29008 | 3.39E-07 | 5.26E-06 | 2.96E-06 |
| **794** | KO | GO:0006839 | mitochondrial transport | 40/2334 | 212/29008 | 3.42E-07 | 5.29E-06 | 2.97E-06 |
| **795** | KO | GO:0032757 | positive regulation of interleukin-8 production | 18/2334 | 58/29008 | 3.68E-07 | 5.69E-06 | 3.20E-06 |
| **796** | KO | GO:0002444 | myeloid leukocyte mediated immunity | 29/2334 | 130/29008 | 3.86E-07 | 5.95E-06 | 3.35E-06 |
| **797** | KO | GO:1905954 | positive regulation of lipid localization | 28/2334 | 123/29008 | 3.92E-07 | 6.02E-06 | 3.39E-06 |
| **798** | KO | GO:0031664 | regulation of lipopolysaccharide-mediated signaling pathway | 12/2334 | 27/29008 | 3.96E-07 | 6.06E-06 | 3.41E-06 |
| **799** | KO | GO:0034284 | response to monosaccharide | 37/2334 | 190/29008 | 4.12E-07 | 6.30E-06 | 3.55E-06 |
| **800** | KO | GO:0110053 | regulation of actin filament organization | 48/2334 | 279/29008 | 4.39E-07 | 6.70E-06 | 3.77E-06 |
| **801** | KO | GO:0042445 | hormone metabolic process | 40/2334 | 214/29008 | 4.41E-07 | 6.70E-06 | 3.77E-06 |
| **802** | KO | GO:1901617 | organic hydroxy compound biosynthetic process | 42/2334 | 230/29008 | 4.43E-07 | 6.72E-06 | 3.78E-06 |
| **803** | KO | GO:0140694 | non-membrane-bounded organelle assembly | 59/2334 | 373/29008 | 4.45E-07 | 6.73E-06 | 3.79E-06 |
| **804** | KO | GO:0010770 | positive regulation of cell morphogenesis involved in differentiation | 25/2334 | 103/29008 | 4.48E-07 | 6.77E-06 | 3.81E-06 |
| **805** | KO | GO:0060760 | positive regulation of response to cytokine stimulus | 17/2334 | 53/29008 | 4.56E-07 | 6.87E-06 | 3.87E-06 |
| **806** | KO | GO:0032642 | regulation of chemokine production | 26/2334 | 110/29008 | 4.64E-07 | 6.97E-06 | 3.92E-06 |
| **807** | KO | GO:0032368 | regulation of lipid transport | 32/2334 | 153/29008 | 4.69E-07 | 7.02E-06 | 3.95E-06 |
| **808** | KO | GO:0001909 | leukocyte mediated cytotoxicity | 34/2334 | 168/29008 | 4.71E-07 | 7.04E-06 | 3.96E-06 |
| **809** | KO | GO:1902624 | positive regulation of neutrophil migration | 14/2334 | 37/29008 | 4.74E-07 | 7.08E-06 | 3.98E-06 |
| **810** | KO | GO:0031669 | cellular response to nutrient levels | 38/2334 | 199/29008 | 4.86E-07 | 7.22E-06 | 4.07E-06 |
| **811** | KO | GO:0032732 | positive regulation of interleukin-1 production | 20/2334 | 71/29008 | 4.88E-07 | 7.25E-06 | 4.08E-06 |
| **812** | KO | GO:0006639 | acylglycerol metabolic process | 31/2334 | 146/29008 | 4.97E-07 | 7.36E-06 | 4.14E-06 |
| **813** | KO | GO:0050854 | regulation of antigen receptor-mediated signaling pathway | 19/2334 | 65/29008 | 4.99E-07 | 7.38E-06 | 4.15E-06 |
| **814** | KO | GO:0015833 | peptide transport | 52/2334 | 314/29008 | 5.06E-07 | 7.46E-06 | 4.20E-06 |
| **815** | KO | GO:0045582 | positive regulation of T cell differentiation | 25/2334 | 104/29008 | 5.45E-07 | 8.01E-06 | 4.51E-06 |
| **816** | KO | GO:0009306 | protein secretion | 61/2334 | 393/29008 | 5.54E-07 | 8.13E-06 | 4.57E-06 |
| **817** | KO | GO:0050708 | regulation of protein secretion | 50/2334 | 298/29008 | 5.57E-07 | 8.15E-06 | 4.59E-06 |
| **818** | KO | GO:0044770 | cell cycle phase transition | 72/2334 | 492/29008 | 5.63E-07 | 8.22E-06 | 4.63E-06 |
| **819** | KO | GO:0071466 | cellular response to xenobiotic stimulus | 35/2334 | 177/29008 | 5.74E-07 | 8.36E-06 | 4.70E-06 |
| **820** | KO | GO:2000379 | positive regulation of reactive oxygen species metabolic process | 21/2334 | 78/29008 | 5.85E-07 | 8.50E-06 | 4.78E-06 |
| **821** | KO | GO:0035592 | establishment of protein localization to extracellular region | 61/2334 | 394/29008 | 6.05E-07 | 8.77E-06 | 4.94E-06 |
| **822** | KO | GO:0017157 | regulation of exocytosis | 44/2334 | 249/29008 | 6.14E-07 | 8.88E-06 | 5.00E-06 |
| **823** | KO | GO:0034154 | toll-like receptor 7 signaling pathway | 8/2334 | 12/29008 | 6.40E-07 | 9.23E-06 | 5.19E-06 |
| **824** | KO | GO:2001238 | positive regulation of extrinsic apoptotic signaling pathway | 18/2334 | 60/29008 | 6.48E-07 | 9.32E-06 | 5.25E-06 |
| **825** | KO | GO:0045926 | negative regulation of growth | 46/2334 | 266/29008 | 6.51E-07 | 9.34E-06 | 5.25E-06 |
| **826** | KO | GO:0002768 | immune response-regulating cell surface receptor signaling pathway | 56/2334 | 351/29008 | 6.52E-07 | 9.34E-06 | 5.25E-06 |
| **827** | KO | GO:2000106 | regulation of leukocyte apoptotic process | 26/2334 | 112/29008 | 6.74E-07 | 9.63E-06 | 5.42E-06 |
| **828** | KO | GO:0006638 | neutral lipid metabolic process | 31/2334 | 148/29008 | 6.80E-07 | 9.69E-06 | 5.45E-06 |
| **829** | KO | GO:0034504 | protein localization to nucleus | 52/2334 | 317/29008 | 6.82E-07 | 9.70E-06 | 5.46E-06 |
| **830** | KO | GO:0046474 | glycerophospholipid biosynthetic process | 33/2334 | 163/29008 | 6.84E-07 | 9.70E-06 | 5.46E-06 |
| **831** | KO | GO:1901987 | regulation of cell cycle phase transition | 59/2334 | 378/29008 | 6.99E-07 | 9.90E-06 | 5.57E-06 |
| **832** | KO | GO:0048285 | organelle fission | 72/2334 | 495/29008 | 7.10E-07 | 1.00E-05 | 5.64E-06 |
| **833** | KO | GO:0003018 | vascular process in circulatory system | 42/2334 | 234/29008 | 7.15E-07 | 1.00E-05 | 5.65E-06 |
| **834** | KO | GO:0010038 | response to metal ion | 49/2334 | 292/29008 | 7.17E-07 | 1.00E-05 | 5.65E-06 |
| **835** | KO | GO:0006575 | cellular modified amino acid metabolic process | 38/2334 | 202/29008 | 7.17E-07 | 1.00E-05 | 5.65E-06 |
| **836** | KO | GO:0048771 | tissue remodeling | 38/2334 | 202/29008 | 7.17E-07 | 1.00E-05 | 5.65E-06 |
| **837** | KO | GO:0007163 | establishment or maintenance of cell polarity | 41/2334 | 226/29008 | 7.22E-07 | 1.01E-05 | 5.67E-06 |
| **838** | KO | GO:0001558 | regulation of cell growth | 67/2334 | 450/29008 | 7.38E-07 | 1.03E-05 | 5.79E-06 |
| **839** | KO | GO:0046034 | ATP metabolic process | 45/2334 | 259/29008 | 7.50E-07 | 1.04E-05 | 5.86E-06 |
| **840** | KO | GO:1901990 | regulation of mitotic cell cycle phase transition | 49/2334 | 293/29008 | 7.94E-07 | 1.10E-05 | 6.20E-06 |
| **841** | KO | GO:0042440 | pigment metabolic process | 20/2334 | 73/29008 | 7.96E-07 | 1.10E-05 | 6.20E-06 |
| **842** | KO | GO:0051047 | positive regulation of secretion | 63/2334 | 415/29008 | 7.98E-07 | 1.10E-05 | 6.20E-06 |
| **843** | KO | GO:0030101 | natural killer cell activation | 25/2334 | 106/29008 | 8.00E-07 | 1.10E-05 | 6.20E-06 |
| **844** | KO | GO:0006805 | xenobiotic metabolic process | 27/2334 | 120/29008 | 8.02E-07 | 1.10E-05 | 6.21E-06 |
| **845** | KO | GO:0070374 | positive regulation of ERK1 and ERK2 cascade | 41/2334 | 227/29008 | 8.14E-07 | 1.12E-05 | 6.28E-06 |
| **846** | KO | GO:0032930 | positive regulation of superoxide anion generation | 11/2334 | 24/29008 | 8.32E-07 | 1.14E-05 | 6.41E-06 |
| **847** | KO | GO:0032651 | regulation of interleukin-1 beta production | 23/2334 | 93/29008 | 8.97E-07 | 1.22E-05 | 6.87E-06 |
| **848** | KO | GO:0032945 | negative regulation of mononuclear cell proliferation | 23/2334 | 93/29008 | 8.97E-07 | 1.22E-05 | 6.87E-06 |
| **849** | KO | GO:0045580 | regulation of T cell differentiation | 33/2334 | 165/29008 | 9.13E-07 | 1.24E-05 | 6.98E-06 |
| **850** | KO | GO:0002371 | dendritic cell cytokine production | 9/2334 | 16/29008 | 9.45E-07 | 1.28E-05 | 7.20E-06 |
| **851** | KO | GO:0034162 | toll-like receptor 9 signaling pathway | 9/2334 | 16/29008 | 9.45E-07 | 1.28E-05 | 7.20E-06 |
| **852** | KO | GO:0043270 | positive regulation of ion transport | 53/2334 | 329/29008 | 9.50E-07 | 1.28E-05 | 7.22E-06 |
| **853** | KO | GO:0032652 | regulation of interleukin-1 production | 26/2334 | 114/29008 | 9.68E-07 | 1.30E-05 | 7.34E-06 |
| **854** | KO | GO:0002888 | positive regulation of myeloid leukocyte mediated immunity | 12/2334 | 29/29008 | 1.01E-06 | 1.36E-05 | 7.67E-06 |
| **855** | KO | GO:0009746 | response to hexose | 36/2334 | 189/29008 | 1.02E-06 | 1.37E-05 | 7.70E-06 |
| **856** | KO | GO:0042088 | T-helper 1 type immune response | 16/2334 | 50/29008 | 1.03E-06 | 1.37E-05 | 7.70E-06 |
| **857** | KO | GO:0060337 | type I interferon signaling pathway | 16/2334 | 50/29008 | 1.03E-06 | 1.37E-05 | 7.70E-06 |
| **858** | KO | GO:0071357 | cellular response to type I interferon | 16/2334 | 50/29008 | 1.03E-06 | 1.37E-05 | 7.70E-06 |
| **859** | KO | GO:2000406 | positive regulation of T cell migration | 13/2334 | 34/29008 | 1.06E-06 | 1.41E-05 | 7.93E-06 |
| **860** | KO | GO:0050921 | positive regulation of chemotaxis | 31/2334 | 151/29008 | 1.07E-06 | 1.42E-05 | 7.99E-06 |
| **861** | KO | GO:0006721 | terpenoid metabolic process | 19/2334 | 68/29008 | 1.07E-06 | 1.42E-05 | 7.99E-06 |
| **862** | KO | GO:1901224 | positive regulation of NIK/NF-kappaB signaling | 19/2334 | 68/29008 | 1.07E-06 | 1.42E-05 | 7.99E-06 |
| **863** | KO | GO:0046632 | alpha-beta T cell differentiation | 28/2334 | 129/29008 | 1.09E-06 | 1.44E-05 | 8.12E-06 |
| **864** | KO | GO:0045055 | regulated exocytosis | 48/2334 | 288/29008 | 1.13E-06 | 1.49E-05 | 8.38E-06 |
| **865** | KO | GO:0000070 | mitotic sister chromatid segregation | 32/2334 | 159/29008 | 1.15E-06 | 1.51E-05 | 8.48E-06 |
| **866** | KO | GO:0045824 | negative regulation of innate immune response | 21/2334 | 81/29008 | 1.15E-06 | 1.51E-05 | 8.50E-06 |
| **867** | KO | GO:0051225 | spindle assembly | 26/2334 | 115/29008 | 1.16E-06 | 1.51E-05 | 8.50E-06 |
| **868** | KO | GO:0043405 | regulation of MAP kinase activity | 37/2334 | 198/29008 | 1.18E-06 | 1.54E-05 | 8.66E-06 |
| **869** | KO | GO:1901215 | negative regulation of neuron death | 44/2334 | 255/29008 | 1.20E-06 | 1.56E-05 | 8.79E-06 |
| **870** | KO | GO:0009261 | ribonucleotide catabolic process | 15/2334 | 45/29008 | 1.24E-06 | 1.61E-05 | 9.05E-06 |
| **871** | KO | GO:0002824 | positive regulation of adaptive immune response based on somatic recombination of immune receptors built from immunoglobulin superfamily domains | 31/2334 | 152/29008 | 1.25E-06 | 1.61E-05 | 9.07E-06 |
| **872** | KO | GO:0010631 | epithelial cell migration | 50/2334 | 306/29008 | 1.25E-06 | 1.61E-05 | 9.07E-06 |
| **873** | KO | GO:0002757 | immune response-activating signal transduction | 54/2334 | 341/29008 | 1.30E-06 | 1.67E-05 | 9.41E-06 |
| **874** | KO | GO:0009636 | response to toxic substance | 27/2334 | 123/29008 | 1.34E-06 | 1.73E-05 | 9.72E-06 |
| **875** | KO | GO:0034446 | substrate adhesion-dependent cell spreading | 25/2334 | 109/29008 | 1.39E-06 | 1.78E-05 | 1.00E-05 |
| **876** | KO | GO:0046939 | nucleotide phosphorylation | 25/2334 | 109/29008 | 1.39E-06 | 1.78E-05 | 1.00E-05 |
| **877** | KO | GO:0006633 | fatty acid biosynthetic process | 31/2334 | 153/29008 | 1.44E-06 | 1.85E-05 | 1.04E-05 |
| **878** | KO | GO:0090132 | epithelium migration | 50/2334 | 308/29008 | 1.51E-06 | 1.93E-05 | 1.09E-05 |
| **879** | KO | GO:0002758 | innate immune response-activating signal transduction | 8/2334 | 13/29008 | 1.55E-06 | 1.97E-05 | 1.11E-05 |
| **880** | KO | GO:0043032 | positive regulation of macrophage activation | 12/2334 | 30/29008 | 1.57E-06 | 1.99E-05 | 1.12E-05 |
| **881** | KO | GO:1901658 | glycosyl compound catabolic process | 12/2334 | 30/29008 | 1.57E-06 | 1.99E-05 | 1.12E-05 |
| **882** | KO | GO:0007088 | regulation of mitotic nuclear division | 25/2334 | 110/29008 | 1.66E-06 | 2.11E-05 | 1.19E-05 |
| **883** | KO | GO:0044772 | mitotic cell cycle phase transition | 61/2334 | 406/29008 | 1.68E-06 | 2.12E-05 | 1.19E-05 |
| **884** | KO | GO:0072523 | purine-containing compound catabolic process | 15/2334 | 46/29008 | 1.70E-06 | 2.15E-05 | 1.21E-05 |
| **885** | KO | GO:0010959 | regulation of metal ion transport | 65/2334 | 443/29008 | 1.79E-06 | 2.25E-05 | 1.27E-05 |
| **886** | KO | GO:0045787 | positive regulation of cell cycle | 53/2334 | 336/29008 | 1.82E-06 | 2.29E-05 | 1.29E-05 |
| **887** | KO | GO:0007596 | blood coagulation | 33/2334 | 170/29008 | 1.83E-06 | 2.30E-05 | 1.29E-05 |
| **888** | KO | GO:0090130 | tissue migration | 50/2334 | 310/29008 | 1.83E-06 | 2.30E-05 | 1.29E-05 |
| **889** | KO | GO:0016311 | dephosphorylation | 54/2334 | 345/29008 | 1.86E-06 | 2.33E-05 | 1.31E-05 |
| **890** | KO | GO:0051260 | protein homooligomerization | 34/2334 | 178/29008 | 1.88E-06 | 2.35E-05 | 1.32E-05 |
| **891** | KO | GO:0009749 | response to glucose | 35/2334 | 186/29008 | 1.92E-06 | 2.39E-05 | 1.34E-05 |
| **892** | KO | GO:0030308 | negative regulation of cell growth | 37/2334 | 202/29008 | 1.94E-06 | 2.41E-05 | 1.35E-05 |
| **893** | KO | GO:0001503 | ossification | 62/2334 | 417/29008 | 1.97E-06 | 2.44E-05 | 1.38E-05 |
| **894** | KO | GO:0031334 | positive regulation of protein-containing complex assembly | 42/2334 | 243/29008 | 1.98E-06 | 2.45E-05 | 1.38E-05 |
| **895** | KO | GO:0048144 | fibroblast proliferation | 25/2334 | 111/29008 | 1.98E-06 | 2.45E-05 | 1.38E-05 |
| **896** | KO | GO:0006220 | pyrimidine nucleotide metabolic process | 14/2334 | 41/29008 | 2.01E-06 | 2.48E-05 | 1.39E-05 |
| **897** | KO | GO:0009895 | negative regulation of catabolic process | 50/2334 | 311/29008 | 2.02E-06 | 2.48E-05 | 1.40E-05 |
| **898** | KO | GO:0034163 | regulation of toll-like receptor 9 signaling pathway | 7/2334 | 10/29008 | 2.09E-06 | 2.56E-05 | 1.44E-05 |
| **899** | KO | GO:0010675 | regulation of cellular carbohydrate metabolic process | 32/2334 | 164/29008 | 2.32E-06 | 2.84E-05 | 1.60E-05 |
| **900** | KO | GO:0009166 | nucleotide catabolic process | 18/2334 | 65/29008 | 2.36E-06 | 2.88E-05 | 1.62E-05 |
| **901** | KO | GO:1901264 | carbohydrate derivative transport | 18/2334 | 65/29008 | 2.36E-06 | 2.88E-05 | 1.62E-05 |
| **902** | KO | GO:0002825 | regulation of T-helper 1 type immune response | 12/2334 | 31/29008 | 2.37E-06 | 2.88E-05 | 1.62E-05 |
| **903** | KO | GO:0035455 | response to interferon-alpha | 12/2334 | 31/29008 | 2.37E-06 | 2.88E-05 | 1.62E-05 |
| **904** | KO | GO:1905039 | carboxylic acid transmembrane transport | 28/2334 | 134/29008 | 2.43E-06 | 2.94E-05 | 1.66E-05 |
| **905** | KO | GO:0046638 | positive regulation of alpha-beta T cell differentiation | 16/2334 | 53/29008 | 2.45E-06 | 2.97E-05 | 1.67E-05 |
| **906** | KO | GO:0002429 | immune response-activating cell surface receptor signaling pathway | 53/2334 | 340/29008 | 2.62E-06 | 3.16E-05 | 1.78E-05 |
| **907** | KO | GO:0034329 | cell junction assembly | 64/2334 | 439/29008 | 2.66E-06 | 3.21E-05 | 1.81E-05 |
| **908** | KO | GO:0007599 | hemostasis | 33/2334 | 173/29008 | 2.73E-06 | 3.28E-05 | 1.85E-05 |
| **909** | KO | GO:0050817 | coagulation | 33/2334 | 173/29008 | 2.73E-06 | 3.28E-05 | 1.85E-05 |
| **910** | KO | GO:0001655 | urogenital system development | 58/2334 | 385/29008 | 2.74E-06 | 3.28E-05 | 1.85E-05 |
| **911** | KO | GO:0010822 | positive regulation of mitochondrion organization | 19/2334 | 72/29008 | 2.76E-06 | 3.30E-05 | 1.86E-05 |
| **912** | KO | GO:0010721 | negative regulation of cell development | 37/2334 | 205/29008 | 2.78E-06 | 3.32E-05 | 1.87E-05 |
| **913** | KO | GO:0043409 | negative regulation of MAPK cascade | 35/2334 | 189/29008 | 2.80E-06 | 3.34E-05 | 1.88E-05 |
| **914** | KO | GO:0006096 | glycolytic process | 22/2334 | 92/29008 | 2.81E-06 | 3.34E-05 | 1.88E-05 |
| **915** | KO | GO:0050672 | negative regulation of lymphocyte proliferation | 22/2334 | 92/29008 | 2.81E-06 | 3.34E-05 | 1.88E-05 |
| **916** | KO | GO:1903825 | organic acid transmembrane transport | 28/2334 | 135/29008 | 2.83E-06 | 3.35E-05 | 1.89E-05 |
| **917** | KO | GO:0006165 | nucleoside diphosphate phosphorylation | 24/2334 | 106/29008 | 2.86E-06 | 3.37E-05 | 1.90E-05 |
| **918** | KO | GO:0044409 | entry into host | 24/2334 | 106/29008 | 2.86E-06 | 3.37E-05 | 1.90E-05 |
| **919** | KO | GO:0048732 | gland development | 68/2334 | 477/29008 | 2.91E-06 | 3.43E-05 | 1.93E-05 |
| **920** | KO | GO:0002283 | neutrophil activation involved in immune response | 10/2334 | 22/29008 | 2.92E-06 | 3.43E-05 | 1.93E-05 |
| **921** | KO | GO:0061081 | positive regulation of myeloid leukocyte cytokine production involved in immune response | 10/2334 | 22/29008 | 2.92E-06 | 3.43E-05 | 1.93E-05 |
| **922** | KO | GO:0010001 | glial cell differentiation | 43/2334 | 255/29008 | 2.93E-06 | 3.43E-05 | 1.93E-05 |
| **923** | KO | GO:0001889 | liver development | 27/2334 | 128/29008 | 3.02E-06 | 3.52E-05 | 1.98E-05 |
| **924** | KO | GO:0051961 | negative regulation of nervous system development | 32/2334 | 166/29008 | 3.04E-06 | 3.55E-05 | 2.00E-05 |
| **925** | KO | GO:0071375 | cellular response to peptide hormone stimulus | 44/2334 | 264/29008 | 3.10E-06 | 3.61E-05 | 2.03E-05 |
| **926** | KO | GO:0031647 | regulation of protein stability | 49/2334 | 307/29008 | 3.13E-06 | 3.64E-05 | 2.05E-05 |
| **927** | KO | GO:0043122 | regulation of I-kappaB kinase/NF-kappaB signaling | 35/2334 | 190/29008 | 3.18E-06 | 3.68E-05 | 2.07E-05 |
| **928** | KO | GO:0006757 | ATP generation from ADP | 22/2334 | 93/29008 | 3.41E-06 | 3.94E-05 | 2.22E-05 |
| **929** | KO | GO:0044403 | biological process involved in symbiotic interaction | 44/2334 | 265/29008 | 3.44E-06 | 3.96E-05 | 2.23E-05 |
| **930** | KO | GO:0046635 | positive regulation of alpha-beta T cell activation | 19/2334 | 73/29008 | 3.46E-06 | 3.96E-05 | 2.23E-05 |
| **931** | KO | GO:0009165 | nucleotide biosynthetic process | 38/2334 | 215/29008 | 3.46E-06 | 3.96E-05 | 2.23E-05 |
| **932** | KO | GO:0002827 | positive regulation of T-helper 1 type immune response | 9/2334 | 18/29008 | 3.46E-06 | 3.96E-05 | 2.23E-05 |
| **933** | KO | GO:0006862 | nucleotide transport | 11/2334 | 27/29008 | 3.46E-06 | 3.96E-05 | 2.23E-05 |
| **934** | KO | GO:0032069 | regulation of nuclease activity | 11/2334 | 27/29008 | 3.46E-06 | 3.96E-05 | 2.23E-05 |
| **935** | KO | GO:0051048 | negative regulation of secretion | 36/2334 | 199/29008 | 3.57E-06 | 4.08E-05 | 2.29E-05 |
| **936** | KO | GO:0007051 | spindle organization | 34/2334 | 183/29008 | 3.58E-06 | 4.09E-05 | 2.30E-05 |
| **937** | KO | GO:0030041 | actin filament polymerization | 35/2334 | 191/29008 | 3.59E-06 | 4.09E-05 | 2.30E-05 |
| **938** | KO | GO:0048608 | reproductive structure development | 68/2334 | 480/29008 | 3.62E-06 | 4.12E-05 | 2.32E-05 |
| **939** | KO | GO:0006816 | calcium ion transport | 63/2334 | 434/29008 | 3.66E-06 | 4.15E-05 | 2.34E-05 |
| **940** | KO | GO:0002832 | negative regulation of response to biotic stimulus | 25/2334 | 115/29008 | 3.92E-06 | 4.43E-05 | 2.50E-05 |
| **941** | KO | GO:0002637 | regulation of immunoglobulin production | 21/2334 | 87/29008 | 4.01E-06 | 4.52E-05 | 2.55E-05 |
| **942** | KO | GO:0048145 | regulation of fibroblast proliferation | 24/2334 | 108/29008 | 4.05E-06 | 4.57E-05 | 2.57E-05 |
| **943** | KO | GO:0043524 | negative regulation of neuron apoptotic process | 34/2334 | 184/29008 | 4.06E-06 | 4.57E-05 | 2.57E-05 |
| **944** | KO | GO:0007009 | plasma membrane organization | 29/2334 | 145/29008 | 4.07E-06 | 4.57E-05 | 2.57E-05 |
| **945** | KO | GO:0010812 | negative regulation of cell-substrate adhesion | 17/2334 | 61/29008 | 4.08E-06 | 4.57E-05 | 2.57E-05 |
| **946** | KO | GO:0002312 | B cell activation involved in immune response | 22/2334 | 94/29008 | 4.11E-06 | 4.59E-05 | 2.59E-05 |
| **947** | KO | GO:0002532 | production of molecular mediator involved in inflammatory response | 22/2334 | 94/29008 | 4.11E-06 | 4.59E-05 | 2.59E-05 |
| **948** | KO | GO:1902622 | regulation of neutrophil migration | 15/2334 | 49/29008 | 4.15E-06 | 4.63E-05 | 2.61E-05 |
| **949** | KO | GO:0009116 | nucleoside metabolic process | 16/2334 | 55/29008 | 4.21E-06 | 4.69E-05 | 2.64E-05 |
| **950** | KO | GO:0031099 | regeneration | 30/2334 | 153/29008 | 4.27E-06 | 4.75E-05 | 2.67E-05 |
| **951** | KO | GO:0050688 | regulation of defense response to virus | 19/2334 | 74/29008 | 4.30E-06 | 4.77E-05 | 2.69E-05 |
| **952** | KO | GO:0045765 | regulation of angiogenesis | 48/2334 | 302/29008 | 4.42E-06 | 4.89E-05 | 2.75E-05 |
| **953** | KO | GO:0046165 | alcohol biosynthetic process | 28/2334 | 138/29008 | 4.43E-06 | 4.90E-05 | 2.76E-05 |
| **954** | KO | GO:0001953 | negative regulation of cell-matrix adhesion | 13/2334 | 38/29008 | 4.56E-06 | 5.02E-05 | 2.83E-05 |
| **955** | KO | GO:0019218 | regulation of steroid metabolic process | 25/2334 | 116/29008 | 4.62E-06 | 5.08E-05 | 2.86E-05 |
| **956** | KO | GO:1902850 | microtubule cytoskeleton organization involved in mitosis | 29/2334 | 146/29008 | 4.70E-06 | 5.16E-05 | 2.91E-05 |
| **957** | KO | GO:0097006 | regulation of plasma lipoprotein particle levels | 18/2334 | 68/29008 | 4.79E-06 | 5.24E-05 | 2.95E-05 |
| **958** | KO | GO:0061008 | hepaticobiliary system development | 27/2334 | 131/29008 | 4.79E-06 | 5.24E-05 | 2.95E-05 |
| **959** | KO | GO:0071359 | cellular response to dsRNA | 10/2334 | 23/29008 | 4.80E-06 | 5.25E-05 | 2.95E-05 |
| **960** | KO | GO:0061458 | reproductive system development | 68/2334 | 484/29008 | 4.83E-06 | 5.27E-05 | 2.96E-05 |
| **961** | KO | GO:1903829 | positive regulation of cellular protein localization | 45/2334 | 277/29008 | 4.84E-06 | 5.27E-05 | 2.97E-05 |
| **962** | KO | GO:0001906 | cell killing | 43/2334 | 260/29008 | 4.89E-06 | 5.32E-05 | 2.99E-05 |
| **963** | KO | GO:0046718 | viral entry into host cell | 23/2334 | 102/29008 | 4.92E-06 | 5.34E-05 | 3.01E-05 |
| **964** | KO | GO:0009260 | ribonucleotide biosynthetic process | 31/2334 | 162/29008 | 5.05E-06 | 5.46E-05 | 3.08E-05 |
| **965** | KO | GO:0050768 | negative regulation of neurogenesis | 31/2334 | 162/29008 | 5.05E-06 | 5.46E-05 | 3.08E-05 |
| **966** | KO | GO:0031341 | regulation of cell killing | 28/2334 | 139/29008 | 5.13E-06 | 5.54E-05 | 3.12E-05 |
| **967** | KO | GO:0008207 | C21-steroid hormone metabolic process | 14/2334 | 44/29008 | 5.22E-06 | 5.61E-05 | 3.16E-05 |
| **968** | KO | GO:0030225 | macrophage differentiation | 14/2334 | 44/29008 | 5.22E-06 | 5.61E-05 | 3.16E-05 |
| **969** | KO | GO:0051495 | positive regulation of cytoskeleton organization | 39/2334 | 227/29008 | 5.25E-06 | 5.64E-05 | 3.17E-05 |
| **970** | KO | GO:0052548 | regulation of endopeptidase activity | 54/2334 | 357/29008 | 5.28E-06 | 5.65E-05 | 3.18E-05 |
| **971** | KO | GO:0032928 | regulation of superoxide anion generation | 11/2334 | 28/29008 | 5.29E-06 | 5.65E-05 | 3.18E-05 |
| **972** | KO | GO:0090023 | positive regulation of neutrophil chemotaxis | 11/2334 | 28/29008 | 5.29E-06 | 5.65E-05 | 3.18E-05 |
| **973** | KO | GO:0002886 | regulation of myeloid leukocyte mediated immunity | 19/2334 | 75/29008 | 5.33E-06 | 5.69E-05 | 3.20E-05 |
| **974** | KO | GO:0009314 | response to radiation | 62/2334 | 430/29008 | 5.43E-06 | 5.78E-05 | 3.25E-05 |
| **975** | KO | GO:0016358 | dendrite development | 49/2334 | 313/29008 | 5.43E-06 | 5.78E-05 | 3.25E-05 |
| **976** | KO | GO:0002753 | cytoplasmic pattern recognition receptor signaling pathway | 15/2334 | 50/29008 | 5.49E-06 | 5.83E-05 | 3.28E-05 |
| **977** | KO | GO:0051053 | negative regulation of DNA metabolic process | 27/2334 | 132/29008 | 5.56E-06 | 5.89E-05 | 3.32E-05 |
| **978** | KO | GO:0034101 | erythrocyte homeostasis | 30/2334 | 155/29008 | 5.62E-06 | 5.94E-05 | 3.34E-05 |
| **979** | KO | GO:0031532 | actin cytoskeleton reorganization | 24/2334 | 110/29008 | 5.68E-06 | 6.00E-05 | 3.37E-05 |
| **980** | KO | GO:0008625 | extrinsic apoptotic signaling pathway via death domain receptors | 20/2334 | 82/29008 | 5.69E-06 | 6.00E-05 | 3.38E-05 |
| **981** | KO | GO:0045786 | negative regulation of cell cycle | 55/2334 | 367/29008 | 5.74E-06 | 6.04E-05 | 3.40E-05 |
| **982** | KO | GO:0015980 | energy derivation by oxidation of organic compounds | 48/2334 | 305/29008 | 5.83E-06 | 6.12E-05 | 3.44E-05 |
| **983** | KO | GO:0046390 | ribose phosphate biosynthetic process | 32/2334 | 171/29008 | 5.85E-06 | 6.13E-05 | 3.45E-05 |
| **984** | KO | GO:0001912 | positive regulation of leukocyte mediated cytotoxicity | 21/2334 | 89/29008 | 5.89E-06 | 6.15E-05 | 3.46E-05 |
| **985** | KO | GO:0019915 | lipid storage | 21/2334 | 89/29008 | 5.89E-06 | 6.15E-05 | 3.46E-05 |
| **986** | KO | GO:0045931 | positive regulation of mitotic cell cycle | 26/2334 | 125/29008 | 5.98E-06 | 6.24E-05 | 3.51E-05 |
| **987** | KO | GO:0046503 | glycerolipid catabolic process | 18/2334 | 69/29008 | 5.99E-06 | 6.24E-05 | 3.51E-05 |
| **988** | KO | GO:1901293 | nucleoside phosphate biosynthetic process | 38/2334 | 220/29008 | 6.06E-06 | 6.29E-05 | 3.54E-05 |
| **989** | KO | GO:0061448 | connective tissue development | 46/2334 | 288/29008 | 6.06E-06 | 6.29E-05 | 3.54E-05 |
| **990** | KO | GO:0042448 | progesterone metabolic process | 9/2334 | 19/29008 | 6.10E-06 | 6.32E-05 | 3.56E-05 |
| **991** | KO | GO:0043547 | positive regulation of GTPase activity | 40/2334 | 237/29008 | 6.25E-06 | 6.46E-05 | 3.64E-05 |
| **992** | KO | GO:0046640 | regulation of alpha-beta T cell proliferation | 13/2334 | 39/29008 | 6.33E-06 | 6.54E-05 | 3.68E-05 |
| **993** | KO | GO:1901342 | regulation of vasculature development | 48/2334 | 306/29008 | 6.38E-06 | 6.57E-05 | 3.70E-05 |
| **994** | KO | GO:0010508 | positive regulation of autophagy | 27/2334 | 133/29008 | 6.45E-06 | 6.64E-05 | 3.74E-05 |
| **995** | KO | GO:0034612 | response to tumor necrosis factor | 35/2334 | 196/29008 | 6.53E-06 | 6.71E-05 | 3.77E-05 |
| **996** | KO | GO:1901292 | nucleoside phosphate catabolic process | 19/2334 | 76/29008 | 6.57E-06 | 6.74E-05 | 3.80E-05 |
| **997** | KO | GO:0009620 | response to fungus | 17/2334 | 63/29008 | 6.59E-06 | 6.74E-05 | 3.80E-05 |
| **998** | KO | GO:0000819 | sister chromatid segregation | 34/2334 | 188/29008 | 6.61E-06 | 6.76E-05 | 3.81E-05 |
| **999** | KO | GO:0002730 | regulation of dendritic cell cytokine production | 8/2334 | 15/29008 | 6.67E-06 | 6.80E-05 | 3.83E-05 |
| **1000** | KO | GO:0032308 | positive regulation of prostaglandin secretion | 8/2334 | 15/29008 | 6.67E-06 | 6.80E-05 | 3.83E-05 |
| **1001** | KO | GO:0006641 | triglyceride metabolic process | 24/2334 | 111/29008 | 6.70E-06 | 6.81E-05 | 3.83E-05 |
| **1002** | KO | GO:0072001 | renal system development | 52/2334 | 342/29008 | 6.72E-06 | 6.83E-05 | 3.84E-05 |
| **1003** | KO | GO:0050769 | positive regulation of neurogenesis | 47/2334 | 298/29008 | 6.83E-06 | 6.93E-05 | 3.90E-05 |
| **1004** | KO | GO:0002573 | myeloid leukocyte differentiation | 40/2334 | 238/29008 | 6.93E-06 | 7.02E-05 | 3.95E-05 |
| **1005** | KO | GO:0043123 | positive regulation of I-kappaB kinase/NF-kappaB signaling | 26/2334 | 126/29008 | 6.96E-06 | 7.04E-05 | 3.96E-05 |
| **1006** | KO | GO:0050691 | regulation of defense response to virus by host | 14/2334 | 45/29008 | 7.01E-06 | 7.06E-05 | 3.98E-05 |
| **1007** | KO | GO:0071622 | regulation of granulocyte chemotaxis | 16/2334 | 57/29008 | 7.03E-06 | 7.06E-05 | 3.98E-05 |
| **1008** | KO | GO:0072527 | pyrimidine-containing compound metabolic process | 16/2334 | 57/29008 | 7.03E-06 | 7.06E-05 | 3.98E-05 |
| **1009** | KO | GO:0120162 | positive regulation of cold-induced thermogenesis | 22/2334 | 97/29008 | 7.10E-06 | 7.13E-05 | 4.01E-05 |
| **1010** | KO | GO:1904407 | positive regulation of nitric oxide metabolic process | 15/2334 | 51/29008 | 7.21E-06 | 7.22E-05 | 4.07E-05 |
| **1011** | KO | GO:1902903 | regulation of supramolecular fiber organization | 57/2334 | 388/29008 | 7.25E-06 | 7.25E-05 | 4.08E-05 |
| **1012** | KO | GO:0061351 | neural precursor cell proliferation | 35/2334 | 197/29008 | 7.33E-06 | 7.32E-05 | 4.12E-05 |
| **1013** | KO | GO:0007052 | mitotic spindle organization | 25/2334 | 119/29008 | 7.45E-06 | 7.43E-05 | 4.18E-05 |
| **1014** | KO | GO:0009218 | pyrimidine ribonucleotide metabolic process | 11/2334 | 29/29008 | 7.90E-06 | 7.87E-05 | 4.43E-05 |
| **1015** | KO | GO:0008286 | insulin receptor signaling pathway | 26/2334 | 127/29008 | 8.09E-06 | 8.05E-05 | 4.53E-05 |
| **1016** | KO | GO:0014074 | response to purine-containing compound | 23/2334 | 105/29008 | 8.23E-06 | 8.17E-05 | 4.60E-05 |
| **1017** | KO | GO:0050821 | protein stabilization | 34/2334 | 190/29008 | 8.38E-06 | 8.29E-05 | 4.67E-05 |
| **1018** | KO | GO:0060401 | cytosolic calcium ion transport | 34/2334 | 190/29008 | 8.38E-06 | 8.29E-05 | 4.67E-05 |
| **1019** | KO | GO:0043299 | leukocyte degranulation | 22/2334 | 98/29008 | 8.46E-06 | 8.36E-05 | 4.70E-05 |
| **1020** | KO | GO:0001890 | placenta development | 32/2334 | 174/29008 | 8.52E-06 | 8.40E-05 | 4.73E-05 |
| **1021** | KO | GO:0009063 | cellular amino acid catabolic process | 21/2334 | 91/29008 | 8.54E-06 | 8.41E-05 | 4.73E-05 |
| **1022** | KO | GO:0019884 | antigen processing and presentation of exogenous antigen | 13/2334 | 40/29008 | 8.69E-06 | 8.54E-05 | 4.81E-05 |
| **1023** | KO | GO:0098754 | detoxification | 16/2334 | 58/29008 | 8.98E-06 | 8.81E-05 | 4.96E-05 |
| **1024** | KO | GO:0050728 | negative regulation of inflammatory response | 28/2334 | 143/29008 | 9.04E-06 | 8.84E-05 | 4.98E-05 |
| **1025** | KO | GO:0051783 | regulation of nuclear division | 28/2334 | 143/29008 | 9.04E-06 | 8.84E-05 | 4.98E-05 |
| **1026** | KO | GO:0030888 | regulation of B cell proliferation | 18/2334 | 71/29008 | 9.25E-06 | 9.02E-05 | 5.08E-05 |
| **1027** | KO | GO:0042178 | xenobiotic catabolic process | 18/2334 | 71/29008 | 9.25E-06 | 9.02E-05 | 5.08E-05 |
| **1028** | KO | GO:0033157 | regulation of intracellular protein transport | 38/2334 | 224/29008 | 9.32E-06 | 9.07E-05 | 5.11E-05 |
| **1029** | KO | GO:0034142 | toll-like receptor 4 signaling pathway | 14/2334 | 46/29008 | 9.34E-06 | 9.08E-05 | 5.11E-05 |
| **1030** | KO | GO:0042752 | regulation of circadian rhythm | 26/2334 | 128/29008 | 9.39E-06 | 9.11E-05 | 5.13E-05 |
| **1031** | KO | GO:0008064 | regulation of actin polymerization or depolymerization | 34/2334 | 191/29008 | 9.42E-06 | 9.13E-05 | 5.14E-05 |
| **1032** | KO | GO:0051346 | negative regulation of hydrolase activity | 55/2334 | 374/29008 | 1.01E-05 | 9.75E-05 | 5.49E-05 |
| **1033** | KO | GO:0032305 | positive regulation of icosanoid secretion | 9/2334 | 20/29008 | 1.03E-05 | 9.93E-05 | 5.59E-05 |
| **1034** | KO | GO:0032695 | negative regulation of interleukin-12 production | 9/2334 | 20/29008 | 1.03E-05 | 9.93E-05 | 5.59E-05 |
| **1035** | KO | GO:0000038 | very long-chain fatty acid metabolic process | 12/2334 | 35/29008 | 1.03E-05 | 9.93E-05 | 5.59E-05 |
| **1036** | KO | GO:0090022 | regulation of neutrophil chemotaxis | 12/2334 | 35/29008 | 1.03E-05 | 9.93E-05 | 5.59E-05 |
| **1037** | KO | GO:2001234 | negative regulation of apoptotic signaling pathway | 40/2334 | 242/29008 | 1.04E-05 | 0.0001001989031 | 5.64E-05 |
| **1038** | KO | GO:0042594 | response to starvation | 33/2334 | 184/29008 | 1.08E-05 | 0.0001031312423 | 5.80E-05 |
| **1039** | KO | GO:0007265 | Ras protein signal transduction | 50/2334 | 330/29008 | 1.10E-05 | 0.0001055689587 | 5.94E-05 |
| **1040** | KO | GO:0002548 | monocyte chemotaxis | 16/2334 | 59/29008 | 1.14E-05 | 0.0001089922227 | 6.13E-05 |
| **1041** | KO | GO:0071624 | positive regulation of granulocyte chemotaxis | 11/2334 | 30/29008 | 1.16E-05 | 0.0001103761812 | 6.21E-05 |
| **1042** | KO | GO:0002446 | neutrophil mediated immunity | 13/2334 | 41/29008 | 1.18E-05 | 0.0001114085626 | 6.27E-05 |
| **1043** | KO | GO:0032733 | positive regulation of interleukin-10 production | 13/2334 | 41/29008 | 1.18E-05 | 0.0001114085626 | 6.27E-05 |
| **1044** | KO | GO:0046320 | regulation of fatty acid oxidation | 13/2334 | 41/29008 | 1.18E-05 | 0.0001114085626 | 6.27E-05 |
| **1045** | KO | GO:0071827 | plasma lipoprotein particle organization | 13/2334 | 41/29008 | 1.18E-05 | 0.0001114085626 | 6.27E-05 |
| **1046** | KO | GO:0006783 | heme biosynthetic process | 10/2334 | 25/29008 | 1.18E-05 | 0.0001114085626 | 6.27E-05 |
| **1047** | KO | GO:0032303 | regulation of icosanoid secretion | 10/2334 | 25/29008 | 1.18E-05 | 0.0001114085626 | 6.27E-05 |
| **1048** | KO | GO:2000193 | positive regulation of fatty acid transport | 10/2334 | 25/29008 | 1.18E-05 | 0.0001114085626 | 6.27E-05 |
| **1049** | KO | GO:0002220 | innate immune response activating cell surface receptor signaling pathway | 7/2334 | 12/29008 | 1.19E-05 | 0.0001120638595 | 6.31E-05 |
| **1050** | KO | GO:0038094 | Fc-gamma receptor signaling pathway | 7/2334 | 12/29008 | 1.19E-05 | 0.0001120638595 | 6.31E-05 |
| **1051** | KO | GO:0032370 | positive regulation of lipid transport | 22/2334 | 100/29008 | 1.19E-05 | 0.0001120638595 | 6.31E-05 |
| **1052** | KO | GO:0046626 | regulation of insulin receptor signaling pathway | 19/2334 | 79/29008 | 1.20E-05 | 0.0001128362477 | 6.35E-05 |
| **1053** | KO | GO:0033044 | regulation of chromosome organization | 33/2334 | 185/29008 | 1.21E-05 | 0.0001133055571 | 6.38E-05 |
| **1054** | KO | GO:0051983 | regulation of chromosome segregation | 20/2334 | 86/29008 | 1.23E-05 | 0.0001148272047 | 6.46E-05 |
| **1055** | KO | GO:0009755 | hormone-mediated signaling pathway | 30/2334 | 161/29008 | 1.23E-05 | 0.0001148272047 | 6.46E-05 |
| **1056** | KO | GO:2000404 | regulation of T cell migration | 14/2334 | 47/29008 | 1.23E-05 | 0.0001148272047 | 6.46E-05 |
| **1057** | KO | GO:0043312 | neutrophil degranulation | 8/2334 | 16/29008 | 1.24E-05 | 0.0001154158331 | 6.50E-05 |
| **1058** | KO | GO:0035710 | CD4-positive, alpha-beta T cell activation | 24/2334 | 115/29008 | 1.27E-05 | 0.0001176346299 | 6.62E-05 |
| **1059** | KO | GO:0032868 | response to insulin | 40/2334 | 244/29008 | 1.27E-05 | 0.0001182791924 | 6.66E-05 |
| **1060** | KO | GO:0030832 | regulation of actin filament length | 34/2334 | 194/29008 | 1.33E-05 | 0.000123034737 | 6.92E-05 |
| **1061** | KO | GO:0045637 | regulation of myeloid cell differentiation | 37/2334 | 219/29008 | 1.33E-05 | 0.0001233685798 | 6.94E-05 |
| **1062** | KO | GO:0050714 | positive regulation of protein secretion | 30/2334 | 162/29008 | 1.40E-05 | 0.0001289569299 | 7.26E-05 |
| **1063** | KO | GO:0046637 | regulation of alpha-beta T cell differentiation | 18/2334 | 73/29008 | 1.40E-05 | 0.0001293397149 | 7.28E-05 |
| **1064** | KO | GO:0045185 | maintenance of protein location | 22/2334 | 101/29008 | 1.41E-05 | 0.0001299059159 | 7.31E-05 |
| **1065** | KO | GO:0048638 | regulation of developmental growth | 58/2334 | 406/29008 | 1.42E-05 | 0.0001305253369 | 7.35E-05 |
| **1066** | KO | GO:0001523 | retinoid metabolic process | 16/2334 | 60/29008 | 1.44E-05 | 0.0001322418287 | 7.44E-05 |
| **1067** | KO | GO:0019233 | sensory perception of pain | 28/2334 | 147/29008 | 1.55E-05 | 0.0001418758959 | 7.98E-05 |
| **1068** | KO | GO:0048247 | lymphocyte chemotaxis | 15/2334 | 54/29008 | 1.55E-05 | 0.0001418758959 | 7.98E-05 |
| **1069** | KO | GO:0045766 | positive regulation of angiogenesis | 32/2334 | 179/29008 | 1.55E-05 | 0.0001418758959 | 7.98E-05 |
| **1070** | KO | GO:1904018 | positive regulation of vasculature development | 32/2334 | 179/29008 | 1.55E-05 | 0.0001418758959 | 7.98E-05 |
| **1071** | KO | GO:0002286 | T cell activation involved in immune response | 25/2334 | 124/29008 | 1.58E-05 | 0.0001442771373 | 8.12E-05 |
| **1072** | KO | GO:0000281 | mitotic cytokinesis | 17/2334 | 67/29008 | 1.61E-05 | 0.0001461643579 | 8.23E-05 |
| **1073** | KO | GO:0043277 | apoptotic cell clearance | 14/2334 | 48/29008 | 1.61E-05 | 0.0001461643579 | 8.23E-05 |
| **1074** | KO | GO:0045923 | positive regulation of fatty acid metabolic process | 14/2334 | 48/29008 | 1.61E-05 | 0.0001461643579 | 8.23E-05 |
| **1075** | KO | GO:0019217 | regulation of fatty acid metabolic process | 22/2334 | 102/29008 | 1.66E-05 | 0.0001504792905 | 8.47E-05 |
| **1076** | KO | GO:0050858 | negative regulation of antigen receptor-mediated signaling pathway | 11/2334 | 31/29008 | 1.66E-05 | 0.0001504792905 | 8.47E-05 |
| **1077** | KO | GO:1903975 | regulation of glial cell migration | 9/2334 | 21/29008 | 1.67E-05 | 0.0001512541503 | 8.51E-05 |
| **1078** | KO | GO:0098813 | nuclear chromosome segregation | 43/2334 | 273/29008 | 1.70E-05 | 0.0001535338084 | 8.64E-05 |
| **1079** | KO | GO:0032623 | interleukin-2 production | 18/2334 | 74/29008 | 1.71E-05 | 0.0001539045371 | 8.66E-05 |
| **1080** | KO | GO:0033627 | cell adhesion mediated by integrin | 18/2334 | 74/29008 | 1.71E-05 | 0.0001539045371 | 8.66E-05 |
| **1081** | KO | GO:1903036 | positive regulation of response to wounding | 18/2334 | 74/29008 | 1.71E-05 | 0.0001539045371 | 8.66E-05 |
| **1082** | KO | GO:0051282 | regulation of sequestering of calcium ion | 24/2334 | 117/29008 | 1.72E-05 | 0.0001539045371 | 8.66E-05 |
| **1083** | KO | GO:0045576 | mast cell activation | 21/2334 | 95/29008 | 1.73E-05 | 0.000154771992 | 8.71E-05 |
| **1084** | KO | GO:0072594 | establishment of protein localization to organelle | 59/2334 | 418/29008 | 1.73E-05 | 0.000154771992 | 8.71E-05 |
| **1085** | KO | GO:0006914 | autophagy | 61/2334 | 437/29008 | 1.77E-05 | 0.000157950295 | 8.89E-05 |
| **1086** | KO | GO:0061919 | process utilizing autophagic mechanism | 61/2334 | 437/29008 | 1.77E-05 | 0.000157950295 | 8.89E-05 |
| **1087** | KO | GO:0006925 | inflammatory cell apoptotic process | 10/2334 | 26/29008 | 1.78E-05 | 0.0001580299778 | 8.89E-05 |
| **1088** | KO | GO:0034656 | nucleobase-containing small molecule catabolic process | 10/2334 | 26/29008 | 1.78E-05 | 0.0001580299778 | 8.89E-05 |
| **1089** | KO | GO:0045730 | respiratory burst | 10/2334 | 26/29008 | 1.78E-05 | 0.0001580299778 | 8.89E-05 |
| **1090** | KO | GO:0010634 | positive regulation of epithelial cell migration | 29/2334 | 156/29008 | 1.79E-05 | 0.0001582491892 | 8.91E-05 |
| **1091** | KO | GO:0009267 | cellular response to starvation | 30/2334 | 164/29008 | 1.79E-05 | 0.0001582491892 | 8.91E-05 |
| **1092** | KO | GO:0043300 | regulation of leukocyte degranulation | 16/2334 | 61/29008 | 1.81E-05 | 0.0001597462388 | 8.99E-05 |
| **1093** | KO | GO:0001822 | kidney development | 49/2334 | 327/29008 | 1.81E-05 | 0.0001599929281 | 9.00E-05 |
| **1094** | KO | GO:0009308 | amine metabolic process | 25/2334 | 125/29008 | 1.83E-05 | 0.0001612167601 | 9.07E-05 |
| **1095** | KO | GO:0001666 | response to hypoxia | 36/2334 | 214/29008 | 1.91E-05 | 0.0001678626603 | 9.45E-05 |
| **1096** | KO | GO:2000191 | regulation of fatty acid transport | 12/2334 | 37/29008 | 1.97E-05 | 0.0001732340833 | 9.75E-05 |
| **1097** | KO | GO:0071902 | positive regulation of protein serine/threonine kinase activity | 35/2334 | 206/29008 | 1.99E-05 | 0.0001746629727 | 9.83E-05 |
| **1098** | KO | GO:0034502 | protein localization to chromosome | 21/2334 | 96/29008 | 2.05E-05 | 0.0001791440661 | 0.0001008237831 |
| **1099** | KO | GO:0042632 | cholesterol homeostasis | 21/2334 | 96/29008 | 2.05E-05 | 0.0001791440661 | 0.0001008237831 |
| **1100** | KO | GO:0016051 | carbohydrate biosynthetic process | 34/2334 | 198/29008 | 2.07E-05 | 0.0001804224186 | 0.0001015432506 |
| **1101** | KO | GO:0019722 | calcium-mediated signaling | 34/2334 | 198/29008 | 2.07E-05 | 0.0001804224186 | 0.0001015432506 |
| **1102** | KO | GO:0022411 | cellular component disassembly | 57/2334 | 402/29008 | 2.09E-05 | 0.0001807998497 | 0.000101755672 |
| **1103** | KO | GO:0042130 | negative regulation of T cell proliferation | 18/2334 | 75/29008 | 2.09E-05 | 0.0001807998497 | 0.000101755672 |
| **1104** | KO | GO:0080164 | regulation of nitric oxide metabolic process | 18/2334 | 75/29008 | 2.09E-05 | 0.0001807998497 | 0.000101755672 |
| **1105** | KO | GO:0045429 | positive regulation of nitric oxide biosynthetic process | 14/2334 | 49/29008 | 2.09E-05 | 0.0001807998497 | 0.000101755672 |
| **1106** | KO | GO:1902930 | regulation of alcohol biosynthetic process | 14/2334 | 49/29008 | 2.09E-05 | 0.0001807998497 | 0.000101755672 |
| **1107** | KO | GO:0008206 | bile acid metabolic process | 13/2334 | 43/29008 | 2.10E-05 | 0.0001807998497 | 0.000101755672 |
| **1108** | KO | GO:0032735 | positive regulation of interleukin-12 production | 13/2334 | 43/29008 | 2.10E-05 | 0.0001807998497 | 0.000101755672 |
| **1109** | KO | GO:0033046 | negative regulation of sister chromatid segregation | 13/2334 | 43/29008 | 2.10E-05 | 0.0001807998497 | 0.000101755672 |
| **1110** | KO | GO:0033048 | negative regulation of mitotic sister chromatid segregation | 13/2334 | 43/29008 | 2.10E-05 | 0.0001807998497 | 0.000101755672 |
| **1111** | KO | GO:2000816 | negative regulation of mitotic sister chromatid separation | 13/2334 | 43/29008 | 2.10E-05 | 0.0001807998497 | 0.000101755672 |
| **1112** | KO | GO:0046209 | nitric oxide metabolic process | 20/2334 | 89/29008 | 2.11E-05 | 0.0001810530137 | 0.0001018981548 |
| **1113** | KO | GO:0050810 | regulation of steroid biosynthetic process | 19/2334 | 82/29008 | 2.12E-05 | 0.0001823029687 | 0.00010260164 |
| **1114** | KO | GO:0002431 | Fc receptor mediated stimulatory signaling pathway | 8/2334 | 17/29008 | 2.18E-05 | 0.0001854458761 | 0.0001043704946 |
| **1115** | KO | GO:0010935 | regulation of macrophage cytokine production | 8/2334 | 17/29008 | 2.18E-05 | 0.0001854458761 | 0.0001043704946 |
| **1116** | KO | GO:0032306 | regulation of prostaglandin secretion | 8/2334 | 17/29008 | 2.18E-05 | 0.0001854458761 | 0.0001043704946 |
| **1117** | KO | GO:0038093 | Fc receptor signaling pathway | 8/2334 | 17/29008 | 2.18E-05 | 0.0001854458761 | 0.0001043704946 |
| **1118** | KO | GO:0071360 | cellular response to exogenous dsRNA | 8/2334 | 17/29008 | 2.18E-05 | 0.0001854458761 | 0.0001043704946 |
| **1119** | KO | GO:0050852 | T cell receptor signaling pathway | 26/2334 | 134/29008 | 2.20E-05 | 0.0001868826984 | 0.0001051791503 |
| **1120** | KO | GO:0034765 | regulation of ion transmembrane transport | 67/2334 | 497/29008 | 2.20E-05 | 0.0001872001351 | 0.0001053578063 |
| **1121** | KO | GO:0006690 | icosanoid metabolic process | 27/2334 | 142/29008 | 2.25E-05 | 0.0001908223439 | 0.0001073964158 |
| **1122** | KO | GO:0016101 | diterpenoid metabolic process | 16/2334 | 62/29008 | 2.25E-05 | 0.0001908223439 | 0.0001073964158 |
| **1123** | KO | GO:1905517 | macrophage migration | 16/2334 | 62/29008 | 2.25E-05 | 0.0001908223439 | 0.0001073964158 |
| **1124** | KO | GO:0001678 | cellular glucose homeostasis | 30/2334 | 166/29008 | 2.28E-05 | 0.0001924277155 | 0.0001082999324 |
| **1125** | KO | GO:0010256 | endomembrane system organization | 66/2334 | 488/29008 | 2.28E-05 | 0.0001925172484 | 0.0001083503223 |
| **1126** | KO | GO:0002708 | positive regulation of lymphocyte mediated immunity | 29/2334 | 158/29008 | 2.29E-05 | 0.0001931084976 | 0.0001086830823 |
| **1127** | KO | GO:0021782 | glial cell development | 24/2334 | 119/29008 | 2.31E-05 | 0.000193849551 | 0.0001091001533 |
| **1128** | KO | GO:0051208 | sequestering of calcium ion | 24/2334 | 119/29008 | 2.31E-05 | 0.000193849551 | 0.0001091001533 |
| **1129** | KO | GO:0051056 | regulation of small GTPase mediated signal transduction | 38/2334 | 233/29008 | 2.33E-05 | 0.0001957992794 | 0.0001101974769 |
| **1130** | KO | GO:0010506 | regulation of autophagy | 41/2334 | 259/29008 | 2.34E-05 | 0.0001962851422 | 0.0001104709245 |
| **1131** | KO | GO:0032060 | bleb assembly | 7/2334 | 13/29008 | 2.40E-05 | 0.0002012123424 | 0.0001132439941 |
| **1132** | KO | GO:0055092 | sterol homeostasis | 21/2334 | 97/29008 | 2.42E-05 | 0.0002019842336 | 0.0001136784209 |
| **1133** | KO | GO:0051924 | regulation of calcium ion transport | 44/2334 | 286/29008 | 2.47E-05 | 0.0002064329089 | 0.0001161821727 |
| **1134** | KO | GO:0034113 | heterotypic cell-cell adhesion | 15/2334 | 56/29008 | 2.50E-05 | 0.0002081775211 | 0.0001171640551 |
| **1135** | KO | GO:0090303 | positive regulation of wound healing | 15/2334 | 56/29008 | 2.50E-05 | 0.0002081775211 | 0.0001171640551 |
| **1136** | KO | GO:0006835 | dicarboxylic acid transport | 20/2334 | 90/29008 | 2.51E-05 | 0.0002081775211 | 0.0001171640551 |
| **1137** | KO | GO:0009100 | glycoprotein metabolic process | 49/2334 | 331/29008 | 2.51E-05 | 0.0002081775211 | 0.0001171640551 |
| **1138** | KO | GO:0048708 | astrocyte differentiation | 19/2334 | 83/29008 | 2.55E-05 | 0.0002113673297 | 0.0001189593062 |
| **1139** | KO | GO:0030218 | erythrocyte differentiation | 27/2334 | 143/29008 | 2.57E-05 | 0.0002126381201 | 0.0001196745177 |
| **1140** | KO | GO:1900180 | regulation of protein localization to nucleus | 28/2334 | 151/29008 | 2.59E-05 | 0.0002143406183 | 0.0001206326979 |
| **1141** | KO | GO:0006536 | glutamate metabolic process | 10/2334 | 27/29008 | 2.62E-05 | 0.000216332651 | 0.0001217538306 |
| **1142** | KO | GO:0090077 | foam cell differentiation | 10/2334 | 27/29008 | 2.62E-05 | 0.000216332651 | 0.0001217538306 |
| **1143** | KO | GO:0009164 | nucleoside catabolic process | 9/2334 | 22/29008 | 2.63E-05 | 0.0002166495289 | 0.0001219321722 |
| **1144** | KO | GO:0051962 | positive regulation of nervous system development | 52/2334 | 359/29008 | 2.64E-05 | 0.0002169116907 | 0.000122079719 |
| **1145** | KO | GO:0001910 | regulation of leukocyte mediated cytotoxicity | 24/2334 | 120/29008 | 2.67E-05 | 0.0002184574649 | 0.0001229496937 |
| **1146** | KO | GO:2000177 | regulation of neural precursor cell proliferation | 24/2334 | 120/29008 | 2.67E-05 | 0.0002184574649 | 0.0001229496937 |
| **1147** | KO | GO:0002701 | negative regulation of production of molecular mediator of immune response | 12/2334 | 38/29008 | 2.67E-05 | 0.0002184574649 | 0.0001229496937 |
| **1148** | KO | GO:0033028 | myeloid cell apoptotic process | 12/2334 | 38/29008 | 2.67E-05 | 0.0002184574649 | 0.0001229496937 |
| **1149** | KO | GO:0048661 | positive regulation of smooth muscle cell proliferation | 22/2334 | 105/29008 | 2.68E-05 | 0.0002192221445 | 0.0001233800618 |
| **1150** | KO | GO:0051656 | establishment of organelle localization | 58/2334 | 415/29008 | 2.71E-05 | 0.0002214726244 | 0.0001246466508 |
| **1151** | KO | GO:0002691 | regulation of cellular extravasation | 13/2334 | 44/29008 | 2.76E-05 | 0.0002248841993 | 0.0001265667137 |
| **1152** | KO | GO:0045940 | positive regulation of steroid metabolic process | 13/2334 | 44/29008 | 2.76E-05 | 0.0002248841993 | 0.0001265667137 |
| **1153** | KO | GO:0030048 | actin filament-based movement | 25/2334 | 128/29008 | 2.79E-05 | 0.0002266818203 | 0.0001275784299 |
| **1154** | KO | GO:0009064 | glutamine family amino acid metabolic process | 16/2334 | 63/29008 | 2.80E-05 | 0.0002268137546 | 0.0001276526836 |
| **1155** | KO | GO:0032653 | regulation of interleukin-10 production | 16/2334 | 63/29008 | 2.80E-05 | 0.0002268137546 | 0.0001276526836 |
| **1156** | KO | GO:0003012 | muscle system process | 59/2334 | 425/29008 | 2.84E-05 | 0.0002295041363 | 0.000129166853 |
| **1157** | KO | GO:0051052 | regulation of DNA metabolic process | 55/2334 | 388/29008 | 2.91E-05 | 0.0002350847603 | 0.000132307675 |
| **1158** | KO | GO:2001057 | reactive nitrogen species metabolic process | 20/2334 | 91/29008 | 2.97E-05 | 0.0002398929013 | 0.0001350137371 |
| **1159** | KO | GO:1901216 | positive regulation of neuron death | 25/2334 | 129/29008 | 3.20E-05 | 0.0002578959863 | 0.0001451460244 |
| **1160** | KO | GO:1901991 | negative regulation of mitotic cell cycle phase transition | 30/2334 | 169/29008 | 3.24E-05 | 0.0002610299093 | 0.0001469098225 |
| **1161** | KO | GO:0009119 | ribonucleoside metabolic process | 11/2334 | 33/29008 | 3.27E-05 | 0.0002625916985 | 0.0001477888106 |
| **1162** | KO | GO:1903901 | negative regulation of viral life cycle | 11/2334 | 33/29008 | 3.27E-05 | 0.0002625916985 | 0.0001477888106 |
| **1163** | KO | GO:0050773 | regulation of dendrite development | 26/2334 | 137/29008 | 3.28E-05 | 0.0002629612395 | 0.0001479967914 |
| **1164** | KO | GO:0034976 | response to endoplasmic reticulum stress | 40/2334 | 254/29008 | 3.30E-05 | 0.0002645067694 | 0.0001488666285 |
| **1165** | KO | GO:0006417 | regulation of translation | 55/2334 | 390/29008 | 3.36E-05 | 0.0002683306699 | 0.0001510187518 |
| **1166** | KO | GO:0001892 | embryonic placenta development | 23/2334 | 114/29008 | 3.36E-05 | 0.0002683306699 | 0.0001510187518 |
| **1167** | KO | GO:0051209 | release of sequestered calcium ion into cytosol | 23/2334 | 114/29008 | 3.36E-05 | 0.0002683306699 | 0.0001510187518 |
| **1168** | KO | GO:0061756 | leukocyte adhesion to vascular endothelial cell | 14/2334 | 51/29008 | 3.43E-05 | 0.0002731524134 | 0.0001537324695 |
| **1169** | KO | GO:0046777 | protein autophosphorylation | 38/2334 | 237/29008 | 3.43E-05 | 0.0002731524134 | 0.0001537324695 |
| **1170** | KO | GO:0007584 | response to nutrient | 16/2334 | 64/29008 | 3.45E-05 | 0.0002745626987 | 0.0001545261899 |
| **1171** | KO | GO:0019932 | second-messenger-mediated signaling | 46/2334 | 308/29008 | 3.50E-05 | 0.0002777214862 | 0.000156303982 |
| **1172** | KO | GO:0035264 | multicellular organism growth | 36/2334 | 220/29008 | 3.51E-05 | 0.000278145311 | 0.0001565425142 |
| **1173** | KO | GO:0006469 | negative regulation of protein kinase activity | 34/2334 | 203/29008 | 3.51E-05 | 0.0002782070476 | 0.0001565772601 |
| **1174** | KO | GO:0071825 | protein-lipid complex subunit organization | 13/2334 | 45/29008 | 3.60E-05 | 0.000284397325 | 0.0001600612001 |
| **1175** | KO | GO:1905819 | negative regulation of chromosome separation | 13/2334 | 45/29008 | 3.60E-05 | 0.000284397325 | 0.0001600612001 |
| **1176** | KO | GO:1901606 | alpha-amino acid catabolic process | 17/2334 | 71/29008 | 3.63E-05 | 0.0002852819451 | 0.0001605590717 |
| **1177** | KO | GO:1990868 | response to chemokine | 17/2334 | 71/29008 | 3.63E-05 | 0.0002852819451 | 0.0001605590717 |
| **1178** | KO | GO:1990869 | cellular response to chemokine | 17/2334 | 71/29008 | 3.63E-05 | 0.0002852819451 | 0.0001605590717 |
| **1179** | KO | GO:0010934 | macrophage cytokine production | 8/2334 | 18/29008 | 3.64E-05 | 0.0002860152122 | 0.0001609717606 |
| **1180** | KO | GO:0043406 | positive regulation of MAP kinase activity | 25/2334 | 130/29008 | 3.66E-05 | 0.0002872790683 | 0.0001616830694 |
| **1181** | KO | GO:0045453 | bone resorption | 18/2334 | 78/29008 | 3.68E-05 | 0.0002882369798 | 0.00016222219 |
| **1182** | KO | GO:0006665 | sphingolipid metabolic process | 26/2334 | 138/29008 | 3.73E-05 | 0.0002922673628 | 0.0001644905234 |
| **1183** | KO | GO:0042572 | retinol metabolic process | 10/2334 | 28/29008 | 3.79E-05 | 0.0002959953096 | 0.0001665886431 |
| **1184** | KO | GO:0051283 | negative regulation of sequestering of calcium ion | 23/2334 | 115/29008 | 3.89E-05 | 0.0003034677855 | 0.0001707942152 |
| **1185** | KO | GO:0032092 | positive regulation of protein binding | 21/2334 | 100/29008 | 3.91E-05 | 0.000304863324 | 0.000171579636 |
| **1186** | KO | GO:0043030 | regulation of macrophage activation | 15/2334 | 58/29008 | 3.93E-05 | 0.0003053998544 | 0.0001718815998 |
| **1187** | KO | GO:0071715 | icosanoid transport | 15/2334 | 58/29008 | 3.93E-05 | 0.0003053998544 | 0.0001718815998 |
| **1188** | KO | GO:0046426 | negative regulation of receptor signaling pathway via JAK-STAT | 9/2334 | 23/29008 | 4.01E-05 | 0.000311324072 | 0.0001752157991 |
| **1189** | KO | GO:0030073 | insulin secretion | 37/2334 | 230/29008 | 4.02E-05 | 0.000311324072 | 0.0001752157991 |
| **1190** | KO | GO:0071559 | response to transforming growth factor beta | 37/2334 | 230/29008 | 4.02E-05 | 0.000311324072 | 0.0001752157991 |
| **1191** | KO | GO:0061640 | cytoskeleton-dependent cytokinesis | 20/2334 | 93/29008 | 4.14E-05 | 0.0003203318344 | 0.0001802854434 |
| **1192** | KO | GO:0022612 | gland morphogenesis | 28/2334 | 155/29008 | 4.23E-05 | 0.0003256892667 | 0.0001833006512 |
| **1193** | KO | GO:0015909 | long-chain fatty acid transport | 16/2334 | 65/29008 | 4.24E-05 | 0.0003256892667 | 0.0001833006512 |
| **1194** | KO | GO:0032613 | interleukin-10 production | 16/2334 | 65/29008 | 4.24E-05 | 0.0003256892667 | 0.0001833006512 |
| **1195** | KO | GO:0043467 | regulation of generation of precursor metabolites and energy | 27/2334 | 147/29008 | 4.26E-05 | 0.0003256892667 | 0.0001833006512 |
| **1196** | KO | GO:0002223 | stimulatory C-type lectin receptor signaling pathway | 6/2334 | 10/29008 | 4.26E-05 | 0.0003256892667 | 0.0001833006512 |
| **1197** | KO | GO:0002357 | defense response to tumor cell | 6/2334 | 10/29008 | 4.26E-05 | 0.0003256892667 | 0.0001833006512 |
| **1198** | KO | GO:0035744 | T-helper 1 cell cytokine production | 6/2334 | 10/29008 | 4.26E-05 | 0.0003256892667 | 0.0001833006512 |
| **1199** | KO | GO:1905709 | negative regulation of membrane permeability | 6/2334 | 10/29008 | 4.26E-05 | 0.0003256892667 | 0.0001833006512 |
| **1200** | KO | GO:1990840 | response to lectin | 6/2334 | 10/29008 | 4.26E-05 | 0.0003256892667 | 0.0001833006512 |
| **1201** | KO | GO:1990858 | cellular response to lectin | 6/2334 | 10/29008 | 4.26E-05 | 0.0003256892667 | 0.0001833006512 |
| **1202** | KO | GO:2000300 | regulation of synaptic vesicle exocytosis | 19/2334 | 86/29008 | 4.31E-05 | 0.0003292632037 | 0.0001853120929 |
| **1203** | KO | GO:0007059 | chromosome segregation | 49/2334 | 338/29008 | 4.34E-05 | 0.0003306828601 | 0.0001861110875 |
| **1204** | KO | GO:0009914 | hormone transport | 53/2334 | 375/29008 | 4.36E-05 | 0.000331768352 | 0.0001867220114 |
| **1205** | KO | GO:0045428 | regulation of nitric oxide biosynthetic process | 17/2334 | 72/29008 | 4.39E-05 | 0.0003341503994 | 0.0001880626477 |
| **1206** | KO | GO:0052547 | regulation of peptidase activity | 60/2334 | 441/29008 | 4.42E-05 | 0.0003353193787 | 0.000188720559 |
| **1207** | KO | GO:0009173 | pyrimidine ribonucleoside monophosphate metabolic process | 7/2334 | 14/29008 | 4.47E-05 | 0.0003375392243 | 0.0001899699067 |
| **1208** | KO | GO:0015867 | ATP transport | 7/2334 | 14/29008 | 4.47E-05 | 0.0003375392243 | 0.0001899699067 |
| **1209** | KO | GO:0046049 | UMP metabolic process | 7/2334 | 14/29008 | 4.47E-05 | 0.0003375392243 | 0.0001899699067 |
| **1210** | KO | GO:0002790 | peptide secretion | 44/2334 | 293/29008 | 4.48E-05 | 0.0003375392243 | 0.0001899699067 |
| **1211** | KO | GO:0035335 | peptidyl-tyrosine dephosphorylation | 11/2334 | 34/29008 | 4.48E-05 | 0.0003375392243 | 0.0001899699067 |
| **1212** | KO | GO:0042908 | xenobiotic transport | 11/2334 | 34/29008 | 4.48E-05 | 0.0003375392243 | 0.0001899699067 |
| **1213** | KO | GO:0090322 | regulation of superoxide metabolic process | 11/2334 | 34/29008 | 4.48E-05 | 0.0003375392243 | 0.0001899699067 |
| **1214** | KO | GO:0033047 | regulation of mitotic sister chromatid segregation | 13/2334 | 46/29008 | 4.65E-05 | 0.0003489956385 | 0.0001964176728 |
| **1215** | KO | GO:0051985 | negative regulation of chromosome segregation | 13/2334 | 46/29008 | 4.65E-05 | 0.0003489956385 | 0.0001964176728 |
| **1216** | KO | GO:1900077 | negative regulation of cellular response to insulin stimulus | 13/2334 | 46/29008 | 4.65E-05 | 0.0003489956385 | 0.0001964176728 |
| **1217** | KO | GO:0000280 | nuclear division | 60/2334 | 442/29008 | 4.72E-05 | 0.0003533319786 | 0.0001988582014 |
| **1218** | KO | GO:0000302 | response to reactive oxygen species | 32/2334 | 189/29008 | 4.74E-05 | 0.0003544311759 | 0.0001994768388 |
| **1219** | KO | GO:0046148 | pigment biosynthetic process | 15/2334 | 59/29008 | 4.88E-05 | 0.0003645061166 | 0.0002051470999 |
| **1220** | KO | GO:0010632 | regulation of epithelial cell migration | 38/2334 | 241/29008 | 4.99E-05 | 0.0003722321538 | 0.0002094953785 |
| **1221** | KO | GO:0018105 | peptidyl-serine phosphorylation | 49/2334 | 340/29008 | 5.05E-05 | 0.0003766098193 | 0.0002119591654 |
| **1222** | KO | GO:0048017 | inositol lipid-mediated signaling | 30/2334 | 173/29008 | 5.10E-05 | 0.0003799349727 | 0.0002138305896 |
| **1223** | KO | GO:0090307 | mitotic spindle assembly | 16/2334 | 66/29008 | 5.19E-05 | 0.0003857101365 | 0.0002170809002 |
| **1224** | KO | GO:2000107 | negative regulation of leukocyte apoptotic process | 17/2334 | 73/29008 | 5.30E-05 | 0.000393961455 | 0.0002217248115 |
| **1225** | KO | GO:0003158 | endothelium development | 25/2334 | 133/29008 | 5.43E-05 | 0.000402602411 | 0.000226588014 |
| **1226** | KO | GO:0033559 | unsaturated fatty acid metabolic process | 25/2334 | 133/29008 | 5.43E-05 | 0.000402602411 | 0.000226588014 |
| **1227** | KO | GO:0000910 | cytokinesis | 31/2334 | 182/29008 | 5.49E-05 | 0.0004063429438 | 0.0002286932173 |
| **1228** | KO | GO:0007517 | muscle organ development | 51/2334 | 360/29008 | 5.65E-05 | 0.0004177854851 | 0.0002351331755 |
| **1229** | KO | GO:0002526 | acute inflammatory response | 22/2334 | 110/29008 | 5.67E-05 | 0.0004188944757 | 0.0002357573247 |
| **1230** | KO | GO:0030833 | regulation of actin filament polymerization | 30/2334 | 174/29008 | 5.70E-05 | 0.0004191826523 | 0.0002359195129 |
| **1231** | KO | GO:0071356 | cellular response to tumor necrosis factor | 30/2334 | 174/29008 | 5.70E-05 | 0.0004191826523 | 0.0002359195129 |
| **1232** | KO | GO:0072522 | purine-containing compound biosynthetic process | 30/2334 | 174/29008 | 5.70E-05 | 0.0004191826523 | 0.0002359195129 |
| **1233** | KO | GO:0032271 | regulation of protein polymerization | 37/2334 | 234/29008 | 5.85E-05 | 0.0004300997267 | 0.0002420637339 |
| **1234** | KO | GO:0001961 | positive regulation of cytokine-mediated signaling pathway | 13/2334 | 47/29008 | 5.96E-05 | 0.0004356232568 | 0.000245172423 |
| **1235** | KO | GO:0120163 | negative regulation of cold-induced thermogenesis | 13/2334 | 47/29008 | 5.96E-05 | 0.0004356232568 | 0.000245172423 |
| **1236** | KO | GO:0015732 | prostaglandin transport | 9/2334 | 24/29008 | 5.96E-05 | 0.0004356232568 | 0.000245172423 |
| **1237** | KO | GO:0035743 | CD4-positive, alpha-beta T cell cytokine production | 9/2334 | 24/29008 | 5.96E-05 | 0.0004356232568 | 0.000245172423 |
| **1238** | KO | GO:0070498 | interleukin-1-mediated signaling pathway | 9/2334 | 24/29008 | 5.96E-05 | 0.0004356232568 | 0.000245172423 |
| **1239** | KO | GO:0033003 | regulation of mast cell activation | 15/2334 | 60/29008 | 6.02E-05 | 0.0004394403515 | 0.0002473207159 |
| **1240** | KO | GO:0042176 | regulation of protein catabolic process | 54/2334 | 389/29008 | 6.06E-05 | 0.0004414409395 | 0.0002484466637 |
| **1241** | KO | GO:0014002 | astrocyte development | 11/2334 | 35/29008 | 6.06E-05 | 0.0004414409395 | 0.0002484466637 |
| **1242** | KO | GO:0002347 | response to tumor cell | 12/2334 | 41/29008 | 6.21E-05 | 0.0004502891523 | 0.000253426512 |
| **1243** | KO | GO:0042092 | type 2 immune response | 12/2334 | 41/29008 | 6.21E-05 | 0.0004502891523 | 0.000253426512 |
| **1244** | KO | GO:0046627 | negative regulation of insulin receptor signaling pathway | 12/2334 | 41/29008 | 6.21E-05 | 0.0004502891523 | 0.000253426512 |
| **1245** | KO | GO:0071560 | cellular response to transforming growth factor beta stimulus | 36/2334 | 226/29008 | 6.25E-05 | 0.0004531681106 | 0.0002550468139 |
| **1246** | KO | GO:0007622 | rhythmic behavior | 16/2334 | 67/29008 | 6.31E-05 | 0.0004564818039 | 0.0002569117883 |
| **1247** | KO | GO:0002791 | regulation of peptide secretion | 37/2334 | 235/29008 | 6.42E-05 | 0.0004637823979 | 0.0002610206238 |
| **1248** | KO | GO:0002709 | regulation of T cell mediated immunity | 22/2334 | 111/29008 | 6.54E-05 | 0.0004724294668 | 0.0002658872667 |
| **1249** | KO | GO:0043393 | regulation of protein binding | 35/2334 | 218/29008 | 6.67E-05 | 0.0004808661041 | 0.0002706354769 |
| **1250** | KO | GO:0060537 | muscle tissue development | 64/2334 | 486/29008 | 6.73E-05 | 0.0004850876978 | 0.0002730114253 |
| **1251** | KO | GO:0032963 | collagen metabolic process | 23/2334 | 119/29008 | 6.80E-05 | 0.0004889303861 | 0.0002751741225 |
| **1252** | KO | GO:0009161 | ribonucleoside monophosphate metabolic process | 14/2334 | 54/29008 | 6.85E-05 | 0.0004922208019 | 0.0002770259961 |
| **1253** | KO | GO:0032386 | regulation of intracellular transport | 48/2334 | 335/29008 | 6.93E-05 | 0.0004975052685 | 0.0002800001383 |
| **1254** | KO | GO:0007272 | ensheathment of neurons | 30/2334 | 176/29008 | 7.08E-05 | 0.0005069089148 | 0.0002852925893 |
| **1255** | KO | GO:0008366 | axon ensheathment | 30/2334 | 176/29008 | 7.08E-05 | 0.0005069089148 | 0.0002852925893 |
| **1256** | KO | GO:0046683 | response to organophosphorus | 19/2334 | 89/29008 | 7.09E-05 | 0.0005072238487 | 0.0002854698367 |
| **1257** | KO | GO:0060402 | calcium ion transport into cytosol | 29/2334 | 168/29008 | 7.34E-05 | 0.0005247009822 | 0.000295306114 |
| **1258** | KO | GO:0046470 | phosphatidylcholine metabolic process | 15/2334 | 61/29008 | 7.40E-05 | 0.0005266053905 | 0.0002963779311 |
| **1259** | KO | GO:0070098 | chemokine-mediated signaling pathway | 15/2334 | 61/29008 | 7.40E-05 | 0.0005266053905 | 0.0002963779311 |
| **1260** | KO | GO:1903409 | reactive oxygen species biosynthetic process | 15/2334 | 61/29008 | 7.40E-05 | 0.0005266053905 | 0.0002963779311 |
| **1261** | KO | GO:0006140 | regulation of nucleotide metabolic process | 18/2334 | 82/29008 | 7.43E-05 | 0.0005282833803 | 0.0002973223178 |
| **1262** | KO | GO:0006779 | porphyrin-containing compound biosynthetic process | 10/2334 | 30/29008 | 7.47E-05 | 0.0005291622606 | 0.000297816959 |
| **1263** | KO | GO:0033014 | tetrapyrrole biosynthetic process | 10/2334 | 30/29008 | 7.47E-05 | 0.0005291622606 | 0.000297816959 |
| **1264** | KO | GO:0046597 | negative regulation of viral entry into host cell | 10/2334 | 30/29008 | 7.47E-05 | 0.0005291622606 | 0.000297816959 |
| **1265** | KO | GO:0000959 | mitochondrial RNA metabolic process | 13/2334 | 48/29008 | 7.58E-05 | 0.000534985558 | 0.0003010943596 |
| **1266** | KO | GO:0048246 | macrophage chemotaxis | 13/2334 | 48/29008 | 7.58E-05 | 0.000534985558 | 0.0003010943596 |
| **1267** | KO | GO:0050856 | regulation of T cell receptor signaling pathway | 13/2334 | 48/29008 | 7.58E-05 | 0.000534985558 | 0.0003010943596 |
| **1268** | KO | GO:0032233 | positive regulation of actin filament bundle assembly | 16/2334 | 68/29008 | 7.64E-05 | 0.0005387883471 | 0.0003032346012 |
| **1269** | KO | GO:0090087 | regulation of peptide transport | 37/2334 | 237/29008 | 7.69E-05 | 0.0005421475588 | 0.0003051251937 |
| **1270** | KO | GO:0060700 | regulation of ribonuclease activity | 7/2334 | 15/29008 | 7.80E-05 | 0.0005484764163 | 0.0003086871278 |
| **1271** | KO | GO:1902563 | regulation of neutrophil activation | 7/2334 | 15/29008 | 7.80E-05 | 0.0005484764163 | 0.0003086871278 |
| **1272** | KO | GO:0090316 | positive regulation of intracellular protein transport | 26/2334 | 144/29008 | 7.88E-05 | 0.0005535356491 | 0.0003115345065 |
| **1273** | KO | GO:0033002 | muscle cell proliferation | 39/2334 | 255/29008 | 7.90E-05 | 0.0005538676746 | 0.0003117213732 |
| **1274** | KO | GO:0014013 | regulation of gliogenesis | 24/2334 | 128/29008 | 7.91E-05 | 0.0005538676746 | 0.0003117213732 |
| **1275** | KO | GO:0010948 | negative regulation of cell cycle process | 42/2334 | 282/29008 | 7.97E-05 | 0.0005579007297 | 0.0003139912104 |
| **1276** | KO | GO:0045841 | negative regulation of mitotic metaphase/anaphase transition | 12/2334 | 42/29008 | 8.06E-05 | 0.0005632246708 | 0.0003169875692 |
| **1277** | KO | GO:0060338 | regulation of type I interferon-mediated signaling pathway | 11/2334 | 36/29008 | 8.10E-05 | 0.0005655381722 | 0.0003182896271 |
| **1278** | KO | GO:0032388 | positive regulation of intracellular transport | 31/2334 | 186/29008 | 8.36E-05 | 0.000582033494 | 0.0003275733326 |
| **1279** | KO | GO:0050796 | regulation of insulin secretion | 31/2334 | 186/29008 | 8.36E-05 | 0.000582033494 | 0.0003275733326 |
| **1280** | KO | GO:0030100 | regulation of endocytosis | 37/2334 | 238/29008 | 8.42E-05 | 0.0005856291842 | 0.0003295970172 |
| **1281** | KO | GO:0022898 | regulation of transmembrane transporter activity | 42/2334 | 283/29008 | 8.65E-05 | 0.0005986934267 | 0.0003369496825 |
| **1282** | KO | GO:0018904 | ether metabolic process | 9/2334 | 25/29008 | 8.65E-05 | 0.0005986934267 | 0.0003369496825 |
| **1283** | KO | GO:0032753 | positive regulation of interleukin-4 production | 9/2334 | 25/29008 | 8.65E-05 | 0.0005986934267 | 0.0003369496825 |
| **1284** | KO | GO:0044273 | sulfur compound catabolic process | 9/2334 | 25/29008 | 8.65E-05 | 0.0005986934267 | 0.0003369496825 |
| **1285** | KO | GO:1904893 | negative regulation of receptor signaling pathway via STAT | 9/2334 | 25/29008 | 8.65E-05 | 0.0005986934267 | 0.0003369496825 |
| **1286** | KO | GO:0009222 | pyrimidine ribonucleotide catabolic process | 6/2334 | 11/29008 | 8.74E-05 | 0.0006030196326 | 0.0003393845075 |
| **1287** | KO | GO:0032959 | inositol trisphosphate biosynthetic process | 6/2334 | 11/29008 | 8.74E-05 | 0.0006030196326 | 0.0003393845075 |
| **1288** | KO | GO:0048660 | regulation of smooth muscle cell proliferation | 30/2334 | 178/29008 | 8.76E-05 | 0.000603775668 | 0.0003398100105 |
| **1289** | KO | GO:0070373 | negative regulation of ERK1 and ERK2 cascade | 18/2334 | 83/29008 | 8.78E-05 | 0.000604778205 | 0.0003403742468 |
| **1290** | KO | GO:0008652 | cellular amino acid biosynthetic process | 15/2334 | 62/29008 | 9.04E-05 | 0.0006200966706 | 0.0003489956077 |
| **1291** | KO | GO:0010965 | regulation of mitotic sister chromatid separation | 15/2334 | 62/29008 | 9.04E-05 | 0.0006200966706 | 0.0003489956077 |
| **1292** | KO | GO:0016126 | sterol biosynthetic process | 15/2334 | 62/29008 | 9.04E-05 | 0.0006200966706 | 0.0003489956077 |
| **1293** | KO | GO:1904062 | regulation of cation transmembrane transport | 52/2334 | 376/29008 | 9.04E-05 | 0.0006200966706 | 0.0003489956077 |
| **1294** | KO | GO:0032310 | prostaglandin secretion | 8/2334 | 20/29008 | 9.05E-05 | 0.0006200966706 | 0.0003489956077 |
| **1295** | KO | GO:0002704 | negative regulation of leukocyte mediated immunity | 17/2334 | 76/29008 | 9.10E-05 | 0.0006224810338 | 0.0003503375473 |
| **1296** | KO | GO:0030183 | B cell differentiation | 29/2334 | 170/29008 | 9.13E-05 | 0.0006233796385 | 0.0003508432895 |
| **1297** | KO | GO:1903531 | negative regulation of secretion by cell | 29/2334 | 170/29008 | 9.13E-05 | 0.0006233796385 | 0.0003508432895 |
| **1298** | KO | GO:0006470 | protein dephosphorylation | 37/2334 | 239/29008 | 9.20E-05 | 0.0006258281342 | 0.0003522213235 |
| **1299** | KO | GO:0032507 | maintenance of protein location in cell | 16/2334 | 69/29008 | 9.21E-05 | 0.0006258281342 | 0.0003522213235 |
| **1300** | KO | GO:0033045 | regulation of sister chromatid segregation | 16/2334 | 69/29008 | 9.21E-05 | 0.0006258281342 | 0.0003522213235 |
| **1301** | KO | GO:1903556 | negative regulation of tumor necrosis factor superfamily cytokine production | 16/2334 | 69/29008 | 9.21E-05 | 0.0006258281342 | 0.0003522213235 |
| **1302** | KO | GO:0001505 | regulation of neurotransmitter levels | 39/2334 | 257/29008 | 9.38E-05 | 0.0006367606406 | 0.0003583742298 |
| **1303** | KO | GO:0002064 | epithelial cell development | 40/2334 | 266/29008 | 9.41E-05 | 0.0006381867603 | 0.0003591768619 |
| **1304** | KO | GO:0045930 | negative regulation of mitotic cell cycle | 36/2334 | 231/29008 | 9.90E-05 | 0.0006705163425 | 0.000377372222 |
| **1305** | KO | GO:0090068 | positive regulation of cell cycle process | 38/2334 | 249/29008 | 0.0001014884642 | 0.0006861520004 | 0.0003861721014 |
| **1306** | KO | GO:0030072 | peptide hormone secretion | 42/2334 | 285/29008 | 0.0001015156524 | 0.0006861520004 | 0.0003861721014 |
| **1307** | KO | GO:1903034 | regulation of response to wounding | 29/2334 | 171/29008 | 0.0001016388048 | 0.0006862294686 | 0.0003862157011 |
| **1308** | KO | GO:0072574 | hepatocyte proliferation | 10/2334 | 31/29008 | 0.0001023107305 | 0.0006892512372 | 0.0003879163779 |
| **1309** | KO | GO:0072575 | epithelial cell proliferation involved in liver morphogenesis | 10/2334 | 31/29008 | 0.0001023107305 | 0.0006892512372 | 0.0003879163779 |
| **1310** | KO | GO:0006809 | nitric oxide biosynthetic process | 18/2334 | 84/29008 | 0.0001034363146 | 0.0006943789275 | 0.0003908022849 |
| **1311** | KO | GO:0046461 | neutral lipid catabolic process | 12/2334 | 43/29008 | 0.0001036369591 | 0.0006943789275 | 0.0003908022849 |
| **1312** | KO | GO:0046464 | acylglycerol catabolic process | 12/2334 | 43/29008 | 0.0001036369591 | 0.0006943789275 | 0.0003908022849 |
| **1313** | KO | GO:0097300 | programmed necrotic cell death | 12/2334 | 43/29008 | 0.0001036369591 | 0.0006943789275 | 0.0003908022849 |
| **1314** | KO | GO:1990748 | cellular detoxification | 12/2334 | 43/29008 | 0.0001036369591 | 0.0006943789275 | 0.0003908022849 |
| **1315** | KO | GO:0001974 | blood vessel remodeling | 14/2334 | 56/29008 | 0.0001051757471 | 0.0007023910763 | 0.0003953115895 |
| **1316** | KO | GO:0006695 | cholesterol biosynthetic process | 14/2334 | 56/29008 | 0.0001051757471 | 0.0007023910763 | 0.0003953115895 |
| **1317** | KO | GO:1902653 | secondary alcohol biosynthetic process | 14/2334 | 56/29008 | 0.0001051757471 | 0.0007023910763 | 0.0003953115895 |
| **1318** | KO | GO:0002335 | mature B cell differentiation | 11/2334 | 37/29008 | 0.0001069331306 | 0.0007125782588 | 0.0004010450213 |
| **1319** | KO | GO:0009069 | serine family amino acid metabolic process | 11/2334 | 37/29008 | 0.0001069331306 | 0.0007125782588 | 0.0004010450213 |
| **1320** | KO | GO:0048146 | positive regulation of fibroblast proliferation | 16/2334 | 70/29008 | 0.000110536318 | 0.0007349947377 | 0.0004136612036 |
| **1321** | KO | GO:2000514 | regulation of CD4-positive, alpha-beta T cell activation | 16/2334 | 70/29008 | 0.000110536318 | 0.0007349947377 | 0.0004136612036 |
| **1322** | KO | GO:0032535 | regulation of cellular component size | 56/2334 | 417/29008 | 0.0001106825995 | 0.0007351717745 | 0.0004137608414 |
| **1323** | KO | GO:0043367 | CD4-positive, alpha-beta T cell differentiation | 19/2334 | 92/29008 | 0.0001134800947 | 0.0007505077524 | 0.0004223920584 |
| **1324** | KO | GO:0097581 | lamellipodium organization | 19/2334 | 92/29008 | 0.0001134800947 | 0.0007505077524 | 0.0004223920584 |
| **1325** | KO | GO:1900182 | positive regulation of protein localization to nucleus | 19/2334 | 92/29008 | 0.0001134800947 | 0.0007505077524 | 0.0004223920584 |
| **1326** | KO | GO:1990542 | mitochondrial transmembrane transport | 19/2334 | 92/29008 | 0.0001134800947 | 0.0007505077524 | 0.0004223920584 |
| **1327** | KO | GO:0010906 | regulation of glucose metabolic process | 24/2334 | 131/29008 | 0.0001153324552 | 0.0007619382849 | 0.000428825258 |
| **1328** | KO | GO:0008643 | carbohydrate transport | 27/2334 | 156/29008 | 0.0001221754159 | 0.0008062790069 | 0.0004537805883 |
| **1329** | KO | GO:0010742 | macrophage derived foam cell differentiation | 9/2334 | 26/29008 | 0.0001229168882 | 0.0008103018893 | 0.0004560447002 |
| **1330** | KO | GO:0042552 | myelination | 29/2334 | 173/29008 | 0.000125478982 | 0.0008263053219 | 0.0004650515664 |
| **1331** | KO | GO:0071901 | negative regulation of protein serine/threonine kinase activity | 21/2334 | 108/29008 | 0.0001263361349 | 0.0008310591145 | 0.0004677270407 |
| **1332** | KO | GO:0030900 | forebrain development | 54/2334 | 400/29008 | 0.0001267707118 | 0.0008330259392 | 0.0004688339861 |
| **1333** | KO | GO:0032409 | regulation of transporter activity | 43/2334 | 297/29008 | 0.0001270213002 | 0.0008337808427 | 0.0004692588521 |
| **1334** | KO | GO:0002292 | T cell differentiation involved in immune response | 17/2334 | 78/29008 | 0.0001279724098 | 0.0008364494533 | 0.0004707607686 |
| **1335** | KO | GO:0042306 | regulation of protein import into nucleus | 17/2334 | 78/29008 | 0.0001279724098 | 0.0008364494533 | 0.0004707607686 |
| **1336** | KO | GO:0048002 | antigen processing and presentation of peptide antigen | 17/2334 | 78/29008 | 0.0001279724098 | 0.0008364494533 | 0.0004707607686 |
| **1337** | KO | GO:0071277 | cellular response to calcium ion | 17/2334 | 78/29008 | 0.0001279724098 | 0.0008364494533 | 0.0004707607686 |
| **1338** | KO | GO:0032604 | granulocyte macrophage colony-stimulating factor production | 7/2334 | 16/29008 | 0.0001290630978 | 0.0008399025777 | 0.000472704216 |
| **1339** | KO | GO:0042454 | ribonucleoside catabolic process | 7/2334 | 16/29008 | 0.0001290630978 | 0.0008399025777 | 0.000472704216 |
| **1340** | KO | GO:0072673 | lamellipodium morphogenesis | 7/2334 | 16/29008 | 0.0001290630978 | 0.0008399025777 | 0.000472704216 |
| **1341** | KO | GO:0009395 | phospholipid catabolic process | 14/2334 | 57/29008 | 0.0001291842344 | 0.0008399025777 | 0.000472704216 |
| **1342** | KO | GO:0048634 | regulation of muscle organ development | 14/2334 | 57/29008 | 0.0001291842344 | 0.0008399025777 | 0.000472704216 |
| **1343** | KO | GO:0060348 | bone development | 37/2334 | 243/29008 | 0.00013031457 | 0.0008463559385 | 0.0004763362216 |
| **1344** | KO | GO:0071383 | cellular response to steroid hormone stimulus | 28/2334 | 165/29008 | 0.0001311867466 | 0.0008511207719 | 0.0004790179098 |
| **1345** | KO | GO:1902100 | negative regulation of metaphase/anaphase transition of cell cycle | 12/2334 | 44/29008 | 0.0001321378078 | 0.0008563868051 | 0.0004819816774 |
| **1346** | KO | GO:0048512 | circadian behavior | 15/2334 | 64/29008 | 0.0001329389336 | 0.0008606710308 | 0.0004843928756 |
| **1347** | KO | GO:0032869 | cellular response to insulin stimulus | 33/2334 | 208/29008 | 0.000133723227 | 0.0008639279777 | 0.0004862259126 |
| **1348** | KO | GO:1902905 | positive regulation of supramolecular fiber organization | 33/2334 | 208/29008 | 0.000133723227 | 0.0008639279777 | 0.0004862259126 |
| **1349** | KO | GO:0002577 | regulation of antigen processing and presentation | 8/2334 | 21/29008 | 0.0001359417368 | 0.0008736673966 | 0.0004917073393 |
| **1350** | KO | GO:0006662 | glycerol ether metabolic process | 8/2334 | 21/29008 | 0.0001359417368 | 0.0008736673966 | 0.0004917073393 |
| **1351** | KO | GO:0007252 | I-kappaB phosphorylation | 8/2334 | 21/29008 | 0.0001359417368 | 0.0008736673966 | 0.0004917073393 |
| **1352** | KO | GO:0046321 | positive regulation of fatty acid oxidation | 8/2334 | 21/29008 | 0.0001359417368 | 0.0008736673966 | 0.0004917073393 |
| **1353** | KO | GO:0051503 | adenine nucleotide transport | 8/2334 | 21/29008 | 0.0001359417368 | 0.0008736673966 | 0.0004917073393 |
| **1354** | KO | GO:0071322 | cellular response to carbohydrate stimulus | 27/2334 | 157/29008 | 0.000136409491 | 0.0008757574845 | 0.0004928836583 |
| **1355** | KO | GO:0000096 | sulfur amino acid metabolic process | 10/2334 | 32/29008 | 0.0001381329562 | 0.0008840509199 | 0.0004975512733 |
| **1356** | KO | GO:0032673 | regulation of interleukin-4 production | 10/2334 | 32/29008 | 0.0001381329562 | 0.0008840509199 | 0.0004975512733 |
| **1357** | KO | GO:0043372 | positive regulation of CD4-positive, alpha-beta T cell differentiation | 10/2334 | 32/29008 | 0.0001381329562 | 0.0008840509199 | 0.0004975512733 |
| **1358** | KO | GO:0033673 | negative regulation of kinase activity | 35/2334 | 226/29008 | 0.0001392284986 | 0.0008901351671 | 0.0005009755387 |
| **1359** | KO | GO:0030168 | platelet activation | 18/2334 | 86/29008 | 0.0001422001419 | 0.000907982866 | 0.0005110203733 |
| **1360** | KO | GO:0034764 | positive regulation of transmembrane transport | 38/2334 | 253/29008 | 0.0001423156738 | 0.000907982866 | 0.0005110203733 |
| **1361** | KO | GO:0050808 | synapse organization | 63/2334 | 489/29008 | 0.0001433184447 | 0.0009134320794 | 0.0005140872363 |
| **1362** | KO | GO:0008637 | apoptotic mitochondrial changes | 22/2334 | 117/29008 | 0.0001474970951 | 0.000939090313 | 0.0005285279054 |
| **1363** | KO | GO:0018209 | peptidyl-serine modification | 50/2334 | 365/29008 | 0.0001545882225 | 0.0009832195024 | 0.0005533641834 |
| **1364** | KO | GO:0009152 | purine ribonucleotide biosynthetic process | 26/2334 | 150/29008 | 0.0001576560145 | 0.0009974125022 | 0.0005613521227 |
| **1365** | KO | GO:0072073 | kidney epithelium development | 26/2334 | 150/29008 | 0.0001576560145 | 0.0009974125022 | 0.0005613521227 |
| **1366** | KO | GO:1902806 | regulation of cell cycle G1/S phase transition | 26/2334 | 150/29008 | 0.0001576560145 | 0.0009974125022 | 0.0005613521227 |
| **1367** | KO | GO:0030071 | regulation of mitotic metaphase/anaphase transition | 14/2334 | 58/29008 | 0.0001577937748 | 0.0009974125022 | 0.0005613521227 |
| **1368** | KO | GO:0043470 | regulation of carbohydrate catabolic process | 14/2334 | 58/29008 | 0.0001577937748 | 0.0009974125022 | 0.0005613521227 |
| **1369** | KO | GO:0045058 | T cell selection | 14/2334 | 58/29008 | 0.0001577937748 | 0.0009974125022 | 0.0005613521227 |
| **1370** | KO | GO:0051306 | mitotic sister chromatid separation | 15/2334 | 65/29008 | 0.0001600924478 | 0.001010902363 | 0.0005689443295 |
| **1371** | KO | GO:0048659 | smooth muscle cell proliferation | 30/2334 | 184/29008 | 0.0001613807052 | 0.001017990814 | 0.000572933769 |
| **1372** | KO | GO:0071248 | cellular response to metal ion | 28/2334 | 167/29008 | 0.0001620185687 | 0.00101842817 | 0.0005731799166 |
| **1373** | KO | GO:0001781 | neutrophil apoptotic process | 6/2334 | 12/29008 | 0.000162776117 | 0.00101842817 | 0.0005731799166 |
| **1374** | KO | GO:0002246 | wound healing involved in inflammatory response | 6/2334 | 12/29008 | 0.000162776117 | 0.00101842817 | 0.0005731799166 |
| **1375** | KO | GO:0006213 | pyrimidine nucleoside metabolic process | 6/2334 | 12/29008 | 0.000162776117 | 0.00101842817 | 0.0005731799166 |
| **1376** | KO | GO:0043301 | negative regulation of leukocyte degranulation | 6/2334 | 12/29008 | 0.000162776117 | 0.00101842817 | 0.0005731799166 |
| **1377** | KO | GO:0043313 | regulation of neutrophil degranulation | 6/2334 | 12/29008 | 0.000162776117 | 0.00101842817 | 0.0005731799166 |
| **1378** | KO | GO:0051132 | NK T cell activation | 6/2334 | 12/29008 | 0.000162776117 | 0.00101842817 | 0.0005731799166 |
| **1379** | KO | GO:0060907 | positive regulation of macrophage cytokine production | 6/2334 | 12/29008 | 0.000162776117 | 0.00101842817 | 0.0005731799166 |
| **1380** | KO | GO:0005976 | polysaccharide metabolic process | 21/2334 | 110/29008 | 0.0001655518578 | 0.001034741215 | 0.0005823610354 |
| **1381** | KO | GO:0021675 | nerve development | 18/2334 | 87/29008 | 0.0001659723902 | 0.001036315412 | 0.0005832470065 |
| **1382** | KO | GO:0042771 | intrinsic apoptotic signaling pathway in response to DNA damage by p53 class mediator | 12/2334 | 45/29008 | 0.0001670924463 | 0.001042249736 | 0.0005865868942 |
| **1383** | KO | GO:0001780 | neutrophil homeostasis | 9/2334 | 27/29008 | 0.000171259461 | 0.001064998106 | 0.0005993898674 |
| **1384** | KO | GO:0033198 | response to ATP | 9/2334 | 27/29008 | 0.000171259461 | 0.001064998106 | 0.0005993898674 |
| **1385** | KO | GO:0046128 | purine ribonucleoside metabolic process | 9/2334 | 27/29008 | 0.000171259461 | 0.001064998106 | 0.0005993898674 |
| **1386** | KO | GO:0030010 | establishment of cell polarity | 26/2334 | 151/29008 | 0.0001760933933 | 0.001093951272 | 0.0006156849524 |
| **1387** | KO | GO:0046879 | hormone secretion | 50/2334 | 367/29008 | 0.0001767419981 | 0.001096871552 | 0.00061732851 |
| **1388** | KO | GO:0038034 | signal transduction in absence of ligand | 17/2334 | 80/29008 | 0.0001775073956 | 0.001098293493 | 0.0006181287906 |
| **1389** | KO | GO:0097192 | extrinsic apoptotic signaling pathway in absence of ligand | 17/2334 | 80/29008 | 0.0001775073956 | 0.001098293493 | 0.0006181287906 |
| **1390** | KO | GO:1900076 | regulation of cellular response to insulin stimulus | 17/2334 | 80/29008 | 0.0001775073956 | 0.001098293493 | 0.0006181287906 |
| **1391** | KO | GO:0006164 | purine nucleotide biosynthetic process | 28/2334 | 168/29008 | 0.0001797333877 | 0.001109626716 | 0.0006245072237 |
| **1392** | KO | GO:0001773 | myeloid dendritic cell activation | 11/2334 | 39/29008 | 0.0001804227034 | 0.001109626716 | 0.0006245072237 |
| **1393** | KO | GO:0032506 | cytokinetic process | 11/2334 | 39/29008 | 0.0001804227034 | 0.001109626716 | 0.0006245072237 |
| **1394** | KO | GO:0032633 | interleukin-4 production | 11/2334 | 39/29008 | 0.0001804227034 | 0.001109626716 | 0.0006245072237 |
| **1395** | KO | GO:0032743 | positive regulation of interleukin-2 production | 11/2334 | 39/29008 | 0.0001804227034 | 0.001109626716 | 0.0006245072237 |
| **1396** | KO | GO:1904645 | response to amyloid-beta | 11/2334 | 39/29008 | 0.0001804227034 | 0.001109626716 | 0.0006245072237 |
| **1397** | KO | GO:0006898 | receptor-mediated endocytosis | 39/2334 | 265/29008 | 0.000181034679 | 0.001112277068 | 0.0006259988637 |
| **1398** | KO | GO:0002230 | positive regulation of defense response to virus by host | 10/2334 | 33/29008 | 0.0001839752225 | 0.001119156952 | 0.0006298709205 |
| **1399** | KO | GO:0002478 | antigen processing and presentation of exogenous peptide antigen | 10/2334 | 33/29008 | 0.0001839752225 | 0.001119156952 | 0.0006298709205 |
| **1400** | KO | GO:0002828 | regulation of type 2 immune response | 10/2334 | 33/29008 | 0.0001839752225 | 0.001119156952 | 0.0006298709205 |
| **1401** | KO | GO:0033032 | regulation of myeloid cell apoptotic process | 10/2334 | 33/29008 | 0.0001839752225 | 0.001119156952 | 0.0006298709205 |
| **1402** | KO | GO:0044275 | cellular carbohydrate catabolic process | 10/2334 | 33/29008 | 0.0001839752225 | 0.001119156952 | 0.0006298709205 |
| **1403** | KO | GO:0072576 | liver morphogenesis | 10/2334 | 33/29008 | 0.0001839752225 | 0.001119156952 | 0.0006298709205 |
| **1404** | KO | GO:1904646 | cellular response to amyloid-beta | 10/2334 | 33/29008 | 0.0001839752225 | 0.001119156952 | 0.0006298709205 |
| **1405** | KO | GO:0045839 | negative regulation of mitotic nuclear division | 13/2334 | 52/29008 | 0.0001840582243 | 0.001119156952 | 0.0006298709205 |
| **1406** | KO | GO:0061028 | establishment of endothelial barrier | 13/2334 | 52/29008 | 0.0001840582243 | 0.001119156952 | 0.0006298709205 |
| **1407** | KO | GO:1902041 | regulation of extrinsic apoptotic signaling pathway via death domain receptors | 13/2334 | 52/29008 | 0.0001840582243 | 0.001119156952 | 0.0006298709205 |
| **1408** | KO | GO:0098656 | anion transmembrane transport | 31/2334 | 194/29008 | 0.0001841581509 | 0.001119156952 | 0.0006298709205 |
| **1409** | KO | GO:0046883 | regulation of hormone secretion | 43/2334 | 302/29008 | 0.0001850286679 | 0.001123336102 | 0.0006322229812 |
| **1410** | KO | GO:0018107 | peptidyl-threonine phosphorylation | 22/2334 | 119/29008 | 0.0001903372713 | 0.001153898276 | 0.000649423629 |
| **1411** | KO | GO:0009416 | response to light stimulus | 45/2334 | 321/29008 | 0.0001904382897 | 0.001153898276 | 0.000649423629 |
| **1412** | KO | GO:0046902 | regulation of mitochondrial membrane permeability | 14/2334 | 59/29008 | 0.0001917132371 | 0.001157059065 | 0.0006512025476 |
| **1413** | KO | GO:0050775 | positive regulation of dendrite morphogenesis | 14/2334 | 59/29008 | 0.0001917132371 | 0.001157059065 | 0.0006512025476 |
| **1414** | KO | GO:0050922 | negative regulation of chemotaxis | 14/2334 | 59/29008 | 0.0001917132371 | 0.001157059065 | 0.0006512025476 |
| **1415** | KO | GO:1900024 | regulation of substrate adhesion-dependent cell spreading | 14/2334 | 59/29008 | 0.0001917132371 | 0.001157059065 | 0.0006512025476 |
| **1416** | KO | GO:0006936 | muscle contraction | 44/2334 | 312/29008 | 0.0001949406735 | 0.001175383217 | 0.0006615155336 |
| **1417** | KO | GO:0090276 | regulation of peptide hormone secretion | 35/2334 | 230/29008 | 0.0001973262593 | 0.001185756511 | 0.0006673537107 |
| **1418** | KO | GO:0021537 | telencephalon development | 38/2334 | 257/29008 | 0.0001973912688 | 0.001185756511 | 0.0006673537107 |
| **1419** | KO | GO:2000116 | regulation of cysteine-type endopeptidase activity | 36/2334 | 239/29008 | 0.0001981490284 | 0.001185756511 | 0.0006673537107 |
| **1420** | KO | GO:0000272 | polysaccharide catabolic process | 8/2334 | 22/29008 | 0.0001985910563 | 0.001185756511 | 0.0006673537107 |
| **1421** | KO | GO:0010743 | regulation of macrophage derived foam cell differentiation | 8/2334 | 22/29008 | 0.0001985910563 | 0.001185756511 | 0.0006673537107 |
| **1422** | KO | GO:0015865 | purine nucleotide transport | 8/2334 | 22/29008 | 0.0001985910563 | 0.001185756511 | 0.0006673537107 |
| **1423** | KO | GO:0020027 | hemoglobin metabolic process | 8/2334 | 22/29008 | 0.0001985910563 | 0.001185756511 | 0.0006673537107 |
| **1424** | KO | GO:0071800 | podosome assembly | 8/2334 | 22/29008 | 0.0001985910563 | 0.001185756511 | 0.0006673537107 |
| **1425** | KO | GO:0090026 | positive regulation of monocyte chemotaxis | 8/2334 | 22/29008 | 0.0001985910563 | 0.001185756511 | 0.0006673537107 |
| **1426** | KO | GO:0140632 | inflammasome complex assembly | 8/2334 | 22/29008 | 0.0001985910563 | 0.001185756511 | 0.0006673537107 |
| **1427** | KO | GO:0015931 | nucleobase-containing compound transport | 31/2334 | 195/29008 | 0.0002023787183 | 0.001207198879 | 0.0006794216553 |
| **1428** | KO | GO:0032957 | inositol trisphosphate metabolic process | 7/2334 | 17/29008 | 0.000204173781 | 0.001213195078 | 0.0006827963662 |
| **1429** | KO | GO:0035337 | fatty-acyl-CoA metabolic process | 7/2334 | 17/29008 | 0.000204173781 | 0.001213195078 | 0.0006827963662 |
| **1430** | KO | GO:0045342 | MHC class II biosynthetic process | 7/2334 | 17/29008 | 0.000204173781 | 0.001213195078 | 0.0006827963662 |
| **1431** | KO | GO:0048143 | astrocyte activation | 7/2334 | 17/29008 | 0.000204173781 | 0.001213195078 | 0.0006827963662 |
| **1432** | KO | GO:0002711 | positive regulation of T cell mediated immunity | 17/2334 | 81/29008 | 0.0002080174947 | 0.001232458522 | 0.0006936379943 |
| **1433** | KO | GO:0045471 | response to ethanol | 17/2334 | 81/29008 | 0.0002080174947 | 0.001232458522 | 0.0006936379943 |
| **1434** | KO | GO:1900542 | regulation of purine nucleotide metabolic process | 17/2334 | 81/29008 | 0.0002080174947 | 0.001232458522 | 0.0006936379943 |
| **1435** | KO | GO:0010821 | regulation of mitochondrion organization | 24/2334 | 136/29008 | 0.0002093448255 | 0.001236152377 | 0.0006957169269 |
| **1436** | KO | GO:0002369 | T cell cytokine production | 12/2334 | 46/29008 | 0.0002096469364 | 0.001236152377 | 0.0006957169269 |
| **1437** | KO | GO:0006040 | amino sugar metabolic process | 12/2334 | 46/29008 | 0.0002096469364 | 0.001236152377 | 0.0006957169269 |
| **1438** | KO | GO:0030890 | positive regulation of B cell proliferation | 12/2334 | 46/29008 | 0.0002096469364 | 0.001236152377 | 0.0006957169269 |
| **1439** | KO | GO:0031952 | regulation of protein autophosphorylation | 12/2334 | 46/29008 | 0.0002096469364 | 0.001236152377 | 0.0006957169269 |
| **1440** | KO | GO:2001020 | regulation of response to DNA damage stimulus | 34/2334 | 222/29008 | 0.0002134262594 | 0.001257230046 | 0.0007075796156 |
| **1441** | KO | GO:0044106 | cellular amine metabolic process | 22/2334 | 120/29008 | 0.0002156110768 | 0.001268883578 | 0.0007141383215 |
| **1442** | KO | GO:0048015 | phosphatidylinositol-mediated signaling | 28/2334 | 170/29008 | 0.000220416425 | 0.001295922024 | 0.0007293557857 |
| **1443** | KO | GO:0015914 | phospholipid transport | 18/2334 | 89/29008 | 0.000224141376 | 0.001315305267 | 0.0007402648372 |
| **1444** | KO | GO:0046364 | monosaccharide biosynthetic process | 18/2334 | 89/29008 | 0.000224141376 | 0.001315305267 | 0.0007402648372 |
| **1445** | KO | GO:0072331 | signal transduction by p53 class mediator | 25/2334 | 145/29008 | 0.0002274129859 | 0.00133323033 | 0.0007503532129 |
| **1446** | KO | GO:0009123 | nucleoside monophosphate metabolic process | 15/2334 | 67/29008 | 0.0002290949178 | 0.001340532548 | 0.0007544629624 |
| **1447** | KO | GO:0032663 | regulation of interleukin-2 production | 15/2334 | 67/29008 | 0.0002290949178 | 0.001340532548 | 0.0007544629624 |
| **1448** | KO | GO:0006195 | purine nucleotide catabolic process | 11/2334 | 40/29008 | 0.0002308956219 | 0.001344666067 | 0.0007567893419 |
| **1449** | KO | GO:0006730 | one-carbon metabolic process | 11/2334 | 40/29008 | 0.0002308956219 | 0.001344666067 | 0.0007567893419 |
| **1450** | KO | GO:0007094 | mitotic spindle assembly checkpoint signaling | 11/2334 | 40/29008 | 0.0002308956219 | 0.001344666067 | 0.0007567893419 |
| **1451** | KO | GO:0071173 | spindle assembly checkpoint signaling | 11/2334 | 40/29008 | 0.0002308956219 | 0.001344666067 | 0.0007567893419 |
| **1452** | KO | GO:2000516 | positive regulation of CD4-positive, alpha-beta T cell activation | 11/2334 | 40/29008 | 0.0002308956219 | 0.001344666067 | 0.0007567893419 |
| **1453** | KO | GO:0019748 | secondary metabolic process | 14/2334 | 60/29008 | 0.0002317320711 | 0.001348259323 | 0.0007588116562 |
| **1454** | KO | GO:0036293 | response to decreased oxygen levels | 36/2334 | 241/29008 | 0.0002339542374 | 0.001359900506 | 0.0007653634118 |
| **1455** | KO | GO:0010893 | positive regulation of steroid biosynthetic process | 9/2334 | 28/29008 | 0.0002344455591 | 0.001361468351 | 0.0007662458079 |
| **1456** | KO | GO:0046822 | regulation of nucleocytoplasmic transport | 23/2334 | 129/29008 | 0.000240364308 | 0.001394521537 | 0.0007848484187 |
| **1457** | KO | GO:0032205 | negative regulation of telomere maintenance | 10/2334 | 34/29008 | 0.0002419526368 | 0.001398454375 | 0.0007870618528 |
| **1458** | KO | GO:0036037 | CD8-positive, alpha-beta T cell activation | 10/2334 | 34/29008 | 0.0002419526368 | 0.001398454375 | 0.0007870618528 |
| **1459** | KO | GO:0042278 | purine nucleoside metabolic process | 10/2334 | 34/29008 | 0.0002419526368 | 0.001398454375 | 0.0007870618528 |
| **1460** | KO | GO:0070542 | response to fatty acid | 10/2334 | 34/29008 | 0.0002419526368 | 0.001398454375 | 0.0007870618528 |
| **1461** | KO | GO:0002260 | lymphocyte homeostasis | 17/2334 | 82/29008 | 0.000242992417 | 0.001401826676 | 0.0007889598122 |
| **1462** | KO | GO:0019319 | hexose biosynthetic process | 17/2334 | 82/29008 | 0.000242992417 | 0.001401826676 | 0.0007889598122 |
| **1463** | KO | GO:0006643 | membrane lipid metabolic process | 29/2334 | 180/29008 | 0.0002532629161 | 0.001459706714 | 0.0008215351827 |
| **1464** | KO | GO:1901988 | negative regulation of cell cycle phase transition | 36/2334 | 242/29008 | 0.0002539417883 | 0.001462247748 | 0.0008229652983 |
| **1465** | KO | GO:1901992 | positive regulation of mitotic cell cycle phase transition | 18/2334 | 90/29008 | 0.0002593800255 | 0.001492163742 | 0.0008398022706 |
| **1466** | KO | GO:1905897 | regulation of response to endoplasmic reticulum stress | 16/2334 | 75/29008 | 0.0002596391184 | 0.001492257009 | 0.0008398547622 |
| **1467** | KO | GO:0045910 | negative regulation of DNA recombination | 12/2334 | 47/29008 | 0.0002610916612 | 0.001499202959 | 0.0008437640011 |
| **1468** | KO | GO:0050890 | cognition | 48/2334 | 354/29008 | 0.0002630117192 | 0.00150881793 | 0.000849175387 |
| **1469** | KO | GO:0032872 | regulation of stress-activated MAPK cascade | 31/2334 | 198/29008 | 0.0002670737699 | 0.001530691458 | 0.000861485992 |
| **1470** | KO | GO:0062014 | negative regulation of small molecule metabolic process | 19/2334 | 98/29008 | 0.0002701234376 | 0.001543914907 | 0.0008689282603 |
| **1471** | KO | GO:0051592 | response to calcium ion | 23/2334 | 130/29008 | 0.0002701348511 | 0.001543914907 | 0.0008689282603 |
| **1472** | KO | GO:0061041 | regulation of wound healing | 23/2334 | 130/29008 | 0.0002701348511 | 0.001543914907 | 0.0008689282603 |
| **1473** | KO | GO:0032720 | negative regulation of tumor necrosis factor production | 15/2334 | 68/29008 | 0.0002723158478 | 0.00155348985 | 0.0008743171187 |
| **1474** | KO | GO:1905818 | regulation of chromosome separation | 15/2334 | 68/29008 | 0.0002723158478 | 0.00155348985 | 0.0008743171187 |
| **1475** | KO | GO:0044843 | cell cycle G1/S phase transition | 33/2334 | 216/29008 | 0.0002744190877 | 0.001564036062 | 0.0008802526149 |
| **1476** | KO | GO:0007091 | metaphase/anaphase transition of mitotic cell cycle | 14/2334 | 61/29008 | 0.0002787255728 | 0.001585638814 | 0.0008924108251 |
| **1477** | KO | GO:1902099 | regulation of metaphase/anaphase transition of cell cycle | 14/2334 | 61/29008 | 0.0002787255728 | 0.001585638814 | 0.0008924108251 |
| **1478** | KO | GO:0050678 | regulation of epithelial cell proliferation | 53/2334 | 403/29008 | 0.0002805552666 | 0.001594571284 | 0.0008974380941 |
| **1479** | KO | GO:0032725 | positive regulation of granulocyte macrophage colony-stimulating factor production | 6/2334 | 13/29008 | 0.0002816944489 | 0.001595143497 | 0.0008977601403 |
| **1480** | KO | GO:0042590 | antigen processing and presentation of exogenous peptide antigen via MHC class I | 6/2334 | 13/29008 | 0.0002816944489 | 0.001595143497 | 0.0008977601403 |
| **1481** | KO | GO:0046462 | monoacylglycerol metabolic process | 6/2334 | 13/29008 | 0.0002816944489 | 0.001595143497 | 0.0008977601403 |
| **1482** | KO | GO:0061620 | glycolytic process through glucose-6-phosphate | 6/2334 | 13/29008 | 0.0002816944489 | 0.001595143497 | 0.0008977601403 |
| **1483** | KO | GO:0002693 | positive regulation of cellular extravasation | 8/2334 | 23/29008 | 0.0002831033009 | 0.001598700993 | 0.0008997623292 |
| **1484** | KO | GO:0010232 | vascular transport | 8/2334 | 23/29008 | 0.0002831033009 | 0.001598700993 | 0.0008997623292 |
| **1485** | KO | GO:1901623 | regulation of lymphocyte chemotaxis | 8/2334 | 23/29008 | 0.0002831033009 | 0.001598700993 | 0.0008997623292 |
| **1486** | KO | GO:0006865 | amino acid transport | 27/2334 | 164/29008 | 0.0002847983816 | 0.001606796379 | 0.0009043184799 |
| **1487** | KO | GO:0050803 | regulation of synapse structure or activity | 39/2334 | 271/29008 | 0.0002885641818 | 0.001626548929 | 0.0009154353812 |
| **1488** | KO | GO:0006953 | acute-phase response | 11/2334 | 41/29008 | 0.0002927961518 | 0.001645873337 | 0.0009263113196 |
| **1489** | KO | GO:0043368 | positive T cell selection | 11/2334 | 41/29008 | 0.0002927961518 | 0.001645873337 | 0.0009263113196 |
| **1490** | KO | GO:1901568 | fatty acid derivative metabolic process | 11/2334 | 41/29008 | 0.0002927961518 | 0.001645873337 | 0.0009263113196 |
| **1491** | KO | GO:0016079 | synaptic vesicle exocytosis | 24/2334 | 139/29008 | 0.0002938170564 | 0.001650102372 | 0.0009286914561 |
| **1492** | KO | GO:0006836 | neurotransmitter transport | 36/2334 | 244/29008 | 0.0002985537485 | 0.001675172813 | 0.000942801311 |
| **1493** | KO | GO:1903578 | regulation of ATP metabolic process | 18/2334 | 91/29008 | 0.0002993454969 | 0.001678082786 | 0.0009444390679 |
| **1494** | KO | GO:0006766 | vitamin metabolic process | 16/2334 | 76/29008 | 0.000304614586 | 0.001706063825 | 0.0009601870316 |
| **1495** | KO | GO:0042742 | defense response to bacterium | 60/2334 | 473/29008 | 0.0003076327631 | 0.001721398631 | 0.0009688175888 |
| **1496** | KO | GO:0001676 | long-chain fatty acid metabolic process | 22/2334 | 123/29008 | 0.0003100284427 | 0.001733225434 | 0.0009754738129 |
| **1497** | KO | GO:0046184 | aldehyde biosynthetic process | 7/2334 | 18/29008 | 0.000310936605 | 0.001736722274 | 0.0009774418636 |
| **1498** | KO | GO:0042113 | B cell activation | 56/2334 | 434/29008 | 0.0003127361071 | 0.001745186778 | 0.0009822057574 |
| **1499** | KO | GO:0002068 | glandular epithelial cell development | 9/2334 | 29/29008 | 0.000315809107 | 0.001755955795 | 0.0009882666507 |
| **1500** | KO | GO:0002313 | mature B cell differentiation involved in immune response | 9/2334 | 29/29008 | 0.000315809107 | 0.001755955795 | 0.0009882666507 |
| **1501** | KO | GO:0019835 | cytolysis | 9/2334 | 29/29008 | 0.000315809107 | 0.001755955795 | 0.0009882666507 |
| **1502** | KO | GO:0090200 | positive regulation of release of cytochrome c from mitochondria | 9/2334 | 29/29008 | 0.000315809107 | 0.001755955795 | 0.0009882666507 |
| **1503** | KO | GO:0006949 | syncytium formation | 15/2334 | 69/29008 | 0.000322380949 | 0.001790875724 | 0.00100791988 |
| **1504** | KO | GO:0031330 | negative regulation of cellular catabolic process | 36/2334 | 245/29008 | 0.0003233800542 | 0.001794803119 | 0.001010130251 |
| **1505** | KO | GO:0034103 | regulation of tissue remodeling | 17/2334 | 84/29008 | 0.0003285175894 | 0.001820028917 | 0.001024327541 |
| **1506** | KO | GO:1904589 | regulation of protein import | 17/2334 | 84/29008 | 0.0003285175894 | 0.001820028917 | 0.001024327541 |
| **1507** | KO | GO:0032964 | collagen biosynthetic process | 13/2334 | 55/29008 | 0.000335133817 | 0.001851674615 | 0.001042138005 |
| **1508** | KO | GO:0140353 | lipid export from cell | 13/2334 | 55/29008 | 0.000335133817 | 0.001851674615 | 0.001042138005 |
| **1509** | KO | GO:1901607 | alpha-amino acid biosynthetic process | 13/2334 | 55/29008 | 0.000335133817 | 0.001851674615 | 0.001042138005 |
| **1510** | KO | GO:0072659 | protein localization to plasma membrane | 42/2334 | 301/29008 | 0.0003374036743 | 0.001862541038 | 0.001048253719 |
| **1511** | KO | GO:0030148 | sphingolipid biosynthetic process | 18/2334 | 92/29008 | 0.0003445560002 | 0.001900316037 | 0.001069513806 |
| **1512** | KO | GO:0070302 | regulation of stress-activated protein kinase signaling cascade | 31/2334 | 201/29008 | 0.0003495513253 | 0.001925131561 | 0.001083480191 |
| **1513** | KO | GO:0071326 | cellular response to monosaccharide stimulus | 25/2334 | 149/29008 | 0.0003496821 | 0.001925131561 | 0.001083480191 |
| **1514** | KO | GO:0043281 | regulation of cysteine-type endopeptidase activity involved in apoptotic process | 32/2334 | 210/29008 | 0.0003528886382 | 0.001941045473 | 0.001092436674 |
| **1515** | KO | GO:0016241 | regulation of macroautophagy | 19/2334 | 100/29008 | 0.0003535948752 | 0.001943190441 | 0.00109364388 |
| **1516** | KO | GO:0009141 | nucleoside triphosphate metabolic process | 21/2334 | 116/29008 | 0.0003546846143 | 0.001947437239 | 0.001096034013 |
| **1517** | KO | GO:0043255 | regulation of carbohydrate biosynthetic process | 20/2334 | 108/29008 | 0.0003566336963 | 0.001956390563 | 0.001101073019 |
| **1518** | KO | GO:0031023 | microtubule organizing center organization | 24/2334 | 141/29008 | 0.0003656062307 | 0.002003822196 | 0.00112776794 |
| **1519** | KO | GO:0045927 | positive regulation of growth | 44/2334 | 321/29008 | 0.0003659953829 | 0.00200416723 | 0.001127962128 |
| **1520** | KO | GO:0071174 | mitotic spindle checkpoint signaling | 11/2334 | 42/29008 | 0.0003680991791 | 0.00201389257 | 0.001133435631 |
| **1521** | KO | GO:0016485 | protein processing | 37/2334 | 256/29008 | 0.0003738108897 | 0.002043322159 | 0.001149998851 |
| **1522** | KO | GO:0043525 | positive regulation of neuron apoptotic process | 17/2334 | 85/29008 | 0.0003802802906 | 0.00207651123 | 0.001168677938 |
| **1523** | KO | GO:0016050 | vesicle organization | 40/2334 | 284/29008 | 0.0003805585359 | 0.00207651123 | 0.001168677938 |
| **1524** | KO | GO:0043433 | negative regulation of DNA-binding transcription factor activity | 27/2334 | 167/29008 | 0.0003834806824 | 0.002090599213 | 0.001176606773 |
| **1525** | KO | GO:0031345 | negative regulation of cell projection organization | 32/2334 | 211/29008 | 0.0003844707973 | 0.002092836485 | 0.001177865929 |
| **1526** | KO | GO:0017038 | protein import | 34/2334 | 229/29008 | 0.0003845723294 | 0.002092836485 | 0.001177865929 |
| **1527** | KO | GO:1901888 | regulation of cell junction assembly | 33/2334 | 220/29008 | 0.0003853714654 | 0.002095329454 | 0.001179268993 |
| **1528** | KO | GO:0048814 | regulation of dendrite morphogenesis | 18/2334 | 93/29008 | 0.0003955723434 | 0.0021449388 | 0.001207189549 |
| **1529** | KO | GO:2001242 | regulation of intrinsic apoptotic signaling pathway | 28/2334 | 176/29008 | 0.000395780252 | 0.0021449388 | 0.001207189549 |
| **1530** | KO | GO:0007157 | heterophilic cell-cell adhesion via plasma membrane cell adhesion molecules | 12/2334 | 49/29008 | 0.000396590247 | 0.0021449388 | 0.001207189549 |
| **1531** | KO | GO:0009262 | deoxyribonucleotide metabolic process | 12/2334 | 49/29008 | 0.000396590247 | 0.0021449388 | 0.001207189549 |
| **1532** | KO | GO:0032309 | icosanoid secretion | 12/2334 | 49/29008 | 0.000396590247 | 0.0021449388 | 0.001207189549 |
| **1533** | KO | GO:0032814 | regulation of natural killer cell activation | 12/2334 | 49/29008 | 0.000396590247 | 0.0021449388 | 0.001207189549 |
| **1534** | KO | GO:0043401 | steroid hormone mediated signaling pathway | 21/2334 | 117/29008 | 0.0004000068841 | 0.002161514772 | 0.001216518645 |
| **1535** | KO | GO:0045862 | positive regulation of proteolysis | 47/2334 | 351/29008 | 0.0004024159105 | 0.002172621577 | 0.001222769648 |
| **1536** | KO | GO:0009154 | purine ribonucleotide catabolic process | 10/2334 | 36/29008 | 0.0004042758434 | 0.002175018198 | 0.001224118486 |
| **1537** | KO | GO:0050869 | negative regulation of B cell activation | 10/2334 | 36/29008 | 0.0004042758434 | 0.002175018198 | 0.001224118486 |
| **1538** | KO | GO:0050901 | leukocyte tethering or rolling | 10/2334 | 36/29008 | 0.0004042758434 | 0.002175018198 | 0.001224118486 |
| **1539** | KO | GO:0097242 | amyloid-beta clearance | 10/2334 | 36/29008 | 0.0004042758434 | 0.002175018198 | 0.001224118486 |
| **1540** | KO | GO:1901861 | regulation of muscle tissue development | 13/2334 | 56/29008 | 0.0004046560242 | 0.002175158891 | 0.00122419767 |
| **1541** | KO | GO:0000209 | protein polyubiquitination | 36/2334 | 248/29008 | 0.0004092647095 | 0.002196089411 | 0.001235977542 |
| **1542** | KO | GO:0031098 | stress-activated protein kinase signaling cascade | 36/2334 | 248/29008 | 0.0004092647095 | 0.002196089411 | 0.001235977542 |
| **1543** | KO | GO:0005977 | glycogen metabolic process | 16/2334 | 78/29008 | 0.0004150472857 | 0.002221298365 | 0.001250165352 |
| **1544** | KO | GO:0006073 | cellular glucan metabolic process | 16/2334 | 78/29008 | 0.0004150472857 | 0.002221298365 | 0.001250165352 |
| **1545** | KO | GO:0044042 | glucan metabolic process | 16/2334 | 78/29008 | 0.0004150472857 | 0.002221298365 | 0.001250165352 |
| **1546** | KO | GO:0043542 | endothelial cell migration | 33/2334 | 221/29008 | 0.0004186834108 | 0.002235486173 | 0.00125815037 |
| **1547** | KO | GO:0048639 | positive regulation of developmental growth | 33/2334 | 221/29008 | 0.0004186834108 | 0.002235486173 | 0.00125815037 |
| **1548** | KO | GO:0043153 | entrainment of circadian clock by photoperiod | 9/2334 | 30/29008 | 0.0004191536574 | 0.002235486173 | 0.00125815037 |
| **1549** | KO | GO:0046885 | regulation of hormone biosynthetic process | 9/2334 | 30/29008 | 0.0004191536574 | 0.002235486173 | 0.00125815037 |
| **1550** | KO | GO:0016055 | Wnt signaling pathway | 57/2334 | 449/29008 | 0.0004201129176 | 0.002238658947 | 0.001259936033 |
| **1551** | KO | GO:0002793 | positive regulation of peptide secretion | 22/2334 | 126/29008 | 0.0004388968634 | 0.002336726455 | 0.001315129249 |
| **1552** | KO | GO:0001913 | T cell mediated cytotoxicity | 15/2334 | 71/29008 | 0.0004465890544 | 0.002375621775 | 0.001337019862 |
| **1553** | KO | GO:0000212 | meiotic spindle organization | 7/2334 | 19/29008 | 0.0004582262141 | 0.002418753135 | 0.001361294553 |
| **1554** | KO | GO:0034501 | protein localization to kinetochore | 7/2334 | 19/29008 | 0.0004582262141 | 0.002418753135 | 0.001361294553 |
| **1555** | KO | GO:0044247 | cellular polysaccharide catabolic process | 7/2334 | 19/29008 | 0.0004582262141 | 0.002418753135 | 0.001361294553 |
| **1556** | KO | GO:0046485 | ether lipid metabolic process | 7/2334 | 19/29008 | 0.0004582262141 | 0.002418753135 | 0.001361294553 |
| **1557** | KO | GO:1903083 | protein localization to condensed chromosome | 7/2334 | 19/29008 | 0.0004582262141 | 0.002418753135 | 0.001361294553 |
| **1558** | KO | GO:0046636 | negative regulation of alpha-beta T cell activation | 11/2334 | 43/29008 | 0.0004590080672 | 0.002418753135 | 0.001361294553 |
| **1559** | KO | GO:1900026 | positive regulation of substrate adhesion-dependent cell spreading | 11/2334 | 43/29008 | 0.0004590080672 | 0.002418753135 | 0.001361294553 |
| **1560** | KO | GO:0001660 | fever generation | 6/2334 | 14/29008 | 0.0004594213718 | 0.002418753135 | 0.001361294553 |
| **1561** | KO | GO:0002638 | negative regulation of immunoglobulin production | 6/2334 | 14/29008 | 0.0004594213718 | 0.002418753135 | 0.001361294553 |
| **1562** | KO | GO:0006002 | fructose 6-phosphate metabolic process | 6/2334 | 14/29008 | 0.0004594213718 | 0.002418753135 | 0.001361294553 |
| **1563** | KO | GO:0010755 | regulation of plasminogen activation | 6/2334 | 14/29008 | 0.0004594213718 | 0.002418753135 | 0.001361294553 |
| **1564** | KO | GO:0070207 | protein homotrimerization | 6/2334 | 14/29008 | 0.0004594213718 | 0.002418753135 | 0.001361294553 |
| **1565** | KO | GO:0198738 | cell-cell signaling by wnt | 57/2334 | 451/29008 | 0.0004694187452 | 0.002469271207 | 0.001389726548 |
| **1566** | KO | GO:0044784 | metaphase/anaphase transition of cell cycle | 14/2334 | 64/29008 | 0.0004717024308 | 0.002478793437 | 0.001395085739 |
| **1567** | KO | GO:0014706 | striated muscle tissue development | 58/2334 | 461/29008 | 0.0004720358595 | 0.002478793437 | 0.001395085739 |
| **1568** | KO | GO:0050673 | epithelial cell proliferation | 59/2334 | 471/29008 | 0.0004740199186 | 0.002487086575 | 0.001399753187 |
| **1569** | KO | GO:0021700 | developmental maturation | 46/2334 | 344/29008 | 0.0004747094499 | 0.002488579232 | 0.001400593266 |
| **1570** | KO | GO:0006094 | gluconeogenesis | 16/2334 | 79/29008 | 0.0004821134857 | 0.002525238922 | 0.001421225648 |
| **1571** | KO | GO:0045646 | regulation of erythrocyte differentiation | 12/2334 | 50/29008 | 0.0004840276749 | 0.002530949817 | 0.001424439787 |
| **1572** | KO | GO:0097237 | cellular response to toxic substance | 12/2334 | 50/29008 | 0.0004840276749 | 0.002530949817 | 0.001424439787 |
| **1573** | KO | GO:0018210 | peptidyl-threonine modification | 22/2334 | 127/29008 | 0.0004911804468 | 0.002566167232 | 0.001444260444 |
| **1574** | KO | GO:0043491 | protein kinase B signaling | 31/2334 | 205/29008 | 0.0004942832639 | 0.002576123126 | 0.001449863704 |
| **1575** | KO | GO:2000241 | regulation of reproductive process | 31/2334 | 205/29008 | 0.0004942832639 | 0.002576123126 | 0.001449863704 |
| **1576** | KO | GO:0048469 | cell maturation | 32/2334 | 214/29008 | 0.0004947632305 | 0.002576123126 | 0.001449863704 |
| **1577** | KO | GO:1903050 | regulation of proteolysis involved in cellular protein catabolic process | 32/2334 | 214/29008 | 0.0004947632305 | 0.002576123126 | 0.001449863704 |
| **1578** | KO | GO:0097553 | calcium ion transmembrane import into cytosol | 24/2334 | 144/29008 | 0.0005021231365 | 0.00261223078 | 0.001470185395 |
| **1579** | KO | GO:0016572 | histone phosphorylation | 10/2334 | 37/29008 | 0.000514402774 | 0.002667080712 | 0.001501055396 |
| **1580** | KO | GO:0019076 | viral release from host cell | 10/2334 | 37/29008 | 0.000514402774 | 0.002667080712 | 0.001501055396 |
| **1581** | KO | GO:0035890 | exit from host | 10/2334 | 37/29008 | 0.000514402774 | 0.002667080712 | 0.001501055396 |
| **1582** | KO | GO:0035891 | exit from host cell | 10/2334 | 37/29008 | 0.000514402774 | 0.002667080712 | 0.001501055396 |
| **1583** | KO | GO:0010522 | regulation of calcium ion transport into cytosol | 20/2334 | 111/29008 | 0.0005162673971 | 0.002674491474 | 0.001505226235 |
| **1584** | KO | GO:0033077 | T cell differentiation in thymus | 18/2334 | 95/29008 | 0.0005174909035 | 0.002678571281 | 0.001507522386 |
| **1585** | KO | GO:0051170 | import into nucleus | 28/2334 | 179/29008 | 0.0005227781874 | 0.002703660929 | 0.001521643049 |
| **1586** | KO | GO:0060562 | epithelial tube morphogenesis | 51/2334 | 394/29008 | 0.0005243917011 | 0.002709724652 | 0.001525055764 |
| **1587** | KO | GO:0014065 | phosphatidylinositol 3-kinase signaling | 23/2334 | 136/29008 | 0.0005266279913 | 0.002718993595 | 0.001530272403 |
| **1588** | KO | GO:0045333 | cellular respiration | 32/2334 | 215/29008 | 0.0005372876896 | 0.002771700726 | 0.001559936418 |
| **1589** | KO | GO:0002726 | positive regulation of T cell cytokine production | 8/2334 | 25/29008 | 0.0005399279348 | 0.002773676615 | 0.001561048465 |
| **1590** | KO | GO:0034138 | toll-like receptor 3 signaling pathway | 8/2334 | 25/29008 | 0.0005399279348 | 0.002773676615 | 0.001561048465 |
| **1591** | KO | GO:0050765 | negative regulation of phagocytosis | 8/2334 | 25/29008 | 0.0005399279348 | 0.002773676615 | 0.001561048465 |
| **1592** | KO | GO:0050860 | negative regulation of T cell receptor signaling pathway | 8/2334 | 25/29008 | 0.0005399279348 | 0.002773676615 | 0.001561048465 |
| **1593** | KO | GO:1903306 | negative regulation of regulated secretory pathway | 8/2334 | 25/29008 | 0.0005399279348 | 0.002773676615 | 0.001561048465 |
| **1594** | KO | GO:0010977 | negative regulation of neuron projection development | 26/2334 | 162/29008 | 0.0005460557048 | 0.002802812239 | 0.001577446239 |
| **1595** | KO | GO:0009648 | photoperiodism | 9/2334 | 31/29008 | 0.0005487712183 | 0.002809894245 | 0.001581432051 |
| **1596** | KO | GO:1902186 | regulation of viral release from host cell | 9/2334 | 31/29008 | 0.0005487712183 | 0.002809894245 | 0.001581432051 |
| **1597** | KO | GO:2000045 | regulation of G1/S transition of mitotic cell cycle | 22/2334 | 128/29008 | 0.0005488074697 | 0.002809894245 | 0.001581432051 |
| **1598** | KO | GO:0051262 | protein tetramerization | 16/2334 | 80/29008 | 0.0005582650082 | 0.002853560907 | 0.001606008015 |
| **1599** | KO | GO:0070228 | regulation of lymphocyte apoptotic process | 16/2334 | 80/29008 | 0.0005582650082 | 0.002853560907 | 0.001606008015 |
| **1600** | KO | GO:1904019 | epithelial cell apoptotic process | 21/2334 | 120/29008 | 0.0005676013584 | 0.002893512319 | 0.001628493004 |
| **1601** | KO | GO:0009394 | 2'-deoxyribonucleotide metabolic process | 11/2334 | 44/29008 | 0.0005679648203 | 0.002893512319 | 0.001628493004 |
| **1602** | KO | GO:0019692 | deoxyribose phosphate metabolic process | 11/2334 | 44/29008 | 0.0005679648203 | 0.002893512319 | 0.001628493004 |
| **1603** | KO | GO:0097028 | dendritic cell differentiation | 11/2334 | 44/29008 | 0.0005679648203 | 0.002893512319 | 0.001628493004 |
| **1604** | KO | GO:0010761 | fibroblast migration | 13/2334 | 58/29008 | 0.0005808736688 | 0.002951933682 | 0.001661373037 |
| **1605** | KO | GO:0014009 | glial cell proliferation | 13/2334 | 58/29008 | 0.0005808736688 | 0.002951933682 | 0.001661373037 |
| **1606** | KO | GO:0051496 | positive regulation of stress fiber assembly | 13/2334 | 58/29008 | 0.0005808736688 | 0.002951933682 | 0.001661373037 |
| **1607** | KO | GO:0000018 | regulation of DNA recombination | 20/2334 | 112/29008 | 0.0005817329053 | 0.002953857 | 0.001662455497 |
| **1608** | KO | GO:0035265 | organ growth | 32/2334 | 216/29008 | 0.0005830039682 | 0.002957866541 | 0.0016647121 |
| **1609** | KO | GO:0002761 | regulation of myeloid leukocyte differentiation | 23/2334 | 137/29008 | 0.0005855174507 | 0.00296588009 | 0.001669222193 |
| **1610** | KO | GO:0007568 | aging | 37/2334 | 262/29008 | 0.0005855489175 | 0.00296588009 | 0.001669222193 |
| **1611** | KO | GO:0050832 | defense response to fungus | 12/2334 | 51/29008 | 0.0005871382511 | 0.002969034909 | 0.001670997752 |
| **1612** | KO | GO:0051289 | protein homotetramerization | 12/2334 | 51/29008 | 0.0005871382511 | 0.002969034909 | 0.001670997752 |
| **1613** | KO | GO:0001657 | ureteric bud development | 19/2334 | 104/29008 | 0.000589693873 | 0.002969998474 | 0.001671540054 |
| **1614** | KO | GO:0070227 | lymphocyte apoptotic process | 19/2334 | 104/29008 | 0.000589693873 | 0.002969998474 | 0.001671540054 |
| **1615** | KO | GO:0072163 | mesonephric epithelium development | 19/2334 | 104/29008 | 0.000589693873 | 0.002969998474 | 0.001671540054 |
| **1616** | KO | GO:0072164 | mesonephric tubule development | 19/2334 | 104/29008 | 0.000589693873 | 0.002969998474 | 0.001671540054 |
| **1617** | KO | GO:0009408 | response to heat | 18/2334 | 96/29008 | 0.0005897457908 | 0.002969998474 | 0.001671540054 |
| **1618** | KO | GO:0006735 | NADH regeneration | 5/2334 | 10/29008 | 0.0005995701331 | 0.003004697306 | 0.001691068848 |
| **1619** | KO | GO:0046130 | purine ribonucleoside catabolic process | 5/2334 | 10/29008 | 0.0005995701331 | 0.003004697306 | 0.001691068848 |
| **1620** | KO | GO:0061621 | canonical glycolysis | 5/2334 | 10/29008 | 0.0005995701331 | 0.003004697306 | 0.001691068848 |
| **1621** | KO | GO:0061718 | glucose catabolic process to pyruvate | 5/2334 | 10/29008 | 0.0005995701331 | 0.003004697306 | 0.001691068848 |
| **1622** | KO | GO:0070673 | response to interleukin-18 | 5/2334 | 10/29008 | 0.0005995701331 | 0.003004697306 | 0.001691068848 |
| **1623** | KO | GO:0090306 | meiotic spindle assembly | 5/2334 | 10/29008 | 0.0005995701331 | 0.003004697306 | 0.001691068848 |
| **1624** | KO | GO:0043113 | receptor clustering | 15/2334 | 73/29008 | 0.0006096245958 | 0.003052594553 | 0.00171802582 |
| **1625** | KO | GO:0051017 | actin filament bundle assembly | 27/2334 | 172/29008 | 0.0006156951678 | 0.003080440668 | 0.001733697847 |
| **1626** | KO | GO:0044839 | cell cycle G2/M phase transition | 24/2334 | 146/29008 | 0.0006161884083 | 0.003080440668 | 0.001733697847 |
| **1627** | KO | GO:0120032 | regulation of plasma membrane bounded cell projection assembly | 30/2334 | 199/29008 | 0.0006355443199 | 0.003174621383 | 0.001786703544 |
| **1628** | KO | GO:0051146 | striated muscle cell differentiation | 43/2334 | 320/29008 | 0.0006432606751 | 0.003210555311 | 0.00180692746 |
| **1629** | KO | GO:0042267 | natural killer cell mediated cytotoxicity | 16/2334 | 81/29008 | 0.0006444797808 | 0.003211422363 | 0.001807415444 |
| **1630** | KO | GO:0045913 | positive regulation of carbohydrate metabolic process | 16/2334 | 81/29008 | 0.0006444797808 | 0.003211422363 | 0.001807415444 |
| **1631** | KO | GO:0006739 | NADP metabolic process | 10/2334 | 38/29008 | 0.0006482539107 | 0.003224997593 | 0.001815055697 |
| **1632** | KO | GO:0009595 | detection of biotic stimulus | 10/2334 | 38/29008 | 0.0006482539107 | 0.003224997593 | 0.001815055697 |
| **1633** | KO | GO:0060350 | endochondral bone morphogenesis | 14/2334 | 66/29008 | 0.0006555965746 | 0.003238384318 | 0.001822589858 |
| **1634** | KO | GO:0006390 | mitochondrial transcription | 7/2334 | 20/29008 | 0.0006562155723 | 0.003238384318 | 0.001822589858 |
| **1635** | KO | GO:0033033 | negative regulation of myeloid cell apoptotic process | 7/2334 | 20/29008 | 0.0006562155723 | 0.003238384318 | 0.001822589858 |
| **1636** | KO | GO:0044321 | response to leptin | 7/2334 | 20/29008 | 0.0006562155723 | 0.003238384318 | 0.001822589858 |
| **1637** | KO | GO:0044546 | NLRP3 inflammasome complex assembly | 7/2334 | 20/29008 | 0.0006562155723 | 0.003238384318 | 0.001822589858 |
| **1638** | KO | GO:0050665 | hydrogen peroxide biosynthetic process | 7/2334 | 20/29008 | 0.0006562155723 | 0.003238384318 | 0.001822589858 |
| **1639** | KO | GO:0060099 | regulation of phagocytosis, engulfment | 7/2334 | 20/29008 | 0.0006562155723 | 0.003238384318 | 0.001822589858 |
| **1640** | KO | GO:0098856 | intestinal lipid absorption | 7/2334 | 20/29008 | 0.0006562155723 | 0.003238384318 | 0.001822589858 |
| **1641** | KO | GO:1901028 | regulation of mitochondrial outer membrane permeabilization involved in apoptotic signaling pathway | 7/2334 | 20/29008 | 0.0006562155723 | 0.003238384318 | 0.001822589858 |
| **1642** | KO | GO:1902187 | negative regulation of viral release from host cell | 7/2334 | 20/29008 | 0.0006562155723 | 0.003238384318 | 0.001822589858 |
| **1643** | KO | GO:0032412 | regulation of ion transmembrane transporter activity | 38/2334 | 273/29008 | 0.0006594562597 | 0.003251765056 | 0.00183012065 |
| **1644** | KO | GO:0032024 | positive regulation of insulin secretion | 17/2334 | 89/29008 | 0.0006639629141 | 0.003271361783 | 0.001841149852 |
| **1645** | KO | GO:0046849 | bone remodeling | 19/2334 | 105/29008 | 0.0006665449312 | 0.003281451969 | 0.001846828693 |
| **1646** | KO | GO:0006672 | ceramide metabolic process | 18/2334 | 97/29008 | 0.0006705156933 | 0.003298357422 | 0.001856343224 |
| **1647** | KO | GO:0002639 | positive regulation of immunoglobulin production | 13/2334 | 59/29008 | 0.0006908537908 | 0.00338952483 | 0.001907653006 |
| **1648** | KO | GO:0043388 | positive regulation of DNA binding | 13/2334 | 59/29008 | 0.0006908537908 | 0.00338952483 | 0.001907653006 |
| **1649** | KO | GO:0050873 | brown fat cell differentiation | 13/2334 | 59/29008 | 0.0006908537908 | 0.00338952483 | 0.001907653006 |
| **1650** | KO | GO:0046578 | regulation of Ras protein signal transduction | 30/2334 | 200/29008 | 0.0006912556335 | 0.00338952483 | 0.001907653006 |
| **1651** | KO | GO:0031577 | spindle checkpoint signaling | 11/2334 | 45/29008 | 0.0006976588237 | 0.003418194428 | 0.001923788496 |
| **1652** | KO | GO:1903362 | regulation of cellular protein catabolic process | 35/2334 | 246/29008 | 0.00070768623 | 0.003456697988 | 0.001945458623 |
| **1653** | KO | GO:0032715 | negative regulation of interleukin-6 production | 12/2334 | 52/29008 | 0.0007080622591 | 0.003456697988 | 0.001945458623 |
| **1654** | KO | GO:0042307 | positive regulation of protein import into nucleus | 12/2334 | 52/29008 | 0.0007080622591 | 0.003456697988 | 0.001945458623 |
| **1655** | KO | GO:0045124 | regulation of bone resorption | 12/2334 | 52/29008 | 0.0007080622591 | 0.003456697988 | 0.001945458623 |
| **1656** | KO | GO:0002437 | inflammatory response to antigenic stimulus | 15/2334 | 74/29008 | 0.0007085482018 | 0.003456697988 | 0.001945458623 |
| **1657** | KO | GO:0009649 | entrainment of circadian clock | 9/2334 | 32/29008 | 0.0007094557557 | 0.003456697988 | 0.001945458623 |
| **1658** | KO | GO:0048535 | lymph node development | 9/2334 | 32/29008 | 0.0007094557557 | 0.003456697988 | 0.001945458623 |
| **1659** | KO | GO:0006244 | pyrimidine nucleotide catabolic process | 6/2334 | 15/29008 | 0.0007136874874 | 0.003466320887 | 0.001950874471 |
| **1660** | KO | GO:0010832 | negative regulation of myotube differentiation | 6/2334 | 15/29008 | 0.0007136874874 | 0.003466320887 | 0.001950874471 |
| **1661** | KO | GO:0032645 | regulation of granulocyte macrophage colony-stimulating factor production | 6/2334 | 15/29008 | 0.0007136874874 | 0.003466320887 | 0.001950874471 |
| **1662** | KO | GO:0046835 | carbohydrate phosphorylation | 6/2334 | 15/29008 | 0.0007136874874 | 0.003466320887 | 0.001950874471 |
| **1663** | KO | GO:0010811 | positive regulation of cell-substrate adhesion | 23/2334 | 139/29008 | 0.0007206908906 | 0.003497570957 | 0.001968462273 |
| **1664** | KO | GO:0002504 | antigen processing and presentation of peptide or polysaccharide antigen via MHC class II | 8/2334 | 26/29008 | 0.0007252661778 | 0.003505928715 | 0.001973166089 |
| **1665** | KO | GO:0002523 | leukocyte migration involved in inflammatory response | 8/2334 | 26/29008 | 0.0007252661778 | 0.003505928715 | 0.001973166089 |
| **1666** | KO | GO:0019320 | hexose catabolic process | 8/2334 | 26/29008 | 0.0007252661778 | 0.003505928715 | 0.001973166089 |
| **1667** | KO | GO:0031639 | plasminogen activation | 8/2334 | 26/29008 | 0.0007252661778 | 0.003505928715 | 0.001973166089 |
| **1668** | KO | GO:0050857 | positive regulation of antigen receptor-mediated signaling pathway | 8/2334 | 26/29008 | 0.0007252661778 | 0.003505928715 | 0.001973166089 |
| **1669** | KO | GO:0098657 | import into cell | 33/2334 | 228/29008 | 0.0007322807648 | 0.00353705426 | 0.001990683807 |
| **1670** | KO | GO:0032231 | regulation of actin filament bundle assembly | 20/2334 | 114/29008 | 0.0007344636757 | 0.00354481133 | 0.001995049551 |
| **1671** | KO | GO:0051303 | establishment of chromosome localization | 16/2334 | 82/29008 | 0.0007418100508 | 0.003577457576 | 0.002013423132 |
| **1672** | KO | GO:0045598 | regulation of fat cell differentiation | 24/2334 | 148/29008 | 0.0007521982103 | 0.003621869752 | 0.002038418677 |
| **1673** | KO | GO:0071331 | cellular response to hexose stimulus | 24/2334 | 148/29008 | 0.0007521982103 | 0.003621869752 | 0.002038418677 |
| **1674** | KO | GO:0044272 | sulfur compound biosynthetic process | 17/2334 | 90/29008 | 0.000758180847 | 0.003647817638 | 0.002053022365 |
| **1675** | KO | GO:0000768 | syncytium formation by plasma membrane fusion | 14/2334 | 67/29008 | 0.0007682573896 | 0.003690518688 | 0.002077054874 |
| **1676** | KO | GO:0140253 | cell-cell fusion | 14/2334 | 67/29008 | 0.0007682573896 | 0.003690518688 | 0.002077054874 |
| **1677** | KO | GO:0051403 | stress-activated MAPK cascade | 34/2334 | 238/29008 | 0.0007773852296 | 0.003731449102 | 0.002100090855 |
| **1678** | KO | GO:0097305 | response to alcohol | 33/2334 | 229/29008 | 0.0007908235115 | 0.003792989582 | 0.002134726353 |
| **1679** | KO | GO:0034614 | cellular response to reactive oxygen species | 23/2334 | 140/29008 | 0.0007978887282 | 0.003823891066 | 0.002152117967 |
| **1680** | KO | GO:0006606 | protein import into nucleus | 27/2334 | 175/29008 | 0.0008075751496 | 0.003864284828 | 0.002174851915 |
| **1681** | KO | GO:0061572 | actin filament bundle organization | 27/2334 | 175/29008 | 0.0008075751496 | 0.003864284828 | 0.002174851915 |
| **1682** | KO | GO:0001782 | B cell homeostasis | 10/2334 | 39/29008 | 0.0008095750166 | 0.003864824322 | 0.002175155547 |
| **1683** | KO | GO:0019432 | triglyceride biosynthetic process | 10/2334 | 39/29008 | 0.0008095750166 | 0.003864824322 | 0.002175155547 |
| **1684** | KO | GO:0042745 | circadian sleep/wake cycle | 10/2334 | 39/29008 | 0.0008095750166 | 0.003864824322 | 0.002175155547 |
| **1685** | KO | GO:0060491 | regulation of cell projection assembly | 30/2334 | 202/29008 | 0.0008156563238 | 0.00389083265 | 0.002189793253 |
| **1686** | KO | GO:0019751 | polyol metabolic process | 20/2334 | 115/29008 | 0.0008230007779 | 0.003922821396 | 0.002207796787 |
| **1687** | KO | GO:0001823 | mesonephros development | 19/2334 | 107/29008 | 0.0008464097916 | 0.004031272682 | 0.00226883408 |
| **1688** | KO | GO:0014014 | negative regulation of gliogenesis | 12/2334 | 53/29008 | 0.0008491306939 | 0.004031730281 | 0.002269091621 |
| **1689** | KO | GO:0016202 | regulation of striated muscle tissue development | 12/2334 | 53/29008 | 0.0008491306939 | 0.004031730281 | 0.002269091621 |
| **1690** | KO | GO:0043370 | regulation of CD4-positive, alpha-beta T cell differentiation | 12/2334 | 53/29008 | 0.0008491306939 | 0.004031730281 | 0.002269091621 |
| **1691** | KO | GO:2000351 | regulation of endothelial cell apoptotic process | 12/2334 | 53/29008 | 0.0008491306939 | 0.004031730281 | 0.002269091621 |
| **1692** | KO | GO:0032965 | regulation of collagen biosynthetic process | 11/2334 | 46/29008 | 0.0008510339883 | 0.004037646968 | 0.002272421582 |
| **1693** | KO | GO:0044264 | cellular polysaccharide metabolic process | 18/2334 | 99/29008 | 0.0008608679024 | 0.004081151537 | 0.002296906318 |
| **1694** | KO | GO:0070588 | calcium ion transmembrane transport | 40/2334 | 296/29008 | 0.00087108924 | 0.00412642428 | 0.002322386198 |
| **1695** | KO | GO:0051588 | regulation of neurotransmitter transport | 23/2334 | 141/29008 | 0.0008821471811 | 0.004175587273 | 0.002350055543 |
| **1696** | KO | GO:0008608 | attachment of spindle microtubules to kinetochore | 9/2334 | 33/29008 | 0.0009065109556 | 0.004271168183 | 0.002403849281 |
| **1697** | KO | GO:0044319 | wound healing, spreading of cells | 9/2334 | 33/29008 | 0.0009065109556 | 0.004271168183 | 0.002403849281 |
| **1698** | KO | GO:0055094 | response to lipoprotein particle | 9/2334 | 33/29008 | 0.0009065109556 | 0.004271168183 | 0.002403849281 |
| **1699** | KO | GO:0060706 | cell differentiation involved in embryonic placenta development | 9/2334 | 33/29008 | 0.0009065109556 | 0.004271168183 | 0.002403849281 |
| **1700** | KO | GO:0070266 | necroptotic process | 9/2334 | 33/29008 | 0.0009065109556 | 0.004271168183 | 0.002403849281 |
| **1701** | KO | GO:0090505 | epiboly involved in wound healing | 9/2334 | 33/29008 | 0.0009065109556 | 0.004271168183 | 0.002403849281 |
| **1702** | KO | GO:0007034 | vacuolar transport | 24/2334 | 150/29008 | 0.0009135563485 | 0.00429121166 | 0.002415129918 |
| **1703** | KO | GO:0002281 | macrophage activation involved in immune response | 7/2334 | 21/29008 | 0.00091635249 | 0.00429121166 | 0.002415129918 |
| **1704** | KO | GO:0006700 | C21-steroid hormone biosynthetic process | 7/2334 | 21/29008 | 0.00091635249 | 0.00429121166 | 0.002415129918 |
| **1705** | KO | GO:0015868 | purine ribonucleotide transport | 7/2334 | 21/29008 | 0.00091635249 | 0.00429121166 | 0.002415129918 |
| **1706** | KO | GO:0016137 | glycoside metabolic process | 7/2334 | 21/29008 | 0.00091635249 | 0.00429121166 | 0.002415129918 |
| **1707** | KO | GO:0060333 | interferon-gamma-mediated signaling pathway | 7/2334 | 21/29008 | 0.00091635249 | 0.00429121166 | 0.002415129918 |
| **1708** | KO | GO:1905153 | regulation of membrane invagination | 7/2334 | 21/29008 | 0.00091635249 | 0.00429121166 | 0.002415129918 |
| **1709** | KO | GO:2000114 | regulation of establishment of cell polarity | 7/2334 | 21/29008 | 0.00091635249 | 0.00429121166 | 0.002415129918 |
| **1710** | KO | GO:1904659 | glucose transmembrane transport | 20/2334 | 116/29008 | 0.0009205666453 | 0.004307662962 | 0.002424388848 |
| **1711** | KO | GO:0002712 | regulation of B cell mediated immunity | 15/2334 | 76/29008 | 0.0009477002809 | 0.004427886331 | 0.002492051569 |
| **1712** | KO | GO:0002889 | regulation of immunoglobulin mediated immune response | 15/2334 | 76/29008 | 0.0009477002809 | 0.004427886331 | 0.002492051569 |
| **1713** | KO | GO:0045216 | cell-cell junction organization | 31/2334 | 213/29008 | 0.0009494205395 | 0.004432553036 | 0.002494678029 |
| **1714** | KO | GO:0007006 | mitochondrial membrane organization | 19/2334 | 108/29008 | 0.0009509687333 | 0.004436409945 | 0.00249684873 |
| **1715** | KO | GO:0034368 | protein-lipid complex remodeling | 8/2334 | 27/29008 | 0.0009585321638 | 0.004464914037 | 0.002512891072 |
| **1716** | KO | GO:0034369 | plasma lipoprotein particle remodeling | 8/2334 | 27/29008 | 0.0009585321638 | 0.004464914037 | 0.002512891072 |
| **1717** | KO | GO:0002228 | natural killer cell mediated immunity | 16/2334 | 84/29008 | 0.0009744124857 | 0.004532013862 | 0.002550655417 |
| **1718** | KO | GO:0050000 | chromosome localization | 16/2334 | 84/29008 | 0.0009744124857 | 0.004532013862 | 0.002550655417 |
| **1719** | KO | GO:0032660 | regulation of interleukin-17 production | 10/2334 | 40/29008 | 0.001002468148 | 0.004644920289 | 0.002614200101 |
| **1720** | KO | GO:0045066 | regulatory T cell differentiation | 10/2334 | 40/29008 | 0.001002468148 | 0.004644920289 | 0.002614200101 |
| **1721** | KO | GO:0051154 | negative regulation of striated muscle cell differentiation | 10/2334 | 40/29008 | 0.001002468148 | 0.004644920289 | 0.002614200101 |
| **1722** | KO | GO:0090181 | regulation of cholesterol metabolic process | 10/2334 | 40/29008 | 0.001002468148 | 0.004644920289 | 0.002614200101 |
| **1723** | KO | GO:1903573 | negative regulation of response to endoplasmic reticulum stress | 10/2334 | 40/29008 | 0.001002468148 | 0.004644920289 | 0.002614200101 |
| **1724** | KO | GO:0010883 | regulation of lipid storage | 12/2334 | 54/29008 | 0.00101287 | 0.004689580469 | 0.002639335225 |
| **1725** | KO | GO:0006013 | mannose metabolic process | 5/2334 | 11/29008 | 0.001026419013 | 0.004727102336 | 0.002660452846 |
| **1726** | KO | GO:0008611 | ether lipid biosynthetic process | 5/2334 | 11/29008 | 0.001026419013 | 0.004727102336 | 0.002660452846 |
| **1727** | KO | GO:0035336 | long-chain fatty-acyl-CoA metabolic process | 5/2334 | 11/29008 | 0.001026419013 | 0.004727102336 | 0.002660452846 |
| **1728** | KO | GO:0046504 | glycerol ether biosynthetic process | 5/2334 | 11/29008 | 0.001026419013 | 0.004727102336 | 0.002660452846 |
| **1729** | KO | GO:0097384 | cellular lipid biosynthetic process | 5/2334 | 11/29008 | 0.001026419013 | 0.004727102336 | 0.002660452846 |
| **1730** | KO | GO:0008645 | hexose transmembrane transport | 20/2334 | 117/29008 | 0.001027898555 | 0.004727102336 | 0.002660452846 |
| **1731** | KO | GO:0022600 | digestive system process | 20/2334 | 117/29008 | 0.001027898555 | 0.004727102336 | 0.002660452846 |
| **1732** | KO | GO:0042100 | B cell proliferation | 20/2334 | 117/29008 | 0.001027898555 | 0.004727102336 | 0.002660452846 |
| **1733** | KO | GO:0045446 | endothelial cell differentiation | 20/2334 | 117/29008 | 0.001027898555 | 0.004727102336 | 0.002660452846 |
| **1734** | KO | GO:1903018 | regulation of glycoprotein metabolic process | 11/2334 | 47/29008 | 0.001031294144 | 0.004739170695 | 0.00266724502 |
| **1735** | KO | GO:0007586 | digestion | 22/2334 | 134/29008 | 0.001033868871 | 0.004747451678 | 0.002671905627 |
| **1736** | KO | GO:0015800 | acidic amino acid transport | 14/2334 | 69/29008 | 0.00104305945 | 0.004782505417 | 0.002691634165 |
| **1737** | KO | GO:0150116 | regulation of cell-substrate junction organization | 14/2334 | 69/29008 | 0.00104305945 | 0.004782505417 | 0.002691634165 |
| **1738** | KO | GO:0002468 | dendritic cell antigen processing and presentation | 6/2334 | 16/29008 | 0.001064462121 | 0.004855274887 | 0.002732589433 |
| **1739** | KO | GO:0042541 | hemoglobin biosynthetic process | 6/2334 | 16/29008 | 0.001064462121 | 0.004855274887 | 0.002732589433 |
| **1740** | KO | GO:0045346 | regulation of MHC class II biosynthetic process | 6/2334 | 16/29008 | 0.001064462121 | 0.004855274887 | 0.002732589433 |
| **1741** | KO | GO:0046500 | S-adenosylmethionine metabolic process | 6/2334 | 16/29008 | 0.001064462121 | 0.004855274887 | 0.002732589433 |
| **1742** | KO | GO:0061760 | antifungal innate immune response | 6/2334 | 16/29008 | 0.001064462121 | 0.004855274887 | 0.002732589433 |
| **1743** | KO | GO:0070431 | nucleotide-binding oligomerization domain containing 2 signaling pathway | 6/2334 | 16/29008 | 0.001064462121 | 0.004855274887 | 0.002732589433 |
| **1744** | KO | GO:1902043 | positive regulation of extrinsic apoptotic signaling pathway via death domain receptors | 6/2334 | 16/29008 | 0.001064462121 | 0.004855274887 | 0.002732589433 |
| **1745** | KO | GO:0060560 | developmental growth involved in morphogenesis | 38/2334 | 280/29008 | 0.001065727251 | 0.004857439339 | 0.002733807605 |
| **1746** | KO | GO:0007178 | transmembrane receptor protein serine/threonine kinase signaling pathway | 49/2334 | 387/29008 | 0.00108146225 | 0.004925503383 | 0.00277211462 |
| **1747** | KO | GO:0010976 | positive regulation of neuron projection development | 32/2334 | 224/29008 | 0.001089912456 | 0.004960312691 | 0.00279170559 |
| **1748** | KO | GO:0061097 | regulation of protein tyrosine kinase activity | 15/2334 | 77/29008 | 0.001090814833 | 0.004960744881 | 0.002791948831 |
| **1749** | KO | GO:2001243 | negative regulation of intrinsic apoptotic signaling pathway | 18/2334 | 101/29008 | 0.00109563192 | 0.00497896636 | 0.002802204032 |
| **1750** | KO | GO:0071695 | anatomical structure maturation | 39/2334 | 290/29008 | 0.001098836196 | 0.004989837096 | 0.002808322173 |
| **1751** | KO | GO:0009101 | glycoprotein biosynthetic process | 37/2334 | 271/29008 | 0.001101841748 | 0.004999790028 | 0.002813923767 |
| **1752** | KO | GO:0050679 | positive regulation of epithelial cell proliferation | 31/2334 | 215/29008 | 0.001108850351 | 0.005027879379 | 0.002829732689 |
| **1753** | KO | GO:0043648 | dicarboxylic acid metabolic process | 16/2334 | 85/29008 | 0.00111218227 | 0.005039268339 | 0.002836142491 |
| **1754** | KO | GO:0002067 | glandular epithelial cell differentiation | 13/2334 | 62/29008 | 0.001131051335 | 0.005105936372 | 0.00287366382 |
| **1755** | KO | GO:0043407 | negative regulation of MAP kinase activity | 13/2334 | 62/29008 | 0.001131051335 | 0.005105936372 | 0.00287366382 |
| **1756** | KO | GO:0046850 | regulation of bone remodeling | 13/2334 | 62/29008 | 0.001131051335 | 0.005105936372 | 0.00287366382 |
| **1757** | KO | GO:0051058 | negative regulation of small GTPase mediated signal transduction | 13/2334 | 62/29008 | 0.001131051335 | 0.005105936372 | 0.00287366382 |
| **1758** | KO | GO:0051784 | negative regulation of nuclear division | 13/2334 | 62/29008 | 0.001131051335 | 0.005105936372 | 0.00287366382 |
| **1759** | KO | GO:0045920 | negative regulation of exocytosis | 9/2334 | 34/29008 | 0.00114575195 | 0.005160923741 | 0.002904611172 |
| **1760** | KO | GO:0061842 | microtubule organizing center localization | 9/2334 | 34/29008 | 0.00114575195 | 0.005160923741 | 0.002904611172 |
| **1761** | KO | GO:0090504 | epiboly | 9/2334 | 34/29008 | 0.00114575195 | 0.005160923741 | 0.002904611172 |
| **1762** | KO | GO:0030518 | intracellular steroid hormone receptor signaling pathway | 19/2334 | 110/29008 | 0.001193564401 | 0.005372351413 | 0.00302360444 |
| **1763** | KO | GO:0046324 | regulation of glucose import | 14/2334 | 70/29008 | 0.001208785123 | 0.005428929674 | 0.003055447159 |
| **1764** | KO | GO:0097194 | execution phase of apoptosis | 14/2334 | 70/29008 | 0.001208785123 | 0.005428929674 | 0.003055447159 |
| **1765** | KO | GO:1901616 | organic hydroxy compound catabolic process | 14/2334 | 70/29008 | 0.001208785123 | 0.005428929674 | 0.003055447159 |
| **1766** | KO | GO:0046467 | membrane lipid biosynthetic process | 21/2334 | 127/29008 | 0.001210733429 | 0.00542928252 | 0.003055645744 |
| **1767** | KO | GO:0051101 | regulation of DNA binding | 21/2334 | 127/29008 | 0.001210733429 | 0.00542928252 | 0.003055645744 |
| **1768** | KO | GO:0050830 | defense response to Gram-positive bacterium | 24/2334 | 153/29008 | 0.001211514703 | 0.00542928252 | 0.003055645744 |
| **1769** | KO | GO:0050807 | regulation of synapse organization | 36/2334 | 263/29008 | 0.001217806534 | 0.005453500979 | 0.003069276096 |
| **1770** | KO | GO:0030198 | extracellular matrix organization | 41/2334 | 311/29008 | 0.00123021066 | 0.005501607202 | 0.003096350682 |
| **1771** | KO | GO:0006636 | unsaturated fatty acid biosynthetic process | 10/2334 | 41/29008 | 0.001231394599 | 0.005501607202 | 0.003096350682 |
| **1772** | KO | GO:0060395 | SMAD protein signal transduction | 18/2334 | 102/29008 | 0.00123213078 | 0.005501607202 | 0.003096350682 |
| **1773** | KO | GO:0150115 | cell-substrate junction organization | 18/2334 | 102/29008 | 0.00123213078 | 0.005501607202 | 0.003096350682 |
| **1774** | KO | GO:0002920 | regulation of humoral immune response | 11/2334 | 48/29008 | 0.001241906543 | 0.005520831097 | 0.003107170051 |
| **1775** | KO | GO:0033628 | regulation of cell adhesion mediated by integrin | 11/2334 | 48/29008 | 0.001241906543 | 0.005520831097 | 0.003107170051 |
| **1776** | KO | GO:0045061 | thymic T cell selection | 8/2334 | 28/29008 | 0.001248166806 | 0.005520831097 | 0.003107170051 |
| **1777** | KO | GO:0045724 | positive regulation of cilium assembly | 8/2334 | 28/29008 | 0.001248166806 | 0.005520831097 | 0.003107170051 |
| **1778** | KO | GO:0098581 | detection of external biotic stimulus | 8/2334 | 28/29008 | 0.001248166806 | 0.005520831097 | 0.003107170051 |
| **1779** | KO | GO:1902932 | positive regulation of alcohol biosynthetic process | 8/2334 | 28/29008 | 0.001248166806 | 0.005520831097 | 0.003107170051 |
| **1780** | KO | GO:1903902 | positive regulation of viral life cycle | 8/2334 | 28/29008 | 0.001248166806 | 0.005520831097 | 0.003107170051 |
| **1781** | KO | GO:2000178 | negative regulation of neural precursor cell proliferation | 8/2334 | 28/29008 | 0.001248166806 | 0.005520831097 | 0.003107170051 |
| **1782** | KO | GO:0006656 | phosphatidylcholine biosynthetic process | 7/2334 | 22/29008 | 0.001251307826 | 0.005520831097 | 0.003107170051 |
| **1783** | KO | GO:0009158 | ribonucleoside monophosphate catabolic process | 7/2334 | 22/29008 | 0.001251307826 | 0.005520831097 | 0.003107170051 |
| **1784** | KO | GO:0034143 | regulation of toll-like receptor 4 signaling pathway | 7/2334 | 22/29008 | 0.001251307826 | 0.005520831097 | 0.003107170051 |
| **1785** | KO | GO:0034377 | plasma lipoprotein particle assembly | 7/2334 | 22/29008 | 0.001251307826 | 0.005520831097 | 0.003107170051 |
| **1786** | KO | GO:0071404 | cellular response to low-density lipoprotein particle stimulus | 7/2334 | 22/29008 | 0.001251307826 | 0.005520831097 | 0.003107170051 |
| **1787** | KO | GO:0072529 | pyrimidine-containing compound catabolic process | 7/2334 | 22/29008 | 0.001251307826 | 0.005520831097 | 0.003107170051 |
| **1788** | KO | GO:0098869 | cellular oxidant detoxification | 7/2334 | 22/29008 | 0.001251307826 | 0.005520831097 | 0.003107170051 |
| **1789** | KO | GO:0150104 | transport across blood-brain barrier | 7/2334 | 22/29008 | 0.001251307826 | 0.005520831097 | 0.003107170051 |
| **1790** | KO | GO:0006919 | activation of cysteine-type endopeptidase activity involved in apoptotic process | 15/2334 | 78/29008 | 0.001251711868 | 0.005520831097 | 0.003107170051 |
| **1791** | KO | GO:0030278 | regulation of ossification | 22/2334 | 136/29008 | 0.00126203304 | 0.00556236083 | 0.003130543333 |
| **1792** | KO | GO:0015749 | monosaccharide transmembrane transport | 20/2334 | 119/29008 | 0.001275023486 | 0.00561558731 | 0.003160499643 |
| **1793** | KO | GO:0065004 | protein-DNA complex assembly | 23/2334 | 145/29008 | 0.001300695844 | 0.005724552481 | 0.003221826155 |
| **1794** | KO | GO:0043062 | extracellular structure organization | 41/2334 | 312/29008 | 0.001308652676 | 0.005755448849 | 0.00323921489 |
| **1795** | KO | GO:0030865 | cortical cytoskeleton organization | 13/2334 | 63/29008 | 0.001321749224 | 0.005808889294 | 0.00326929162 |
| **1796** | KO | GO:1901989 | positive regulation of cell cycle phase transition | 19/2334 | 111/29008 | 0.001333440956 | 0.005856083798 | 0.003295853084 |
| **1797** | KO | GO:0007098 | centrosome cycle | 21/2334 | 128/29008 | 0.001340644131 | 0.005879313022 | 0.003308926685 |
| **1798** | KO | GO:0045807 | positive regulation of endocytosis | 21/2334 | 128/29008 | 0.001340644131 | 0.005879313022 | 0.003308926685 |
| **1799** | KO | GO:0060070 | canonical Wnt signaling pathway | 40/2334 | 303/29008 | 0.001363969703 | 0.005977339411 | 0.00336409676 |
| **1800** | KO | GO:0014066 | regulation of phosphatidylinositol 3-kinase signaling | 18/2334 | 103/29008 | 0.001382805574 | 0.006051251743 | 0.00340569524 |
| **1801** | KO | GO:0070167 | regulation of biomineral tissue development | 18/2334 | 103/29008 | 0.001382805574 | 0.006051251743 | 0.00340569524 |
| **1802** | KO | GO:0045229 | external encapsulating structure organization | 41/2334 | 313/29008 | 0.001391440477 | 0.006084704835 | 0.003424522921 |
| **1803** | KO | GO:0034219 | carbohydrate transmembrane transport | 20/2334 | 120/29008 | 0.001416507911 | 0.006189631634 | 0.003483576603 |
| **1804** | KO | GO:0045685 | regulation of glial cell differentiation | 17/2334 | 95/29008 | 0.001419133353 | 0.006189631634 | 0.003483576603 |
| **1805** | KO | GO:0071774 | response to fibroblast growth factor | 17/2334 | 95/29008 | 0.001419133353 | 0.006189631634 | 0.003483576603 |
| **1806** | KO | GO:0072577 | endothelial cell apoptotic process | 12/2334 | 56/29008 | 0.001419464676 | 0.006189631634 | 0.003483576603 |
| **1807** | KO | GO:0071333 | cellular response to glucose stimulus | 23/2334 | 146/29008 | 0.001428693169 | 0.00622545449 | 0.003503738007 |
| **1808** | KO | GO:0071402 | cellular response to lipoprotein particle stimulus | 9/2334 | 35/29008 | 0.001433500822 | 0.006241976649 | 0.003513036817 |
| **1809** | KO | GO:0032890 | regulation of organic acid transport | 16/2334 | 87/29008 | 0.001437523064 | 0.006255057863 | 0.003520399034 |
| **1810** | KO | GO:0072006 | nephron development | 24/2334 | 155/29008 | 0.001453698872 | 0.006320966645 | 0.003557493049 |
| **1811** | KO | GO:0051896 | regulation of protein kinase B signaling | 26/2334 | 173/29008 | 0.001472363753 | 0.006397597525 | 0.003600621551 |
| **1812** | KO | GO:0048640 | negative regulation of developmental growth | 21/2334 | 129/29008 | 0.001482281051 | 0.00643613765 | 0.003622312257 |
| **1813** | KO | GO:0032210 | regulation of telomere maintenance via telomerase | 11/2334 | 49/29008 | 0.001486603366 | 0.006447582624 | 0.003628753584 |
| **1814** | KO | GO:0045639 | positive regulation of myeloid cell differentiation | 19/2334 | 112/29008 | 0.001487015719 | 0.006447582624 | 0.003628753584 |
| **1815** | KO | GO:0060251 | regulation of glial cell proliferation | 10/2334 | 42/29008 | 0.001501174417 | 0.006504383371 | 0.003660721521 |
| **1816** | KO | GO:0031032 | actomyosin structure organization | 30/2334 | 210/29008 | 0.001529943037 | 0.006596022691 | 0.003712296899 |
| **1817** | KO | GO:0010566 | regulation of ketone biosynthetic process | 6/2334 | 17/29008 | 0.001533710307 | 0.006596022691 | 0.003712296899 |
| **1818** | KO | GO:0045059 | positive thymic T cell selection | 6/2334 | 17/29008 | 0.001533710307 | 0.006596022691 | 0.003712296899 |
| **1819** | KO | GO:0045064 | T-helper 2 cell differentiation | 6/2334 | 17/29008 | 0.001533710307 | 0.006596022691 | 0.003712296899 |
| **1820** | KO | GO:0061615 | glycolytic process through fructose-6-phosphate | 6/2334 | 17/29008 | 0.001533710307 | 0.006596022691 | 0.003712296899 |
| **1821** | KO | GO:0061952 | midbody abscission | 6/2334 | 17/29008 | 0.001533710307 | 0.006596022691 | 0.003712296899 |
| **1822** | KO | GO:0070206 | protein trimerization | 6/2334 | 17/29008 | 0.001533710307 | 0.006596022691 | 0.003712296899 |
| **1823** | KO | GO:1901569 | fatty acid derivative catabolic process | 6/2334 | 17/29008 | 0.001533710307 | 0.006596022691 | 0.003712296899 |
| **1824** | KO | GO:1902916 | positive regulation of protein polyubiquitination | 6/2334 | 17/29008 | 0.001533710307 | 0.006596022691 | 0.003712296899 |
| **1825** | KO | GO:0001836 | release of cytochrome c from mitochondria | 13/2334 | 64/29008 | 0.001538427818 | 0.006596022691 | 0.003712296899 |
| **1826** | KO | GO:0001954 | positive regulation of cell-matrix adhesion | 13/2334 | 64/29008 | 0.001538427818 | 0.006596022691 | 0.003712296899 |
| **1827** | KO | GO:0051310 | metaphase plate congression | 13/2334 | 64/29008 | 0.001538427818 | 0.006596022691 | 0.003712296899 |
| **1828** | KO | GO:0051893 | regulation of focal adhesion assembly | 13/2334 | 64/29008 | 0.001538427818 | 0.006596022691 | 0.003712296899 |
| **1829** | KO | GO:0070059 | intrinsic apoptotic signaling pathway in response to endoplasmic reticulum stress | 13/2334 | 64/29008 | 0.001538427818 | 0.006596022691 | 0.003712296899 |
| **1830** | KO | GO:0090109 | regulation of cell-substrate junction assembly | 13/2334 | 64/29008 | 0.001538427818 | 0.006596022691 | 0.003712296899 |
| **1831** | KO | GO:1903320 | regulation of protein modification by small protein conjugation or removal | 34/2334 | 248/29008 | 0.001592782613 | 0.006824307097 | 0.003840777278 |
| **1832** | KO | GO:0046887 | positive regulation of hormone secretion | 25/2334 | 165/29008 | 0.00160045898 | 0.006836621149 | 0.003847707729 |
| **1833** | KO | GO:0006699 | bile acid biosynthetic process | 8/2334 | 29/29008 | 0.001603350691 | 0.006836621149 | 0.003847707729 |
| **1834** | KO | GO:0032967 | positive regulation of collagen biosynthetic process | 8/2334 | 29/29008 | 0.001603350691 | 0.006836621149 | 0.003847707729 |
| **1835** | KO | GO:0034367 | protein-containing complex remodeling | 8/2334 | 29/29008 | 0.001603350691 | 0.006836621149 | 0.003847707729 |
| **1836** | KO | GO:0046365 | monosaccharide catabolic process | 8/2334 | 29/29008 | 0.001603350691 | 0.006836621149 | 0.003847707729 |
| **1837** | KO | GO:0061003 | positive regulation of dendritic spine morphogenesis | 8/2334 | 29/29008 | 0.001603350691 | 0.006836621149 | 0.003847707729 |
| **1838** | KO | GO:0002287 | alpha-beta T cell activation involved in immune response | 14/2334 | 72/29008 | 0.001606784007 | 0.006836621149 | 0.003847707729 |
| **1839** | KO | GO:0007566 | embryo implantation | 14/2334 | 72/29008 | 0.001606784007 | 0.006836621149 | 0.003847707729 |
| **1840** | KO | GO:0009201 | ribonucleoside triphosphate biosynthetic process | 14/2334 | 72/29008 | 0.001606784007 | 0.006836621149 | 0.003847707729 |
| **1841** | KO | GO:0048678 | response to axon injury | 14/2334 | 72/29008 | 0.001606784007 | 0.006836621149 | 0.003847707729 |
| **1842** | KO | GO:0006112 | energy reserve metabolic process | 16/2334 | 88/29008 | 0.001628094398 | 0.006917712297 | 0.003893346508 |
| **1843** | KO | GO:0034620 | cellular response to unfolded protein | 16/2334 | 88/29008 | 0.001628094398 | 0.006917712297 | 0.003893346508 |
| **1844** | KO | GO:0006152 | purine nucleoside catabolic process | 5/2334 | 12/29008 | 0.001643306796 | 0.006934393515 | 0.003902734837 |
| **1845** | KO | GO:0006534 | cysteine metabolic process | 5/2334 | 12/29008 | 0.001643306796 | 0.006934393515 | 0.003902734837 |
| **1846** | KO | GO:0032490 | detection of molecule of bacterial origin | 5/2334 | 12/29008 | 0.001643306796 | 0.006934393515 | 0.003902734837 |
| **1847** | KO | GO:0032825 | positive regulation of natural killer cell differentiation | 5/2334 | 12/29008 | 0.001643306796 | 0.006934393515 | 0.003902734837 |
| **1848** | KO | GO:0036005 | response to macrophage colony-stimulating factor | 5/2334 | 12/29008 | 0.001643306796 | 0.006934393515 | 0.003902734837 |
| **1849** | KO | GO:0036006 | cellular response to macrophage colony-stimulating factor stimulus | 5/2334 | 12/29008 | 0.001643306796 | 0.006934393515 | 0.003902734837 |
| **1850** | KO | GO:0048241 | epinephrine transport | 5/2334 | 12/29008 | 0.001643306796 | 0.006934393515 | 0.003902734837 |
| **1851** | KO | GO:1901503 | ether biosynthetic process | 5/2334 | 12/29008 | 0.001643306796 | 0.006934393515 | 0.003902734837 |
| **1852** | KO | GO:1902947 | regulation of tau-protein kinase activity | 5/2334 | 12/29008 | 0.001643306796 | 0.006934393515 | 0.003902734837 |
| **1853** | KO | GO:2000322 | regulation of glucocorticoid receptor signaling pathway | 5/2334 | 12/29008 | 0.001643306796 | 0.006934393515 | 0.003902734837 |
| **1854** | KO | GO:0120034 | positive regulation of plasma membrane bounded cell projection assembly | 19/2334 | 113/29008 | 0.001655338776 | 0.006980371613 | 0.003928611696 |
| **1855** | KO | GO:0050732 | negative regulation of peptidyl-tyrosine phosphorylation | 12/2334 | 57/29008 | 0.001668376479 | 0.007019487752 | 0.003950626587 |
| **1856** | KO | GO:0006595 | polyamine metabolic process | 7/2334 | 23/29008 | 0.001674897305 | 0.007019487752 | 0.003950626587 |
| **1857** | KO | GO:0009129 | pyrimidine nucleoside monophosphate metabolic process | 7/2334 | 23/29008 | 0.001674897305 | 0.007019487752 | 0.003950626587 |
| **1858** | KO | GO:0031649 | heat generation | 7/2334 | 23/29008 | 0.001674897305 | 0.007019487752 | 0.003950626587 |
| **1859** | KO | GO:0046641 | positive regulation of alpha-beta T cell proliferation | 7/2334 | 23/29008 | 0.001674897305 | 0.007019487752 | 0.003950626587 |
| **1860** | KO | GO:0051156 | glucose 6-phosphate metabolic process | 7/2334 | 23/29008 | 0.001674897305 | 0.007019487752 | 0.003950626587 |
| **1861** | KO | GO:0120255 | olefinic compound biosynthetic process | 7/2334 | 23/29008 | 0.001674897305 | 0.007019487752 | 0.003950626587 |
| **1862** | KO | GO:1902410 | mitotic cytokinetic process | 7/2334 | 23/29008 | 0.001674897305 | 0.007019487752 | 0.003950626587 |
| **1863** | KO | GO:1903428 | positive regulation of reactive oxygen species biosynthetic process | 7/2334 | 23/29008 | 0.001674897305 | 0.007019487752 | 0.003950626587 |
| **1864** | KO | GO:2001056 | positive regulation of cysteine-type endopeptidase activity | 23/2334 | 148/29008 | 0.001717317236 | 0.007192363395 | 0.004047922449 |
| **1865** | KO | GO:0110149 | regulation of biomineralization | 18/2334 | 105/29008 | 0.001731342408 | 0.007246163319 | 0.004078201498 |
| **1866** | KO | GO:0090277 | positive regulation of peptide hormone secretion | 20/2334 | 122/29008 | 0.001739888123 | 0.007276972519 | 0.004095541174 |
| **1867** | KO | GO:0007292 | female gamete generation | 25/2334 | 166/29008 | 0.001743250116 | 0.007286073954 | 0.004100663538 |
| **1868** | KO | GO:0002066 | columnar/cuboidal epithelial cell development | 11/2334 | 50/29008 | 0.001769381125 | 0.007385242956 | 0.004156476684 |
| **1869** | KO | GO:0007520 | myoblast fusion | 11/2334 | 50/29008 | 0.001769381125 | 0.007385242956 | 0.004156476684 |
| **1870** | KO | GO:0001779 | natural killer cell differentiation | 9/2334 | 36/29008 | 0.001776575815 | 0.007405211539 | 0.00416771517 |
| **1871** | KO | GO:0045589 | regulation of regulatory T cell differentiation | 9/2334 | 36/29008 | 0.001776575815 | 0.007405211539 | 0.00416771517 |
| **1872** | KO | GO:0090559 | regulation of membrane permeability | 17/2334 | 97/29008 | 0.001794616519 | 0.007475338234 | 0.004207183062 |
| **1873** | KO | GO:0032874 | positive regulation of stress-activated MAPK cascade | 21/2334 | 131/29008 | 0.00180412059 | 0.007509835301 | 0.004226598301 |
| **1874** | KO | GO:0000082 | G1/S transition of mitotic cell cycle | 28/2334 | 194/29008 | 0.001845458085 | 0.007676705803 | 0.00432051442 |
| **1875** | KO | GO:0043271 | negative regulation of ion transport | 26/2334 | 176/29008 | 0.001888430781 | 0.007850147983 | 0.004418129133 |
| **1876** | KO | GO:0060419 | heart growth | 20/2334 | 123/29008 | 0.001923749603 | 0.007991560217 | 0.004497717125 |
| **1877** | KO | GO:0015844 | monoamine transport | 18/2334 | 106/29008 | 0.001931689599 | 0.008019122228 | 0.004513229256 |
| **1878** | KO | GO:0042692 | muscle cell differentiation | 52/2334 | 428/29008 | 0.001935663843 | 0.008030194902 | 0.004519461049 |
| **1879** | KO | GO:0045861 | negative regulation of proteolysis | 45/2334 | 358/29008 | 0.001942898368 | 0.008054768944 | 0.004533291526 |
| **1880** | KO | GO:0006754 | ATP biosynthetic process | 12/2334 | 58/29008 | 0.001952072864 | 0.008081897357 | 0.004548559625 |
| **1881** | KO | GO:1904591 | positive regulation of protein import | 12/2334 | 58/29008 | 0.001952072864 | 0.008081897357 | 0.004548559625 |
| **1882** | KO | GO:0051492 | regulation of stress fiber assembly | 17/2334 | 98/29008 | 0.002011631503 | 0.008322871349 | 0.004684181809 |
| **1883** | KO | GO:0007179 | transforming growth factor beta receptor signaling pathway | 27/2334 | 186/29008 | 0.002024397311 | 0.008364423051 | 0.004707567455 |
| **1884** | KO | GO:0051302 | regulation of cell division | 27/2334 | 186/29008 | 0.002024397311 | 0.008364423051 | 0.004707567455 |
| **1885** | KO | GO:0001516 | prostaglandin biosynthetic process | 8/2334 | 30/29008 | 0.002033967794 | 0.008364590446 | 0.004707661667 |
| **1886** | KO | GO:0039528 | cytoplasmic pattern recognition receptor signaling pathway in response to virus | 8/2334 | 30/29008 | 0.002033967794 | 0.008364590446 | 0.004707661667 |
| **1887** | KO | GO:0046457 | prostanoid biosynthetic process | 8/2334 | 30/29008 | 0.002033967794 | 0.008364590446 | 0.004707661667 |
| **1888** | KO | GO:0071353 | cellular response to interleukin-4 | 8/2334 | 30/29008 | 0.002033967794 | 0.008364590446 | 0.004707661667 |
| **1889** | KO | GO:0090025 | regulation of monocyte chemotaxis | 8/2334 | 30/29008 | 0.002033967794 | 0.008364590446 | 0.004707661667 |
| **1890** | KO | GO:0097345 | mitochondrial outer membrane permeabilization | 8/2334 | 30/29008 | 0.002033967794 | 0.008364590446 | 0.004707661667 |
| **1891** | KO | GO:1904752 | regulation of vascular associated smooth muscle cell migration | 8/2334 | 30/29008 | 0.002033967794 | 0.008364590446 | 0.004707661667 |
| **1892** | KO | GO:0016239 | positive regulation of macroautophagy | 13/2334 | 66/29008 | 0.002060434708 | 0.008467664027 | 0.004765672343 |
| **1893** | KO | GO:0031214 | biomineral tissue development | 25/2334 | 168/29008 | 0.002061787986 | 0.008467664027 | 0.004765672343 |
| **1894** | KO | GO:0034250 | positive regulation of cellular amide metabolic process | 24/2334 | 159/29008 | 0.002064579117 | 0.008473462989 | 0.004768936047 |
| **1895** | KO | GO:0010827 | regulation of glucose transmembrane transport | 16/2334 | 90/29008 | 0.002073190356 | 0.008497452667 | 0.004782437639 |
| **1896** | KO | GO:0031960 | response to corticosteroid | 16/2334 | 90/29008 | 0.002073190356 | 0.008497452667 | 0.004782437639 |
| **1897** | KO | GO:0030324 | lung development | 32/2334 | 233/29008 | 0.002084861187 | 0.008539591423 | 0.004806153685 |
| **1898** | KO | GO:0046456 | icosanoid biosynthetic process | 11/2334 | 51/29008 | 0.002094497915 | 0.008567639941 | 0.004821939626 |
| **1899** | KO | GO:0090207 | regulation of triglyceride metabolic process | 11/2334 | 51/29008 | 0.002094497915 | 0.008567639941 | 0.004821939626 |
| **1900** | KO | GO:0001885 | endothelial cell development | 14/2334 | 74/29008 | 0.002108196415 | 0.008590964384 | 0.004835066818 |
| **1901** | KO | GO:0046824 | positive regulation of nucleocytoplasmic transport | 14/2334 | 74/29008 | 0.002108196415 | 0.008590964384 | 0.004835066818 |
| **1902** | KO | GO:0051298 | centrosome duplication | 14/2334 | 74/29008 | 0.002108196415 | 0.008590964384 | 0.004835066818 |
| **1903** | KO | GO:0002381 | immunoglobulin production involved in immunoglobulin-mediated immune response | 15/2334 | 82/29008 | 0.002108589566 | 0.008590964384 | 0.004835066818 |
| **1904** | KO | GO:0009142 | nucleoside triphosphate biosynthetic process | 15/2334 | 82/29008 | 0.002108589566 | 0.008590964384 | 0.004835066818 |
| **1905** | KO | GO:0072332 | intrinsic apoptotic signaling pathway by p53 class mediator | 15/2334 | 82/29008 | 0.002108589566 | 0.008590964384 | 0.004835066818 |
| **1906** | KO | GO:0046928 | regulation of neurotransmitter secretion | 20/2334 | 124/29008 | 0.002123780946 | 0.008641397438 | 0.004863450963 |
| **1907** | KO | GO:1901890 | positive regulation of cell junction assembly | 20/2334 | 124/29008 | 0.002123780946 | 0.008641397438 | 0.004863450963 |
| **1908** | KO | GO:0005980 | glycogen catabolic process | 6/2334 | 18/29008 | 0.002145097157 | 0.008682132369 | 0.004886376924 |
| **1909** | KO | GO:0009251 | glucan catabolic process | 6/2334 | 18/29008 | 0.002145097157 | 0.008682132369 | 0.004886376924 |
| **1910** | KO | GO:0030033 | microvillus assembly | 6/2334 | 18/29008 | 0.002145097157 | 0.008682132369 | 0.004886376924 |
| **1911** | KO | GO:0030730 | sequestering of triglyceride | 6/2334 | 18/29008 | 0.002145097157 | 0.008682132369 | 0.004886376924 |
| **1912** | KO | GO:0050855 | regulation of B cell receptor signaling pathway | 6/2334 | 18/29008 | 0.002145097157 | 0.008682132369 | 0.004886376924 |
| **1913** | KO | GO:0060253 | negative regulation of glial cell proliferation | 6/2334 | 18/29008 | 0.002145097157 | 0.008682132369 | 0.004886376924 |
| **1914** | KO | GO:0070423 | nucleotide-binding oligomerization domain containing signaling pathway | 6/2334 | 18/29008 | 0.002145097157 | 0.008682132369 | 0.004886376924 |
| **1915** | KO | GO:1900225 | regulation of NLRP3 inflammasome complex assembly | 6/2334 | 18/29008 | 0.002145097157 | 0.008682132369 | 0.004886376924 |
| **1916** | KO | GO:0006576 | cellular biogenic amine metabolic process | 18/2334 | 107/29008 | 0.002151184392 | 0.008701038119 | 0.004897017239 |
| **1917** | KO | GO:0002724 | regulation of T cell cytokine production | 9/2334 | 37/29008 | 0.002182274293 | 0.008765896878 | 0.004933520293 |
| **1918** | KO | GO:0007031 | peroxisome organization | 9/2334 | 37/29008 | 0.002182274293 | 0.008765896878 | 0.004933520293 |
| **1919** | KO | GO:0055090 | acylglycerol homeostasis | 9/2334 | 37/29008 | 0.002182274293 | 0.008765896878 | 0.004933520293 |
| **1920** | KO | GO:0070328 | triglyceride homeostasis | 9/2334 | 37/29008 | 0.002182274293 | 0.008765896878 | 0.004933520293 |
| **1921** | KO | GO:0070304 | positive regulation of stress-activated protein kinase signaling cascade | 21/2334 | 133/29008 | 0.002183434885 | 0.008765896878 | 0.004933520293 |
| **1922** | KO | GO:0090090 | negative regulation of canonical Wnt signaling pathway | 21/2334 | 133/29008 | 0.002183434885 | 0.008765896878 | 0.004933520293 |
| **1923** | KO | GO:0005978 | glycogen biosynthetic process | 10/2334 | 44/29008 | 0.002184340514 | 0.008765896878 | 0.004933520293 |
| **1924** | KO | GO:0009250 | glucan biosynthetic process | 10/2334 | 44/29008 | 0.002184340514 | 0.008765896878 | 0.004933520293 |
| **1925** | KO | GO:0010559 | regulation of glycoprotein biosynthetic process | 10/2334 | 44/29008 | 0.002184340514 | 0.008765896878 | 0.004933520293 |
| **1926** | KO | GO:0032620 | interleukin-17 production | 10/2334 | 44/29008 | 0.002184340514 | 0.008765896878 | 0.004933520293 |
| **1927** | KO | GO:0035774 | positive regulation of insulin secretion involved in cellular response to glucose stimulus | 10/2334 | 44/29008 | 0.002184340514 | 0.008765896878 | 0.004933520293 |
| **1928** | KO | GO:0046326 | positive regulation of glucose import | 10/2334 | 44/29008 | 0.002184340514 | 0.008765896878 | 0.004933520293 |
| **1929** | KO | GO:0007269 | neurotransmitter secretion | 27/2334 | 187/29008 | 0.002188627946 | 0.008765925748 | 0.004933536541 |
| **1930** | KO | GO:0010951 | negative regulation of endopeptidase activity | 27/2334 | 187/29008 | 0.002188627946 | 0.008765925748 | 0.004933536541 |
| **1931** | KO | GO:0099643 | signal release from synapse | 27/2334 | 187/29008 | 0.002188627946 | 0.008765925748 | 0.004933536541 |
| **1932** | KO | GO:0006401 | RNA catabolic process | 36/2334 | 272/29008 | 0.002196365012 | 0.008790748537 | 0.004947507015 |
| **1933** | KO | GO:0002495 | antigen processing and presentation of peptide antigen via MHC class II | 7/2334 | 24/29008 | 0.002201979492 | 0.008790748537 | 0.004947507015 |
| **1934** | KO | GO:0006691 | leukotriene metabolic process | 7/2334 | 24/29008 | 0.002201979492 | 0.008790748537 | 0.004947507015 |
| **1935** | KO | GO:0045980 | negative regulation of nucleotide metabolic process | 7/2334 | 24/29008 | 0.002201979492 | 0.008790748537 | 0.004947507015 |
| **1936** | KO | GO:0060339 | negative regulation of type I interferon-mediated signaling pathway | 7/2334 | 24/29008 | 0.002201979492 | 0.008790748537 | 0.004947507015 |
| **1937** | KO | GO:0051604 | protein maturation | 41/2334 | 321/29008 | 0.002235590675 | 0.008919135784 | 0.005019764434 |
| **1938** | KO | GO:0010950 | positive regulation of endopeptidase activity | 25/2334 | 169/29008 | 0.00223885075 | 0.008926345883 | 0.005023822338 |
| **1939** | KO | GO:0001938 | positive regulation of endothelial cell proliferation | 17/2334 | 99/29008 | 0.002250204444 | 0.00896579514 | 0.005046024711 |
| **1940** | KO | GO:0002820 | negative regulation of adaptive immune response | 12/2334 | 59/29008 | 0.002274086245 | 0.009049213658 | 0.005092973353 |
| **1941** | KO | GO:1903793 | positive regulation of anion transport | 12/2334 | 59/29008 | 0.002274086245 | 0.009049213658 | 0.005092973353 |
| **1942** | KO | GO:0009199 | ribonucleoside triphosphate metabolic process | 16/2334 | 91/29008 | 0.002331238652 | 0.009264637955 | 0.005214215954 |
| **1943** | KO | GO:0040014 | regulation of multicellular organism growth | 16/2334 | 91/29008 | 0.002331238652 | 0.009264637955 | 0.005214215954 |
| **1944** | KO | GO:0051384 | response to glucocorticoid | 15/2334 | 83/29008 | 0.002385914127 | 0.009475795991 | 0.005333057468 |
| **1945** | KO | GO:0014902 | myotube differentiation | 21/2334 | 134/29008 | 0.002397071047 | 0.009513956405 | 0.005354534469 |
| **1946** | KO | GO:0060485 | mesenchyme development | 38/2334 | 293/29008 | 0.002423506331 | 0.009612668108 | 0.005410090244 |
| **1947** | KO | GO:0110148 | biomineralization | 25/2334 | 170/29008 | 0.002428697424 | 0.009627043209 | 0.005418180671 |
| **1948** | KO | GO:0046328 | regulation of JNK cascade | 22/2334 | 143/29008 | 0.002431052794 | 0.009630166581 | 0.005419938531 |
| **1949** | KO | GO:0046460 | neutral lipid biosynthetic process | 11/2334 | 52/29008 | 0.002466468457 | 0.009747030369 | 0.005485710451 |
| **1950** | KO | GO:0046463 | acylglycerol biosynthetic process | 11/2334 | 52/29008 | 0.002466468457 | 0.009747030369 | 0.005485710451 |
| **1951** | KO | GO:0050798 | activated T cell proliferation | 11/2334 | 52/29008 | 0.002466468457 | 0.009747030369 | 0.005485710451 |
| **1952** | KO | GO:0006814 | sodium ion transport | 33/2334 | 245/29008 | 0.002466899776 | 0.009747030369 | 0.005485710451 |
| **1953** | KO | GO:0006957 | complement activation, alternative pathway | 5/2334 | 13/29008 | 0.002494314514 | 0.009754976685 | 0.005490182705 |
| **1954** | KO | GO:0009071 | serine family amino acid catabolic process | 5/2334 | 13/29008 | 0.002494314514 | 0.009754976685 | 0.005490182705 |
| **1955** | KO | GO:0009120 | deoxyribonucleoside metabolic process | 5/2334 | 13/29008 | 0.002494314514 | 0.009754976685 | 0.005490182705 |
| **1956** | KO | GO:0031953 | negative regulation of protein autophosphorylation | 5/2334 | 13/29008 | 0.002494314514 | 0.009754976685 | 0.005490182705 |
| **1957** | KO | GO:0032905 | transforming growth factor beta1 production | 5/2334 | 13/29008 | 0.002494314514 | 0.009754976685 | 0.005490182705 |
| **1958** | KO | GO:0042532 | negative regulation of tyrosine phosphorylation of STAT protein | 5/2334 | 13/29008 | 0.002494314514 | 0.009754976685 | 0.005490182705 |
| **1959** | KO | GO:0045628 | regulation of T-helper 2 cell differentiation | 5/2334 | 13/29008 | 0.002494314514 | 0.009754976685 | 0.005490182705 |
| **1960** | KO | GO:0046642 | negative regulation of alpha-beta T cell proliferation | 5/2334 | 13/29008 | 0.002494314514 | 0.009754976685 | 0.005490182705 |
| **1961** | KO | GO:0051315 | attachment of mitotic spindle microtubules to kinetochore | 5/2334 | 13/29008 | 0.002494314514 | 0.009754976685 | 0.005490182705 |
| **1962** | KO | GO:0051974 | negative regulation of telomerase activity | 5/2334 | 13/29008 | 0.002494314514 | 0.009754976685 | 0.005490182705 |
| **1963** | KO | GO:0060340 | positive regulation of type I interferon-mediated signaling pathway | 5/2334 | 13/29008 | 0.002494314514 | 0.009754976685 | 0.005490182705 |
| **1964** | KO | GO:0060368 | regulation of Fc receptor mediated stimulatory signaling pathway | 5/2334 | 13/29008 | 0.002494314514 | 0.009754976685 | 0.005490182705 |
| **1965** | KO | GO:0071380 | cellular response to prostaglandin E stimulus | 5/2334 | 13/29008 | 0.002494314514 | 0.009754976685 | 0.005490182705 |
| **1966** | KO | GO:1901679 | nucleotide transmembrane transport | 5/2334 | 13/29008 | 0.002494314514 | 0.009754976685 | 0.005490182705 |
| **1967** | KO | GO:1903238 | positive regulation of leukocyte tethering or rolling | 5/2334 | 13/29008 | 0.002494314514 | 0.009754976685 | 0.005490182705 |
| **1968** | KO | GO:1904729 | regulation of intestinal lipid absorption | 5/2334 | 13/29008 | 0.002494314514 | 0.009754976685 | 0.005490182705 |
| **1969** | KO | GO:0044070 | regulation of anion transport | 19/2334 | 117/29008 | 0.002499003322 | 0.009767096955 | 0.005497004094 |
| **1970** | KO | GO:0099003 | vesicle-mediated transport in synapse | 34/2334 | 255/29008 | 0.002535039427 | 0.009901641601 | 0.00557272695 |
| **1971** | KO | GO:0008217 | regulation of blood pressure | 30/2334 | 217/29008 | 0.002547045181 | 0.00991811885 | 0.005582000483 |
| **1972** | KO | GO:0006767 | water-soluble vitamin metabolic process | 8/2334 | 31/29008 | 0.002550557907 | 0.00991811885 | 0.005582000483 |
| **1973** | KO | GO:0015721 | bile acid and bile salt transport | 8/2334 | 31/29008 | 0.002550557907 | 0.00991811885 | 0.005582000483 |
| **1974** | KO | GO:0035094 | response to nicotine | 8/2334 | 31/29008 | 0.002550557907 | 0.00991811885 | 0.005582000483 |
| **1975** | KO | GO:0051923 | sulfation | 8/2334 | 31/29008 | 0.002550557907 | 0.00991811885 | 0.005582000483 |
| **1976** | KO | GO:0062125 | regulation of mitochondrial gene expression | 8/2334 | 31/29008 | 0.002550557907 | 0.00991811885 | 0.005582000483 |
| **1977** | KO | GO:0072337 | modified amino acid transport | 8/2334 | 31/29008 | 0.002550557907 | 0.00991811885 | 0.005582000483 |
| **1978** | KO | GO:0030323 | respiratory tube development | 32/2334 | 236/29008 | 0.002556749479 | 0.009935906892 | 0.005592011743 |
