# Supplementary material for: A hepatocyte-specific transcriptional program driven by Rela and Stat3 exacerbates experimental colitis in mice by modulating bile synthesis
Source: eLife. 2024 Aug 13;12:RP93273. doi: 10.7554/eLife.93273 (PMC11321761; doi:10.7554/eLife.93273)
Supplement: Figure 3—source data 2. [file elife-93273-fig3-data2.docx]

| **RT-qPCR** |  |  |  | **P value for cyp7b1 fc_wt vs fc_dt** |  |
| --- | --- | --- | --- | --- | --- |
| **cyp7b1** | **fc_wt** | **fc_dt** |  | P value | 0.0157 |
|  | 4.237852 | 0.352195 |  | P value summary | * |
|  | 6.423383 | 0.298909 |  | Significantly different (P < 0.05)? | Yes |
|  | 5.910718 | 0.423699 |  | One- or two-tailed P value? | Two-tailed |
|  |  |  |  | t, df | t=7.817, df=2.012 |
|  |  |  |  |  |  |
|  |  |  |  |  |  |
|  |  |  |  |  |  |
|  |  |  |  |  |  |
|  |  |  |  | **P value for cyp7a1 fc_wt vs ko_dt** |  |
| **cyp7a1** | **fc_wt** | **fc_dt** |  | P value | 0.0318 |
|  | 2.016239 | 0.022701 |  | P value summary | * |
|  | 1.139447 | 0.065557 |  | Significantly different (P < 0.05)? | Yes |
|  | 2.175982 | 0.20717 |  | One- or two-tailed P value? | Two-tailed |
|  |  |  |  | t, df | t=5.134, df=2.120 |
|  |  |  |  |  |  |
|  |  |  |  |  |  |
|  |  |  |  |  |  |
|  |  |  |  |  |  |
|  |  |  |  | **P value for cyp27a1 fc_wt vs ko_dt** |  |
| **cyp27a1** | **fc_wt** | **fc_dt** |  | P value | 0.0461 |
|  | 2.181015 | 0.000866 |  | P value summary | * |
|  | 3.215404 | 0.306367 |  | Significantly different (P < 0.05)? | Yes |
|  | 4.66432 | 0.339936 |  | One- or two-tailed P value? | Two-tailed |
|  |  |  |  | Welch-corrected t, df | t=4.309, df=2.090 |
|  |  |  |  |  |  |
|  |  |  |  |  |  |
|  |  |  |  |  |  |
|  |  |  |  |  |  |
|  |  |  |  | **P value for cyp8b1 fc_wt vs ko_dt** |  |
| **cyp8b1** | **fc_wt** | **fc_dt** |  | P value | 0.0371 |
|  | 3.473503 | 0.589383 |  | P value summary | * |
|  | 4.015037 | 0.842816 |  | Significantly different (P < 0.05)? | Yes |
|  | 5.952505 | 0.860745 |  | One- or two-tailed P value? | Two-tailed |
|  |  |  |  | Welch-corrected t, df | t=4.905, df=2.054 |
|  |  |  |  |  |  |
| **code** |  |  |  |  |  |
| **wc** | **wildtype control** |  |  |  |  |
| **wt** | **wild type treated** |  |  |  |  |
| **kc** | **knockout control** |  |  |  |  |
| **kt** | **knockout treated** |  |  |  |  |
