# Supplementary material for: A hepatocyte-specific transcriptional program driven by Rela and Stat3 exacerbates experimental colitis in mice by modulating bile synthesis
Source: eLife. 2024 Aug 13;12:RP93273. doi: 10.7554/eLife.93273 (PMC11321761; doi:10.7554/eLife.93273)
Supplement: Figure 3—source data 3. [file elife-93273-fig3-data3.docx]

| **Patient biopsy - Mass Spec data** |  |  |  |  |  |
| --- | --- | --- | --- | --- | --- |
| **Dinor-chenodeoxycholic acid** |  |  |  |  |  |
| **Control** | **IBD** |  |  |  |  |
| 219.5188118 | 2743.535328 |  | **P value for Dinor-chenodeoxycholic acid control vs IBD patients** |  |  |
| 286.6285794 | 67.05972987 |  | P value | <0.0001 |  |
| 840.6453048 | 2459.041907 |  | P value summary | **** |  |
| 116.2616239 | 2664.570167 |  | Significantly different (P < 0.05)? | Yes |  |
| 251.9651437 | 1058.955299 |  | One- or two-tailed P value? | Two-tailed |  |
| 228.924489 | 1106.660988 |  | Welch-corrected t, df | t=5.969, df=33.63 |  |
| 394.4772778 | 1104.353487 |  |  |  |  |
| 246.7999407 | 1223.101934 |  |  |  |  |
| 479.7950135 | 1530.612997 |  |  |  |  |
| 1183.648982 | 336.364243 |  |  |  |  |
| 175.3351364 | 1650.637144 |  |  |  |  |
| 319.503908 | 1637.903353 |  |  |  |  |
| 524.875176 | 2172.562649 |  |  |  |  |
| 448.4621215 | 4313.937603 |  |  |  |  |
| 246.2266089 | 1543.510918 |  |  |  |  |
| 219.6689567 | 780.8103855 |  |  |  |  |
|  | 215.8782535 |  |  |  |  |
|  | 1239.546821 |  |  |  |  |
|  | 2775.122046 |  |  |  |  |
|  | 1329.23828 |  |  |  |  |
|  | 3477.365703 |  |  |  |  |
|  | 2008.089873 |  |  |  |  |
|  | 1532.51319 |  |  |  |  |
|  | 1389.952091 |  |  |  |  |
|  | 588.5063072 |  |  |  |  |
|  | 1311.936728 |  |  |  |  |
|  | 1942.094662 |  |  |  |  |
|  | 181.5137966 |  |  |  |  |
|  |  |  |  |  |  |
|  |  |  |  |  |  |
|  |  |  |  |  |  |
| **Dinor-cholic acid** |  |  |  |  |  |
| **Control** | **IBD** |  |  |  |  |
| 524.6380334 | 3012.651641 |  | **P value for Dinor-cholic acid control vs IBD patients** |  |  |
| 346.0078434 | 347.1685201 |  | P value | <0.0001 |  |
| 891.297387 | 1745.738815 |  | P value summary | **** |  |
| 363.6261389 | 2724.480061 |  | Significantly different (P < 0.05)? | Yes |  |
| 548.9109924 | 939.9510908 |  | One- or two-tailed P value? | Two-tailed |  |
| 968.6521168 | 1104.104438 |  | Welch-corrected t, df | t=5.917, df=39.25 |  |
| 944.5098005 | 1297.33882 |  |  |  |  |
| 754.3634096 | 1047.622266 |  |  |  |  |
| 960.0993719 | 1232.829388 |  |  |  |  |
| 1695.610362 | 867.3481232 |  |  |  |  |
| 790.2596131 | 2316.101851 |  |  |  |  |
| 773.0479835 | 2046.125241 |  |  |  |  |
| 1278.820625 | 2362.560477 |  |  |  |  |
| 980.7874759 | 2730.149597 |  |  |  |  |
| 925.5378018 | 1973.851964 |  |  |  |  |
| 742.8709533 | 1528.396379 |  |  |  |  |
|  | 953.4285696 |  |  |  |  |
|  | 2364.979069 |  |  |  |  |
|  | 2028.170465 |  |  |  |  |
|  | 2555.465964 |  |  |  |  |
|  | 2826.129771 |  |  |  |  |
|  | 3517.331488 |  |  |  |  |
|  | 2166.89618 |  |  |  |  |
|  | 3047.814141 |  |  |  |  |
|  | 1213.972847 |  |  |  |  |
|  | 1563.166376 |  |  |  |  |
|  | 1967.093352 |  |  |  |  |
|  | 959.7627032 |  |  |  |  |
|  |  |  |  |  |  |
