# Supplementary material for: A hepatocyte-specific transcriptional program driven by Rela and Stat3 exacerbates experimental colitis in mice by modulating bile synthesis
Source: eLife. 2024 Aug 13;12:RP93273. doi: 10.7554/eLife.93273 (PMC11321761; doi:10.7554/eLife.93273)
Supplement: Figure 3—source data 4. [file elife-93273-fig3-data4.docx]

| **Sample code** | **Sample type** | **Age (years)** | **Gender (1=Male)** | **BMI** |
| --- | --- | --- | --- | --- |
| 01-C-F | Control | 50 | 2 | 24.6 |
| 02-C-F | Control | 35 | 1 | 21.8 |
| 03-C-F | Control | 50 | 1 | 16.9 |
| 04-C-F | Control | 27 | 2 | 17.7 |
| 05-C-F | Control | 42 | 2 | 23.4 |
| 06-C-F | Control | 36 | 1 | 25 |
| 07-C-F | Control | 54 | 1 | 21.3 |
| 08-C-F | Control | 52 | 2 | 23.3 |
| 10-C-F | Control | 19 | 1 | 21.17 |
| 12-C-F | Control | 30 | 1 | 17.8 |
| 13-C-F | Control | 28 | 1 | 21.3 |
| 14-C-F | Control | 59 | 2 | 20.2 |
| 16-C-F | Control | 32 | 1 | 23.5 |
| 01-A-F | UC | 50 | 2 | 25 |
| 02-A-F | UC | 44 | 1 | 24 |
| 03-A-F | UC | 29 | 1 | 16 |
| 04-A-F | UC | 25 | 1 | 15 |
| 05-A-F | UC | 32 | 1 | 18 |
| 06-A-F | UC | 50 | 2 | 23 |
| 07-A-F | UC | 24 | 1 | 17 |
| 08-A-F | UC | 50 | 1 | 26 |
| 09-A-F | UC | 27 | 2 | 22 |
| 10-A-F | UC | 35 | 1 | 20 |
| 11-A-F | UC | 22 | 2 | 18 |
| 12-A-F | UC | 25 | 2 | 23 |
| 13-A-F | UC | 30 | 1 | 29 |
| 14-A-F | UC | 26 | 1 | 17 |
| 15-A-F | UC | 25 | 2 | 17 |
| 17-A-F | UC | 24 | 2 | 29 |
| 18-A-F | UC | 26 | 1 | 21 |
| 19-A-F | UC | 45 | 2 | 21 |
| 20-A-F | UC | 23 | 1 | 18 |
| 21-A-F | UC | 24 | 2 | 17 |
| 22-A-F | UC | 26 | 1 | 17 |
| 23-A-F | UC | 50 | 2 | 37 |
| 24-A-F | UC | 43 | 1 | 25 |
| 25-A-F | UC | 30 | 2 | 23 |
| 26-A-F | UC | 31 | 1 | 16 |
| 27-A-F | UC | 32 | 1 | 19 |
| 28-A-F | UC | 26 | 1 | 23 |
