# Supplementary material for: A hepatocyte-specific transcriptional program driven by Rela and Stat3 exacerbates experimental colitis in mice by modulating bile synthesis
Source: eLife. 2024 Aug 13;12:RP93273. doi: 10.7554/eLife.93273 (PMC11321761; doi:10.7554/eLife.93273)
Supplement: Figure 3—figure supplement 1—source data 1. [file elife-93273-fig3-figsupp1-data1.docx]

| **SGPT** | **ko_con** | **ko_d4** | **ko_d6** |
| --- | --- | --- | --- |
|  | 175.04 | 54.07 | 109.74 |
|  | 90.2 | 26.03 | 87.72 |
|  | 70.69 | 161.03 | 68.78 |
|  |  |  |  |
| **Bilurubin-D** | **ko_con** | **ko_d4** | **ko_d6** |
|  | 0.84 | 0.7 | 1.01 |
|  | 0.86 | 0.65 | 0.23 |
|  | 0.35 | 0.89 | 0.7 |
|  |  |  |  |
| **GGT** | **ko_con** | **ko_d4** | **ko_d6** |
|  | 9.09 | 6.85 | 7.86 |
|  | 6.69 | 8.87 | 2.95 |
|  | 4.49 | 6.73 | 6.5 |
|  |  |  |  |
| **SGOT** | **ko_con** | **ko_d4** | **ko_d6** |
|  | 360.18 | 83.56 | 203.42 |
|  | 142.41 | 94.23 | 99.89 |
|  | 112.87 | 194.04 | 122.82 |
