# Supplementary material for: A hepatocyte-specific transcriptional program driven by Rela and Stat3 exacerbates experimental colitis in mice by modulating bile synthesis
Source: eLife. 2024 Aug 13;12:RP93273. doi: 10.7554/eLife.93273 (PMC11321761; doi:10.7554/eLife.93273)
Supplement: Figure 4—source data 1. [file elife-93273-fig4-data1.docx]

| **Liver tissue metabolite quantification data** |  |  |  |  |  |  |  |  |
| --- | --- | --- | --- | --- | --- | --- | --- | --- |
|  | **pg/3 g liver** |  |  |  |  |  |  |  |
| **Sample id.** | **CDCA** | **CA** | **UDCA** | **𝛼-MCA** | **𝛽-MCA** |  | **t-test CA** |  |
| **Wt_dss_1** | 6354 | 70350 | 23295 | 192900 | 532050 |  | P value | 0.0214 |
| **Wt_dss_2** | 8838 | 39525 | 11026.5 | 114450 | 304200 |  | P value summary | * |
| **Wt_dss_3** | 6255 | 32610 | 12774 | 56265 | 114135 |  | Significantly different (P < 0.05)? | Yes |
| **Wt_dss_4** | 13164 | 684150 | 58890 | 294900 | 166950 |  | One- or two-tailed P value? | Two-tailed |
| **Wt_dss_5** | 6360 | 21450 | 22095 | 165855 | 516000 |  | t, df | t=3.574, df=4.210 |
|  |  |  |  |  |  |  |  |  |
| **dko_dss_1** | 765 | 7380 | 0 | 129450 | 685620 |  | **t-test CDCA** |  |
| **dko_dss_2** | 210 | 2205 | 735 | 8970 | 25515 |  | P value | 0.0044 |
| **dko_dss_3** | 1470 | 9960 | 8865 | 37320 | 85380 |  | P value summary | ** |
| **dko_dss_4** | 165 | 9570 | 1485 | 33525 | 58695 |  | Significantly different (P < 0.05)? | Yes |
| **dko_dss_5** | 969 | 7717.5 | 5436 | 13101 | 58410 |  | One- or two-tailed P value? | Two-tailed |
|  |  |  |  |  |  |  | t, df | t=5.512, df=4.268 |
|  |  |  |  |  |  |  |  |  |
|  |  |  |  |  |  |  | **t-test UDCA** |  |
|  |  |  |  |  |  |  | P value | 0.0606 |
|  |  |  |  |  |  |  | P value summary | ns |
|  |  |  |  |  |  |  | Significantly different (P < 0.05)? | No |
|  |  |  |  |  |  |  | One- or two-tailed P value? | Two-tailed |
|  |  |  |  |  |  |  | Welch-corrected t, df | t=2.527, df=4.299 |
|  |  |  |  |  |  |  |  |  |
|  |  |  |  |  |  |  | **t-test 𝛼-MCA** |  |
|  |  |  |  |  |  |  | P value | 0.0374 |
|  |  |  |  |  |  |  | P value summary | * |
|  |  |  |  |  |  |  | Significantly different (P < 0.05)? | Yes |
|  |  |  |  |  |  |  | One- or two-tailed P value? | Two-tailed |
|  |  |  |  |  |  |  | t, df | t=2.638, df=6.207 |
|  |  |  |  |  |  |  |  |  |
|  |  |  |  |  |  |  | **t-test 𝛽-MCA** |  |
|  |  |  |  |  |  |  | P value | 0.3773 |
|  |  |  |  |  |  |  | P value summary | ns |
|  |  |  |  |  |  |  | Significantly different (P < 0.05)? | No |
|  |  |  |  |  |  |  | One- or two-tailed P value? | Two-tailed |
|  |  |  |  |  |  |  | t, df | t=0.9418, df=7.077 |
