# Supplementary material for: A hepatocyte-specific transcriptional program driven by Rela and Stat3 exacerbates experimental colitis in mice by modulating bile synthesis
Source: eLife. 2024 Aug 13;12:RP93273. doi: 10.7554/eLife.93273 (PMC11321761; doi:10.7554/eLife.93273)
Supplement: Figure 4—source data 2. [file elife-93273-fig4-data2.docx]

| **Colon tissue metabolite quantification data** |  |  |  |  |  |  |  |  |
| --- | --- | --- | --- | --- | --- | --- | --- | --- |
|  | **pg/3 g colon** |  |  |  |  |  |  |  |
| **Sample id.** | **CDCA** | **CA** | **UDCA** | **𝛼-MCA** | **𝛽-MCA** |  | **t-test CA** |  |
| **Wt_dss_1** | 228900 | 220200 | 71745 | 757950 | 457200 |  | P value | 0.0856 |
| **Wt_dss_2** | 427950 | 184650 | 188250 | 2514000 | 2317500 |  | P value summary | ns |
| **Wt_dss_3** | 541650 | 134400 | 98595 | 1043100 | 1507500 |  | Significantly different (P < 0.05)? | No |
| **Wt_dss_4** | 101790 | 78690 | 24255 | 187200 | 322500 |  | One- or two-tailed P value? | Two-tailed |
| **Wt_dss_5** | 246150 | 22380 | 66540 | 404700 | 704250 |  | t, df | t=2.102, df=5.377 |
|  |  |  |  |  |  |  |  |  |
| **dko_dss_1** | 50775 | 22545 | 29685 | 143115 | 83535 |  | **t-test CDCA** |  |
| **dko_dss_2** | 52965 | 35790 | 28560 | 331950 | 339300 |  | P value | 0.0274 |
| **dko_dss_3** | 37410 | 17070 | 14794.5 | 275250 | 395250 |  | P value summary | * |
| **dko_dss_4** | 53820 | 59745 | 32430 | 381150 | 434850 |  | Significantly different (P < 0.05)? | Yes |
| **dko_dss_5** | 29865 | 99135 | 12319.5 | 158850 | 177300 |  | One- or two-tailed P value? | Two-tailed |
|  |  |  |  |  |  |  | t, df | t=3.384, df=4.030 |
|  |  |  |  |  |  |  |  |  |
|  |  |  |  |  |  |  | **t-test UDCA** |  |
|  |  |  |  |  |  |  | P value | 0.4886 |
|  |  |  |  |  |  |  | P value summary | ns |
|  |  |  |  |  |  |  | Significantly different (P < 0.05)? | No |
|  |  |  |  |  |  |  | One- or two-tailed P value? | Two-tailed |
|  |  |  |  |  |  |  | Welch-corrected t, df | t=0.7406, df=5.637 |
|  |  |  |  |  |  |  |  |  |
|  |  |  |  |  |  |  | **t-test 𝛼-MCA** |  |
|  |  |  |  |  |  |  | P value | 0.1529 |
|  |  |  |  |  |  |  | P value summary | ns |
|  |  |  |  |  |  |  | Significantly different (P < 0.05)? | No |
|  |  |  |  |  |  |  | One- or two-tailed P value? | Two-tailed |
|  |  |  |  |  |  |  | t, df | t=1.752, df=4.104 |
|  |  |  |  |  |  |  |  |  |
|  |  |  |  |  |  |  | **t-test 𝛽-MCA** |  |
|  |  |  |  |  |  |  | P value | 0.1073 |
|  |  |  |  |  |  |  | P value summary | ns |
|  |  |  |  |  |  |  | Significantly different (P < 0.05)? | No |
|  |  |  |  |  |  |  | One- or two-tailed P value? | Two-tailed |
|  |  |  |  |  |  |  | Welch-corrected t, df | t=2.035, df=4.255 |
|  |  |  |  |  |  |  |  |  |
