# Supplementary material for: A hepatocyte-specific transcriptional program driven by Rela and Stat3 exacerbates experimental colitis in mice by modulating bile synthesis
Source: eLife. 2024 Aug 13;12:RP93273. doi: 10.7554/eLife.93273 (PMC11321761; doi:10.7554/eLife.93273)
Supplement: Figure 4—source data 3. [file elife-93273-fig4-data3.docx]

| **RT-qPCR** |  |  |  |  |  |  |  |
| --- | --- | --- | --- | --- | --- | --- | --- |
| **il6** | **WT_control** | **WT_D6** | **KO_control** | **KO_D6** |  | **Unpaired t test** |  |
|  | 1 | 179.4194 | 1.29759 | 7.44533 |  | P value | 0.018 |
|  | 1 | 198.3076 | 1.531663 | 15.64162 |  | P value summary | * |
|  | 1 | 123.4909 | 0.371822 | 23.04697 |  | Significantly different (P < 0.05)? | Yes |
|  |  |  |  |  |  | One- or two-tailed P value? | Two-tailed |
|  |  |  |  |  |  | Welch-corrected t, df | t=6.621, df=2.161 |
|  |  |  |  |  |  |  |  |
|  |  |  |  |  |  |  |  |
| **tnfa** | **WT_control** | **WT_D6** | **KO_control** | **KO_D6** |  | **Unpaired t test** |  |
|  | 1 | 23.15023 | 1.358805 | 9.67506 |  | P value | 0.0102 |
|  | 1 | 28.55737 | 2.561304 | 9.887616 |  | P value summary | * |
|  | 1 | 21.45027 | 1.972799 | 7.218369 |  | Significantly different (P < 0.05)? | Yes |
|  |  |  |  |  |  | One- or two-tailed P value? | Two-tailed |
|  |  |  |  |  |  | Welch-corrected t, df | t=6.699, df=2.623 |
|  |  |  |  |  |  |  |  |
|  |  |  |  |  |  |  |  |
| **il1b** | **WT_control** | **WT_D6** | **KO_control** | **KO_D6** |  | **Unpaired t test** |  |
|  | 1 | 30.53315 | 1.435819 | 3.334384 |  | P value | 0.0278 |
|  | 1 | 41.96189 | 1.431435 | 4.090341 |  | P value summary | * |
|  | 1 | 53.23286 | 0.765428 | 6.105626 |  | Significantly different (P < 0.05)? | Yes |
|  |  |  |  |  |  | One- or two-tailed P value? | Two-tailed |
|  |  |  |  |  |  | Welch-corrected t, df | t=5.662, df=2.064 |
|  |  |  |  |  |  |  |  |
