# Supplementary material for: A hepatocyte-specific transcriptional program driven by Rela and Stat3 exacerbates experimental colitis in mice by modulating bile synthesis
Source: eLife. 2024 Aug 13;12:RP93273. doi: 10.7554/eLife.93273 (PMC11321761; doi:10.7554/eLife.93273)
Supplement: Figure 4—source data 4. [file elife-93273-fig4-data4.docx]

| **Quantification of Confocal Microscopy** |  |  |  |  |  |  |
| --- | --- | --- | --- | --- | --- | --- |
| Percentage = (no of cells with the desired signal/Total DAPI positive cells)*100 | | | |  |  |  |
| **CD11C data:-** |  |  |  |  | **Unpaired t test CD11c in wt_treated vs dKO_treated** |  |
| Wild Type | dKO | Wt % | dko% |  | P value | <0.0001 |
| 30/413 | 5/433 | 7.263923 | 1.154734 |  | P value summary | **** |
| 25/428 | 6/345 | 5.841121 | 1.73913 |  | Significantly different (P < 0.05)? | Yes |
| 30/358 | 4/320 | 8.379888 | 1.25 |  | One- or two-tailed P value? | Two-tailed |
| 28/411 | 12/800 | 6.812652 | 1.5 |  | t, df | t=13.25, df=5.178 |
| 26/358 | 4/500 | 7.26257 | 0.8 |  |  |  |
|  |  |  |  |  |  |  |
| **Ly6G data:-** |  |  |  |  |  |  |
| Wild Type | dKO | Wt % | dko% |  | **Unpaired t test Ly6G in wt_treated vs dKO_treated** |  |
| 48/351 | 14/356 | 13.67521 | 3.932584 |  | P value | 0.0363 |
| 39/417 | 6/217 | 9.39759 | 2.764977 |  | P value summary | * |
| 17/52 | 20/457 | 32.69231 | 4.376368 |  | Significantly different (P < 0.05)? | Yes |
| 28/150 | 17/411 | 18.66667 | 4.136253 |  | One- or two-tailed P value? | Two-tailed |
| 37/350 | 22/514 | 10.57143 | 4.280156 |  | t, df | t=3.084, df=4.038 |
|  |  |  |  |  |  |  |
| **F4/80 data:-** |  |  |  |  |  |  |
| Wild type | dko | Wt% | dko% |  | **Unpaired t test F4/80 in wt_treated vs dKO_treated** |  |
| 37/700 | 12/863 | 5.285714 | 1.390498 |  | P value | 0.0363 |
| 67/710 | 15/753 | 9.43662 | 1.992032 |  | P value summary | * |
| 55/546 | 8/400 | 10.07326 | 2 |  | Significantly different (P < 0.05)? | Yes |
| 33/187 | 11/543 | 17.64706 | 2.025783 |  | One- or two-tailed P value? | Two-tailed |
|  |  |  |  |  | t, df | t=3.084, df=4.038 |
