# Supplementary material for: A hepatocyte-specific transcriptional program driven by Rela and Stat3 exacerbates experimental colitis in mice by modulating bile synthesis
Source: eLife. 2024 Aug 13;12:RP93273. doi: 10.7554/eLife.93273 (PMC11321761; doi:10.7554/eLife.93273)
Supplement: Figure 5—source data 1. [file elife-93273-fig5-data1.docx]

| **Disease activity index** |  |  |  |  |  |  |  |  |  |  |  |  |  |  |  |  |
| --- | --- | --- | --- | --- | --- | --- | --- | --- | --- | --- | --- | --- | --- | --- | --- | --- |
| **Days** | **WT DSS** | | | | **WT+CDCA+DSS** | | | | **KO DSS** | | | | **KO+CDCA+DSS** | | | |
| **1** | 0 | 0 | 0 | 0 | 0 | 0 | 0 | 0 | 0 | 0 | 0 | 0 | 0 | 0 | 0 | 0 |
| **2** | 0 | 0 | 0 | 0 | 0 | 0 | 0 | 0 | 0 | 0 | 0 | 0 | 0 | 0 | 0 | 0 |
| **3** | 0 | 0 | 0 | 0 | 0 | 0 | 0 | 0 | 0 | 0 | 0 | 0 | 0 | 0 | 0 | 0 |
| **4** | 2 | 1 | 2 | 1 | 2 | 1 | 2 | 1 | 0 | 0 | 1 | 0 | 2 | 1 | 2 | 1 |
| **5** | 2 | 2 | 3 | 2 | 2 | 2 | 3 | 2 | 0 | 0 | 1 | 0 | 2 | 2 | 2 | 2 |
| **6** | 2 | 3 | 3 | 3 | 3 | 3 | 3 | 3 | 1 | 0 | 1 | 1 | 2 | 2 | 2 | 3 |
