# Supplementary material for: A hepatocyte-specific transcriptional program driven by Rela and Stat3 exacerbates experimental colitis in mice by modulating bile synthesis
Source: eLife. 2024 Aug 13;12:RP93273. doi: 10.7554/eLife.93273 (PMC11321761; doi:10.7554/eLife.93273)
Supplement: Figure 5—source data 2. [file elife-93273-fig5-data2.docx]

| **Colon Length** |  |  |  |  |  |  |
| --- | --- | --- | --- | --- | --- | --- |
| **Water** | **Water+CDCA** | **DSS** | **DSS+CDCA** |  |  |  |
| 7.4 | 5.2 | 6.5 | 5.3 |  | ANOVA summary |  |
| 7.1 | 6.2 | 6.4 | 4.8 |  | F | 27.99 |
| 7.2 | 5.6 | 7 | 4.7 |  | P value | <0.0001 |
| 6.9 | 6.2 | 6.9 |  |  | P value summary | **** |
|  |  |  |  |  | Significant diff. among means (P < 0.05)? | Yes |
|  |  |  |  |  | R squared | 0.8842 |
|  |  |  |  |  |  |  |
|  |  |  |  |  |  |  |
