# Supplementary material for: A hepatocyte-specific transcriptional program driven by Rela and Stat3 exacerbates experimental colitis in mice by modulating bile synthesis
Source: eLife. 2024 Aug 13;12:RP93273. doi: 10.7554/eLife.93273 (PMC11321761; doi:10.7554/eLife.93273)
Supplement: Figure 5—source data 3. [file elife-93273-fig5-data3.docx]

| **RT-qPCR** |  |  |  |  |  |  |  |
| --- | --- | --- | --- | --- | --- | --- | --- |
| **tj1** | **CON** | **CDCA** | **DSS** | **DSS+CDCA** |  | **ANOVA summary** |  |
|  | 1.00 | 0.70 | 0.74 | 0.38 |  | F | 29.52 |
|  | 1.00 | 0.81 | 0.80 | 0.56 |  | P value | <0.0001 |
|  | 1.00 | 0.79 | 0.75 | 0.29 |  | P value summary | **** |
|  | 1.00 | 0.60 |  | 0.53 |  | Significant diff. among means (P < 0.05)? | Yes |
|  |  |  |  |  |  | R squared | 0.8895 |
|  |  |  |  |  |  |  |  |
|  |  |  |  |  |  |  |  |
| **occ** | **CON** | **CDCA** | **DSS** | **DSS+CDCA** |  | **ANOVA summary** |  |
|  | 1.00 | 1.44 | 2.03 | 0.93 |  | F | 11.86 |
|  | 1.00 | 1.24 | 1.55 | 0.65 |  | P value | 0.0009 |
|  | 1.00 | 1.33 | 1.25 | 0.48 |  | P value summary | *** |
|  | 1.00 | 1.87 |  | 0.44 |  | Significant diff. among means (P < 0.05)? | Yes |
|  |  |  |  |  |  | R squared | 0.7639 |
|  |  |  |  |  |  |  |  |
|  |  |  |  |  |  |  |  |
| **muc2** | **CON** | **CDCA** | **DSS** | **DSS+CDCA** |  | **ANOVA summary** |  |
|  | 1.00 | 1.42 | 1.50 | 0.82 |  | F | 4.89 |
|  | 1.00 | 3.64 | 1.70 | 0.97 |  | P value | 0.0213 |
|  | 1.00 | 1.44 | 1.14 | 0.66 |  | P value summary | * |
|  | 1.00 | 2.37 |  | 0.81 |  | Significant diff. among means (P < 0.05)? | Yes |
|  |  |  |  |  |  | R squared | 0.5715 |
|  |  |  |  |  |  |  |  |
