# Supplementary material for: A hepatocyte-specific transcriptional program driven by Rela and Stat3 exacerbates experimental colitis in mice by modulating bile synthesis
Source: eLife. 2024 Aug 13;12:RP93273. doi: 10.7554/eLife.93273 (PMC11321761; doi:10.7554/eLife.93273)
Supplement: Figure 5—source data 4. [file elife-93273-fig5-data4.docx]

| **RT-qPCR** |  |  |  |  |  |  |  |
| --- | --- | --- | --- | --- | --- | --- | --- |
| **tnfa** | **CON** | **CDCA** | **DSS** | **DSS+CDCA** |  | **ANOVA summary** |  |
|  | 1.00 | 3.07 | 12.91 | 10.47 |  | F | 9.385 |
|  | 1.00 | 4.01 | 9.96 | 9.54 |  | P value | 0.0023 |
|  | 1.00 | 4.39 | 10.73 | 28.72 |  | P value summary | ** |
|  | 1.00 | 3.01 |  | 20.43 |  | Significant diff. among means (P < 0.05)? | Yes |
|  |  |  |  |  |  | R squared | 0.7191 |
|  |  |  |  |  |  |  |  |
| **il1b** | **CON** | **CDCA** | **DSS** | **DSS+CDCA** |  | **ANOVA summary** |  |
|  | 1.00 | 2.73 | 8.94 | 17.30 |  | F | 13.38 |
|  | 1.00 | 1.59 | 6.78 | 22.36 |  | P value | 0.0005 |
|  | 1.00 | 2.56 | 6.86 | 42.66 |  | P value summary | *** |
|  | 1.00 | 1.53 |  | 19.08 |  | Significant diff. among means (P < 0.05)? | Yes |
|  |  |  |  |  |  | R squared | 0.7849 |
|  |  |  |  |  |  |  |  |
| **il6** | **CON** | **CDCA** | **DSS** | **DSS+CDCA** |  | **ANOVA summary** |  |
|  | 1.00 | 2.03 | 13.66 | 73.51 |  | F | 52.9 |
|  | 1.00 | 1.37 | 14.51 | 51.42 |  | P value | <0.0001 |
|  | 1.00 | 6.88 | 28.12 | 54.61 |  | P value summary | **** |
|  | 1.00 | 3.09 |  | 47.39 |  | Significant diff. among means (P < 0.05)? | Yes |
|  |  |  |  |  |  | R squared | 0.9352 |
