# Supplementary material for: A hepatocyte-specific transcriptional program driven by Rela and Stat3 exacerbates experimental colitis in mice by modulating bile synthesis
Source: eLife. 2024 Aug 13;12:RP93273. doi: 10.7554/eLife.93273 (PMC11321761; doi:10.7554/eLife.93273)
Supplement: Figure 5—figure supplement 1—source data 1. [file elife-93273-fig5-figsupp1-data1.docx]

| CDCA levels |  |  |  |  |
| --- | --- | --- | --- | --- |
| Control | Treated |  |  |  |
| 50.723 | 230.9654 |  | **P value for Dinor-chenodeoxycholic acid control vs IBD patients** |  |
| 39.8166 | 465.2292 |  | P value | 0.0355 |
| 23.98585 | 163.9141 |  | P value summary | * |
| 68.21435 | 136.3987 |  | Significantly different (P < 0.05)? | Yes |
|  |  |  | One- or two-tailed P value? | Two-tailed |
