# Supplementary material for: A hepatocyte-specific transcriptional program driven by Rela and Stat3 exacerbates experimental colitis in mice by modulating bile synthesis
Source: eLife. 2024 Aug 13;12:RP93273. doi: 10.7554/eLife.93273 (PMC11321761; doi:10.7554/eLife.93273)
Supplement: Figure 5—figure supplement 1—source data 2. [file elife-93273-fig5-figsupp1-data2.docx]

| **Colon Length** |  |
| --- | --- |
| **Water** | **Water+CDCA** |
| 7.4 | 5.2 |
| 7.1 | 6.2 |
| 7.2 | 5.6 |
| 6.9 | 6.1 |
| 7.4 | 5.7 |
